# Enantio‐ and Diastereoselective Synthesis of Homopropargyl Amines by Copper‐Catalyzed Coupling of Imines, 1,3‐Enynes, and Diborons

**DOI:** 10.1002/anie.201915191

**Published:** 2020-02-11

**Authors:** Srimanta Manna, Quentin Dherbassy, Gregory J. P. Perry, David J. Procter

**Affiliations:** ^1^ Department of Chemistry The University of Manchester Oxford Road Manchester M13 9PL UK

**Keywords:** asymmetric catalysis, borylative coupling, copper, 1,3-enynes, homopropargyl amines

## Abstract

An efficient, enantio‐ and diastereoselective, copper‐catalyzed coupling of imines, 1,3‐enynes, and diborons is reported. The process shows broad substrate scope and delivers complex, chiral homopropargyl amines; useful building blocks on the way to biologically‐relevant compounds. In particular, functionalized homopropargyl amines bearing up to three contiguous stereocenters can be prepared in a single step.

## Graphical abstract

**The enantio‐ and diastereoselective**, copper‐catalyzed three‐component coupling of imines, 1,3‐enynes, and diborons delivers complex, chiral homopropargyl amines; useful building blocks on the way to biologically‐ and medicinally‐relevant compounds. In particular, functionalized homopropargyl amines bearing up to three contiguous stereocenters can be prepared in a single step.

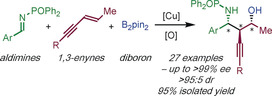

Chiral homopropargyl amines are used in the synthesis of many natural products, and biologically and medicinally important molecules.^1^, ^2^, ^3^ Most methods for homopropargyl amine synthesis involve the union of imines and propargylic or allenic substrates. These methods deliver racemic homopropargylic amines^4^ and asymmetric variants selectively generate products with a stereocenter adjacent to the amino group (Scheme 1 A). In general, these methods use a transition metal catalyst and chiral ligand, or imines bearing a chiral auxiliary.^5^ Constructing homopropargyl amines with more than one stereocenter, particularly if the stereocenters are adjacent, is a more challenging process (Scheme 1 B), few procedures address this goal and these require difficult‐to‐access reagents and/or chiral auxiliaries.^6^ Thus, a general preparation of chiral homopropargylic amines, bearing multiple stereocenters, from readily‐accessible substrates, remains an important challenge.

**Scheme 1:**
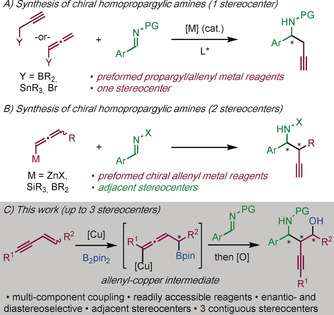
Enantioselective transition metal‐catalyzed nucleophilic addition to imines for the synthesis of homopropargyl amines. PG=protecting group; X=PG or chiral auxiliary; Pin=pinacolato.

Copper‐catalyzed borylative transformations are a powerful method for uniting unsaturated hydrocarbons and electrophiles.^7^ Importantly, these methods produce densely functionalized, chiral molecules from simple, achiral substrates, and use cheap and non‐toxic transition metal catalysts. We and others have described efficient routes to amines through the multicomponent coupling of imines with hydrocarbon pro‐nucleophiles and boron reagents.^8^, ^9^, ^10^ Krische pioneered the use of enynes as hydrocarbon pro‐nucleophiles in transition metal‐catalyzed transformations,^11^, ^12^, ^13^ however, in both reductive and borylative coupling, the asymmetric union of imines and enynes remains an unmet challenge.^14^

We envisaged a new approach to homopropargyl amines involving the copper‐catalyzed enantio‐ and diastereoselective multicomponent coupling of imines, enynes, and diboron reagents (Scheme 1 C). Furthermore, through routine oxidation of the carbon–boron bond, biologically relevant 1,3‐amino alcohols would be accessible.^15^ Herein, we disclose an efficient method for obtaining functionalized chiral homopropargyl amines, bearing up to three stereocenters and various synthetic handles (amino, boron, alkynyl), using an inexpensive, non‐toxic, and readily‐available copper catalyst, and a commercial phosphine ligand.

We explored the copper‐catalyzed coupling of imine **1 a**, 1,3‐enyne **2 a** and bis(pinacolato)diboron (B_2_pin_2_). Using CuCl and (*S*,*S*)‐Ph‐BPE (**L1**), the desired product **3 a′** (PG=PMP) was obtained in 70 % yield and the major diastereoisomer was found to have an *ee* of 53 % (Table 1, entry 1). After screening reaction conditions with imine **1 a**, we turned our attention to *N*‐phosphinoylimine **1 b**. With this imine, the enantioselectivity and diastereoselectivity of the reaction increased (89 % *ee*, >95:5 dr), however, only 37 % yield of the desired product was obtained (entry 2). By screening the copper salt, base, and solvent, we found that the use of CuOAc, KOMe, and THF was optimal; **3 a** was obtained in high yield, with excellent diastereoselectivity and enantioselectivity (entry 3).^16^ X‐ray crystallographic analysis of **3 d** revealed the relative and absolute stereochemistry of the product.^16^ Other diboron reagents are applicable in the reaction; the use of bis(neopentyl glycolato)diboron (B_2_neo_2_) gave **3 a** in moderate yield but with high diastereo‐ and enantiocontrol (entry 11).

**Table 1:** Screening of reaction conditions^[a]^ 
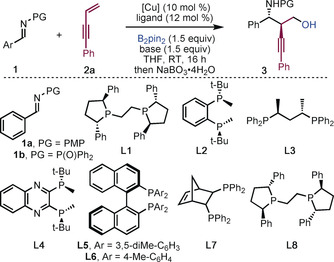

Entry	Imine	Ligand	Cu^I^/base	dr	**3** Yield/*ee* ^[b]^ [%]
1	**1 a**	**L1**	CuCl/NaO*t*Bu	87:13	70/53^[c]^
2	**1 b**	**L1**	CuOAc/NaO*t*Bu	>95:5	37/89
3	**1 b**	**L1**	CuOAc/KOMe	>95:5	92/99
4	**1 b**	**L2**	CuOAc/KOMe	–	–
5	**1 b**	**L3**	CuOAc/KOMe	–	–
6	**1 b**	**L4**	CuOAc/KOMe	–	–
7	**1 b**	**L5**	CuOAc/KOMe	>95:5	56/34
8	**1 b**	**L6**	CuOAc/KOMe	–	–
9	**1 b**	**L7**	CuOAc/KOMe	88:12	37/16
10	**1 b**	**L8**	CuOAc/KOMe	>95:5	88/96^[d]^
11	**1 b**	**L1**	CuOAc/KOMe	90:10	56/92^[e]^

[a] Reaction conditions: **1** (0.2 mmol), **2 a** (0.3 mmol), B_2_pin_2_ (0.3 mmol), base (0.3 mmol), Cu^I^ (10 mol %), ligand (12 mol %) in THF (2.0 mL) at RT for 16 h under nitrogen. The diastereoselectivity was determined by ^1^H NMR analysis of the crude product mixtures. NMR yields are given. [b] The *ee* values were determined by chiral HPLC after oxidation. [c] The *ee* values were measured by chiral HPLC analysis of the boron‐containing product. [d] The enantiomer of **3 a** was formed. [e] B_2_neo_2_ (0.3 mmol) was used. THF=tetrahydrofuran. PMP=4‐methoxyphenyl; Neo=neopentyl glycolato.

The reaction tolerated electron‐donating and electron‐withdrawing substituents on the aryl ring of the aldimine; the desired products were obtained in high yield and with excellent enantio‐ and diastereoselectivity (Scheme 2). For example, electron‐rich aldimines were well tolerated in the reaction and only a slight decrease in enantioselectivity was observed when an *ortho*‐methoxy substituent was used (**3 b**). Similarly, imines bearing electron‐withdrawing groups at the *ortho*‐, *meta*‐, and *para*‐positions (**3 e**–**3 j**), including halogen (**3 e**–**3 g**, **3 i**), ester (**3 h**), and trifluoromethyl (**3 j**) substituents, also performed well. The reaction also proceeded efficiently when heteroaryl‐aldimines were used (**3 l**–**3 o**). The reaction could be executed on a gram scale without significant detriment to the yield or selectivity (**3 a**). Attempts to use an aliphatic aldimine in the process were unsuccessful (See Supporting Information).

**Scheme 2:**
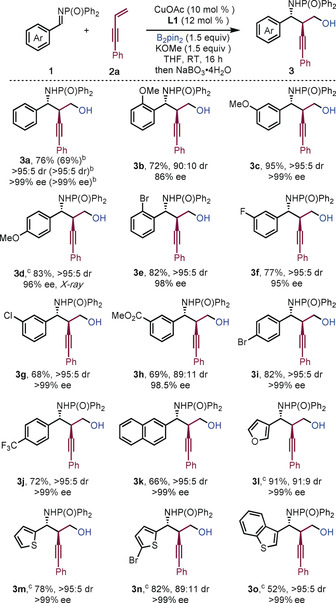
Scope with respect to the imine. [a] Reaction conditions: See Table 1. Yields of isolated products are given. [b] Values in parentheses indicate the result of a 1 g scale reaction. [c] 0 °C in MTBE. MTBE=methyl‐*t*‐butyl ether.

Aryl‐substituted 1,3‐enynes bearing electron‐donating groups delivered the corresponding products in good to excellent yield and with high enantioselectivities (Scheme 3, **4 a**–**4 d**). Mixed results were obtained when using electron‐deficient enynes; for example, whereas the bromo‐substituted product **4 e** was prepared in good yield, with high selectivity, an ester substituent severely affected the efficiency of the coupling (**4 f**). The use of an alkyl substituted enyne gave **4 h** in low yield but with high enantiocontrol. Substitution at the terminal position of the alkene was investigated: *E*‐enynes gave products **6 b**–**6 d** in good to high yield, with good diastereoselectivity and excellent enantioselectivity. The structure of **6 b** was confirmed by X‐ray crystallography.^16^ The use of *Z*‐enyne **5 a**‐*Z* gave alternative diastereoisomeric product **6 a**. Thus, the process delivers amino alcohols bearing three contiguous stereocenters with essentially complete enantiocontrol.

**Scheme 3:**
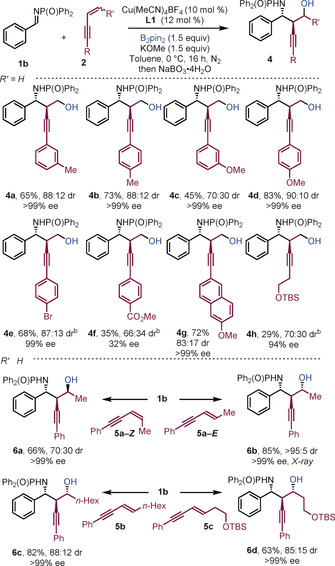
Scope with respect to 1,3‐enyne. [a] Reaction conditions: **1 b** (0.2 mmol), **2** (0.3 mmol), B_2_pin_2_ (0.3 mmol), KOMe (0.3 mmol), Cu(MeCN)_4_BF_4_ (10 mol %), (*S*,*S*)‐Ph‐BPE **L1** (12 mol %) in toluene (2.0 mL) at 0 °C for 16 h under nitrogen. Yields of isolated products. [b] THF at RT with CuOAc.

Amine **3 a** was readily hydrogenated, to give the branched chain alkane **7 a**, and the β‐amino acid derivative **7 b** was accessed by oxidation of **7 a** (Scheme 4). Biologically‐ and medicinally‐relevant *N*‐containing heterocycles were also prepared, for example, azetidine **7 c**, or 2,3‐dihydropyrrole **7 d** through π‐activation of the alkyne bond using a Au–Ag catalyst system.^17^ The phosphinoyl group could be removed to reveal the free amine **7 e**,9a which was subjected to urethanation to give oxazinone **7 f**.

**Scheme 4:**
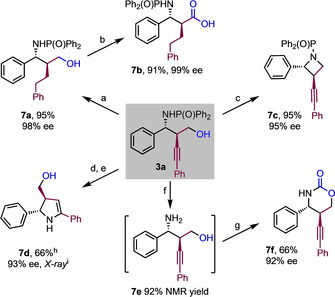
Manipulation of product **3 a**. [a] Pd/C (10 mol %), H_2_ (1 atm), MeOH, 40 °C, 24 h. [b] RuCl_3_ (5 mol %), NaIO_4_ (1.5 equiv), CCl_4_:MeCN:H_2_O=1:1:1.2, 3 h, RT. [c] TsCl (1.5 equiv), NaH (6 equiv), THF, 40 °C, 8 h. [d] From borylated/non‐oxidized form of **3 a**: Ph_3_PAuCl (10 mol %), AgOTf (10 mol %), DCE, 8 h, 80 °C. [e] NaBO_3_⋅4 H_2_O (5 equiv), THF:H_2_O=1:1, 6 h, RT. [f] 4 n HCl, MeOH, RT, 3 h, RT. [g] Triphosgene (1.0 equiv), Et_3_N (2 equiv), THF, 3 h, 0 °C. [h] 4:1 Mixture of tautomers. [i] X‐ray of minor tautomer of **7 d**.

Regioselective borocupration provides intermediate **A** (1),12a, 13a which is proposed to undergo propargyl‐to‐allenyl isomerization to **B** (2) (Scheme 5 A).12d We propose that intermediate **B** is the major allenyl–copper isomer in the reaction.12d Coupling of the allenyl–copper intermediate **B** with imine **1 b** (**C**_***re***_, 3) gives chiral homopropargylic amine **D** and closes the catalytic cycle (4).12b–12d Scheme 5 B provides an explanation for the *anti*‐diastereoselectivity observed in the reaction. Coupling (3) between allenyl intermediate **B** and imine **1 b** can occur from attack at either the *re* face (**C**_***re***_) or the *si* face (**C**_***si***_) of the imine. However, reaction at the *si* face (**C**_***si***_) incurs unfavorable interactions between the *N*‐phosphinoyl group and the ‐CH_2_Bpin group and is disfavored.

**Scheme 5:**
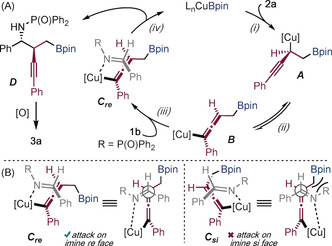
Proposed catalytic cycle for the enantioselective coupling.

In conclusion, a highly enantio‐ and diastereoselective coupling of imines, 1,3‐enynes, and diborons using an inexpensive copper catalyst and a commercial ligand, delivers chiral homopropargyl amines with up to three contiguous stereocenters. The products provide access to important targets, including β‐amino acids and *N*‐heterocycles.

## Conflict of interest

The authors declare no conflict of interest.

## Supporting information

As a service to our authors and readers, this journal provides supporting information supplied by the authors. Such materials are peer reviewed and may be re‐organized for online delivery, but are not copy‐edited or typeset. Technical support issues arising from supporting information (other than missing files) should be addressed to the authors.

Supplementary
