# Supplementary material for: Enantio‐ and Diastereoselective Synthesis of Homopropargyl Amines by Copper‐Catalyzed Coupling of Imines, 1,3‐Enynes, and Diborons
Source: Angew Chem Int Ed Engl. 2020 Feb 11;59(12):4879–82. doi: 10.1002/anie.201915191 (PMC7383811; doi:10.1002/anie.201915191)
Supplement: Supplementary file 1 — Supplementary [file ANIE-59-4879-s001.pdf]

Angewandte Chemie
Eine Zeitschrift der Gesellschaft Deutscher Chemiker

Supporting Information

Enantio- and Diastereoselective Synthesis of Homopropargyl Amines by Copper-Catalyzed Coupling of Imines, 1,3-Enynes, and Diborons

*Srimanta Manna, Quentin Dherbassy, Gregory J. P. Perry, and David J. Procter**

anie_201915191_sm_miscellaneous_information.pdf

Contents

**General Information**	**S3**
**General procedure A**	**S3**
**General procedure B**	**S4**
**Optimization studies**	**S5**
**Unsuccessful imines**	**S9**
**Physical data of products**	**S10**
**Product derivatization**	**S55**
**NMR spectra of synthesized compounds**	**S65**
**X-Ray structures**	**S97**
**References**	**S103**

General Information

All experiments were performed under an atmosphere of nitrogen, using anhydrous solvents, unless stated otherwise. THF was distilled from sodium / benzophenone. ^{1}H, ^{13}C, and ^{31}P NMR spectra were recorded using 400 and 500 MHz spectrometers, with chemical shift values being reported in ppm relative to residual chloroform (δ_H = 7.27 or δ_C = 77.0) as internal standards. All coupling constants (*J*) are reported in Hertz (Hz). Mass spectra were obtained using positive and negative electrospray (ES$^{\pm}$), atmospheric-pressure chemical ionization (APCI) or gas chromatography (GC) methodology. Infra-red spectra were recorded as evaporated films or neat using a FT/IR spectrometer. Column chromatography was carried out using 40 – 63 µm, 60 Å silica gel. Routine TLC analysis was carried out on aluminium sheets coated with silica gel 60 F254, 0.2 mm thickness and plates were viewed using a 254 nm ultraviolet lamp and dipped in aqueous potassium permanganate or *p*-anisaldehyde. Melting points were measured on a melting point apparatus and are uncorrected.

General procedure A for Cu-catalyzed asymmetric borylative coupling of 1, 3-enynes and imines

To a 10 mL reaction vial was added CuOAc (0.02 mmol, 2.4 mg, 10 mol %), (*S*,*S*)-Ph-BPE (0.024 mmol, 12.3 mg), KOMe (0.3 mmol, 21 mg) and THF (1.0 mL). The mixture was stirred at room temperature for 30 min before the addition of $B_2(pin)_2$ (0.3mmol, 78.0 mg). After brief stirring (10 min), 1,3-enyne (0.3-0.4 mmol) was added (via syringe if liquid) followed by the addition of phosphinoylimine (0.2 mmol), and THF (1.0 mL). The reaction mixture was then stirred for 16-24 h. After reaction completion, the resulting solution was filtered through celite, and the crude material was concentrated in vacuo. ^{1}H NMR was used to determine the NMR yield and diastereoselectivity using 1,3,5-trimethoxybenzene (0.2 mmol, 33.6 mg) as the internal standard. After NMR analysis, the solvent was removed in vacuo. Then, to the residue was added THF (2.0 mL), H_2O (2.0 mL) and $NaBO_3.4H_2O$ (1.2 mmol, 180 mg). After stirring for 6 h, the mixture was extracted by EtOAc (3 x2 0 mL). The combined organic layers were dried over Na_2SO_4 and concentrated under vacuum. Column chromatography of the crude material with petroleum/EtOAc (60:30-20:80) afforded the target compounds.

General procedure B for Cu-catalyzed asymmetric borylative coupling of 1, 3-enynes and imines

To a 10 mL reaction vial was added $Cu(MeCN)_4BF_4$ (0.02 mmol, 6.29 mg, 10 mol %), (*S*,*S*)-Ph-BPE (0.024 mmol, 12.3 mg), KOMe (0.3 mmol, 21 mg) and toluene (1.0 mL). The mixture was then stirred at room temperature for 30 min before the addition of $B_2(pin)_2$ (0.3mmol, 78.0 mg). After brief stirring (10 min), 1,3-enyne (0.3-0.4 mmol) was added (via syringe if liquid), before the addition of phosphinoylimine (0.2 mmol), and toluene (1.0 mL). The reaction mixture was then cooled to 0 ˚C and stirred for 16-24 h. After reaction completion, the resulting solution was filtered through celite, and the crude material was concentrated in vacuo. ^1H NMR was used to determine the NMR yield and diastereoselectivity using 1,3,5-trimethoxybenzene (0.2 mmol, 33.6 mg) as the internal standard. After NMR analysis, the solvent was removed in vacuo. Then, to the residue was added THF (2.0 mL), H_2O (2.0 mL) and $NaBO_3.4H_2O$ (1.2 mmol, 180 mg). After stirring for 6 h, the mixture was extracted by EtOAc (3 x 20 mL). The combined organic layers were dried over Na_2SO_4 and concentrated under vacuum. Column chromatography of the crude material with petroleum/EtOAc (60:30-20:80) afforded the target compounds.

Optimization studies

Table 1. Optimization studies with PMP imine

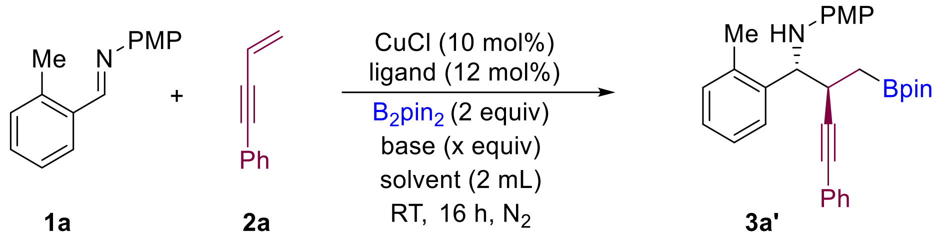

1a **2a** **3a'**

Entry	Ligand	Base (x equiv)	Solvent	NMR yield (%)	d.r.	ee (%)
1	**L1**	NaO*t*Bu (2)	THF	70	87:13	53
2	**L1**	LiO*t*Bu (3)	THF:DME(1:1)	30	90:10	82
3	**L2**	NaO*t*Bu (2)	THF	-	-	-
4	**L3**	NaO*t*Bu (2)	THF	-	-	-
5	**L4**	NaO*t*Bu (2)	THF	-	-	-
6	**L1**	NaO*t*Bu (2)	THF	trace	-	-

Reaction conditions: **1a** (0.2 mmol), **2a** (0.3 mmol), $(Bpin)_2$ (0.3 mmol), CuCl (10 mol bisphosphine ligand (12 mol%) in THF (2.0 mL) at RT for 16 h under argon. The diastereoselectivity and yield were determined by ^{1}H NMR analysis of the crude products. The ee values were measured by chiral HPLC. Pin = pinacolato, PMP = *para*-methoxyphenyl, THF = Tetrahydrofuran, DME = Dimethoxyethane.

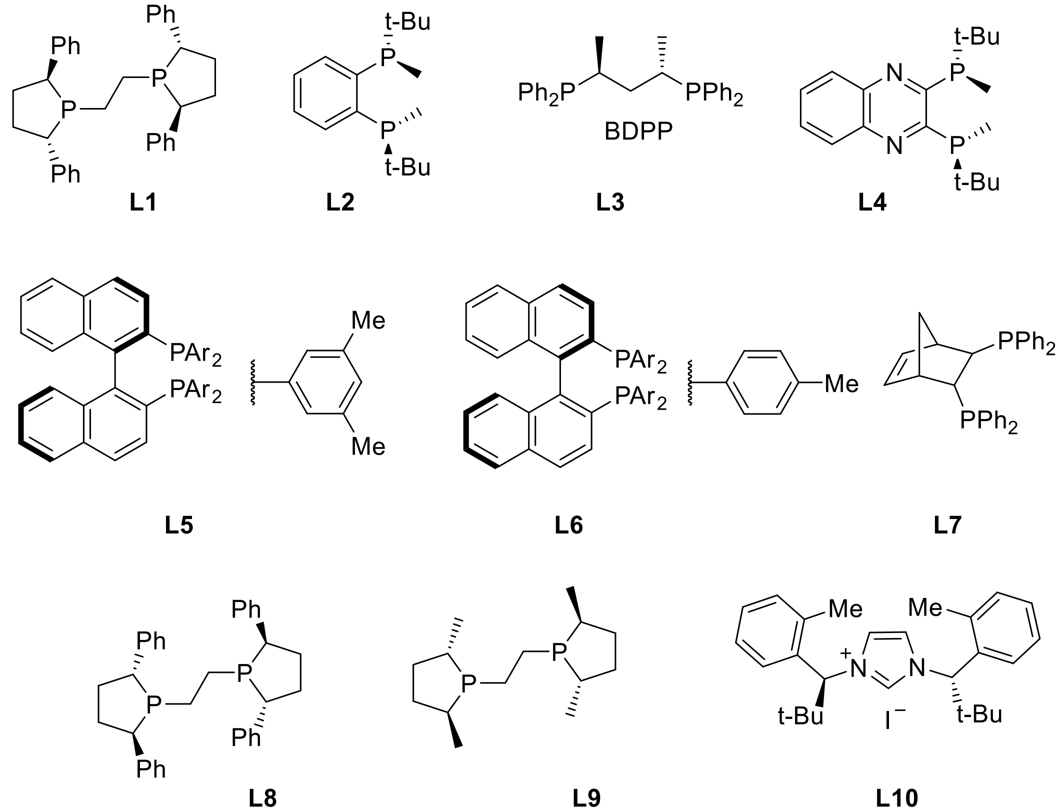

Table 2. Optimization studies with phosphinoylimine

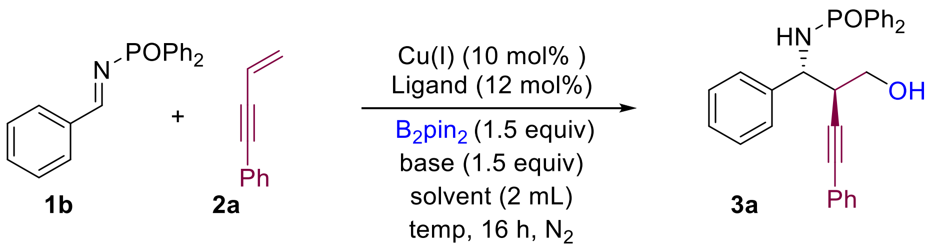

Entry	Ligand	Cu(I)	Temp (°C)	Base (1.5 equiv)	NMR yield (%)	d.r.	ee
1	**L1**	CuOAc	RT	NaOtBu	37	>95:5	89
2	**L1**	CuOAc	RT	KOtBu	-	-	-
3	**L1**	$Cu(MeCN)_4BF_4$	RT	KOMe	78	>95:5	82
4[a]	**L1**	CuOAc	0 °C	KOMe	88	>95:5	88
5[b,c]	**L1**	CuOAc	0 °C	KOMe	88	>95:5	99
**6**	**L1**	**CuOAc**	**RT**	**KOMe**	**92**	**>95:5**	**99**
7	**L2**	CuOAc	RT	KOMe	-	-	-
8	**L3**	CuOAc	RT	KOMe	-	-	-
9	**L4**	CuOAc	RT	KOMe	-	-	-
10	**L5**	CuOAc	RT	NaOtBu	56	>95:5	34
11	**L6**	CuOAc	RT	NaOtBu	-	-	-
12	**L7**	CuOAc	RT	KOMe	37	88:12	16
13	**L8**	CuOAc	RT	KOMe	88	>95:5	-96
14	**L9**	CuOAc	RT	KOMe	trace	-	-
15	**L10**	CuOAc	RT	KOMe	trace	-	-
15[d]	**L1**	CuOAc	RT	KOMe	-	-	-
16[e]	**L1**	CuOAc	RT	KOMe	53	>95:5	99
17	**L1**	$Cu(MeCN)_4PF_6$	RT	KOMe	76	>95:5	99

Reaction conditions: **1b** (0.2 mmol), **2a** (0.3 mmol), $(Bpin)_2$ (0.3 mmol), Cu(I) (10 mol %), bisphosphine ligand (12 mol%) in THF (2.0 mL) at RT for 16 h under argon. The diastereoselectivity and yield were determined by ^{1}H NMR analysis of the crude products. The ee values were measured by chiral HPLC after oxidation of the boron-containing product with $NaBO_3{\bullet}4H_2O$. [a] 11 mol% Ligand was used. [b] MTBE as solvent was used. [c] Reaction was carried out in methyl-*tert*-butyl ether (MTBE) as solvent. [d] Tosyl protected aldimine was used. [e] 5 mol% CuOAc and 6 mol% ligand was used.

Table 3. Optimization of diboron sources

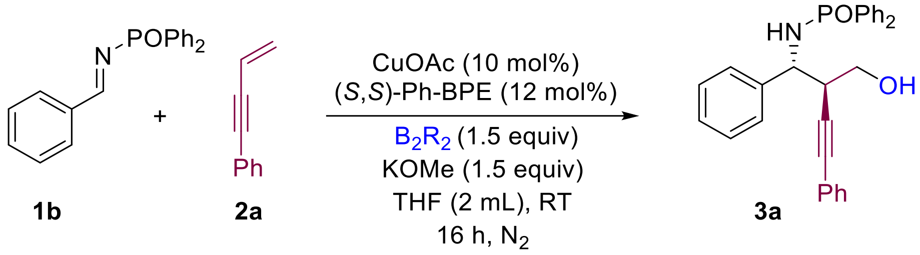

Entry	B_2R_2	Temp (°C)	NMR yield (%)	d.r.	ee (%)
1	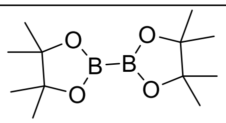	RT	92	>95:5	99
2	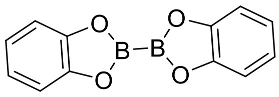	RT	trace	-	-
3	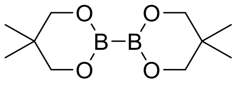	RT	56	90:10	92

Reaction conditions: **1b** (0.2 mmol), **2a** (0.3 mmol), B_2R_2 (0.3 mmol), CuOAc (10 mol %), and (*S*,*S*)-Ph-BPE (12 mol%) in THF (2.0 mL) at RT for 16 h under argon. The diastereoselectivity and yield were determined by ^{1}H NMR analysis of the crude products. The ee values were measured by chiral HPLC after oxidation of the boron-containing product with $NaBO_3{\bullet}4H_2O$.

Table 4. Optimization for 1,3-enyne derivative

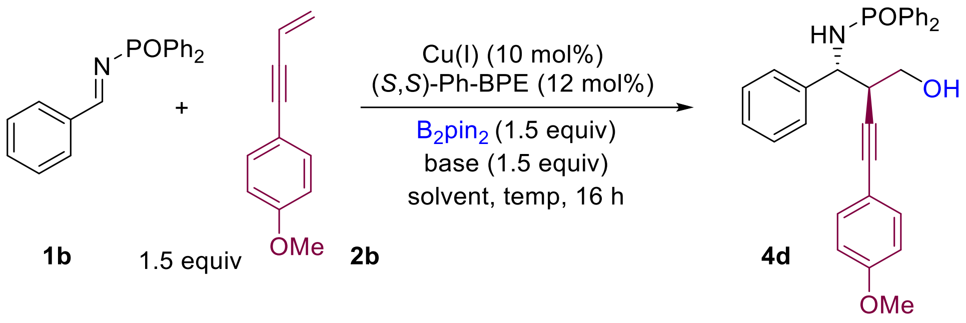

Entry	Solvent	Temp	Base	Cu(I)	d.r.	ee (%)
1	THF	RT	NaOMe	CuOAc	-	-
2	MTBE	RT	LiOMe	CuOAc	71:29	97
3	MTBE	0˚C	KOMe	CuOAc	66:34	99
**5**	**Toluene**	0˚C	**KOMe**	**$Cu(MeCN)_4BF_4$**	**90:10**	**99**
6	THF	RT	KOMe	CuOAc	75:25	99

Reaction conditions: **1b** (0.2 mmol), **2b** (0.3 mmol), $(Bpin)_2$ (0.3 mmol), Cu(I) (10 mol %), (*S*,*S*)-Ph-BPE (12 mol%) in solvent (2.0 mL) at T for 16 h under argon. The diastereoselectivity was determined by ^{1}H NMR analysis of the crude products. The ee values were measured by chiral HPLC after oxidation of the boron-containing product with $NaBO_3$•$4H_2O$. MTBE = methyl-*tert*-butyl ether.

Unsuccessful substrates

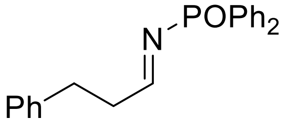

1c

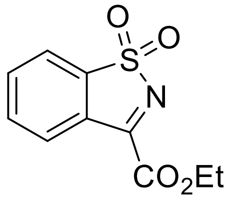

1d

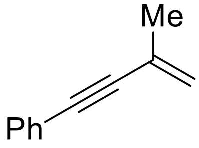

2c

Physical data of products

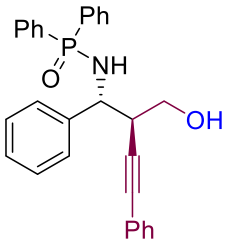

***N*-((1*R*,2*S*)-2-(Hydroxymethyl)-1,4-diphenylbut-3-yn-1-yl)-*P*,*P*-diphenylphosphinic amide (3a)**

Compound **3a** was prepared according to general procedure A. The title compound was isolated by column chromatography (hexane: ethyl acetate, 60:40-40:60) as a white amorphous solid (68.5 mg, 0.152 mmol, 76%). For gram scale reaction obtained 933.5 mg, 20.07 mmol in 69% yield using 5 mol% CuOAc and 6 mol% (*S*,*S*)-BPE.M.p: 163-167 ˚C ($CHCl_3$).

^{1}H NMR (400 MHz, 400 MHz, $CDCl_3$) δ ppm 7.86 – 7.76 (m, 2H, ArC*H* x 2), 7.72 – 7.63 (m, 2H, ArC*H* x 2), 7.52 – 7.44 (m, 2H, ArC*H* x 2), 7.45 – 7.37 (m, 2H, ArC*H* x 2), 7.37 – 7.27 (m, 5H, ArC*H* x 5), 7.25 – 7.21 (m, 2H, ArC*H* x 2), 7.21 – 7.12 (m, 5H, ArC*H* x 5), 5.01 (ddd, *J* = 8.0, 5.9, 1.7 Hz, 1H, O*H*), 4.5 – 4.4 (m, 1H, NHC*H*), 4.18 (ddd, *J* = 11.7, 6.0, 3.7 Hz, 1H, CH_2OH), 3.94 (ddd, *J* = 11.4, 9.2, 2.0 Hz, 1H, N*H*), 3.78 (ddd, *J* = 11.7, 8.3, 4.7 Hz, 1H, CH_2OH), 3.21 – 3.08 (m, 1H, PhCCC*H*).

^{13}C NMR (101 MHz, $CDCl_3$) δ ppm 141.9 (d, *J* = 5.8 Hz, Ar*C*), 133.1 (d, *J* = 10.0 Hz, Ar*C*H, x 2), 132.4 (d, *J* = 2.9 Hz, Ar*C*H), 132.4 (d, *J* = 2.8 Hz, Ar*C*H), 132.3 (d, *J* = 127.6 Hz, Ar*C*), 131.7 (Ar*C*H x 2), 131.6 (d, *J* = 9.8 Hz, Ar*C*H), 130.8 (d, *J* = 133.4 Hz, Ar*C*), 128.8 (d, *J* = 12.7 Hz, Ar*C*H x 2), 128.5 (d, *J* = 12.8 Hz, Ar*C*H x 2), 128.4 (Ar*C*H x 2), 128.1 (Ar*C*H x 2), 127.9 (Ar*C*H), 127.6 (Ar*C*H), 127.3 (Ar*C*H x 2), 123.4 (Ar*C*), 88.1 (Ph*C*≡C), 84.9 (PhC≡*C*) 62.5 (HO*C*H_2), 55.8 (NH*C*H), 44.3 (d, *J* = 2.5 Hz, PhCC*C*H).

^{31}P NMR (162 MHz, $CDCl_3$) δ ppm 25.78.

IR $ν_{max}$ (neat/cm^{-1}): 3298, 3054, 2926, 1591, 1434, 1220, 1153, 1102, 926.

HRMS : calcd for $C_{29}H_{26}NO_2PNa$ $[M + Na]^+$ 474.1593, found 474.1583.

Specific rotation: $[α]_D^{26.5}$ - 13.2 (c 1.0, CH_2Cl_2).

Enantiomeric purity of **3a** was determined by HPLC analysis in comparison with authentic racemic material (ee >99%; **OD-H** column, 90:10 hexanes: *i*-PrOH, 0.5 mL/min, 20 °C, 254 nm)

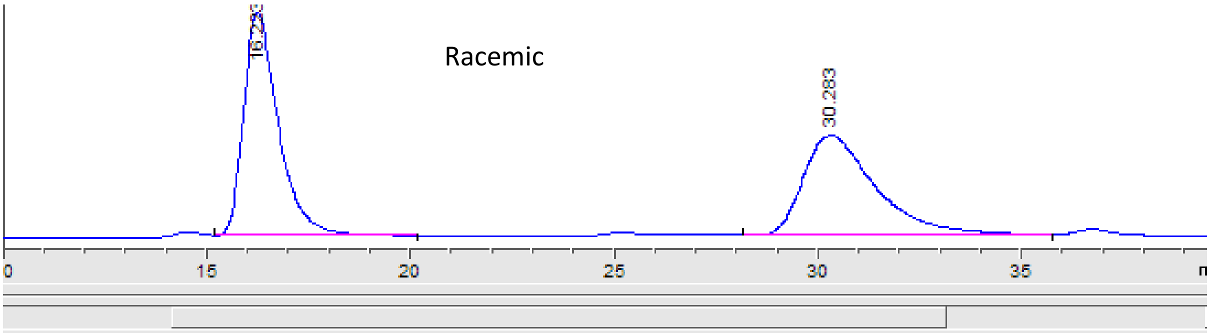

#	Time	Area	Height	Width	Area%	Symmetry
1	16.223	7746.8	131.3	0.8862	51.710	0.613
2	30.283	7234.5	58.7	1.789	48.290	0.561

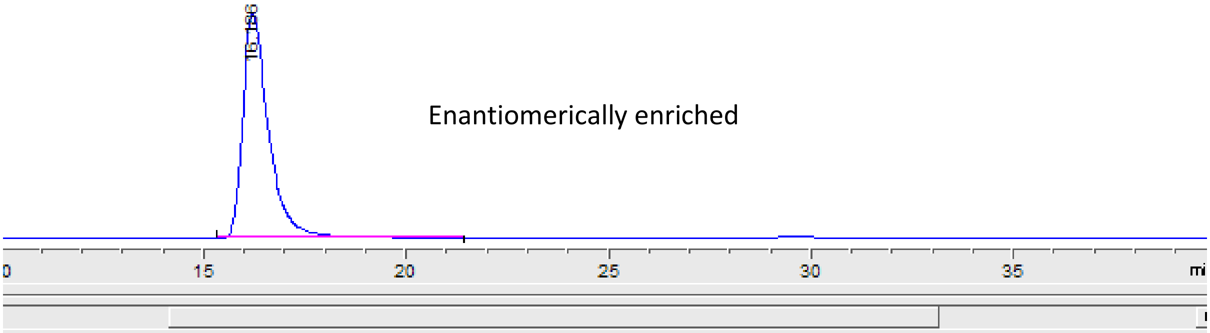

#	Time	Area	Height	Width	Area%	Symmetry
1	16.186	15761.8	355.4	0.6654	100.000	0.56

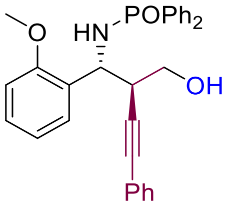

N-((1*R*,2*S*)-2-(Hydroxymethyl)-1-(2-methoxyphenyl)-4-phenylbut-3-yn-1-yl)-*P*,*P*-diphenylphosphinic amide (3b)

Compound **3b** was prepared according to general procedure A. The title compound was isolated by column chromatography (hexane: ethyl acetate, 60:40-20:80) as a white amorphous solid (69 mg, 0.144 mmol, 72%). M.p. 146-151 ˚C ($CHCl_3$).

^{1}H NMR (500 MHz, DMSO-d_6) δ ppm 7.78 – 7.66 (m, 2H, ArC*H* x 2), 7.61 – 7.51 (m, 4H, ArC*H* x 4), 7.48 (td, *J* = 7.4, 6.9, 3.7 Hz, 3H, ArC*H* x 3), 7.35 (d, *J* = 3.2 Hz, 2H, ArC*H* x 2), 7.27 – 7.20 (m, 4H, ArC*H* x 4), 7.10 (dd, *J* = 6.5, 3.1 Hz, 2H, ArC*H* x 2), 6.98 (t, *J* = 7.4 Hz, 1H, ArC*H*), 6.86 (d, *J* = 8.2 Hz, 1H, ArC*H*), 6.09 (t, *J* = 11.7 Hz, 1H, N*H*), 5.39 (dd, *J* = 7.6, 5.8 Hz, 1H, HNC*H*), 4.72 (q,

J = 11.2 Hz, 1H, O*H*), 3.95 (dt, *J* = 10.9, 5.3 Hz, 1H, HOC*H*$_2$), 3.70 (ddd, *J* = 10.8, 7.4, 2.8 Hz, 1H, , HOC*H*$_2$), 3.33 (s, 3H, OC*H*$_3$), 3.06 (ddd, *J* = 8.3, 4.9, 2.8 Hz, 1H, PhCCC*H*).

^{13}C NMR (126 MHz, DMSO-*d*$_6$) δ ppm 155.8 (Ar*C*), 133.4 (d, *J* = 6.0 Hz, Ar*C*), 132.1 (d, *J* = 9.7 Hz, Ar*C*H x 2), 131.9 (d, *J* = 128.3 Hz, Ar*C*), 131.8 (d, *J* = 125.6 Hz, Ar*C*), 131.7 (d, *J* = 2.6 Hz, Ar*C*H), 131.5 (d, *J* = 2.6 Hz, Ar*C*H), 131.4 (d, *J* = 9.7 Hz, Ar*C*H x 2), 131.0 (Ar*C*H x 2), 128.5 (d, *J* = 12.2 Hz, Ar*C*H x 2), 128.4 (Ar*C*H x 2), 128.1 (d, *J* = 12.8 Hz, Ar*C*H x 2), 128.0 (Ar*C*H), 127.9 (Ar*C*H), 127.7 (Ar*C*H), 123.3 (Ar*C*), 120.3 (Ar*C*H), 110.4 (Ar*C*H), 90.9 (PH*C*≡C), 81.9 (PhC≡*C*), 79.18 (HO*C*H$_2$), 61.3 (O*C*H$_3$), 55.0 (HN*C*H), 42.2 (PhCC*C*H).

IR ν$_{max}$ (neat/cm^{-1}): 3319, 2924, 1488, 1435, 1435, 1354, 1156, 1201, 1102, 1069, 1043, 1008, 997.

HRMS : calcd for $C_{30}H_{29}NO_3P$ [M + H]$^+$: 482.1880, found 482.1874.

Specific rotation: $[\alpha]_D^{28.6}$ - 54.5 (c 0.34, $CHCl_3$).

Enantiomeric purity of **3b** was determined by HPLC analysis in comparison with authentic racemic material (ee: 86%; **OD-H** column, 90:10 hexanes: *i*-PrOH, 1 mL/min, 20 °C, 254 nm)

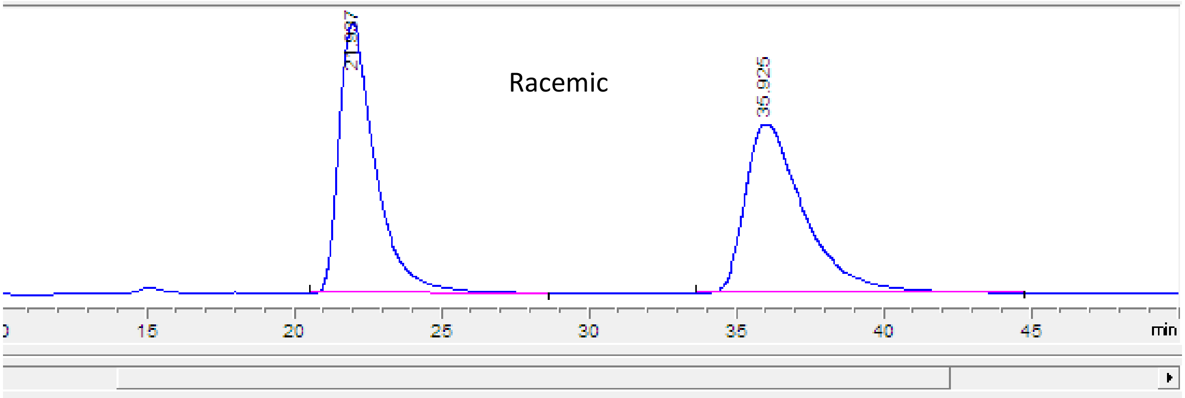

#	Time	Area	Height	Width	Area%	Symmetry
1	21.937	6752.4	78.7	1.2754	49.430	0.52
2	35.925	6908.2	49.1	1.8379	50.570	0.451

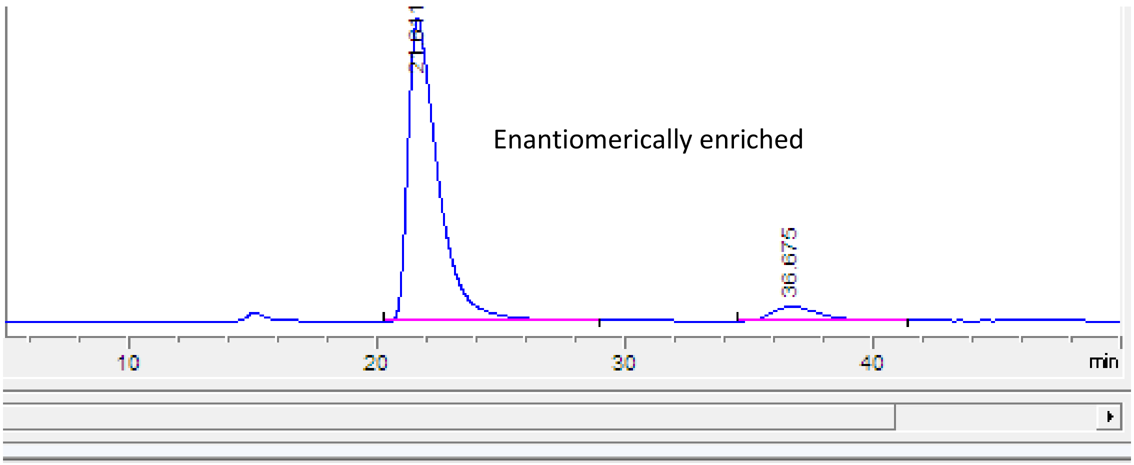

#	Time	Area	Height	Width	Area%	Symmetry
1	21.611	18068.9	214.9	1.2248	93.026	0.428
2	36.675	1354.7	10.2	1.5791	6.974	0.644

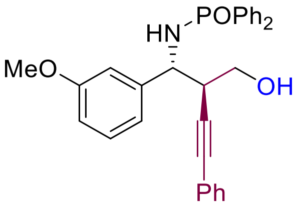

***N*-((1*R*,2*S*)-2-(Hydroxymethyl)-1-(3-methoxyphenyl)-4-phenylbut-3-yn-1-yl)-*P*,*P*-diphenylphosphinic amide (3c)**

Compound **3c** was prepared according to general procedure A. The title compound was isolated by column chromatography (hexane: ethyl acetate, 70:30-20:80) as a white amorphous solid (72.0 mg, 0.190 mmol, 95%). M.p.: 135-140 ˚C ($CHCl_3$).

^{1}H NMR (400 MHz, $CDCl_3$) δ ppm 7.93 – 7.83 (m, 2H, ArC*H* x 2), 7.78 – 7.67 (m, 2H, ArC*H* x 2), 7.54 (ddt, *J* = 8.8, 3.0, 1.4 Hz, 1H, ArC*H*), 7.48 (ddd, *J* = 8.5, 6.6, 3.3 Hz, 2H, ArC*H* x 2), 7.42 (ddt, *J* = 7.4, 5.6, 1.4 Hz, 1H, ArC*H*), 7.34 – 7.21 (m, 8H, ArC*H* x 8), 6.98 – 6.91 (m, 1H, ArC*H*), 6.90 – 6.80 (m, 2H,m ArC*H* x 2), 5.70 (d, *J* = 7.4 Hz, 1H, O*H*), 4.71 (td, *J* = 12.4, 3.2 Hz, 1H, NHC*H*), 4.14 – 4.02 (m, 1H, HOC*H*$_2$), 4.01 – 3.88 (m, 1H, HOC*H*$_2$), 3.82 – 3.73 (m, 4H, 1 OC*H*$_3$ and N*H*), 3.25 – 3.19 (m, 1H, PhCCC*H*).

^{13}C NMR (101 MHz, $CDCl_3$) δ ppm 159.6 (Ar*C*), 143.9 (d, *J* = 6.0 Hz, ArC), 133.0 (d, *J* = 10.2 Hz, Ar*C*H x 2), 132.6 (d, *J* = 2.8 Hz, Ar*C*H), 132.3 (d, *J* = 2.9 Hz, Ar*C*H), 132.1 (d, *J* = 134.4 Hz, Ar*C*), 131.8 (Ar*C*H x 2), 131.4 (d, *J* = 9.8 Hz, Ar*C*H x 2), 130.8 (d, *J* = 135.9 Hz, Ar*C*), 129.3 (Ar*C*H), 129.0 (d, *J* = 12.7 Hz, Ar*C*H x 2), 128.6 (d, *J* = 13.2 Hz, Ar*C*H x 2), 128.4 (Ar*C*H x 2), 128.4 (Ar*C*H),

122.9 (Ar*C*), 119.1 (Ar*C*H), 112.7 (Ar*C*H), 112.6 (Ar*C*H), 86.5 (Ph*C*≡C), 85.2 (PhC≡*C*), 62.0 (HO*C*H_2), 55.4 (O*C*H_3), 52.2 (HN*C*H), 44.4 (PhCC*C*H).

^{31}P NMR (162 MHz, $CDCl_3$) δ 27.66.

IR ν_{max} (neat/cm^{-1}): 3319, 2922, 2852, 1488, 1401, 1157, 1125, 1069, 1041, 1009, 909.

HRMS : calcd for $C_{30}H_{28}NO_3PNa$ $[M + Na]^+$: 504.1699, found 504.1691.

Specific rotation: $[\alpha]_D^{26.7}$ - 48.5 (c 0.47, $CHCl_3$).

Enantiomeric purity of **3c** was determined by HPLC analysis in comparison with authentic racemic material (ee >99%; **OD-H** column, 95:5 hexanes: *i*-PrOH, 1 mL/min, 20 °C, 254 nm)

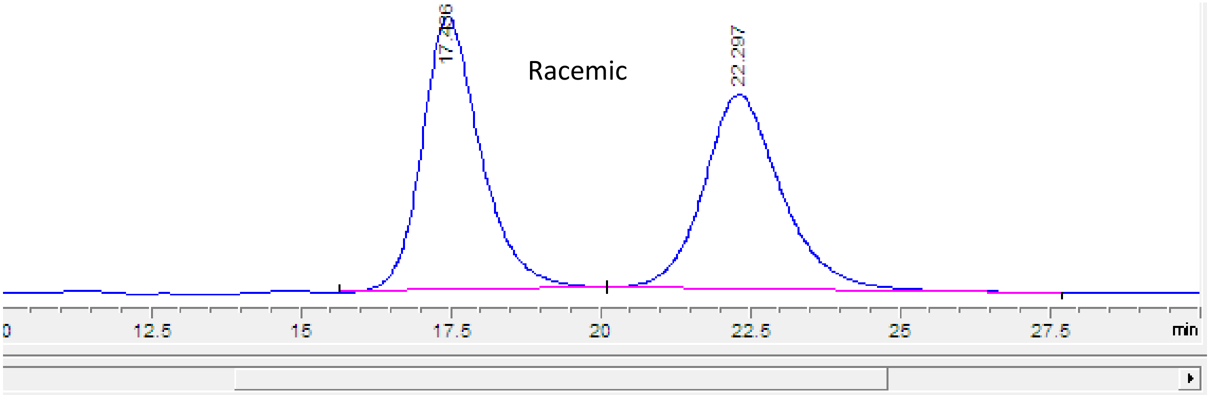

#	Time	Area	Height	Width	Area%	Symmetry
1	17.436	8467.1	123.6	1.0091	51.426	0.743
2	22.297	7997.5	88	1.2977	48.574	0.802

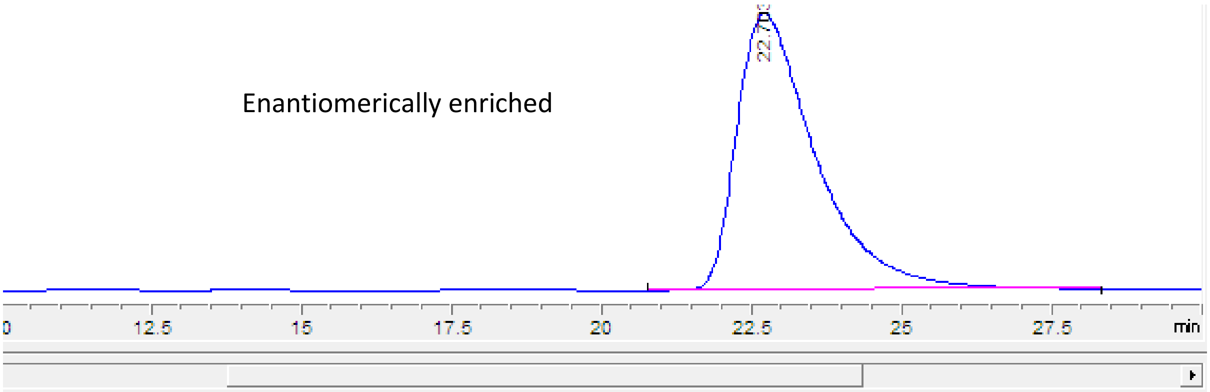

#	Time	Area	Height	Width	Area%	Symmetry
1	22.703	12452.3	135.1	1.351	100.000	0.497

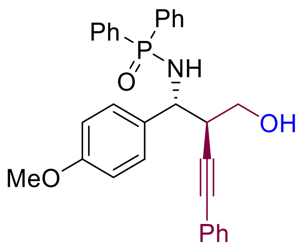

N-((1R,2S)-2-(Hydroxymethyl)-1-(4-methoxyphenyl)-4-phenylbut-3-yn-1-yl)-P,P-diphenylphosphinic amide (3d)

Compound **3d** was prepared according to general procedure A at 0 °C using MTBE as solvent. The title compound was isolated by column chromatography (hexane: ethyl acetate, 60:40-20:80) as a white amorphous solid (88.0 mg, 0.176 mmol, 83%). M.p.: 194-198 ˚C ($CHCl_3$).

^{1}H NMR (400 MHz, $CDCl_3$) δ ppm 7.81 (ddd, *J* = 12.2, 8.2, 1.4 Hz, 2H, ArC*H* x 2), 7.70 (ddd, *J* = 12.2, 8.2, 1.4 Hz, 1H, ArC*H* x 2), 7.48 (dtd, *J* = 10.3, 7.3, 1.4 Hz, 1H, ArC*H* x 2), 7.44 – 7.37 (m, 2H, ArC*H* x 2), 7.33 (td, *J* = 7.7, 3.3 Hz, 2H, ArC*H* x 2), 7.20 (s, 5H, ArC*H* x 5), 7.14 (d, *J* = 8.6 Hz, 2H, ArC*H* x 2), 6.86 (d, *J* = 8.6 Hz, 2H, ArC*H* x 2), 4.90 (t, *J* = 7.1 Hz, 1H, O*H*), 4.40 (td, *J* = 11.6, 8.8 Hz, 1H, HNC*H*), 4.24 – 4.11 (m, 1H, HOCH_2), 3.88 – 3.70 (m, 5H, OCH_3, N*H*, HOCH_2), 3.10 (dt, *J* = 8.5, 4.1 Hz, 1H, PhCCC*H*).

^{13}C NMR (101 MHz, $CDCl_3$) δ ppm 159.0 Ar*C*), 134.3 (d, *J* = 5.8 Hz, Ar*C*), 133.1 (d, *J* = 10.3 Hz, Ar*C*H x 2), 132.4 (d, *J* = 2.8 Hz, Ar*C*H), 132.4 (d, *J* = 129.1 Hz, Ar*C*), 132.2 (d, *J* = 2.8 Hz, Ar*C*H), 131.7 (Ar*C*H x 2), 131.6 (d, *J* = 9.7 Hz, Ar*C*H x 2), 130.9 (d, *J* = 131.7 Hz, Ar*C*), 128.8 (d, *J* = 12.8 Hz, Ar*C*H x 2), 128.5 (d, *J* = 13.0 Hz, Ar*C*H x 2), 128.3 (Ar*C*H x 2), 128.1 (Ar*C*H x 2), 127.9 (Ar*C*), 123.2 (Ar*C*H), 113.7 (Ar*C*H x 2), 88.3 (Ph*C*≡C), 84.8 (PhC≡*C*), 62.5 (HO*C*H_2), 55.4 (O*C*H_3), 55.2 (NH*C*H), 44.5 (d, *J* = 2.6 Hz, PhCC*C*H).

^{31}P NMR (162 MHz, $CDCl_3$) δ ppm 23.16.

IR ν_{max} (neat/cm−1): 3282, 3048, 2919, 1610, 1490, 1435, 1418, 1305, 1217, 1125, 1105, 1086, 925.

HRMS : calcd for $C_{30}H_{29}NO_3P$ [M + H]$^+$: 482.1880, found 482.1878.

Specific rotation: $[\alpha]_D^{28.3}$ - 12.9 (c 0.57, $CHCl_3$)

Enantiomeric purity of **3d** was determined by HPLC analysis in comparison with authentic racemic material (ee = 96%; **ODH** column, 93:7 hexanes: *i*-PrOH, 1 mL/min, 20 °C, 254 nm)

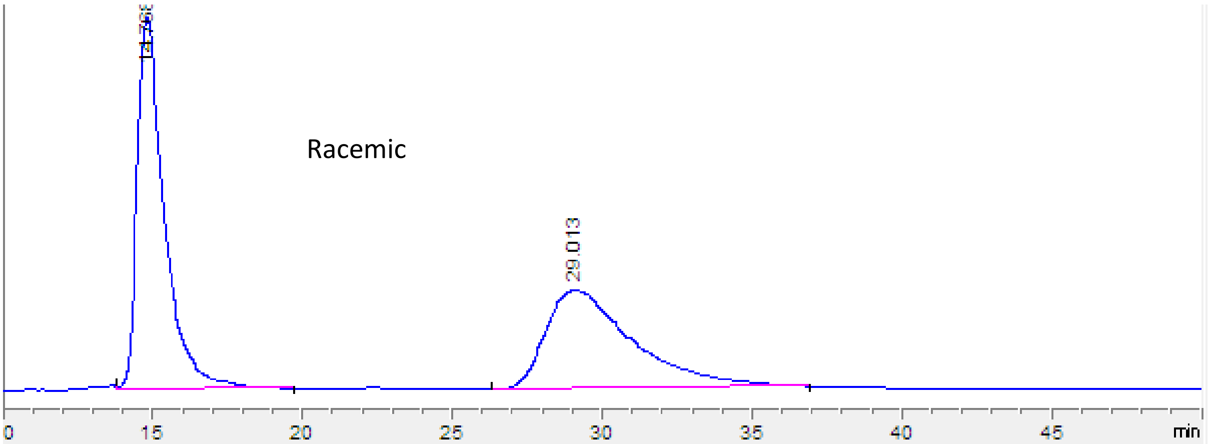

#	Time	Area	Height	Width	Area%	Symmetry
1	14.768	6797.3	107.4	0.9161	54.018	0.509
2	29.013	5786	28.6	2.4148	45.982	0.46

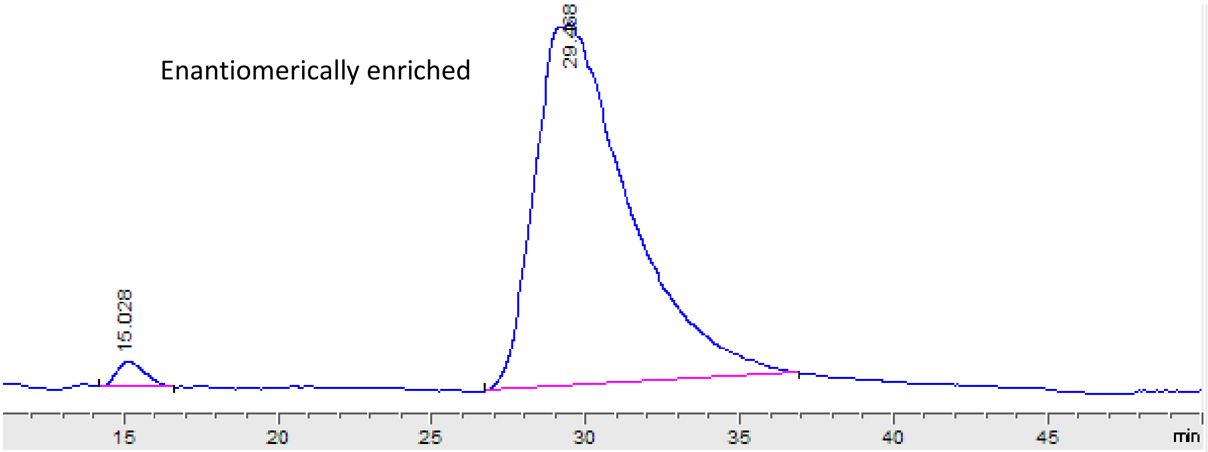

#	Time	Area	Height	Width	Area%	Symmetry
1	15.028	62.8	9.8E-1	0.7616	2.060	0.512
2	29.468	2988.3	14	2.5011	97.940	0.582

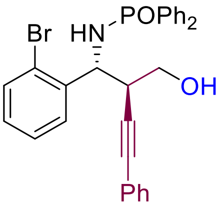

N-((1R,2S)-1-(2-Bromophenyl)-2-(hydroxymethyl)-4-phenylbut-3-yn-1-yl)-P,P-diphenylphosphinic amide (3e)

Compound **3e** was prepared according to general procedure A. The title compound was isolated by column chromatography (hexane: ethyl acetate, 60:40-20:80) as a white amorphous solid (65 mg, 0.164 mmol, 82%). M.p.: 183-188 ˚C ($CHCl_3$).

^{1}H NMR (500 MHz, DMSO-d_6) δ ppm 7.86 (d, J = 7.7 Hz, 1H, ArCH), 7.79 – 7.73 (m, 2H, ArCH x 2), 7.61 – 7.41 (m, 8H, ArCH x 8), 7.33 (td, J = 7.7, 3.1 Hz, 2H, ArCH x 2), 7.29 – 7.23 (m, 3H, ArCH x 3), 7.20 – 7.12 (m, 3H, ArCH x 3), 6.42 (t, J = 11.1 Hz, 1H, NH), 5.45 (dd, J = 7.6, 5.8 Hz, 1H, NHCH), 4.76 (q, J = 11.1 Hz, 1H, OH), 4.02 (dt, J = 11.0, 5.4 Hz, 1H, HOCH_2), 3.81 (ddd, J = 10.7, 7.6, 2.6 Hz, 1H, HOCH_2), 3.05 (d, J = 9.2 Hz, 1H, PhCCCH).

^{13}C NMR (126 MHz, DMSO-d_6) δ ppm 143.5 (ArC), 132.6 (d, J = 9.6 Hz, ArCH x 2), 132.9 (d, J = 126.4 Hz, ArC), 132.8 (d, J = 131.1 Hz, ArC), 132.4 (ArCH), 132.2 (d, J = 2.5 Hz, ArCH), 132.0 (ArCH), 131.9 (d, J = 9.5 Hz, ArCH x 2), 131.5 (ArCH x 2), 129.4 (ArCH), 129.1 (d, J = 13.2 Hz, ArCH x 2), 128.9 (d, J = 13.5 Hz, ArCH x 2), 128.8 (ArCH x 2), 128.7 (ArCH), 128.5 (ArCH), 128.3 (ArCH), 124.0 (ArC), 123.5 (ArC), 90.2 (PhC≡C), 82.7 (PhC≡C), 61.7 (HOCH$_2$), 53.5 (HNCH), 34.4 (PhCCCH).

^{31}P NMR (202 MHz, DMSO-d_6) δ ppm 23.12.

IR ν_{max} (neat/cm^{-1}): 3217, 3056, 2882, 1591, 1472, 1438, 1124, 1082, 1069, 980.

HRMS : calcd for $C_{29}H_{25}NO_2BrPNa$ $[M + Na]^+$: 552.0698, found 552.06845.

Specific rotation: $[\alpha]_D^{27}$ - 51.2 (c 0.48, $CHCl_3$).

Enantiomeric purity of **3e** was determined by HPLC analysis in comparison with authentic racemic material (ee : 98%; **OD-H** column, 90:10 hexanes : i-PrOH, 1 mL/min, 20 °C, 254 nm)

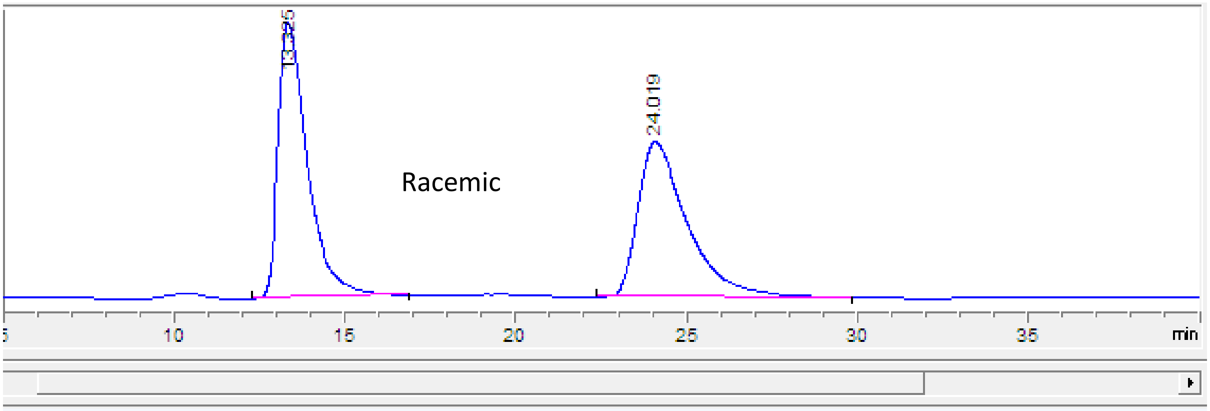

#	Time	Area	Height	Width	Area%	Symmetry
1	13.325	8337.9	140.8	0.897	51.269	0.491
2	24.019	7925.2	79.3	1.4358	48.731	0.462

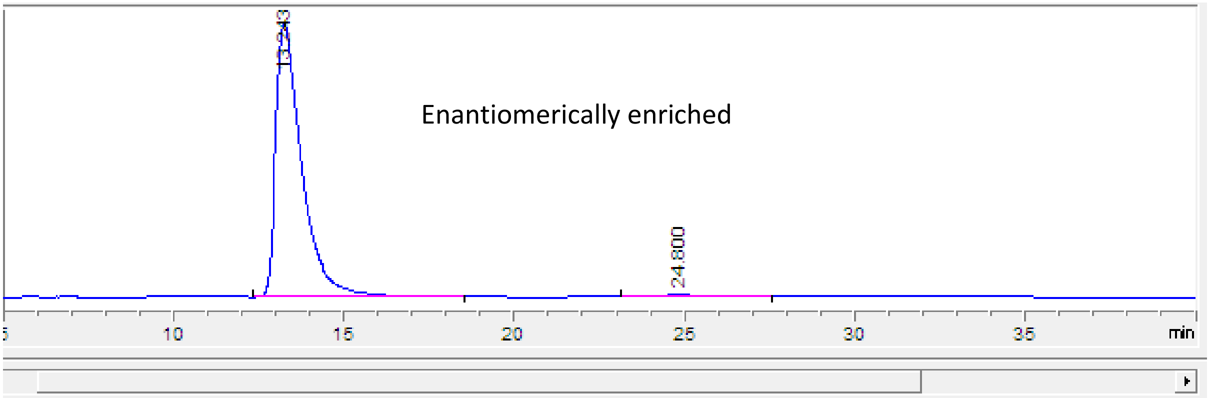

#	Time	Area	Height	Width	Area%	Symmetry
1	13.243	13821.1	262.2	0.765	98.750	0.469
2	24.8	175	1.7	1.2267	1.250	0.874

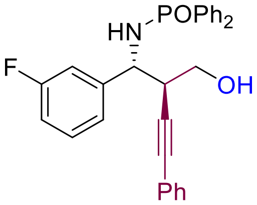

***N*-((1*R*,2*S*)-1-(3-Fluorophenyl)-2-(hydroxymethyl)-4-phenylbut-3-yn-1-yl)-*P*,*P*-diphenylphosphinic amide (3f)**

Compound **3f** was prepared according to general procedure A at 0 °C using MTBE as solvent. The title compound was isolated by column chromatography (hexane: ethyl acetate, 70:30-30:90) as a white amorphous solid (72.0 mg, 0.154 mmol, 77%). M.p.: 145-150 ˚C ($CHCl_3$).

^{1}H NMR (400 MHz, $CDCl_3$) δ ppm 7.95 – 7.75 (m, 2H, ArC*H* x 2), 7.74 – 7.60 (m, 2H, ArC*H* x 2), 7.57 – 7.38 (m, 4H, ArC*H* x 4), 7.35 – 7.25 (m, 3H, ArC*H* x 3), 7.21 (s, 5H, ArC*H* x 5), 7.05 – 6.75 (m, 2H, ArC*H* x 3), 4.47 (dd, *J* = 11.7, 8.4 Hz, 1H, N*H*), 4.21 – 4.09 (m, 1H, NHC*H*), 4.07 – 3.90 (m, 1H, HOC*H_2*), 3.72 (ddd, *J* = 12.7, 5.8, 3.3 Hz, 1H, HOC*H_2*), 3.15 (dt, *J* = 8.7, 3.8 Hz, 1H, PhCCC*H*). (OH peak not observed)

^{13}C NMR (101 MHz, $CDCl_3$) δ ppm 162.8 (d, *J* = 245.8 Hz, Ar*C*), 144.3 (t, *J* = 6.3 Hz, Ar*C*), 133.0 (d, *J* = 10.0 Hz, Ar*C*H x 2), 132.5 (d, *J* = 2.8 Hz, Ar*C*H), 132.4 (d, *J* = 2.9 Hz, Ar*C*H), 132.1 (d, *J* = 127.8 Hz, Ar*C*), 131.7 (Ar*C*H x 2), 131.6 (d, *J* = 10.5 Hz, Ar*C*H x 2), 130.6 (d, *J* = 133.5 Hz, Ar*C*), 128.9 (d, *J* = 12.4 Hz, Ar*C*H x 2), 129.8 (d, *J* = 8.1 Hz, Ar*C*H), 128.6 (d, *J* = 12.9 Hz, Ar*C*H x 2), 128.4 (Ar*C*H), 128.2 (Ar*C*H x 2), 128.1 (Ar*C*H), 123.2 (Ar*C*), 114.55 (d, *J* = 21.0 Hz, FAr*C*H),

114.30 (d, J = 21.9 Hz, FAr*C*H), 87.4 (Ph*C*≡C), 85.3 (PhC≡*C*), 62.4 (HO*C*H_2), 55.1 (NH*C*H), 44.16 (d, J = 2.4 Hz, PhCC*C*H).

^{31}P NMR (162 MHz, $CDCl_3$) δ ppm 26.13.

IR ν_{max} (neat/cm^{-1}): 3239, 3058, 2927, 1590, 1438, 1334, 1248, 1170, 1170, 1107.9, 1068, 1024.

HRMS : calcd for $C_{29}H_{25}NO_2FPNa$ $[M + Na]^+$: 492.1480, found 492.1481.

Specific rotation: $[\alpha]_D^{27.3}$ - 48.5 (c 0.48, $CHCl_3$).

Enantiomeric purity of **3f** was determined by HPLC analysis in comparison with authentic racemic material (ee >99%; **OD-H** column, 95:5 hexanes: *i*-PrOH, 1 mL/min, 20 °C, 254 nm)

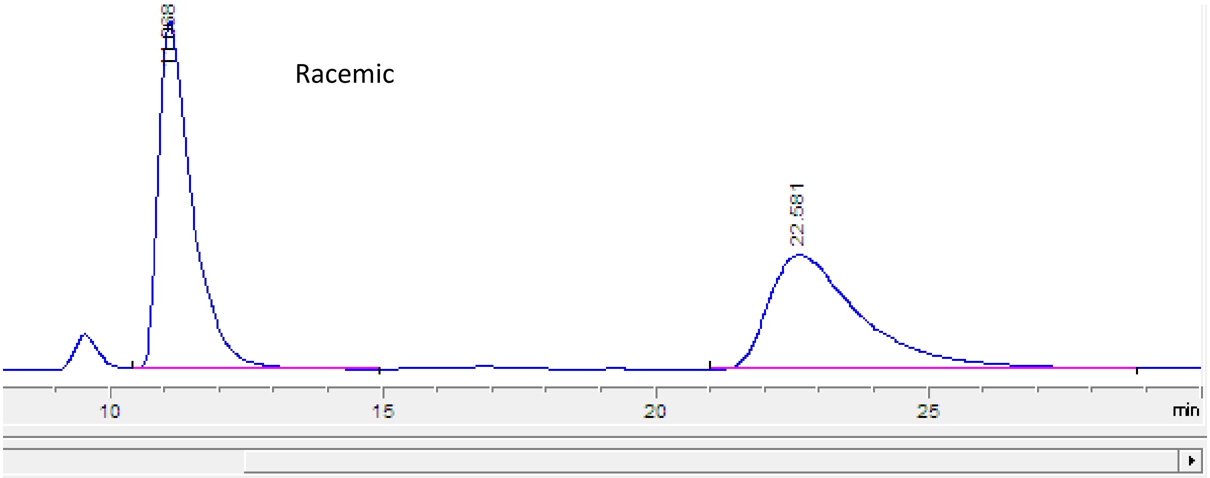

#	Time	Area	Height	Width	Area%	Symmetry
1	11.068	16435.2	384.2	0.6411	52.475	0.45
2	22.581	14884.6	126.6	1.548	47.525	0.427

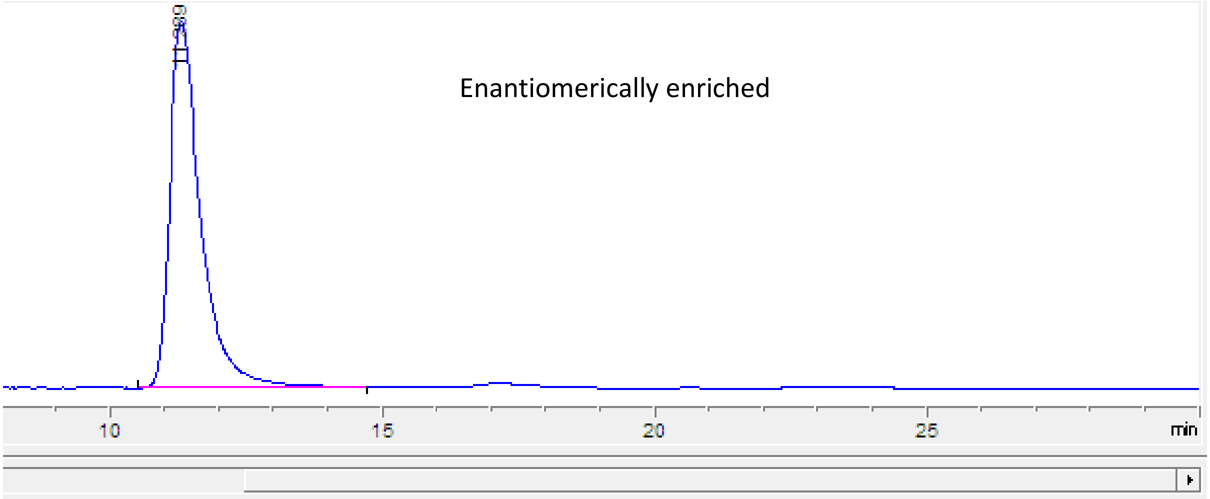

#	Time	Area	Height	Width	Area%	Symmetry
1	11.289	4465.3	112.8	0.5918	100.000	0.541

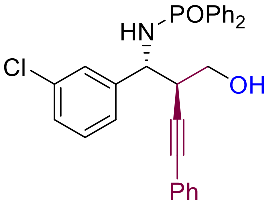

***N*-((1*R*,2*S*)-1-(3-Chlorophenyl)-2-(hydroxymethyl)-4-phenylbut-3-yn-1-yl)-*P*,*P*-diphenylphosphinic amide (3g)**

Compound **3g** was prepared according to general procedure A. The title compound was isolated by column chromatography (hexane: ethyl acetate, 60:40-30:70) as a white amorphous solid (66.0 mg, 0.132 mmol, 68%). M. p.: 189-194 ˚C ($CHCl_3$).

^{1}H NMR (400 MHz, $CDCl_3$) δ ppm 7.90 – 7.79 (m, 2H, ArC*H* x 2), 7.74 – 7.65 (m, 2H, ArC*H* x 2), 7.56 – 7.46 (m, 2H, ArC*H* x 2), 7.43 (td, *J* = 7.6, 3.2 Hz, 2H, ArC*H* x 2), 7.40 – 7.27 (m, 5H, ArC*H* x 5), 7.24 (s, 5H, ArC*H* x 5), 7.12 (dd, *J* = 7.4, 1.5 Hz, 1H, ArC*H*), 5.05 – 4.88 (m, 1H, O*H*), 4.56 – 4.44 (m, 1H, NHC*H*), 4.24 – 4.15 (m, 1H, HOC*H*$_2$), 4.13 – 4.03 (m, 1H, N-*H*), 3.80 – 3.70 (m, 1H, HOC*H*$_2$), 3.14 (dt, *J* = 8.7, 4.3 Hz, 1H, PhCCC*H*).

^{13}C NMR (101 MHz, $CDCl_3$) δ ppm 143.95 (d, *J* = 5.8 Hz, Ar*C*), 134.2 (Ar*C*), 133.0 (d, *J* = 9.9 Hz, Ar*C*H x 2), 132.5 (d, *J* = 2.4 Hz, Ar*C*H), 132.4 (d, *J* = 2.6 Hz, Ar*C*H), 132.1 (d, *J* = 127.9 Hz, Ar*C*), 131.7 (Ar*C*H), 131.6 (d, *J* = 9.9 Hz, Ar*C*H x 2), 130.6 (d, *J* = 133.2 Hz, Ar*C*) 129.5 (Ar*C*H), 128.9 (d, *J* = 12.7 Hz, Ar*C*H x 2), 128.6 (d, *J* = 12.9 Hz, Ar*C*H x 2), 128.3 (Ar*C*H x 2), 128.1 (Ar*C*H), 127.8

(ArCH), 127.5 (ArCH), 125.8 (ArCH), 123.1 (ArC), 87.5 (PhC≡C), 85.4 (PhC≡C), 62.3 ($HOCH_2$), 55.2 (NHCH), 44.1 (d, J = 2.6 Hz, PhCCCH).

^{31}P NMR (162 MHz, $CDCl_3$) δ ppm 25.76.

IR ν_{max} (neat/cm^{-1}): 3246, 2923, 2852, 1600, 1498, 1436, 1269, 1240, 1185, 1155, 1045, 997.

HRMS : calcd for $C_{29}H_{25}NO_2ClPNa$ $[M + Na]^+$: 508.1204, found 508.1196.

Specific rotation: $[\alpha]_D^{29.1}$ -48.5 (c 0.52, $CHCl_3$).

Enantiomeric purity of **3g** was determined by HPLC analysis in comparison with authentic racemic material (ee > 99%; **OD-H** column, 95:5 hexanes: *i*-PrOH, 1 mL/min, 20 °C, 254 nm)

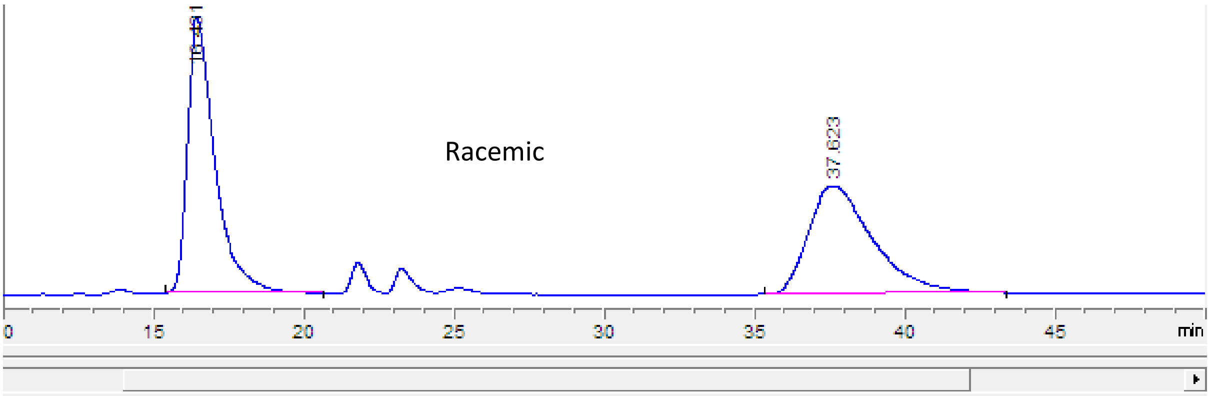

#	Time	Area	Height	Width	Area%	Symmetry
1	16.431	4797.5	74.9	0.9676	52.336	0.517
2	37.623	4369.2	29.3	1.7624	47.664	0.64

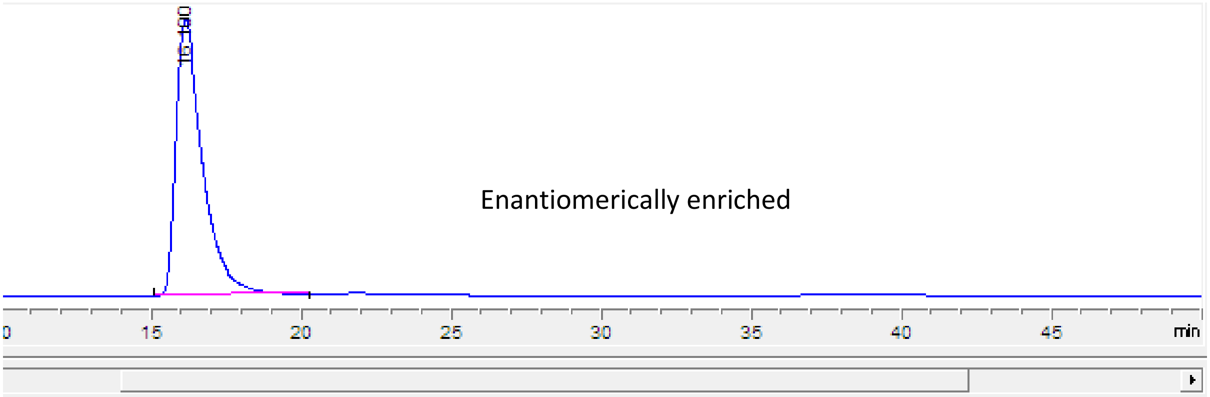

#	Time	Area	Height	Width	Area%	Symmetry
1	16.1	18782.4	304.3	0.9341	100.000	0.477

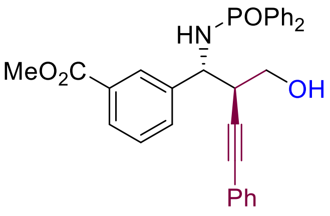

Methyl 3-((1*R*,2*S*)-1-((diphenylphosphoryl)amino)-2-(hydroxymethyl)-4-phenylbut-3-yn-1-yl)benzoate (3h)

Compound **3h** was prepared according to general procedure B. The title compound was isolated by column chromatography (hexane: ethyl acetate, 60:40-20:80) as a white amorphous solid (70.3 mg, 0.138 mmol, 69%). M.p.: 138-145 ˚C ($CHCl_3$).

^{1}H NMR (400 MHz, Acetone-d_6) δ ppm 8.29 (s, 1H, $MeCO_2ArCH$), 7.95 (dt, *J* = 7.8, 1.4 Hz, 1H, Ar*CH*), 7.91 – 7.86 (m, 2H, ArC*H* x 2), 7.81 – 7.70 (m, 3H, ArC*H* x 3), 7.61 – 7.42 (m, 5H, ArC*H* x 5), 7.37 – 7.19 (m, 7H, ArC*H* x 7), 5.89 (dd, *J* = 8.7, 6.2 Hz, 1H, N*H*), 5.59 (t, *J* = 11.8 Hz, 1H, O*H*), 4.81 (td, *J* = 12.4, 3.6 Hz, 1H, NHC*H*), 4.12 (td, *J* = 11.1, 6.0 Hz, 1H, $HOCH_2$), 3.88 (s, 3H, CO_2CH_3), 3.81 (ddd, *J* = 11.7, 8.6, 5.2 Hz, 1H, $HOCH_2$), 3.13 (ddd, *J* = 10.6, 5.1, 3.5 Hz, 1H, PhCCC*H*).

^{13}C NMR (101 MHz, Acetone-d_6) δ ppm 167.4 (*C*O), 144.4 (d, *J* = 5.4 Hz, Ar*C*), 133.6 (d, *J* = 127.2 Hz, ArC), 133.5 (d, *J* = 9.9 Hz, Ar*C*H x 2), 133.3 (d, *J* = 132.1 Hz, Ar*C*), 133.1 (d, *J* = 2.9 Hz, Ar*C*), 132.9 (d, *J* = 2.8 Hz, Ar*C*H), 132.6 (d, *J* = 7.6 Hz, Ar*C*H x 2), 132.5 (Ar*C*H x 3), 131.1 (Ar*C*H), 129.4 (d, *J* = 10.1 Hz, Ar*C*H x 2), 129.3 (d, *J* = 7.9 Hz, Ar*C*H x 2), 129.2 (Ar*C*H x 2), 129.2 (Ar*C*H), 129.1 (Ar*C*H), 129.0 (Ar*C*H), 129.0 (Ar*C*H), 124.2 (Ar*C*), 86.7 (Ph*C*≡C), 86.7 (PhC≡*C*), 62.5 ($HOCH_2$), 53.7 (CO_2CH_3), 52.4 (NH*C*H), 45.3 (d, *J* = 2.4 Hz, PhCC*C*H).

^{31}P NMR (162 MHz, Acetone-d_6) δ ppm 26.4.

IR ν_{max} (neat/cm^{-1}): 3307, 3057, 2949, 2929, 1716, 1590, 1436, 1287, 1176, 1131, 1068, 911.

HRMS : calcd for $C_{31}H_{28}NO_4PNa$ $[M + Na]^+$: 532.1626, found 532.1629.

Specific rotation: $[\alpha]_D^{22.5}$ - 71.1 (c 0.33, MeCOMe)

Enantiomeric purity of **3h** was determined by HPLC analysis in comparison with authentic racemic material (ee >98.4%; **OD-H** column, 95:5 hexanes: *i*-PrOH, 0.5 mL/min, 20 °C, 254 nm.

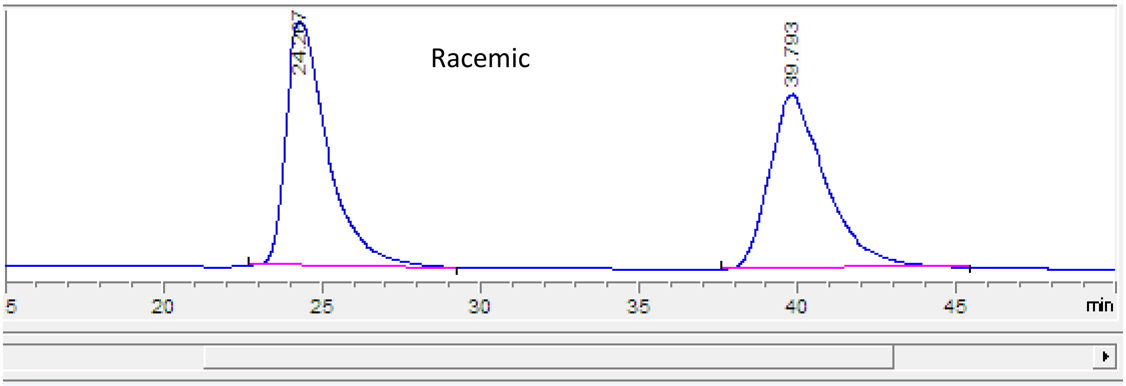

#	Time	Area	Height	Width	Area%	Symmetry
1	24.267	8097.4	87.6	1.3326	50.868	0.488
2	39.793	7821	62.7	1.5835	49.132	0.64

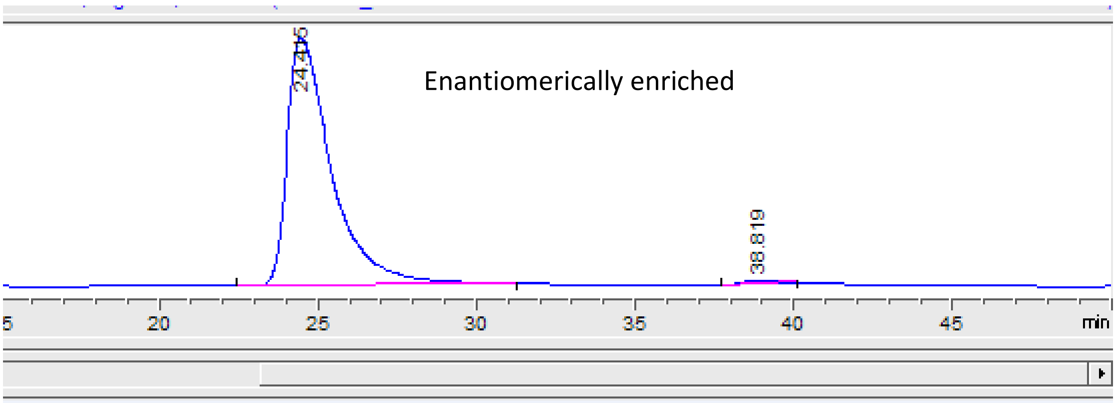

#	Time	Area	Height	Width	Area%	Symmetry
1	24.415	10041.6	103	1.4098	99.195	0.414
2	38.819	81.5	1.1	0.8996	0.805	1.119

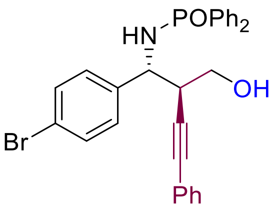

***N*-((1*R*,2*S*)-1-(4-Bromophenyl)-2-(hydroxymethyl)-4-phenylbut-3-yn-1-yl)-*P*,*P*-diphenylphosphinic amide (3i)**

Compound **3i** was prepared according to general procedure A. The title compound was isolated by column chromatography (hexane: ethyl acetate, 60:40-40:60) as a white amorphous solid (87.0 mg, 0.164 mmol, 82%). M. p.: 198-202 ˚C ($CHCl_3$).

^{1}H NMR (400 MHz, CDCl$_3$) δ ppm 7.81 (ddd, *J* = 12.0, 8.2, 1.4 Hz, 1H, ArC*H* x 2), 7.72 – 7.63 (m, 1H, ArC*H* x 2), 7.57 – 7.40 (m, 6H, ArC*H* x 6), 7.34 (td, *J* = 7.7, 3.4 Hz, 2H, ArC*H* x 2), 7.20 (qd, *J* = 5.7, 4.8, 3.0 Hz, 5H, ArC*H* x 5), 7.11 (d, *J* = 8.4 Hz, 2H, ArC*H* x 2), 4.83 (dd, *J* = 8.3, 5.9 Hz, 1H, O*H*), 4.43 (td, *J* = 11.7, 8.7 Hz, 1H, NHC*H*), 4.18 (ddd, *J* = 11.8, 5.9, 3.7 Hz, 1H, HOC*H*$_2$), 3.91 – 3.78 (m, 1H, N*H*), 3.77 – 3.66 (m, 1H, HOC*H*$_2$), 3.09 (dt, *J* = 8.6, 4.2 Hz, 1H, PhCCC*H*).
^{13}C NMR (101 MHz, CDCl$_3$) δ ppm 141.0 (d, *J* = 5.8 Hz, Ar*C*), 133.0 (d, *J* = 9.9 Hz, Ar*C*H x 2), 132.5 (d, *J* = 2.9 Hz, Ar*C*H), 132.4 (d, *J* = 2.8 Hz, Ar*C*H), 132.1 (d, *J* = 128.0 Hz, Ar*C*), 131.7(Ar*C*H x 2), 131.6 (d, *J* = 9.8 Hz, Ar*C*H x 2), 131.5 (Ar*C*H x 2), 130.6 (d, *J* = 133.3 Hz, Ar*C*), 129.0 (Ar*C*H x 2), 128.9 (d, *J* = 12.7 Hz, Ar*C*H x 2), 128.7 (d, *J* = 12.9 Hz, Ar*C*H x 2), 128.3 (Ar*C*H x 2), 128.1 (Ar*C*), 123.1 (Ar*C*H), 121.6 (Ar*C*H), 87.5 (Ph*C*≡C), 85.2 (PhC≡*C*), 62.4 (*C*H$_2$OH), 55.2 (NH*C*H), 44.2 (d, *J* = 2.6 Hz, PhCC*C*H).
^{31}P NMR (162 MHz, CDCl$_3$) δ ppm 25.75.
IR ν_{max} (neat/cm^{-1}): 3320, 2923, 2853, 1589, 1488, 1435, 1354, 1156, 1125, 1069.1, 1009, 876.
HRMS : calcd for $C_{29}H_{25}NO_2BrPNa$ [M + Na]$^+$: 552.0698, found 552.0690.
Specific rotation: $[\alpha]_D^{29.3}$ - 10.29 (c 0.7, CHCl$_3$).
Enantiomeric purity of **3i** was determined by HPLC analysis in comparison with authentic racemic material (ee >99%; **OD-H** column, 93:7 hexanes: *i*-PrOH, 0.7 mL/min, 20 °C, 254 nm)

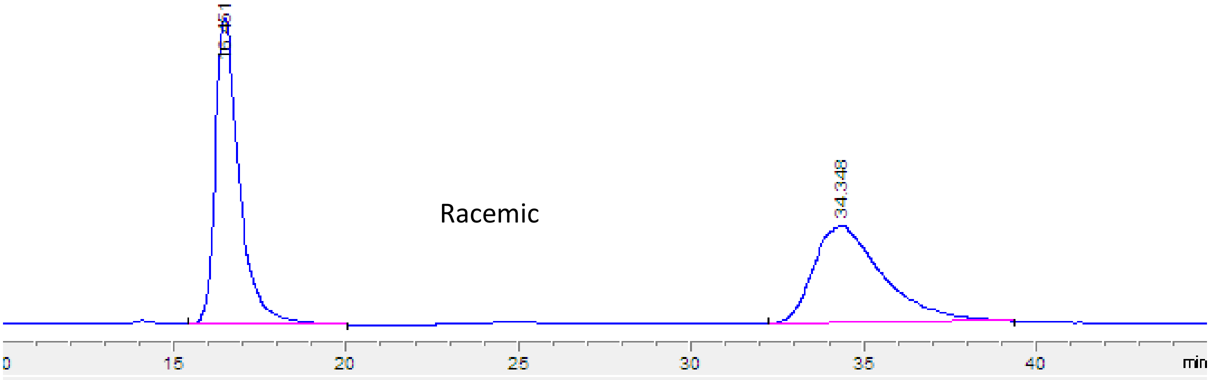

#	Time	Area	Height	Width	Area%	Symmetry
1	16.451	4571.8	94.5	0.7245	52.214	0.599
2	34.348	4184.1	30.1	1.7022	47.786	0.666

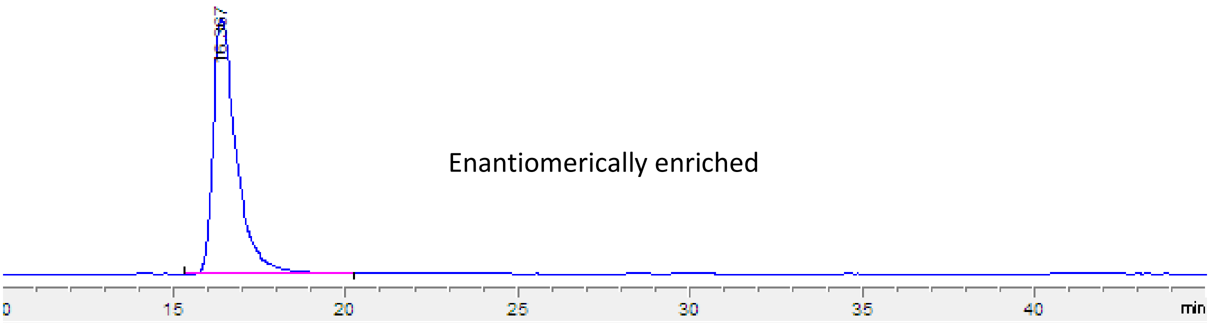

#	Time	Area	Height	Width	Area%	Symmetry
1	16.367	12833	272.6	0.7013	100.000	0.517

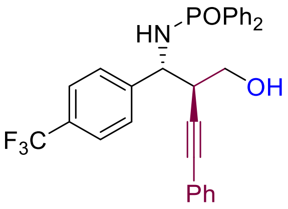

***N*-((1*R*,2*S*)-2-(Hydroxymethyl)-4-phenyl-1-(4-(trifluoromethyl)phenyl)but-3-yn-1-yl)-*P*,*P*-diphenylphosphinic amide (3j)**

Compound **3j** was prepared according to general procedure A. The title compound was isolated by column chromatography (hexane: ethyl acetate, 60:40-40:60) as a white amorphous solid (75.0 mg, 0.144 mmol, 72%). M.p.: >200 ˚C ($CHCl_3$).

^{1}H NMR (500 MHz, $CDCl_3$) δ ppm 7.83 (dd, *J* = 12.2, 7.6 Hz, 2H, ArC*H* x 2), 7.66 (dd, *J* = 12.2, 7.6 Hz, 2H, ArC*H* x 2), 7.59 (d, *J* = 7.9 Hz, 2H, ArC*H* x 2), 7.55 – 7.40 (m, 4H, ArC*H* x 4, 7.36 (d, *J* = 7.9 Hz, 2H, ArC*H* x 2), 7.32 (td, *J* = 7.7, 3.2 Hz, 2H, ArC*H* x 2), 7.2 – 7.1 (m, 5H, ArC*H* x 5), 4.88 (d, *J* = 7.0 Hz, 1H, O*H*), 4.6 – 4.4 (m, 1H, NHC*H*), 4.18 (dt, *J* = 11.9, 4.5 Hz, 1H, HOCH_2), 4.00 (dd, *J* = 11.3, 8.3 Hz, 1H, N*H*), 3.74 (ddd, *J* = 12.4, 7.8, 5.1 Hz, 1H, HOCH_2), 3.24 – 3.00 (m, 1H, PhCCC*H*).

^{13}C NMR (126 MHz, $CDCl_3$) δ ppm 145.8 (d, *J* = 5.5 Hz, Ar*C*), 133.0 (d, *J* = 9.9 Hz, Ar*C*H x 2), 132.6 (d, *J* = 2.7 Hz, Ar*C*H), 132.6 (Ar*C*H), 132.5 (d, *J* = 3.0 Hz, Hz, Ar*C*H), 131.6 (Ar*C*H x 2), 131.6 (d, *J* = 9.9 Hz, Ar*C*H x 2), 131.4 (Ar*C*H), 130.5 (d, *J* = 133.7 Hz, Ar*C*), 129.9 (q, *J* = 33.1 Hz, Ar*C*), 128.9 (d, *J* = 12.7 Hz, Hz, Ar*C*H x 2), 128.7 (d, *J* = 13.0 Hz, Ar*C*H x 2), 128.3 (Ar*C*H x 2), 128.2 (Ar*C*H), 127.8 (Ar*C*H x 2), 125.3 (q, *J* = 3.9 Hz, Hz, Ar*C*H), 124.2 (d, *J* = 261.2 Hz, *C*F_3), 123.0 (Ar*C*), 87.2 (Ph*C*≡C), 85.5 (PhC≡*C*), 62.34 (HO*C*H_2), 55.3 (NH*C*H), 44.1 (PhCC*C*H).

(1 P-Ar coupling overlap)

^{31}P NMR (162 MHz, CDCl$_3$) δ ppm 25.89.

IR ν_{max} (neat/cm^{-1}): 3320, 3199, 2929, 1438, 1415, 1115, 1307, 1152, 1123, 1100, 914.

HRMS : calcd for $C_{30}H_{26}NO_2F_3P$ [M + H]$^+$: 520.1648, found 520.1649.

Specific rotation: $[\alpha]_D^{29.1}$ - 14.1 (c 0.52, CHCl$_3$).

Enantiomeric purity of **3j** was determined by HPLC analysis in comparison with authentic racemic material (ee: 97%; **ODH** column, 90:10 hexanes: *i*-PrOH, 0.5 mL/min, 20 °C, 254 nm)

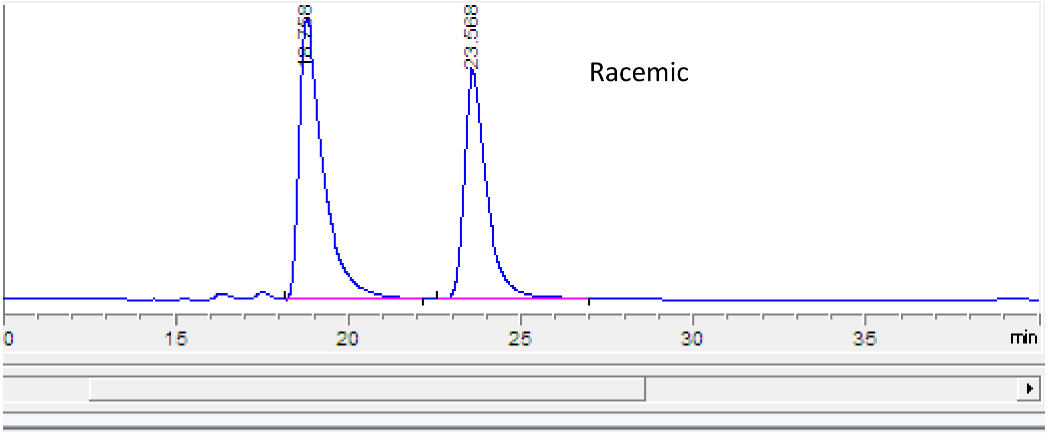

#	Time	Area	Height	Width	Area%	Symmetry
1	18.758	16171.5	337.3	0.7013	56.713	0.405
2	23.568	12343.2	275.4	0.6709	43.287	0.553

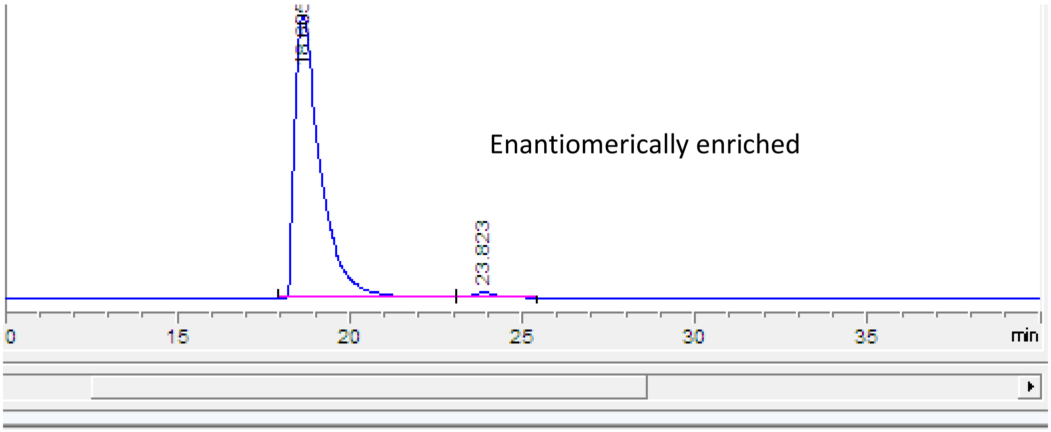

#	Time	Area	Height	Width	Area%	Symmetry
1	18.605	23967.8	471.5	0.7451	98.564	0.392
2	23.823	349.1	7.3	0.6822	1.436	0.661

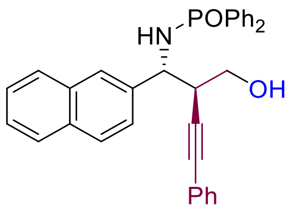

***N*-((1*R*,2*S*)-2-(Hydroxymethyl)-1-(naphthalen-2-yl)-4-phenylbut-3-yn-1-yl)-*P*,*P*-diphenylphosphinic amide (3k)**

Compound **3k** was prepared according to general procedure A. The title compound was isolated by column chromatography (hexane: ethyl acetate, 60:40-10:90) as a white amorphous solid (65.7 mg, 0.132 mmol, 66%). M.p.: 164-169 ˚C ($CHCl_3$).

^{1}H NMR (400 MHz, $CDCl_3$) δ ppm 7.95 – 7.78 (m, 5H, ArC*H* x 5), 7.77 (s, 1H, ArC*H*), 7.76 – 7.66 (m, 2H, ArC*H* x 2), 7.56 (td, *J* = 7.3, 1.5 Hz, 1H, ArC*H*), 7.50 (ddd, *J* = 8.8, 4.3, 2.2 Hz, 5H, ArC*H* x 5), 7.37 (td, *J* = 7.5, 1.7 Hz, 1H, ArC*H*), 7.28 – 7.15 (m, 7H, ArC*H* x 7), 5.74 (t, *J* = 7.5 Hz, 1H, OH), 4.92 (td, *J* = 12.4, 3.2 Hz, 1H, NHC*H*), 4.12 – 4.01 (m, 1H, HOCH_2), 3.96 – 3.88 (m, 1H, HOCH_2), 3.92 (dd, *J* = 11.8, 9.1 Hz, 1H, N*H*), 3.2 – 3.1 (m, 1H, PhCCC*H*).

^{13}C NMR (126 MHz, $CDCl_3$) δ ppm 139.4 (d, *J* = 5.9 Hz, Ar*C*), 133.3 (d, *J* = 10.3 Hz, Ar*C*H x 2), 133.1 (d, *J* = 15.8 Hz, Ar*C*H x 2), 132.4 (d, *J* = 2.6 Hz, Ar*C*H), 132.3 (d, *J* = 2.8 Hz, Ar*C*H), 132.2 (d, *J* = 127.4 Hz, Ar*C*), 132.8 (Ar*C*H x 2), 131.6 (Ar*C*), 131.6 (Ar*C*H), 130.7 (d, *J* = 133.1 Hz, Ar*C*), 128.8 (d, *J* = 12.7 Hz, Ar*C*H x 2), 128.6 (d, *J* = 13.1 Hz, Ar*C*H x 2), 128.2 (Ar*C*), 128.2 (Ar*C*H), 128.1 (Ar*C*H x 2), 127.9 (Ar*C*H), 127.8 (Ar*C*H), 126.4 (Ar*C*H), 126.3 (Ar*C*H), 126.1 (Ar*C*H), 125.2 (Ar*C*H), 123.3 (Ar*C*), 88.1 (Ph*C*≡C), 85.02 (PhC≡*C*), 62.5 (HO*C*H_2), 56.0 (NH*C*H), 44.32 (d, *J* = 2.5 Hz, PhCC*C*H).

IR ν_{max} (neat/cm^{-1}): 3300, 3056, 2922, 1510, 1489, 1435, 1378, 1347, 1156. 1125, 1067, 952.

HRMS : calcd for $C_{33}H_{29}NO_2P$ $[M + H]^+$ 502.1930, found 502.1930.

Specific rotation: $[\alpha]_D^{26.5}$ - 11.5 (c 0.55, $CHCl_3$).

Enantiomeric purity of **3k** was determined by HPLC analysis in comparison with authentic racemic material (ee >99%; **ODH** column, 85:15 hexanes: *i*-PrOH, 1 mL/min, 20 °C, 254 nm)

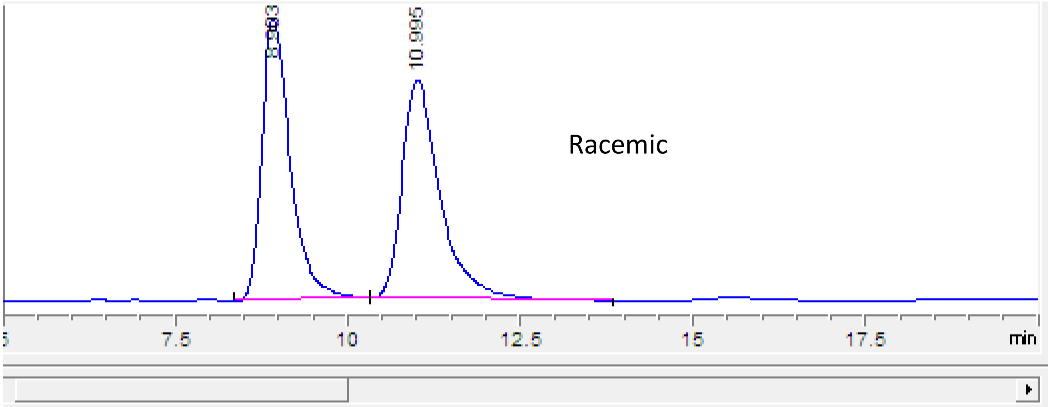

#	Time	Area	Height	Width	Area%	Symmetry
1	8.903	4296.9	149.2	0.438	49.429	0.617
2	10.995	4396.2	115.5	0.5721	50.571	0.622

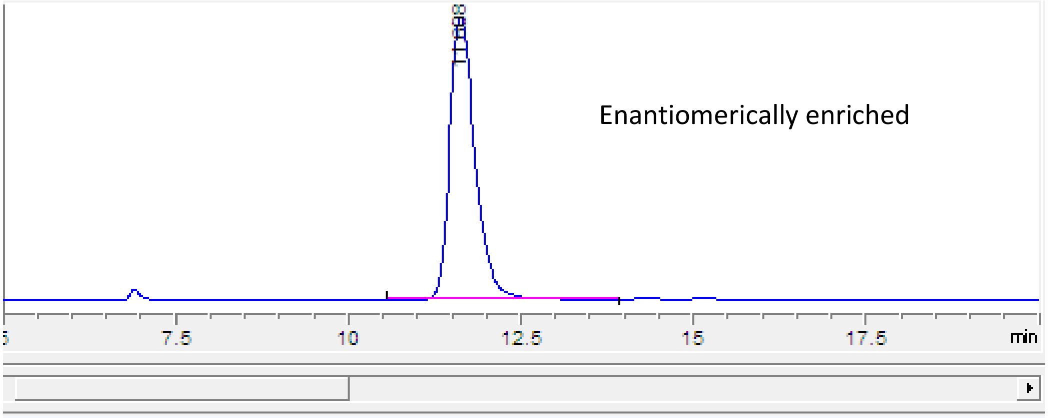

#	Time	Area	Height	Width	Area%	Symmetry
1	11.608	19483.5	754.5	0.3973	100.000	0.683

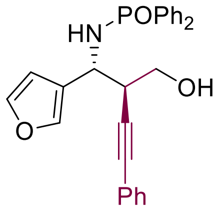

***N*-(1-(Furan-3-yl)-2-(hydroxymethyl)-4-phenylbut-3-yn-1-yl)-*P*,*P*-diphenylphosphinic amide (3l)**

Compound **3l** was prepared according to general procedure A at 0 °C using MTBE as solvent. The title compound was isolated by column chromatography (hexane: ethyl acetate, 60:40-20:80) as a white amorphous solid (80.5 mg, 0.182 mmol, 91%). M.p.: 171-175 ˚C ($CHCl_3$). **^{1}H NMR (400 MHz, $CDCl_3$)** δ ppm 7.79 – 7.63 (m, 4H, ArC*H* x 4), 7.50 – 7.39 (m, 2H, ArC*H* x 2), 7.39 – 7.28 (m, 5H, ArC*H* x 5), 7.22 – 7.05 (m, 5H, ArC*H* x 5), 6.33 – 6.16 (m, 1H, ArC*H*), 6.02

(d, *J* = 3.1 Hz, 1H, ArC*H*), 4.91 (s, 1H, O*H*), 4.47 (td, *J* = 11.4, 9.4 Hz, 1H, HNC*H*), 4.19 (d, *J* = 11.8 Hz, 1H, CH_2OH), 3.80 (d, *J* = 11.8 Hz, 1H, CH_2OH), 3.75 – 3.53 (m, 1H, N*H*), 3.19 – 3.05 (m, 1H, PhC≡C-C*H*).

^{13}C NMR (101 MHz, $CDCl_3$) δ ppm 154.0 (d, *J* = 6.4 Hz, Ar*C*), 141.9 (Ar*C*H), 133.1 (d, *J* = 10.2 Hz, Ar*C*H x 2), 132.5 (d, *J* = 2.7 Hz, Ar*C*H), 132.4 (d, *J* = 2.9 Hz, Ar*C*H), 131.8 (Ar*C*H x 2), 132.6 (d, *J* = 126.6 Hz, Ar*C*), 131.5 (d, *J* = 9.6 Hz, Ar*C*H x 2), 130.7 (d, *J* = 133.4 Hz, Ar*C*), 128.8 (d, *J* = 12.7 Hz, Ar*C*H x 2), 128.6 (d, *J* = 13.0 Hz, Ar*C*H x 2), 128.1 (Ar*C*H x 2), 127.9 (Ar*C*H), 123.4 (Ar*C*), 110.4 (ArCH), 107.3 (Ar*C*H), 87.8 (Ph*C*≡C), 84.3(PhC≡*C*), 61.9 (CH_2OH), 49.83 (N*C*H), 42.09 (PhC≡C*C*H).

^{31}P NMR (162 MHz, $CDCl_3$) δ ppm 25.93.

IR ν_{max} (neat/cm^{-1}): 3255, 2923, 2853, 1435, 1345, 1236, 1159, 1124, 1065, 916.

HRMS : calcd for $C_{27}H_{24}NO_3PNa$ $[M + Na]^+$ 464.1386, found 464.1379.

Specific rotation: $[\alpha]_D^{26.5}$ - 46.1 (c 0.35, $CHCl_3$).

Enantiomeric purity of **3l** was determined by HPLC analysis in comparison with authentic racemic material (ee >99%; **OD-H** column, 95:5 hexanes: *i*-PrOH, 1 mL/min, 20 °C, 254 nm)

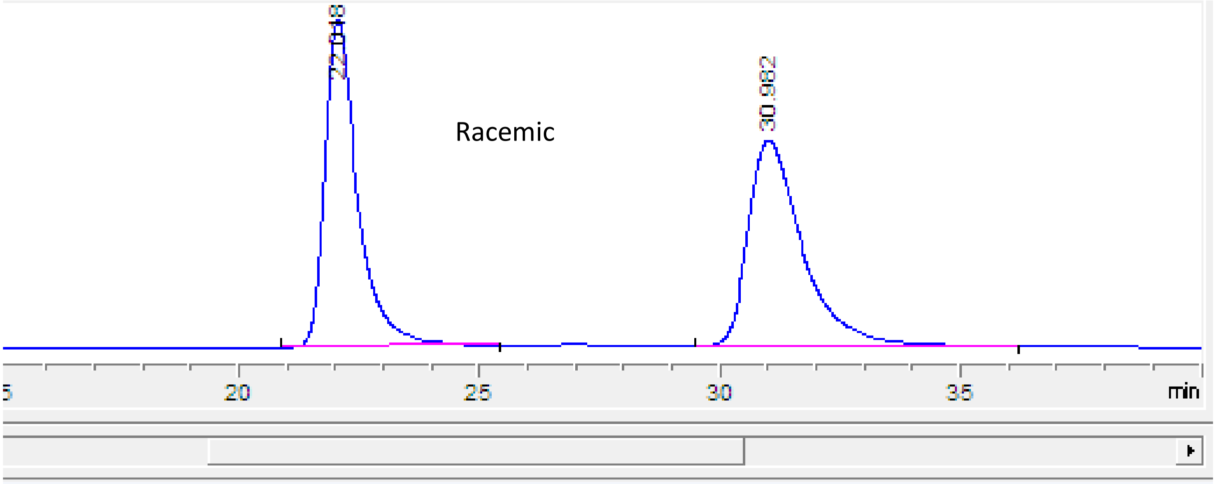

#	Time	Area	Height	Width	Area%	Symmetry
1	22.018	7848	163.8	0.7171	49.224	0.567
2	30.982	8095.6	103.1	1.1583	50.776	0.556

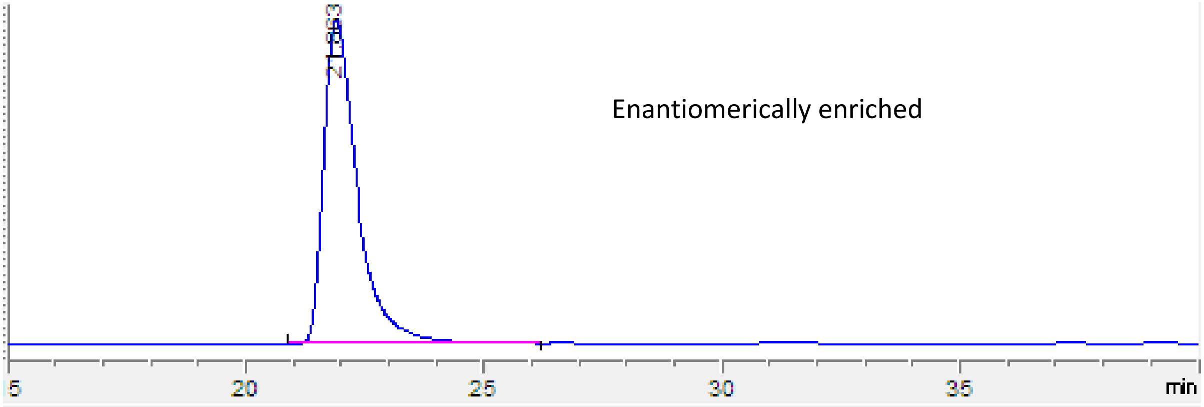

#	Time	Area	Height	Width	Area%	Symmetry
1	21.863	20801.5	425.3	0.7306	100.000	0.519

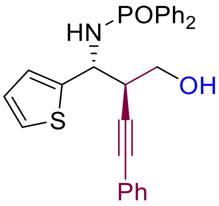

***N*-((1*R*,2*S*)-2-(Hydroxymethyl)-4-phenyl-1-(thiophen-2-yl)but-3-yn-1-yl)-*P*,*P*-diphenylphosphinic amide (3m)**

Compound **3m** was prepared according to general procedure A at 0 °C using MTBE as solvent. The title compound was isolated by column chromatography (hexane: ethyl acetate, 60:40-20:80) as a white amorphous solid (71.0 mg, 0.156 mmol, 78%). M.p.: 140-145 ˚C ($CHCl_3$).

^{1}H NMR (400 MHz, CD_3OD) δ ppm 9.39 (ddd, *J* = 12.3, 8.3, 1.4 Hz, 2H, ArC*H* x 2), 9.31 (ddd, *J* = 12.4, 8.3, 1.4 Hz, 2H, ArC*H* x 2), 9.16 – 9.00 (m, 4H, ArC*H* x 4), 8.95 (td, *J* = 7.6, 3.4 Hz, 2H, ArC*H* x 2), 8.91 – 8.86 (m, 3H, ArC*H* x 3), 8.81 (dd, *J* = 5.2, 1.9 Hz, 3H, ArC*H* x 3), 8.52 – 8.44 (m, 2H, ArC*H* x 2), 6.29 (dd, *J* = 11.1, 7.2 Hz, 1H, HNC*H*), 5.48 (dd, *J* = 11.2, 5.0 Hz, 1H, HOCH_2), 5.19 (dd, *J* = 11.2, 5.9 Hz, 1H, HOCH_2), 4.86 – 4.82 (m, 1H, PhCCC*H*).

^{13}C NMR (101 MHz, CD_3OD) δ ppm 146.0 (d, *J* = 5.4 Hz, Ar*C*), 133.6 (d, *J* = 10.2 Hz, ArCH x 2), 133.5 (d, *J* = 3.1 Hz, Ar*C*H), 133.4 (d, *J* = 2.9 Hz, Ar*C*H), 132.8 (d, *J* = 9.9 Hz, ArCH x 2), 132.6 (ArCH x 2), 132.0 (d, *J* = 130.5 Hz, Ar*C*), 131.7 (d, *J* = 130.9 Hz, Ar*C*), 129.8 (d, *J* = 12.8 Hz, Ar*C*H x 2), 129.6 (d, *J* = 13.1 Hz, Ar*C*H x 2), 129.3 (Ar*C*H x 2), 129.1 (Ar*C*H), 127.2 (Ar*C*H), 126.9 (Ar*C*H),

125.6 (Ar*C*H), 124.6 (Ar*C*), 88.7 (Ph*C*≡C), 86.2 (Ph*C*≡C), 63.1 (HO*C*H_2), 52.7 (NH*C*H)45.5 (d, *J* = 4.3 Hz, PhCC*C*H).

^{31}P NMR (162 MHz, DMSO-*d*$_6$) δ ppm 23.15.

IR ν_{max} (neat/cm^{-1}): 3255, 2952, 2926, 1436, 1346, 1266, 1156, 1065, 1041, 916.

HRMS : calcd for $C_{27}H_{25}NO_2SPNa$ [M + Na]$^+$: 458.1338, found 458.1338.

Specific rotation: $[\alpha]_D^{30.3}$ - 29.2 (c 0.51, $CHCl_3$).

Enantiomeric purity of **3m** was determined by HPLC analysis in comparison with authentic racemic material (ee : >99%; **OD-H** column, 90:10 hexanes: *i*-PrOH, 1 mL/min, 20 °C, 254 nm)

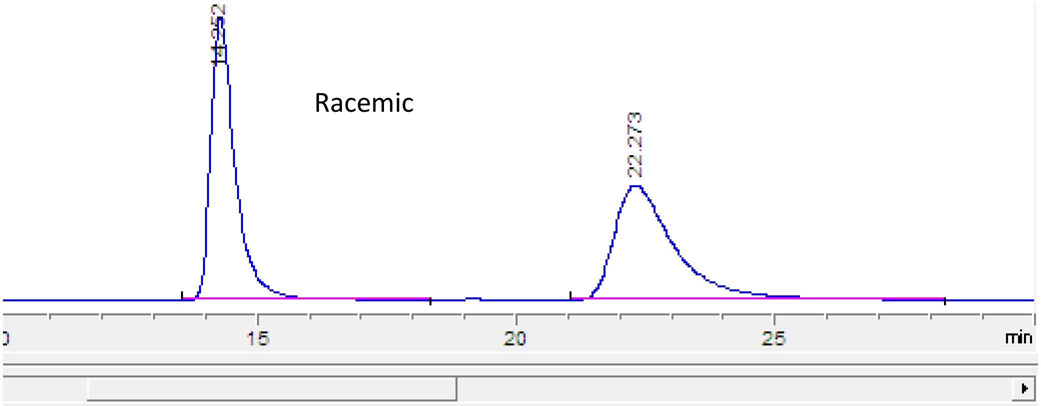

#	Time	Area	Height	Width	Area%	Symmetry
1	14.252	10873.3	327.1	0.4958	49.917	0.558
2	22.273	10909.4	133.1	1.2001	50.083	0.483

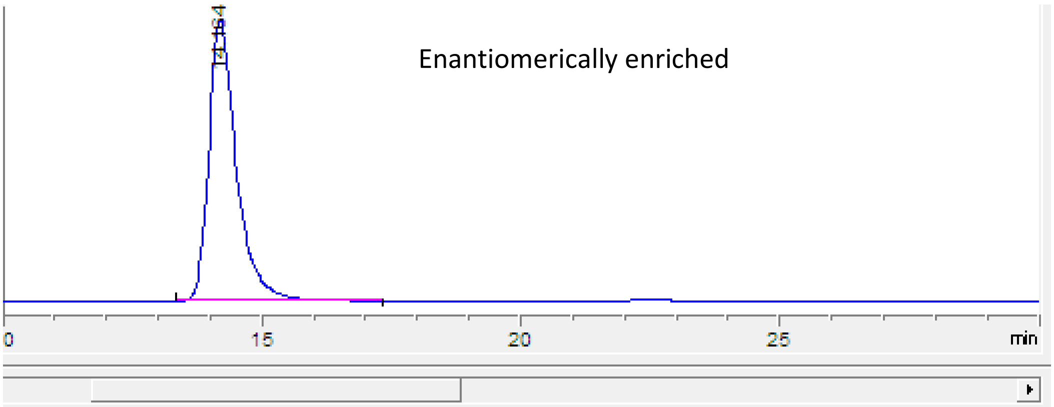

#	Time	Area	Height	Width	Area%	Symmetry
1	14.164	33287.3	893.7	0.5545	100.000	0.635

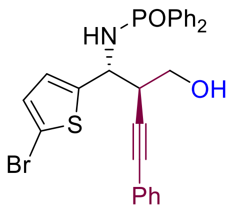

***N*-((1*R*,2*S*)-1-(5-Bromothiophen-2-yl)-2-(hydroxymethyl)-4-phenylbut-3-yn-1-yl)-*P*,*P*-diphenylphosphinic amide (3n)**

Compound **3n** was prepared according to general procedure A at 0 °C using MTBE as solvent. The title compound was isolated by column chromatography (hexane: ethyl acetate, 60:40-40:60) as a white amorphous solid (88.0 mg, 0.176 mmol, 82%). M.p.: 187-192 ˚C ($CHCl_3$).

^{1}H NMR (400 MHz, $CDCl_3$) δ ppm 7.91 – 7.76 (m, 4H, ArC*H* x 4), 7.54 – 7.48 (m, 2H, ArC*H* x 2), 7.46 – 7.36 (m, 4H, ArC*H* x 4), 7.33 (dd, *J* = 7.3, 2.4 Hz, 2H, ArC*H* x 2), 7.28 – 7.21 (m, 3H, ArC*H* x 3), 6.87 (d, *J* = 3.8 Hz, 1H, ArC*H*), 6.61 (d, *J* = 3.7 Hz, 1H, ArC*H*), 4.73 (td, *J* = 11.3, 7.8 Hz, 1H, NHC*H*), 4.62 (t, *J* = 6.9 Hz, 1H, O*H*), 4.11 (dt, *J* = 11.7, 4.7 Hz, 1H, HOCH_2), 3.87 (dd, *J* = 11.3, 8.2 Hz, 1H, N*H*), 3.77 – 3.65 (m, 1H, HOCH_2), 3.21 (ddd, *J* = 7.8, 5.6, 3.9 Hz, 1H, PhCCC*H*).

^{13}C NMR (101 MHz, $CDCl_3$) δ ppm 147.1 (d, *J* = 6.9 Hz, Ar*C*), 132.9 (d, *J* = 10.2 Hz, Ar*C*H x 2), 132.5 (d, *J* = 2.8 Hz, Ar*C*H), 132.4 (d, *J* = 2.8 Hz, Ar*C*H), 132.0 (d, *J* = 127.8 Hz, Ar*C*), 131.8 (Ar*C*H x 2), 131.7 (d, *J* = 9.8 Hz, Ar*C*H x 2), 130.5 (d, *J* = 132.6 Hz, Ar*C*), 129.3 (Ar*C*H), 128.9 (d, *J* = 12.7 Hz, Ar*C*H x 2), 128.7 (d, *J* = 13.0 Hz, Ar*C*H x 2), 128.3 (Ar*C*H x 2), 128.2 (Ar*C*H), 126.1 (Ar*C*H), 123.0 (Ar*C*), 111.4 (Ar*C*), 87,2 (Ph*C*≡C), 85,5 (Ph*C*≡C), 62.4 (HO*C*H_2), 52.0 (NH*C*H), 44.6 (d, *J* = 2.4 Hz, PhC≡C*C*H).

^{31}P NMR (162 MHz, $CDCl_3$) δ ppm 25.10.

IR ν_{max} (neat/cm^{-1}): 3317, 2922, 2853, 1436, 1373, 1329, 1155, 1126, 1068, 997.

HRMS : calcd for $C_{27}H_{24}NO_2BrPSNa$ [M + Na]$^+$: 536.0443, found 536.0443.

Specific rotation: $[\alpha]_D^{28.8}$ - 5.6 (c 0.48, $CHCl_3$).

Enantiomeric purity of **3n** was determined by HPLC analysis in comparison with authentic racemic material (ee >99%; **ODH** column, 90:10 hexanes: *i*-PrOH, 1 mL/min, 20 °C, 254 nm)

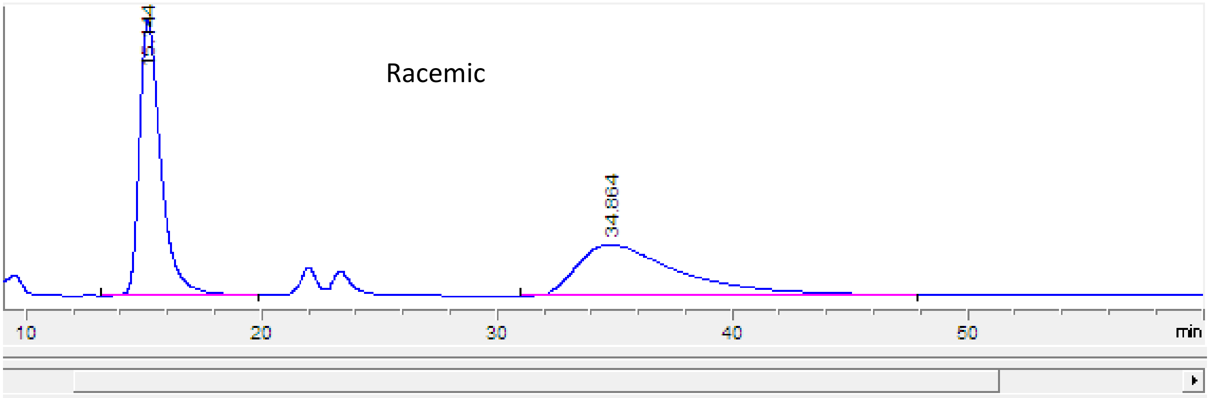

#	Time	Area	Height	Width	Area%	Symmetry
1	15.144	10815	172.5	0.9476	53.265	0.596
2	34.864	9489	31.9	3.5257	46.735	0.499

`

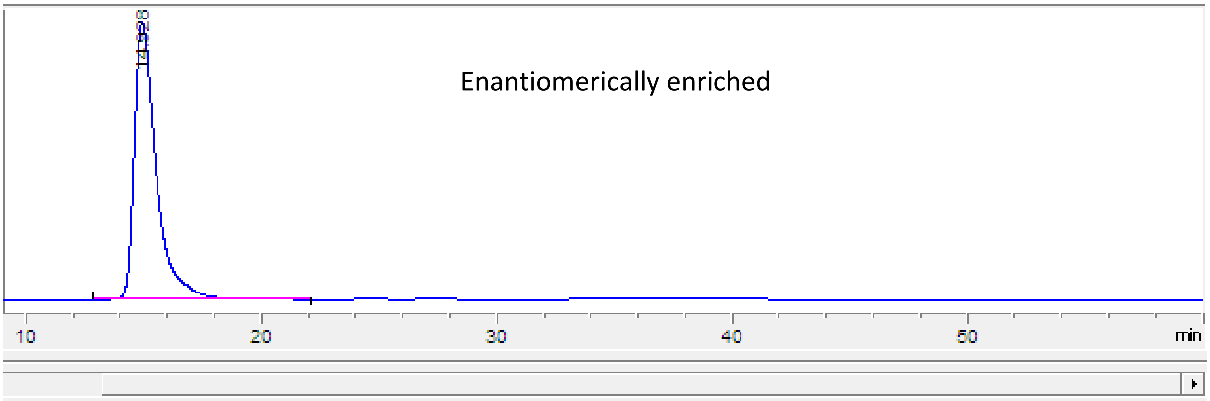

#	Time	Area	Height	Width	Area%	Symmetry
1	14.928	29196.9	454.6	0.9612	100.000	0.536

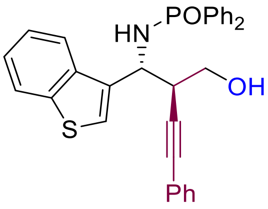

_N_-((1_S_,2_R_)-1-(Benzo[b]thiophen-3-yl)-2-(hydroxymethyl)-4-phenylbut-3-yn-1-yl)-_P_,_P_-diphenylphosphinic amide (3o)

Compound **3o** was prepared according to general procedure A using MTBE as solvent at 0 °C. The title compound was isolated by column chromatography (hexane: ethyl acetate, 60:40-30:70) as a white amorphous solid (52.5 mg, 0.104 mmol, 52%). M.p.: 129-135 ˚C ($CHCl_3$).

^{1}H NMR (400 MHz, $CDCl_3$) δ ppm 7.87 (d, J = 8.1 Hz, 1H, ArCH), 7.85 – 7.75 (m, 2H, ArCH x 2), 7.72 – 7.62 (m, 2H, ArCH x 2), 7.55 (d, J = 8.2 Hz, 1H, ArCH), 7.52 – 7.43 (m, 1H, ArCH), 7.44 –

7.33 (m, 4H, ArC*H* x 4), 7.32 (t, *J* = 7.6 Hz, 1H, ArC*H*), 7.25 – 7.11 (m, 6H, ArC*H* x 6), 7.08 – 7.01 (m, 2H, ArC*H* x 2), 4.98 – 4.87 (m, 1H, NHC*H*), 4.82 (t, *J* = 7.2 Hz, 1H, N*H*), 4.26 – 4.15 (m, 1H, HOC*H*$_2$), 4.14 – 4.04 (m, 1H, O*H*), 3.88 – 3.74 (m, 1H, HOC*H*$_2$), 3.42 (dt, *J* = 8.5, 3.9 Hz, 1H, PHCCC*H*).

^{13}C NMR (101 MHz, CDCl$_3$) δ ppm 140.5 (Ar*C*), 137.6 (Ar*C*), 137.4 (d, *J* = 5.5 Hz, Ar*C*), 132.9 (d, *J* = 10.1 Hz, Ar*C*H x 2), 132.4 (d, *J* = 2.7 Hz, Ar*C*H), 132.2 (d, *J* = 3.0 Hz, Ar*C*H), 132.1 (d, *J* = 127.4 Hz, Ar*C*), 131.7 (Ar*C*H x 3), 131.6 (Ar*C*H), 130.7 (d, *J* = 132.2 Hz, Ar*C*), 128.8 (d, *J* = 12.7 Hz, Ar*C*H x 2), 128.5 (d, *J* = 13.1 Hz, Ar*C*H x 2), 128.1 (Ar*C*H x 2), 127.9 (Ar*C*H), 124.4 (Ar*C*H), 124.1 (Ar*C*H), 123.9 (ArCH), 123.2 (ArCH), 123.0 (ArCH), 122.1 (ArC), 88.1 (Ph*C*≡C), 84.9 (PhC≡*C*), 62.58 (HO*C*H$_2$), 50.7 (HN*C*H), 43.6 (d, *J* = 2.8 Hz, PhC≡C*C*H).

^{31}P NMR (162 MHz, CDCl$_3$) δ ppm 25.81.

IR ν_{max} (neat/cm^{-1}): 3298, 3202, 3055, 2926, 1592, 1488, 1434, 1368, 1165, 1035, 926.

HRMS : calcd for $C_{31}H_{27}NO_2PSNa$ [M + Na]$^+$: 508.1495, found 508.1493.

Specific rotation: $[\alpha]_D^{30.7}$ - 106.29 (c 0.53, CHCl$_3$).

Enantiomeric purity of **3o** was determined by HPLC analysis in comparison with authentic racemic material (ee : >99%; **IA** column, 90:10 hexanes: *i*-PrOH, 0.5 mL/min, 20 °C, 254 nm)

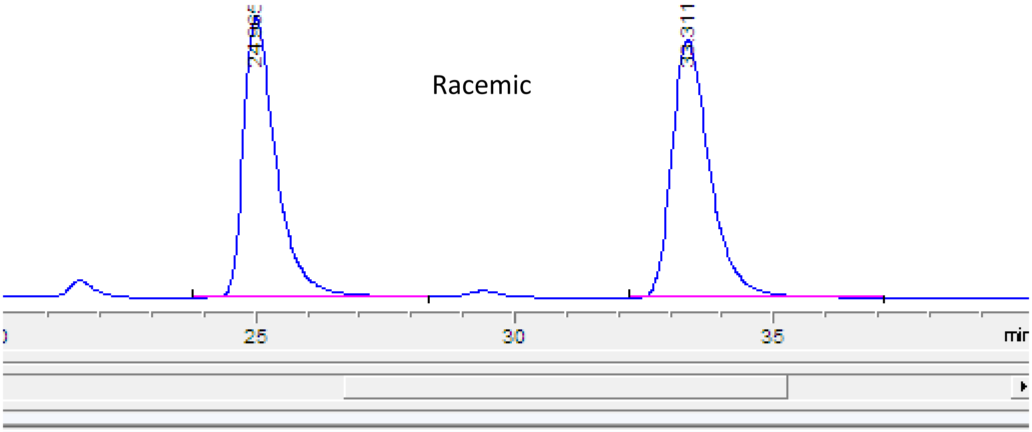

#	Time	Area	Height	Width	Area%	Symmetry
1	24.965	7004.3	159.5	0.6583	48.377	0.542
2	33.311	7474.3	145.8	0.7802	51.623	0.661

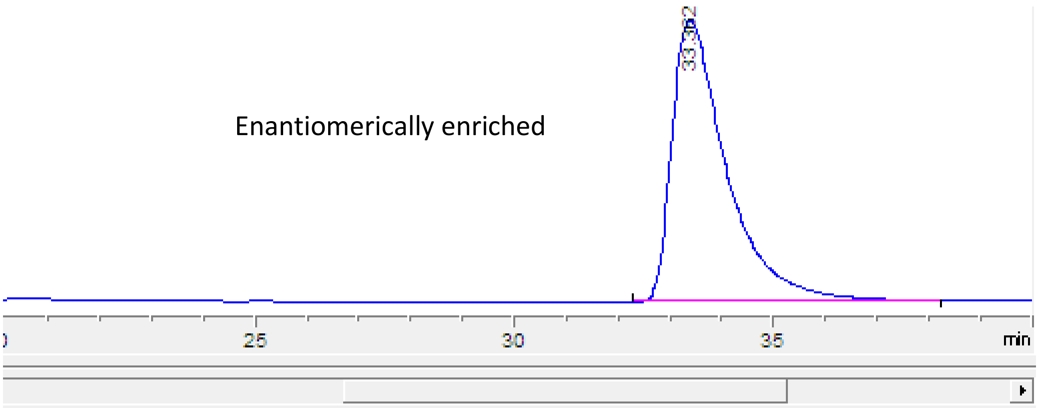

#	Time	Area	Height	Width	Area%	Symmetry
1	33.362	5478.9	74.6	1.0747	100.000	0.447

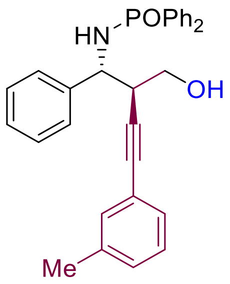

***N*-((1*R*,2*S*)-2-(Hydroxymethyl)-1-phenyl-4-(*m*-tolyl)but-3-yn-1-yl)-*P*,*P*-diphenylphosphinic amide (4a)**

Compound **4a** was prepared according to general procedure B. The title compound was isolated by column chromatography (hexane: ethyl acetate, 60:40-40:60) as a white amorphous solid (60.5 mg, 0.130 mmol, 65%). M.p.: 168-172 ˚C ($CHCl_3$).

^{1}H NMR (400 MHz, $CDCl_3$) δ ppm 7.85 – 7.74 (m, 2H, ArC*H* x 2), 7.73 – 7.64 (m, 2H, ArC*H* x 2), 7.55 – 7.45 (m, 2H, ArC*H* x 2), 7.46 – 7.39 (m, 2H, ArC*H* x 2), 7.37 – 7.30 (m, 5H, ArC*H* x 5), 7.24 – 7.15 (m, 2H, ArC*H* x 2), 7.10 – 7.04 (m, 1H, ArC*H*), 7.04 – 7.00 (m, 2H, ArC*H* x 2), 6.97 (dd, *J* = 7.5, 1.6 Hz, 1H, ArC*H*), 4.81 (br, 1H, O*H*), 4.51 – 4.37 (m, 1H, N*H*), 4.21 (d, *J* = 12.1 Hz, 1H, HOCH_2), 3.86 – 3.76 (m, 1H, HOCH_2), 3.71 (dd, *J* = 11.5, 9.4 Hz, 1H, NHC*H*), 3.14 – 2.97 (m, 1H, ArCCC*H*), 2.24 (s, 3H, CH_3).

^{13}C NMR (126 MHz, $CDCl_3$) δ ppm 142.0 (d, *J* = 5.8 Hz, Ar*C*), 137.8 (Ar*C*), 133.2 (d, *J* = 10.1 Hz, Ar*C*H x 2), 132.4 (d, *J* = 127.2 Hz, Ar*C*), 132.4 (d, *J* = 2.7 Hz, Ar*C*H), 132.3 (Ar*C*H), 132.3 (d, *J* = 3.3 Hz, Ar*C*H), 131.6 (d, *J* = 9.8 Hz, Ar*C*H x 2), 130.7 (d, *J* = 133.2 Hz, Ar*C*), 128.9 (d, *J* = 10.3 Hz, Ar*C*H x 2), 128.8 (Ar*C*H), 128.7 (Ar*C*H), 128.5 (d, *J* = 12.9 Hz, Ar*C*H x 2), 128.4 (Ar*C*H x 2), 128.0 (Ar*C*H), 127.7 (Ar*C*H), 127.2 (Ar*C*H x 2),123.1 (Ar*C*), 87.7 (Ar*C*≡C), 85.1 (ArC≡*C*), 62.5 (HO*C*H_2), 55.9 (NH*C*H), 44.4 (ArCC*C*H), 21.3 (*C*H_3).

^{31}P NMR (162 MHz, $CDCl_3$) ppm δ 25.67.

IR ν_{max} (neat/cm^{-1}): 3308, 3198, 2928, 1448, 1432, 1250, 1176, 1125, 902.

HRMS : calcd for $C_{30}H_{28}NO_2PNa$ $[M + Na]^+$ 488.1750, found 488.1741.

Specific rotation: $[\alpha]_D^{26.5}$ + 13.2 (c 1.0, CH_2Cl_2).

Enantiomeric purity of **4a** was determined by HPLC analysis in comparison with authentic racemic material (ee >99%; **OD-H** column, 97:3 hexanes: *i*-PrOH, 1 mL/min, 20 °C, 254 nm)

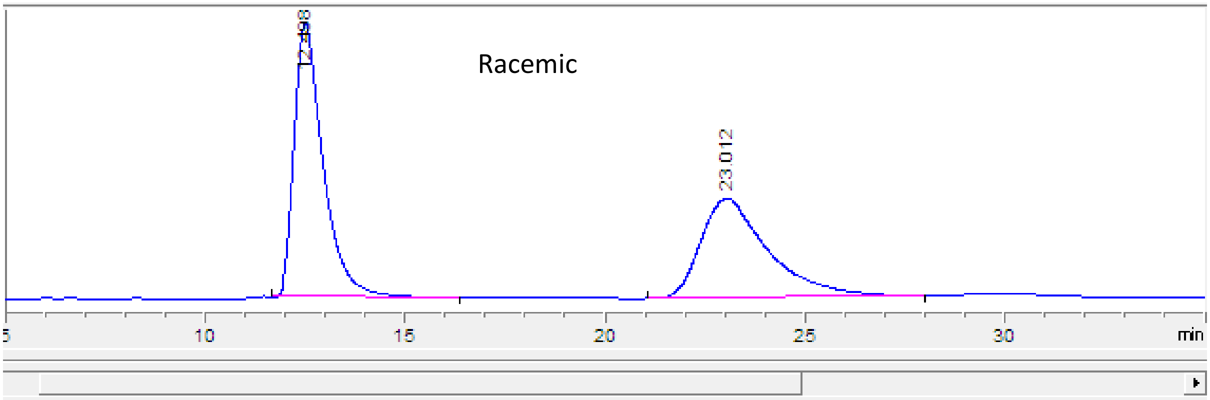

#	Time	Area	Height	Width	Area%	Symmetry
1	12.498	12984	260.1	0.7365	53.796	0.586
2	23.012	11151.8	94.2	1.6685	46.204	0.579

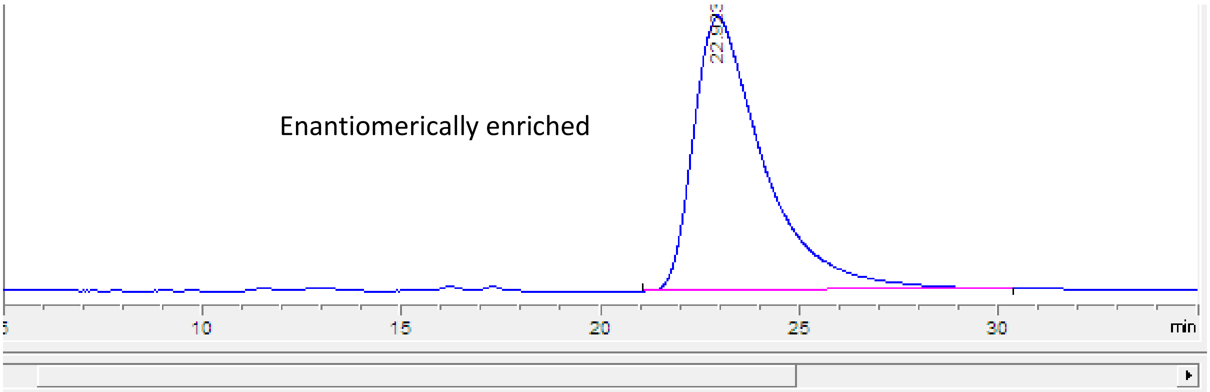

#	Time	Area	Height	Width	Area%	Symmetry
1	22.923	11221.6	91.1	1.7648	100.000	0.496

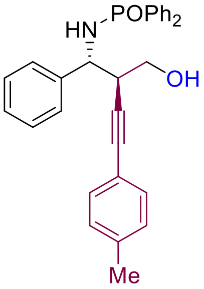

N-((1R,2S)-2-(Hydroxymethyl)-1-phenyl-4-(p-tolyl)but-3-yn-1-yl)-P,P-diphenylphosphinic amide (4b)

Compound **4b** was prepared according to general procedure B. The title compound was isolated by column chromatography (hexane: ethyl acetate, 60:40-20:80) as a white amorphous solid (68 mg, 0.146 mmol, 73%). M.p.: 180-185 ˚C ($CHCl_3$).

^{1}H NMR (500 MHz, $CDCl_3$) δ ppm 7.86 – 7.74 (m, 2H, ArC*H* x 2), 7.73 – 7.66 (m, 2H, ArC*H* x 2), 7.53 – 7.43 (m, 2H, ArC*H* x 2), 7.45 – 7.38 (m, 2H, ArC*H* x 2), 7.32 (dt, *J* = 11.2, 3.0 Hz, 5H, ArC*H* x 5), 7.24 – 7.16 (m, 2H, ArC*H* x 2), 7.07 (d, *J* = 7.8 Hz, 2H, ArC*H* x 2), 6.99 (d, *J* = 7.8 Hz, 2H, ArC*H* x 2), 4.84 (t, *J* = 7.0 Hz, 1H, O*H*), 4.44 (td, *J* = 11.7, 8.9 Hz, 1H, HNC*H*), 4.19 (d, *J* = 11.8 Hz, 1H, N*H*), 3.79 (dd, *J* = 13.4, 7.2 Hz, 2H, HOCH_2), 3.10 (dt, *J* = 8.6, 4.1 Hz, 1H, ArC≡CC*H*), 2.28 (s, 3H, -CH_3).

^{13}C NMR (126 MHz, $CDCl_3$) δ ppm 142.0 (d, *J* = 5.9 Hz, Ar*C*H), 137.9 (Ar*C*), 133.1 (d, *J* = 9.9 Hz, Ar*C*H x 2), 132.4 (d, *J* = 2.4 Hz, Ar*C*H), 132.3 (d, *J* = 127.2 Hz, Ar*C*), 132.3 (d, *J* = 2.6 Hz, Ar*C*H), 131.6 (d, *J* = 8.9 Hz, Ar*C*H x 2), 131.5 (Ar*C*H x 2), 130.8 (d, *J* = 133.3 Hz, Ar*C*), 128.9 (Ar*C*H x 2), 128.8 (d, *J* = 12.8 Hz, Ar*C*H x 2), 128.5 (d, *J* = 13.0 Hz, Ar*C*H x 2), 128.4 (Ar*C*H x 2), 127.6 (Ar*C*H), 127.2 (Ar*C*H x 2), 120.3 (Ar*C*), 87.3 (Ar*C*≡C), 85.0 (ArC≡*C*), 62.5 (HO*C*H_2), 55.8 (NH*C*H), 44.4 (ArCC*C*H), 21.5 (*C*H_3).

^{31}P NMR (202 MHz, $CDCl_3$) δ ppm 25.70.

IR ν_{max} (neat/cm^{-1}): 3309, 3198, 2928, 1448, 1432, 1375, 1187, 1152, 1080, 997.

HRMS : calcd for $C_{30}H_{29}NO_2P$ $[M + H]^+$ 466.1930, found 466.1930.

Specific rotation: $[\alpha]_D^{29}$ - 29.5 (c 0.41, $CHCl_3$).

Enantiomeric purity of **4b** was determined by HPLC analysis in comparison with authentic racemic material (ee >99%; **OD-H** column, 90:10 hexanes: *i*-PrOH, 0.5 mL/min, 20 °C, 254 nm)

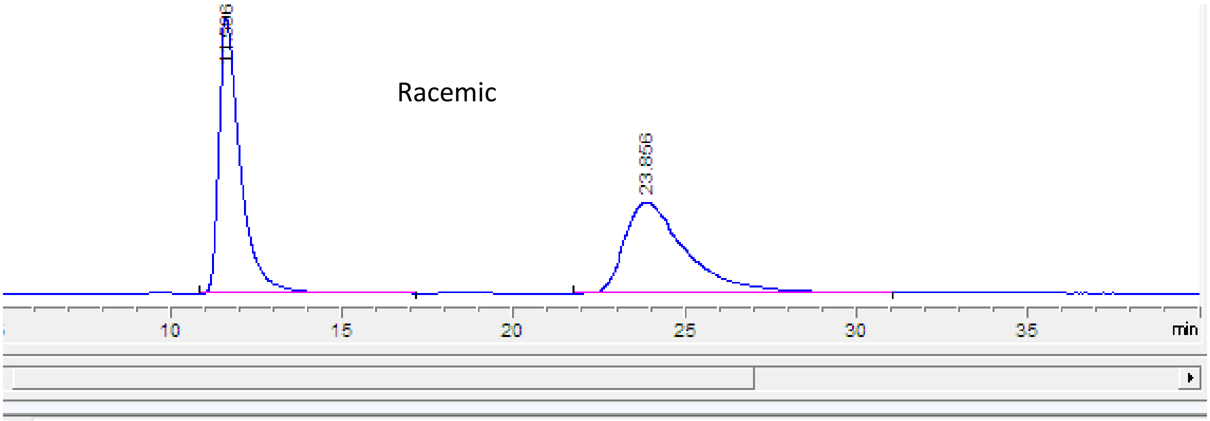

#	Time	Area	Height	Width	Area%	Symmetry
1	11.596	8896.4	197.2	0.6703	52.214	0.504
2	23.856	8142.1	64.9	1.6608	47.786	0.509

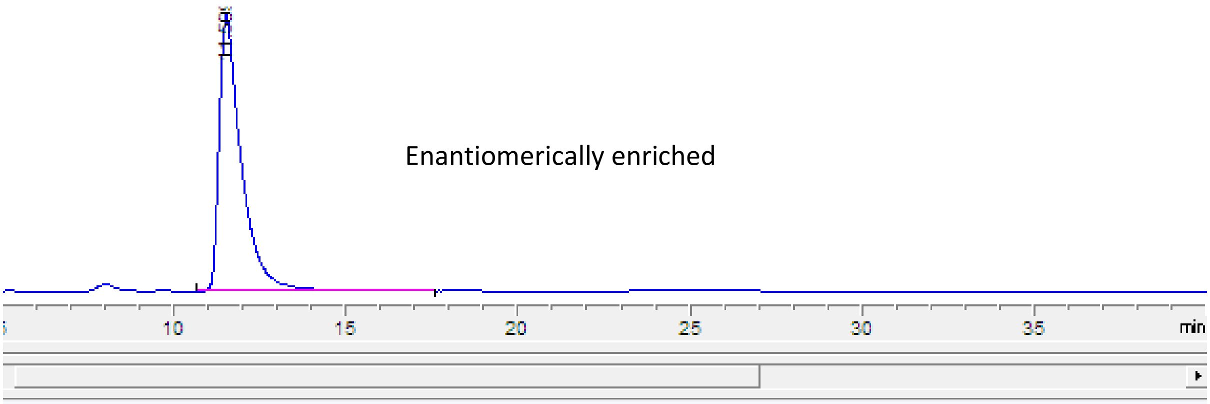

#	Time	Area	Height	Width	Area%	Symmetry
1	11.509	27183.7	588.5	0.6749	100.000	0.453

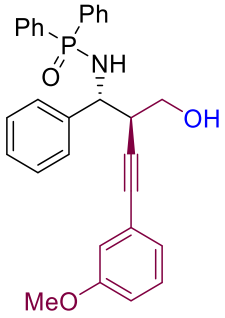

***N*-((1*R*,2*S*)-2-(Hydroxymethyl)-4-(3-methoxyphenyl)-1-phenylbut-3-yn-1-yl)-*P*,*P*-diphenylphosphinic amide (4c)**

Compound **4c** was prepared according to general procedure B. The title compound was isolated by column chromatography (hexane: ethyl acetate, 60:40-20:80) as a white amorphous solid (43.3 mg, 0.09 mmol, 45%). Mp = 178-179°C ($CHCl_3$).

^{1}H NMR (500 MHz, $CDCl_3$) δ ppm 7.81 (dd, *J* = 12.1, 7.1 Hz, 2H, ArC*H* x 2), 7.68 (dd, *J* = 12.4, 7.6 Hz, 2H, ArC*H* x 2), 7.50 (t, *J* = 8.9 Hz, 1H, ArC*H*), 7.46 (t, *J* = 7.4 Hz, 1H, ArC*H*), 7.42 (td, *J* = 7.6, 3.1 Hz, 2H, ArC*H* x 2), 7.38 – 7.28 (m, 6H, ArC*H* x 6), 7.21 (d, *J* = 6.2 Hz, 2H, ArC*H* x 2), 7.08 (t, *J* = 7.9 Hz, 1H, ArC*H*), 6.76 (d, *J* = 8.1 Hz, 2H, ArC*H* x 2), 6.69 (s, 1H, ArC*H*), 4.98 (t, *J* = 7.2 Hz, 1H N*H*), 4.50 – 4.35 (m, 1H NHC*H*), 4.25 – 4.17 (q, *J* = 11.4Hz, 1H C*H*$_2$OH), 3.88 – 3.74 (m, 2H C*H*$_2$OH, OH), 3.71 (s, 3H), 3.10 (dt, *J* = 8.5, 4.0 Hz, 1H ArC≡CC*H*).

^{13}C NMR (125 MHz, $CDCl_3$) δ ppm 158.9 (Ar*C*), 141.8 (d, *J* = 5.7 Hz, Ar*C*), 132.9 (d, *J* = 10.0 Hz, Ar*C*H x 2), 132.2 (d, *J* = 2.8 Hz, Ar*C*H), 132.1 (d, *J* = 2.8 Hz, Ar*C*H), 132.1 (d, *J* = 127.3 Hz, Ar*C*), 131.3 (d, *J* = 9.7 Hz, Ar*C*H x 2), 130.4 (d, *J* = 133.1 Hz, Ar*C*), 128.9 (Ar*C*H), 128.6 (d, *J* = 12.6 Hz, Ar*C*H x 2), 128.3 (d, *J* = 12.9 Hz, Ar*C*H x 2), 128.2 (Ar*C*H x 2), 127.4 (Ar*C*H), 127.0 (Ar*C*H x 2), 124.1 (Ar*C*H), 123.9 (Ar*C*), 116.2 (Ar*C*H), 114.4 (Ar*C*H), 87.8 (Ar*C*≡C), 84.5 (ArC≡*C*), 62.1 (C-OH), 55.6 (HN-C), 55.1 (s, OMe), 44.1 (ArC≡CCH).

^{31}P NMR (202 MHz, $CDCl_3$) δ ppm 25.92.

IR ν_{max} (neat/cm−1): 3277, 2947, 2360, 2340, 1486, 1435, 1160, 1062, 877, 755, 700.

HRMS : calcd for $C_{30}H_{28}NO_3P$ $[M + H]^+$: 482.1834, found 482.1871.

Specific rotation: $[\alpha]_D^{28.0}$ - 41.1 (c 2.2, $CHCl_3$).

Enantiomeric purity of **4c** was determined by HPLC analysis in comparison with authentic racemic material (ee = 91.4%; **OD-H** column, 80:20 hexanes: *i*-PrOH, 1 mL/min, 20 °C, 254 nm).

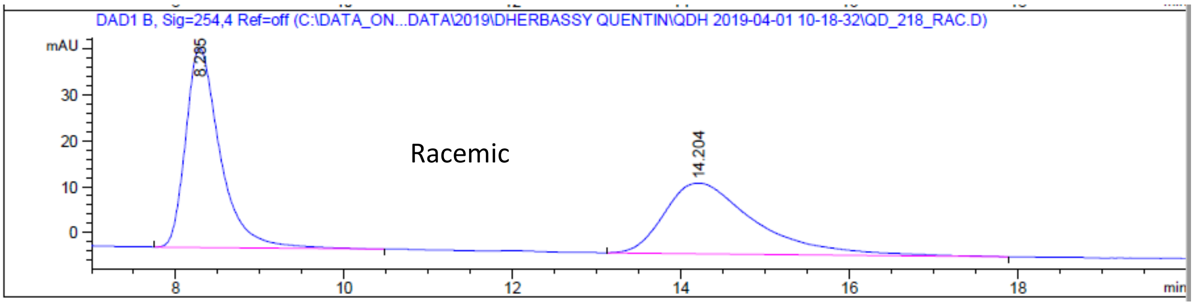

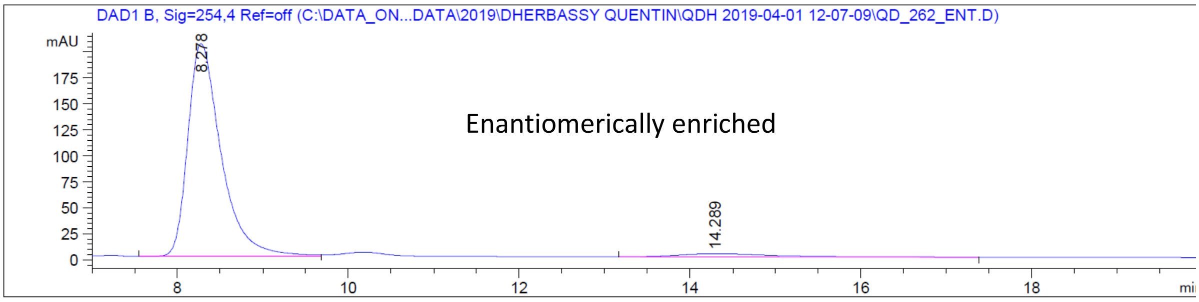

Signal 2: DAD1 B, Sig=254,4 Ref=off

Peak #	RetTime [min]	Type	Width [min]	Area [mAU*s]	Height [mAU]	Area %
1	8.278	BV	0.4091	5565.03418	204.77350	95.6906
2	14.289	BB	0.9850	250.62141	3.20265	4.3094

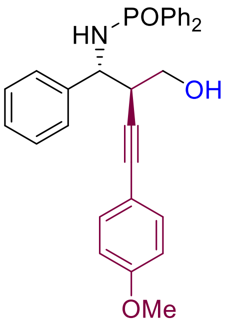

***N*-((1*R*,2*S*)-2-(Hydroxymethyl)-4-(4-methoxyphenyl)-1-phenylbut-3-yn-1-yl)-*P*,*P*-diphenylphosphinic amide (4d)**

Compound **4d** was prepared according to general procedure B. The title compound was isolated by column chromatography (hexane: ethyl acetate, 60:40-20:80) as a white amorphous solid (70.0 mg, 0.166 mmol, 83%). M.p.: 188-192 ˚C ($CHCl_3$).

^{1}H NMR (400 MHz, $CDCl_3$) δ ppm 7.8 – 7.8 (m, 2H, ArC*H* x 2), 7.7 – 7.6 (m, 2H, ArC*H* x 2), 7.48 (qd, *J* = 7.5, 1.5 Hz, 2H, ArC*H* x 2), 7.44 – 7.38 (m, 2H, ArC*H* x 2), 7.4 – 7.3 (m, 5H, ArC*H* x 5), 7.22 (dd, *J* = 7.7, 1.8 Hz, 2H, ArC*H* x 2), 7.11 (d, *J* = 8.7 Hz, 2H, ArC*H* x 2), 6.71 (d, *J* = 8.8 Hz, 2H, ArC*H* x 2), 4.87 (t, *J* = 6.7 Hz, 1H, OH), 4.44 (td, *J* = 11.7, 8.7 Hz, 1H, NHC*H*), 4.17 (ddd, *J* = 11.7, 5.3, 3.6 Hz, 1H, HOCH_2), 3.94 – 3.82 (m, 1H, N*H*), 3.81 – 3.76 (m, 1H, HOCH_2), 3.74 (s, 3H, OCH_3), 3.11 (dt, *J* = 8.6, 4.2 Hz, 1H, ArCCC*H*).

^{13}C NMR (101 MHz, $CDCl_3$) δ ppm 159.3 (Ar*C*), 142.0 (d, *J* = 5.8 Hz, Ar*C*), 133.1 (d, *J* = 10.1 Hz, Ar*C*H x 2), 133.1 (d, *J* = 131.1, Hz, Ar*C*), 133.0 (Ar*C*H x 2), 132.4 (d, *J* = 2.8 Hz, Ar*C*H), 132.2 (d. *J* = 2.7 Hz, Ar*C*H), 131.6 (d, *J* = 9.8 Hz, Ar*C*H x 2), 130.8 (d, *J* = 133.2 Hz, Ar*C*), 128.8 (d, *J* = 12.6 Hz, Ar*C*H x 2), 128.5 (d, *J* = 13.0 Hz, Ar*C*H x 2), 128.3 (Ar*C*H x 2), 127.6 (Ar*C*H), 127.3 (Ar*C*H x 2), 115.5 (Ar*C*), 113.8 (Ar*C*H x 2), 86.5 (Ar*C*≡C), 84.7 (ArC≡*C*), 62.5 (HO*C*H_2), 55.8 (NH*C*H), 55.3 (O*C*H_3), 44.4 (d, *J* = 2.6 Hz, ArCC*C*H).

IR ν_{max} (neat/cm^{-1}): 3285, 3050, 2918, 1515, 1435, 1306, 1270, 1067, 1035, 911.

HRMS : calcd for $C_{30}H_{29}NO_3P$ $[M + H]^+$ 482.1880, found 482.1881.

Specific rotation: $[\alpha]_D^{26.5}$ + 14.10 (c 0.52, $CHCl_3$).

Enantiomeric purity of **4d** was determined by HPLC analysis in comparison with authentic racemic material (ee >99%; **OD-H** column, 90:10 hexanes: *i*-PrOH, 1 mL/min, 20 °C, 254 nm)

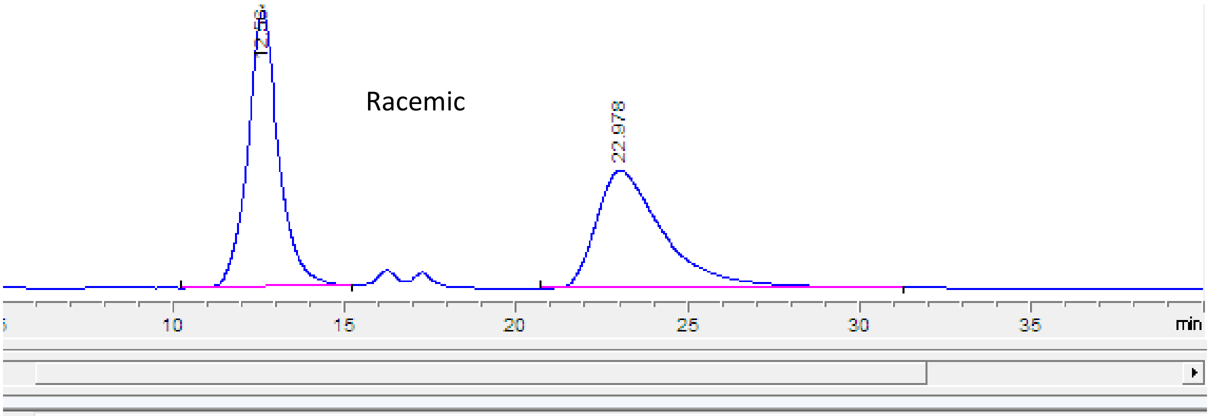

#	Time	Area	Height	Width	Area%	Symmetry
1	12.564	16221.1	258.5	0.9161	51.920	0.81
2	22.978	15021.2	110.2	1.8121	48.080	0.496

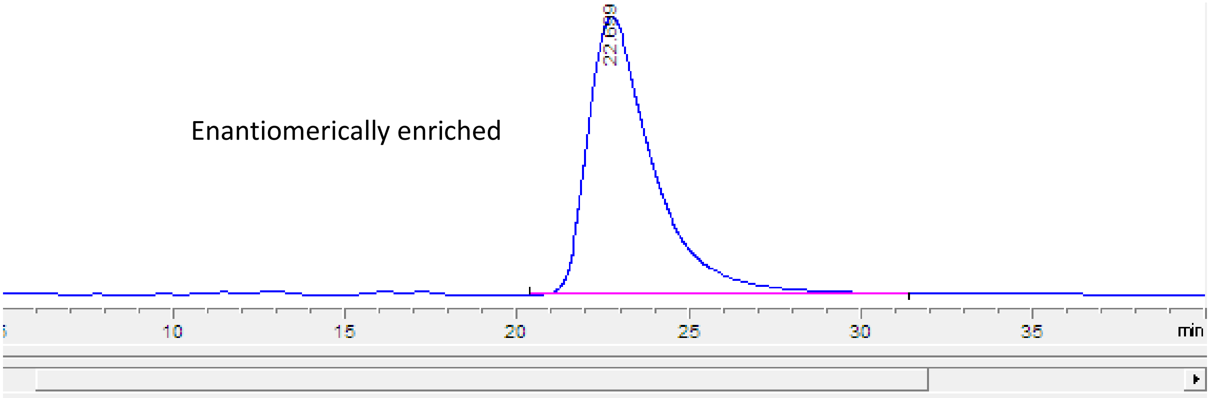

#	Time	Area	Height	Width	Area%	Symmetry
1	22.699	24832.5	188.8	1.8887	100.000	0.509

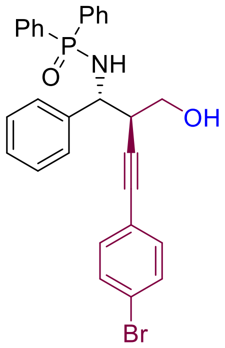

***N*-((1*R*,2*S*)-1-(4-Bromophenyl)-2-(hydroxymethyl)-4-phenylbut-3-yn-1-yl)-*P*,*P*-diphenylphosphinic amide (4e)**

Compound **4e** was prepared according to general procedure B. The title compound was isolated by column chromatography (hexane: ethyl acetate, 60:40-20:80) as a white amorphous solid (72.1 mg, 0.136 mmol, 68%). M.p. = 182-183°C ($CHCl_3$).

^{1}H NMR (400 MHz, $CDCl_3$) δ ppm 7.80 (dd, *J* = 11.9, 7.4 Hz, 2H, ArC*H* x 2), 7.67 (dd, *J* = 12.5, 7.5 Hz, 2H, ArC*H* x 2), 7.55 – 7.44 (m, 2H, ArC*H* x 2), 7.42 (td, *J* = 7.7, 3.1 Hz, 2H, ArC*H* x 2), 7.37 – 7.24 (m, 7H, ArC*H* x 7), 7.18 (d, *J* = 5.8 Hz, 2H, ArC*H* x 2), 7.00 (d, *J* = 8.2 Hz, 2H, ArC*H* x 2), 5.15 (t, *J* = 7.0 Hz, 1H O*H*), 4.42 (q, *J* = 10.7 Hz, 1H NHC*H*), 4.26 (d, *J* = 11.8 Hz, 1H C*H*$_2$OH), 3.89 – 3.78 (m, 1H C*H*$_2$OH), 3.74 (t, *J* = 10.7 Hz, 1H N*H*), 3.04 (dt, *J* = 9.1, 3.4 Hz, 1H ArCCC*H*).

^{13}C NMR (101 MHz, $CDCl_3$) δ ppm 142.0 (d, *J* = 5.8 Hz, Ar*C*), 133.2 (d, *J* = 11.2 Hz, Ar*C*H x 2), 133.1 (Ar*C*H x 2), 132.5 (d, *J* = 2.6 Hz, Ar*C*H), 132.4 (d, *J* = 2.8 Hz, Ar*C*H), 132.2 (d, *J* = 127.2 Hz, Ar*C*), 131.5 (d, *J* = 9.8 Hz, Ar*C*H x 2), 131.4 (Ar*C*H x 2), 130.6 (d, *J* = 133.2 Hz, Ar*C*), 128.9 (d, *J* = 12.7 Hz, Ar*C*H x 2), 128.6 (d, *J* = 13.0 Hz, Ar*C*H x 2), 128.5 (Ar*C*H x 2), 127.7 (Ar*C*H), 127.1 (Ar*C*H

x 2), 122.4 (Ar*C*), 122.0 (Ar*C*), 89.7 (Ar*C*≡C), 83.7 (ArC≡*C*), 62.1 (HO*C*H_2), 55.7 (HNCH), 44.3 (ArC≡CCH).

^{31}P NMR (162 MHz, $CDCl_3$) δ ppm 26.29.

IR ν_{max} (neat/cm^{-1}): 3229, 3059, 2932, 2360, 2340, 1485, 1457, 1438, 1170, 1125, 1109, 1071, 727, 698.

HRMS : calcd for $C_{29}H_{25}BrNO_2P$ $[M+H]^+$: 532.0861, found 532.0852.

Specific rotation: $[\alpha]_D^{28.0}$ - 43.6 (c 0.66, $CHCl_3$).

Enantiomeric purity of **4e** was determined by HPLC analysis in comparison with authentic racemic material (ee > 99%; **OD-H** column, 80:20 hexanes: *i*-PrOH, 1 mL/min, 20 °C, 254 nm)

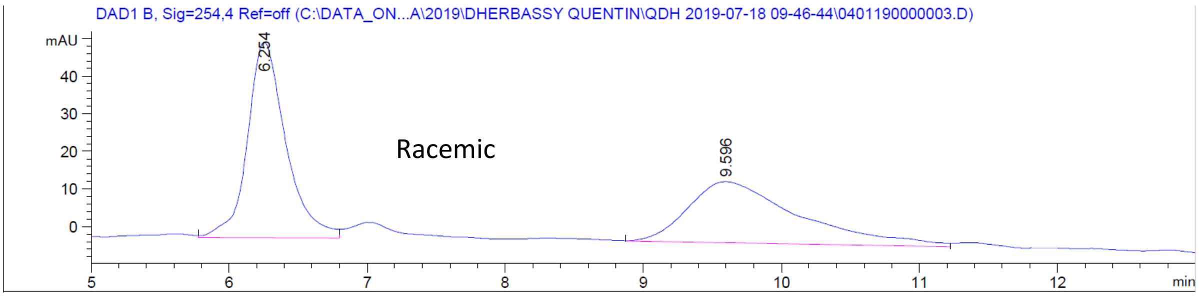

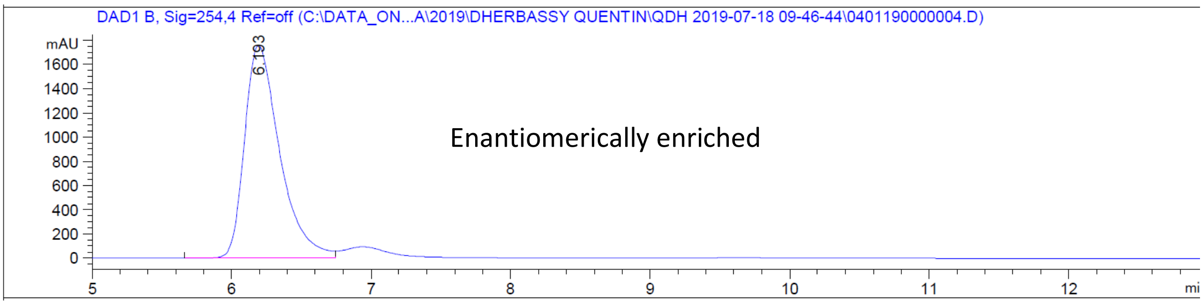

Signal 2: DAD1 B, Sig=254,4 Ref=off

Peak #	RetTime [min]	Type	Width [min]	Area [mAU*s]	Height [mAU]	Area %
1	6.193	BV	0.2669	3.08816e4	1758.34644	100.0000

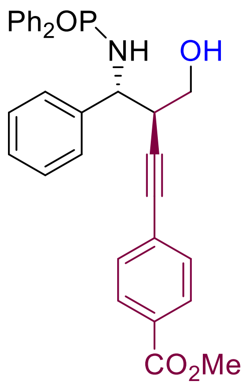

Methyl 4-((3*S*,4*R*)-4-((diphenylphosphoryl)amino)-3-(hydroxymethyl)-4-phenylbut-1-yn-1-yl)benzoate (4f)

Compound **4f** was prepared according to general procedure A. The title compound was isolated by column chromatography (hexane: ethyl acetate, 60:40-0:100) as a white amorphous solid (35.7 mg, 0.07 mmol, 35%). M. p.: 180-181 ˚C ($CHCl_3$).

^{1}H NMR (500 MHz, $CDCl_3$) δ ppm 7.84 (d, *J* = 8.6 Hz, 2H, , ArC*H* x 2), 7.80 (dd, *J* = 12.2, 8.1 Hz, 2H, ArC*H* x 2), 7.68 (dd, *J* = 12.5, 8.0 Hz, 2H, ArC*H* x 2), 7.52 (t, *J* = 7.5 Hz, 1H, ArC*H* x 1), 7.48 (t, *J* = 8.3 Hz, 1H, ArC*H* x 1), 7.43 (td, *J* = 7.4, 2.9 Hz, 2H, ArC*H* x 2), 7.39 – 7.29 (m, 5H, ArC*H* x 5), 7.22 – 7.15 (m, 4H, ArC*H* x 4), 5.15 (Brd s, 1H, OH), 4.43 (q, *J* = 11.2 Hz, 1H, NHC*H*), 4.31 (d, *J* = 11.9 Hz, 1H, HOCH_2), 3.87 (s, 4H, OCH_3 + N*H*), 3.60 (t, *J* = 10.9 Hz, 1H, HOCH_2), 3.18 – 2.93 (m, 1H, PhCCC*H*).

^{13}C NMR (126 MHz, $CDCl_3$) δ ppm 166.6 (*C*O_2Me), 141.9 (d, *J* = 5.6 Hz, Ar*C*), 133.1 (d, *J* = 10.1 Hz, Ar*C*H x 2), 132.4 (d, *J* = 2.4 Hz, Ar*C*H), 132.3 (d, *J* = 2.7 Hz, Ar*C*H), 132.0 (d, *J* = 127.2 Hz, Ar*C*), 131.4 (Ar*C*H x 2), 131.3 (d, *J* = 9.8 Hz, Ar*C*H x 2), 130.3 (d, *J* = 133.2 Hz, Ar*C*), 129.2 (Ar*C*H x 2), 129.0 (Ar*C*), 128.7 (d, *J* = 12.7 Hz, Ar*C*H x 2), 128.5 (d, *J* = 12.7 Hz, Ar*C*H x 2), 128.4 (Ar*C*H x 2), 128.0 (Ar*C*H), 127.7 (Ar*C*), 126.9 (Ar*C*H x 2), 91.6 (Ph*C*≡C), 84.0 (PhC≡*C*), 62.0 (*C*H_2OH), 55.6 (CO_2*C*H_3), 52.1 (d, *J* = 6.3 Hz, NH*C*H), 44.3 (PhCC*C*H).

^{31}P NMR (202 MHz, $CDCl_3$) δ ppm 26.35.

IR ν_{max} (neat/cm^{-1}): 3293, 2969, 2870, 1712, 1603, 1434, 1273, 1154, 1028, 951, 856, 691.

HRMS : calcd for $C_{31}H_{28}NO_4PNa$ [M + Na]$^+$: 532.1648, found 532.1635.

Specific rotation: $[\alpha]_D^{29.3}$ - 3.61 (c 0.97, $CHCl_3$).

Enantiomeric purity of **3i** was determined by HPLC analysis in comparison with authentic racemic material (ee = 32%; **OD-H** column, 90:10 hexanes: *i*-PrOH, 1 mL/min, 20 °C, 254 nm)

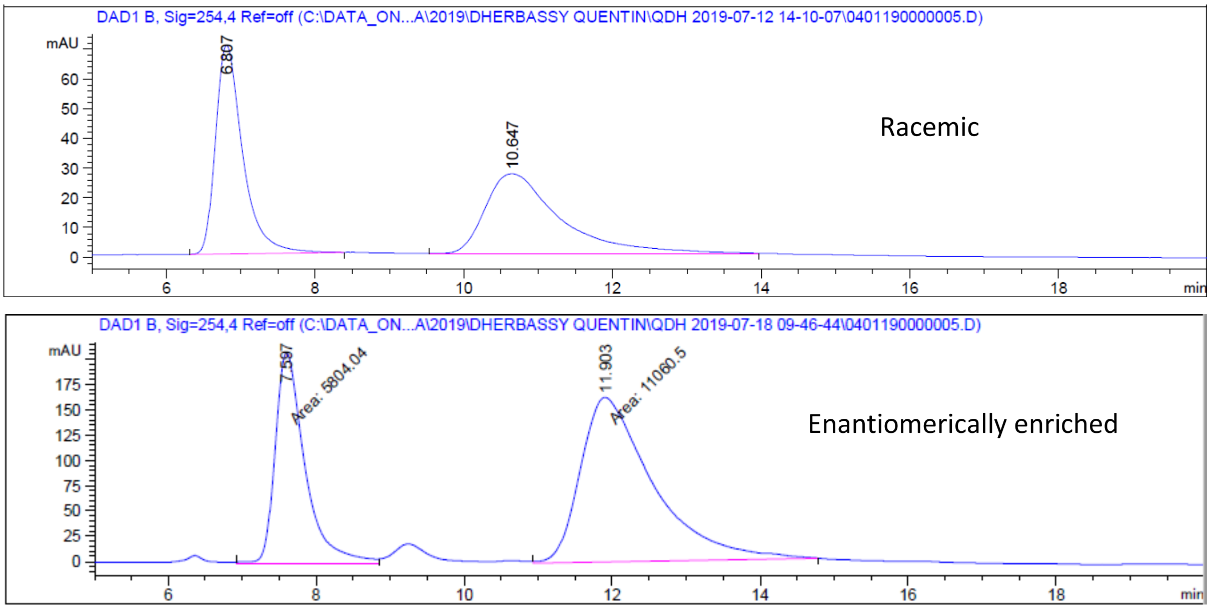

Signal 2: DAD1 B, Sig=254,4 Ref=off

Peak #	RetTime [min]	Type	Width [min]	Area [mAU*s]	Height [mAU]	Area %
1	7.597	MM	0.4654	5804.03955	207.86105	34.4157
2	11.903	MM	1.1337	1.10605e4	162.59921	65.5843

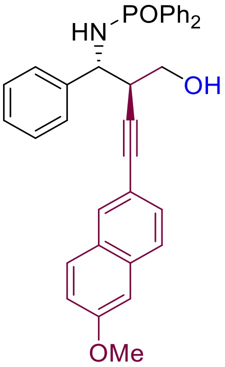

***N*-((1*R*,2*S*)-2-(Hydroxymethyl)-4-(6-methoxynaphthalen-2-yl)-1-phenylbut-3-yn-1-yl)-*P*,*P*-diphenylphosphinic amide (4g)**

Compound **4g** was prepared according to general procedure B. The title compound was isolated by column chromatography (hexane: ethyl acetate, 60:40-20:80) as a white amorphous solid (77.0 mg, 0.144 mmol, 72%). M.p.: > 200 ˚C ($CHCl_3$).

^{1}H NMR (500 MHz, DMSO-*d*$_6$) δ ppm 7.81 – 7.71 (m, 3H, ArC*H* x 3), 7.73 – 7.67 (m, 2H, ArC*H* x 2), 7.62 – 7.52 (m, 3H, ArC*H* x 3), 7.49 (dt, *J* = 13.0, 6.2 Hz, 3H, ArC*H* x 3), 7.4 – 7.3 (m, 6H, ArC*H* x 6), 7.26 (dd, *J* = 9.2, 4.8 Hz, 2H, ArC*H* x 2), 7.20 (d, *J* = 8.5 Hz, 1H, ArC*H*), 7.14 (dd, *J* = 9.0, 2.4 Hz, 1H, ArC*H*), 6.29 (t, *J* = 11.0 Hz, 1H, NC*H*), 5.40 (t, *J* = 6.3 Hz, 1H, O*H*), 4.23 (td, *J* =

11.5, 8.4 Hz, 1H, HNC*H*), 3.95 – 3.89 (m, 1H, HOC*H*$_2$), 3.84 (s, 3H, OCH$_3$), 3.62 – 3.54 (m, 1H, HO-*CH*$_2$), 3.11 (dt, *J* = 8.8, 4.5 Hz, 1H, ArC≡CC*H*).

^{13}C NMR (126 MHz, DMSO) δ ppm 157.9 (Ar*C*), 142.53 (d, *J* = 5.3 Hz, Ar*C*), 133.6 (Ar*C*), 133.0 (d, *J* = 126.9 Hz. Ar*C*), 132.7 (d, *J* = 130.3 Hz, Ar*C*), 132.2 (d, *J* = 9.8 Hz, Ar*C*H x 2), 132.0 (d, *J* = 2.1 Hz, Ar*C*H), 131.8 (d, *J* = 2.2 Hz, Ar*C*H), 131.6 (d, *J* = 9.5 Hz, Ar*C*H x 2), 130.5 (Ar*C*H), 129.2 (Ar*C*), 128.8 (ArCH), 128.7 (d, *J* = 12.2 Hz, Ar*C*H x 2), 128.4 (d, *J* = 12.3 Hz, Ar*C*H x 2), 128.0 (Ar*C*H), 127.8 (Ar*C*H), 127.6 (Ar*C*H x 2), 127.1 (Ar*C*H), 126.9 (Ar*C*H), 119.3 (Ar*C*), 118.0 (Ar*C*H), 106.1 (Ar*C*H), 89.7 (Ar*C*≡C), 84.1 (ArC≡*C*), 61.4 (HO*C*H$_2$), 55.4 (*O*CH$_3$), 55.1 (NH*C*H), 43.5 (ArC≡C*C*H).

^{31}P NMR (202 MHz, DMSO) δ ppm 23.32.

IR ν_{max} (neat/cm^{-1}): 3303, 3056, 2933, 1628, 1600, 1435, 1387, 1271, 1241, 1157, 1063, 936.

HRMS : calcd for $C_{34}H_{31}NO_3P$ $[M + H]^+$: 532.2036, found 532,2039.

Specific rotation: $[\alpha]_D^{28}$ - 84.5 (c 0.29, CHCl$_3$).

Enantiomeric purity of **4g** was determined by HPLC analysis in comparison with authentic racemic material (ee: 98.5%; **AD-H** column, 90:10 hexanes: *i*-PrOH, 1 mL/min, 20 °C, 254 nm)

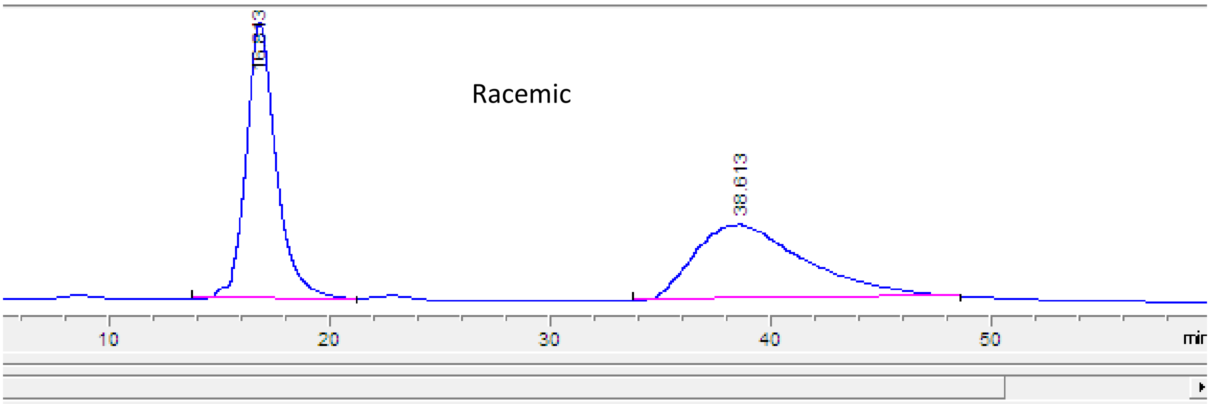

#	Time	Area	Height	Width	Area%	Symmetry
1	16.813	19484.4	202.2	1.3921	50.404	0.783
2	38.613	19171.9	55	4.1221	49.596	0.683

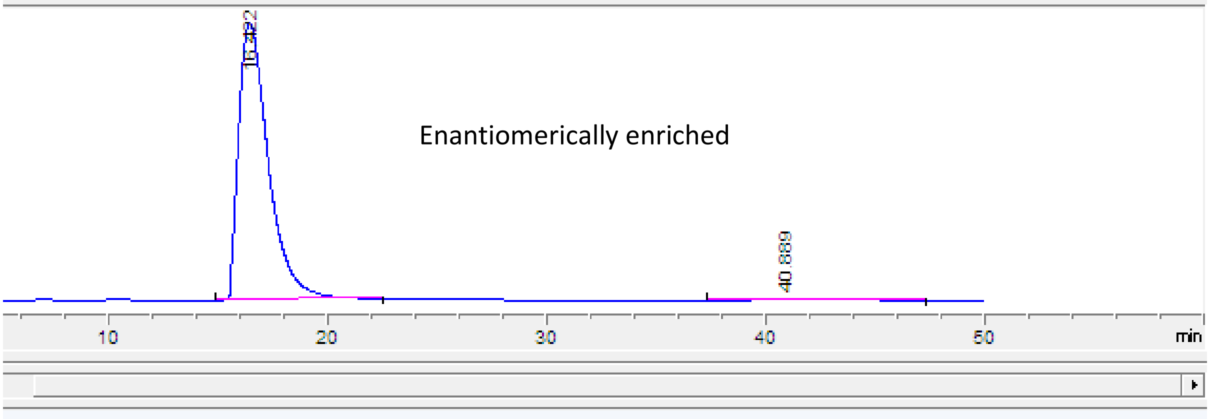

#	Time	Area	Height	Width	Area%	Symmetry
1	16.422	176448	1863.7	1.4126	99.249	0.526
2	40.889	1335.8	4.9	3.1766	0.751	0.526

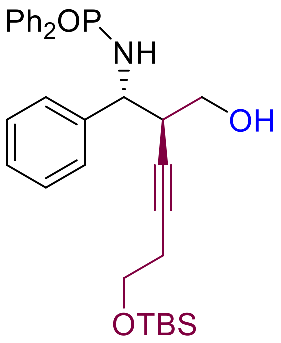

N-((1*R*,2*S*)-7-((*tert*-Butyldimethylsilyl)oxy)-2-(hydroxymethyl)-1-phenylhept-3-yn-1-yl)-*P*,*P*-diphenylphosphinic amide (4h)

Compound **4h** was prepared according to general procedure A (0.18 mmol scale). The title compound was isolated by column chromatography (hexane: ethyl acetate, 60:40-20:80) as a white amorphous solid (28 mg, 0.052 mmol, 29%). M. p.: n.d - amorphous.

^{1}H NMR (500 MHz, CDCl$_3$) δ ppm 7.92 (dd, *J* = 12.1, 7.6 Hz, 2H, ArC*H* x 2), 7.79 (dd, *J* = 12.5, 7.6 Hz, 2H, ArC*H* x 2), 7.63 (t, *J* = 7.6 Hz, 1H, ArC*H* x 1), 7.58 (t, *J* = 7.5 Hz, 1H, ArC*H* x 1), 7.58 – 7.51 (m, 2H, ArC*H* x 2), 7.48 – 7.38 (m, 5H, ArC*H* x 5), 7.27 (d, *J* = 6.9 Hz, 2H, ArC*H* x 2), 4.87 (t, *J* = 6.9 Hz, 1H, OH), 4.43 (q, *J* = 11.0 Hz, 1H, NHC*H*), 4.25 – 4.15 (m, 1H, HOCH_2), 3.85 (t, *J* = 10.5 Hz, 1H, NH), 3.81 – 3.73 (m, 1H, HOCH_2), 3.59 (app. h, *J* = 9.3 Hz, 2H, CH_2OTBS), 2.99 (Brd s, 1H, PhCCC*H*), 2.35 (t, *J* = 7.1 Hz, 2H, CH_2CC), 0.96 (s, 6H, Si(CH_3)$_2$), 0.11 (s, 9H, C(CH_3)$_3$).

^{13}C NMR (126 MHz, CDCl$_3$) δ ppm 141.9 (d, *J* = 5.8 Hz, Ar*C*), 132.95 (d, *J* = 10.1 Hz, Ar*C*H x 2), 132.3 (d, *J* = 127.3 Hz, Ar*C*), 132.2 (d, *J* = 2.6 Hz, Ar*C*H), 132.1 (d, *J* = 2.8 Hz, Ar*C*H), 131.4 (d, *J* = 9.8 Hz, Ar*C*H x 2), 130.7 (d, *J* = 133.3 Hz, Ar*C*), 128.6 (d, *J* = 12.6 Hz, Ar*C*H x 2), 128.3 (d, *J* = 13.0 Hz, Ar*C*H x 2), 128.1 (Ar*C*H x 2), 127.3 (Ar*C*H), 127.1 (Ar*C*H x 2), 81.5 (Ph*C*≡C), 79.2

(PhC≡*C*), 62.4 (*C*H_2OH), 62.0 (*C*H_2OTBS), 55.5 (d, *J* = 4.0 Hz, NH*C*H), 43.5 (PhCC*C*H), 25.8 (Si-C(*C*H_3)$_3$ x 3), 23.0 *C*H_2-CC), 18.2 (Si-*C*(CH_3)$_3$), -5.4 (Si-*C*H_3 x 2).

^{31}P NMR (202 MHz, CDCl$_3$) δ ppm 25.58.

IR ν_{max} (neat/cm^{-1}): 3377, 3228, 2929, 2856, 1438, 1256, 1163, 1103, 835, 650.

HRMS : calcd for $C_{31}H_{40}NO_3PNaSi$ [M + Na]$^+$: 556.2407, found 556.2391.

Specific rotation: $[\alpha]_D^{21}$ - 5.1 (c 0.68, CHCl$_3$).

Enantiomeric purity of **4h** was determined by HPLC analysis in comparison with authentic racemic material (ee = 94%; **OD-H** column, 95:5 hexanes: *i*-PrOH, 1 mL/min, 20 °C, 220 nm)

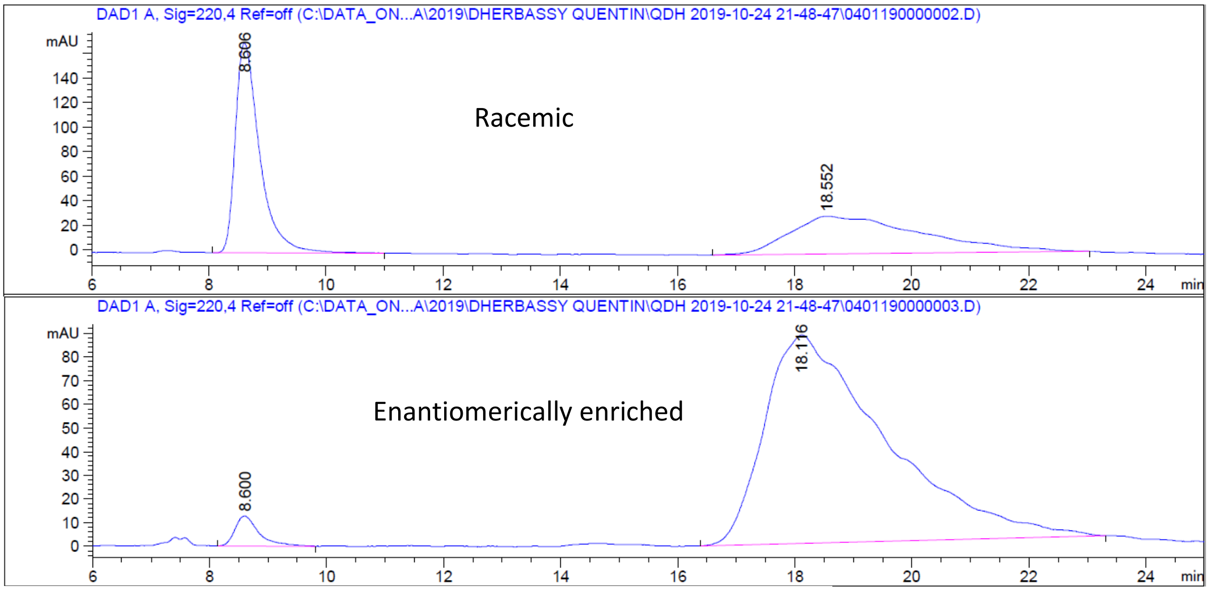

Signal 1: DAD1 A, Sig=220,4 Ref=off

Peak #	RetTime [min]	Type	Width [min]	Area [mAU*s]	Height [mAU]	Area %
1	8.600	BB	0.4223	361.68173	12.69838	2.7694
2	18.116	BB	1.7601	1.26984e4	88.18010	97.2306

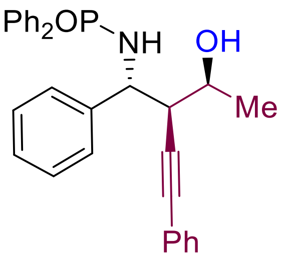

***N*-((1*R*,2*S*)-2-((*S*)-1-Hydroxyethyl)-1,4-diphenylbut-3-yn-1-yl)-*P*,*P*-diphenylphosphinic amide (6a)**

Compound **6a** was prepared according to general procedure B using (*Z*)-enyne. The title compound was isolated by column chromatography (hexane: ethyl acetate, 60:40-20:80) as a white amorphous solid (61.4 mg, 0.132 mmol, 66% (mixture of diastereomers, 70:30). (Major diastereomer was isolated 26, mg, 0.056 mmol, 28%), M.p.: 138-142 ˚C ($CHCl_3$).

^{1}H NMR (400 MHz, Acetone-*d*$_6$) δ ppm 7.97 – 7.87 (m, 2H, ArC*H* x 2), 7 7.78 – 7.72 (m, 2H, ArC*H* x 2), 7.57 – 7.44 (m, 3H, ArC*H* x 3), 7.50 – 7.37 (m, 5H, ArC*H* x 5), 7.37 – 7.23 (m, 9H, ArC*H* x 9), 5.38

(d, *J* = 5.3 Hz, 1H, O*H*), 5.20 – 5.04 (m, 1H, N*H*), 4.71 – 4.50 (m, 1H, HOC*H*), 4.18 – 4.07 (m, 1H, NHC*H*), 2.97 (dd, *J* = 4.1, 2.4 Hz, 1H, PhCCC*H*), 1.30 (d, *J* = 6.3 Hz, 3H, CH_3).

^{13}C NMR (101 MHz, Acetone-*d*$_6$) δ ppm 144.0 (d, *J* = 5.0 Hz, Ar*C*), 133.5 (d, *J* = 127.8 Hz, Ar*C*), 132.5 (d, *J* = 9.6 Hz, Ar*C*H x 2), 132.4 (d, *J* = 128.7 Hz, Ar*C*), 131.8 (d, *J* = 2.7 Hz, Ar*C*H x 2), 131.8 (d, *J* = 9.4 Hz, Ar*C*H x 2), 131.7 (d, *J* = 2.8 Hz, Ar*C*H), 131.6 (Ar*C*H x 2), 128.4 (d, *J* = 12.5 Hz, Ar*C*H x 2), 128.3 (Ar*C*H x 2), 128.2 (d, *J* = 12.8 Hz, Ar*C*H x 2), 128.0 (Ar*C*H x 2), 127.8 (Ar*C*H), 126.9 (Ar*C*H), 126.7 (Ar*C*H x 2), 123.9 (Ar*C*), 86.6 (Ph*C*≡C), 86.2 (PhC≡*C*), 68.4 (HO*C*H_2), 57.4 (NH*C*H), 50.4 (d, *J* = 3.9 Hz, PhCC*C*H), 22.1 (*C*H_3).

^{31}P NMR (162 MHz, Acetone-*d*$_6$) δ ppm 23.70.

IR ν_{max} (neat/cm^{-1}): 3350, 3058, 2927, 1488, 1334, 1248, 1170, 1170, 1108, 1068, 1024.

HRMS : calcd for $C_{30}H_{28}NO_2PNa$ $[M + Na]^+$: 488.1728, found 488.1729.

Enantiomeric purity of **6b** and **6b'** was determined by HPLC analysis in comparison with authentic racemic material (ee >99% (major) and 99% (minor); **OD-H** column, 94:6 hexanes: *i*-PrOH, 0.4 mL/min, 20 °C, 254 nm)

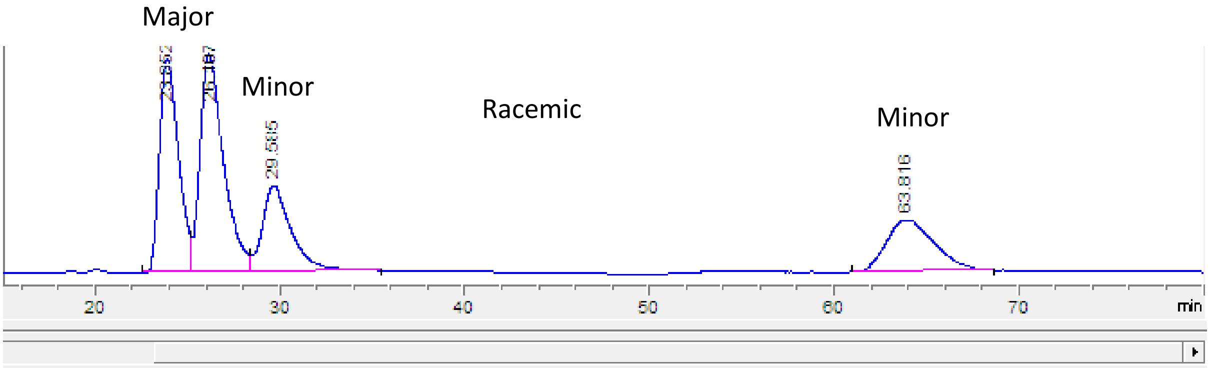

#	Time	Area	Height	Width	Area%	Symmetry
1	23.852	4488.7	64.6	1.089	28.290	0.627
2	26.107	5740.3	66.1	1.2671	36.177	0.543
3	29.585	2839.3	25.9	1.5602	17.895	0.565
4	63.816	2798.7	15.6	2.1116	17.638	0.647

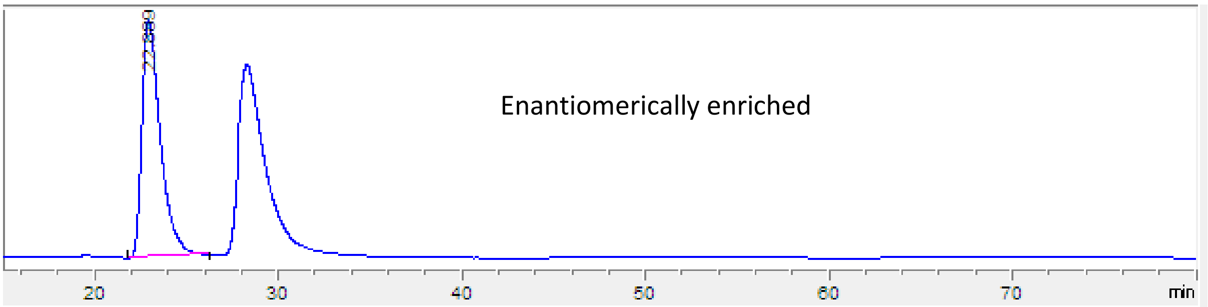

#	Time	Area	Height	Width	Area%	Symmetry
1	22.889	9832	145.1	1.0247	100.000	0.498

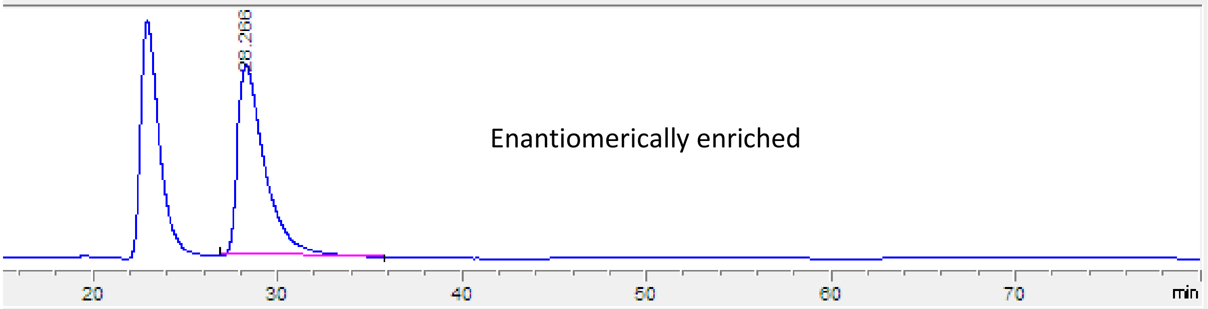

#	Time	Area	Height	Width	Area%	Symmetry
1	28.266	11981.5	116.7	1.5039	100.000	0.463

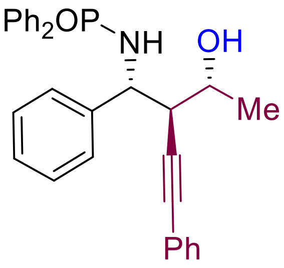

N-((1*R*,2*S*)-2-((*R*)-1-Hydroxyethyl)-1,4-diphenylbut-3-yn-1-yl)-P,P-diphenylphosphinic amide (6b)

Compound **6b** was prepared according to general procedure B using (*E*)-enyne. The title compound was isolated by column chromatography (hexane: ethyl acetate, 60:40-20:80) as a white amorphous solid (79.1 mg, 0.170 mmol, 85%). M.p.: 156-161 ˚C ($CHCl_3$).

^{1}H NMR (400 MHz, Acetone-*d*$_6$) δ ppm 7.90 – 7.85 (m, 2H, ArC*H* x 2), 7.73 (ddd, *J* = 12.0, 8.2, 1.4 Hz, 2H, ArC*H* x 2), 7.58 – 7.55 (m, 2H, ArC*H* x 2), 7.53 – 7.47 (m, 1H, ArC*H*), 7.37 – 7.31 (m,

4H, ArC*H* x 4), 7.30 – 7.25 (m, 6H, ArC*H* x 6), 5.29 (dd, *J* = 11.7, 8.8 Hz, 1H, N*H*), 4.80 (td, *J* = 11.4, 5.5 Hz, 1H, NHC*H*), 4.54 (d, *J* = 6.9 Hz, 1H, O*H*), 3.62 – 3.46 (m, 1H, OHC*H*), 3.38 (dd, *J* = 8.8, 5.6 Hz, 1H, PHCCC*H*), 1.3 (d, *J* = 6.1 Hz, 3H, C*H*$_3$).

^{13}C NMR (101 MHz, Acetone-*d*$_6$) δ ppm 142.2 (d, *J* = 6.4 Hz, Ar*C*), 134.9 (d, *J* = 127.0 Hz, Ar*C*), 134.2 (d, *J* = 130.6 Hz, Ar*C*), 133.4 (d, *J* = 9.5 Hz, Ar*C*H x 2), 132.7 (d, *J* = 9.4 Hz, Ar*C*H x 2), 132.5 (d, *J* = 128.7 Hz), 132.5 (d, *J* = 2.7 Hz, Ar*C*H), 132.4 (d, *J* = 2.8 Hz, Ar*C*H), 132.3 (Ar*C*H x 2), 129.2 (Ar*C*H x 2), 129.2 (d, *J* = 10.3 Hz, Ar*C*H x 2), 129.1 (d, *J* = 13.0 Hz, Ar*C*H x 2), 129.0 (Ar*C*H x 2), 128.8 (Ar*C*H), 128.5 (Ar*C*H x 2), 128.0 (Ar*C*H), 124.6 (Ph*C*), 89.0 (Ph*C*≡C), 86.7 (PhC≡*C*), 68.3 (HO*C*H), 55.7 (NH*C*H), 51.5 (d, *J* = 3.0 Hz, PhCC*C*H), 22.7 (*C*H$_3$).

^{31}P NMR (162 MHz, Acetone-*d*$_6$) δ ppm 21.8.

IR ν_{max} (neat/cm^{-1}): 3351, 3059, 2926, 1490, 1455, 1412, 1253, 1186, 1124, 1028, 998.

HRMS : calcd for $C_{30}H_{28}NO_2PNa$ [M + Na]$^+$: 488.1728, found 488.1731.

Specific rotation: $[\alpha]_D^{27.3}$ + 68.5 (c 0.27, Acetone)

Enantiomeric purity of **6b** was determined by HPLC analysis in comparison with authentic racemic material (ee >99%; **OD-H** column, 95:5 hexanes: *i*-PrOH, 1.0 mL/min, 20 °C, 254 nm)

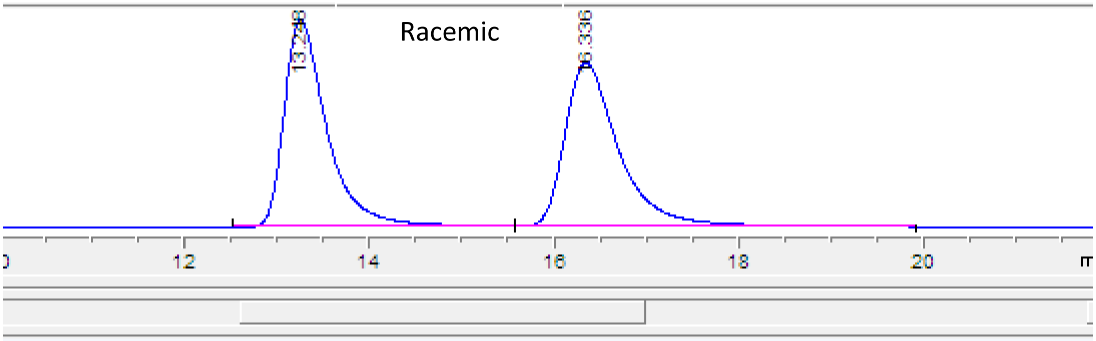

#	Time	Area	Height	Width	Area%	Sym
1	13.248	3582.6	110.2	0.4873	50.079	0.
2	16.336	3571.3	87.3	0.6134	49.921	0.

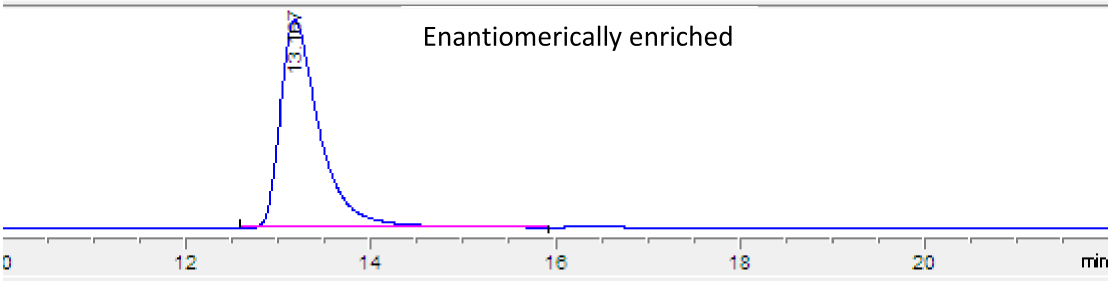

#	Time	Area	Height	Width	Area%	Symm
1	13.167	15295.7	515.6	0.4421	100.000	0.51

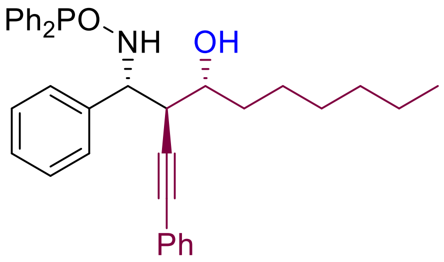

(3*S*,4*R*)-3-((*R*)-(((Diphenylphosphaneyl)oxy)amino)(phenyl)methyl)-1-phenyldec-1-yn-4-ol (6c)

Compound **6c** was prepared according to general procedure B using (*E*)-enyne. The title compound was isolated by column chromatography (hexane: ethyl acetate, 80:20-50:50) as a white amorphous solid (88.0 mg, 0.164 mmol, 82%). M.p.: 62-67 ˚C ($CHCl_3$).

^{1}H NMR (400 MHz, Acetone-*d*$_6$) δ ppm 7.95 – 7.84 (m, 2H, ArC*H* x 2), 7.79 – 7.70 (m, 2H, ArC*H* x 2), 7.64 – 7.57 (m, 2H, ArC*H* x 2), 7.54 – 7.47 (m, 1H, ArC*H* x 2), 7.47 – 7.40 (m, 3H, ArC*H* x 2), 7.37 – 7.20 (m, 10H, ArC*H* x 2), 5.28 (dd, *J* = 11.7, 8.4 Hz, 1H, N*H*), 4.87 (td, *J* = 11.4, 5.4 Hz, 1H, HNC*H*), 4.73 (d, *J* = 7.9 Hz, 1H, O*H*), 3.49 (dd, *J* = 9.1, 5.5 Hz, 1H, PhCCC*H*), 3.35 (qd, *J* = 8.9, 2.4 Hz, 1H, HOC*H*), 1.94 – 1.81 (m, 1H, CH_2), 1.71 – 1.50 (m, 2H, CH_2 x2), 1.40 – 1.17 (m, 7H, CH_2 x 7), 0.87 – 0.77 (m, 3H, CH_3).

^{13}C NMR (101 MHz, Acetone-*d*$_6$) δ ppm 142.4 (d, *J* = 6.3 Hz, Ar*C*), 135.0 (d, *J* = 126.7 Hz, Ar*C*), 134.2 (d, *J* = 130.9 Hz, Ar*C*), 133.5 (d, *J* = 9.5 Hz, Ar*C*H x 2), 132.8 (d, *J* = 9.4 Hz, Ar*C*H x 2), 132.6 (d, *J* = 2.8 Hz, Ar*C*H), 132.5 (d, *J* = 2.9 Hz, Ar*C*H x 2), 132.4 (Ar*C*H x 2), 129.3 (d, *J* = 11.2 Hz, Ar*C*H x 2), 129.3 (Ar*C*H x 2), 129.2 (d, *J* = 12.8 Hz, Ar*C*H x 2), 129.2 (Ar*C*H x 2), 128.9 (Ar*C*H), 128.5 (Ar*C*H x 2), 128.0 (Ar*C*H), 124.7 (Ar*C*), 89.4 (Ph*C*≡C), 86.8 (PhC≡*C*), 72.4 (HO*C*H), 55.9 (NH*C*H), 50.5 (d, *J* = 2.9 Hz, PhCC*C*H), 36.7 (CH(OH)*C*H_2), 32.8 (CH(OH)CH_2*C*H_2), 26.5 (CH(OH)CH_2CH_2*C*H_2), 25.5 (CH(OH)$CH_2CH_2CH_2$*C*H_2), 23.4 (CH(OH)$CH_2CH_2CH_2CH_2$*C*H_2), 14.5 (*C*H_3).

^{31}P NMR (162 MHz, Acetone-*d*$_6$) δ ppm 21.57.

IR ν_{max} (neat/cm^{-1}): 3355, 3058, 2954, 1597, 1454, 1438, 11862, 1123, 1067, 912.

HRMS : calcd for $C_{35}H_{39}NO_2P$ [M + H]$^+$: 536.2705, found 536.2705.

Specific rotation: $[\alpha]_D^{23}$ + 30.5 (c 10.86, $CDCl_3$)

Enantiomeric purity of **6c** was determined by HPLC analysis in comparison with authentic racemic material (ee >99%; **OD-H** column, 98:2 hexanes: *i*-PrOH, 1.0 mL/min, 20 °C, 254 nm)

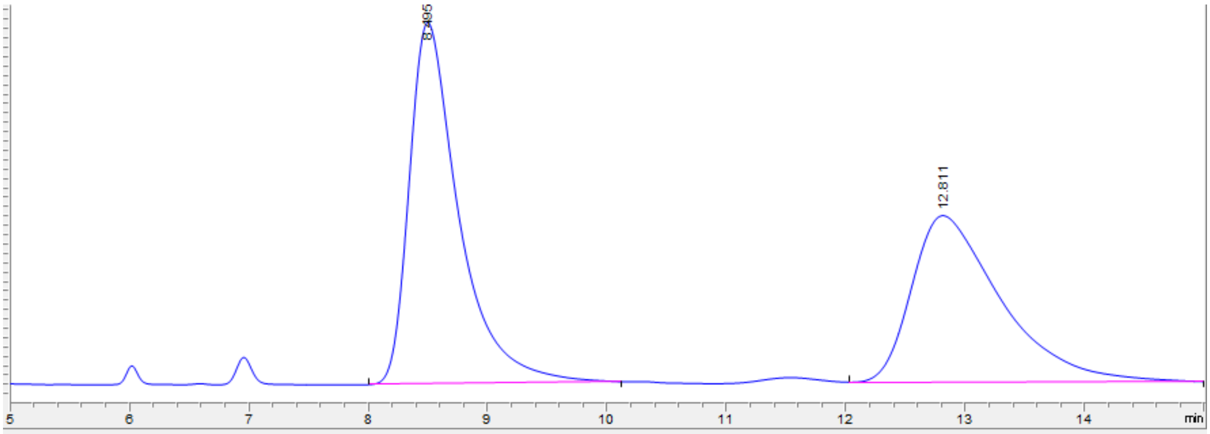

#	Time	Type	Area	Height	Width	Area%	Symmetry
1	8.495	BB	2229.2	77	0.4279	54.347	0.546
2	12.811	VB	1872.6	35.7	0.7944	45.653	0.502

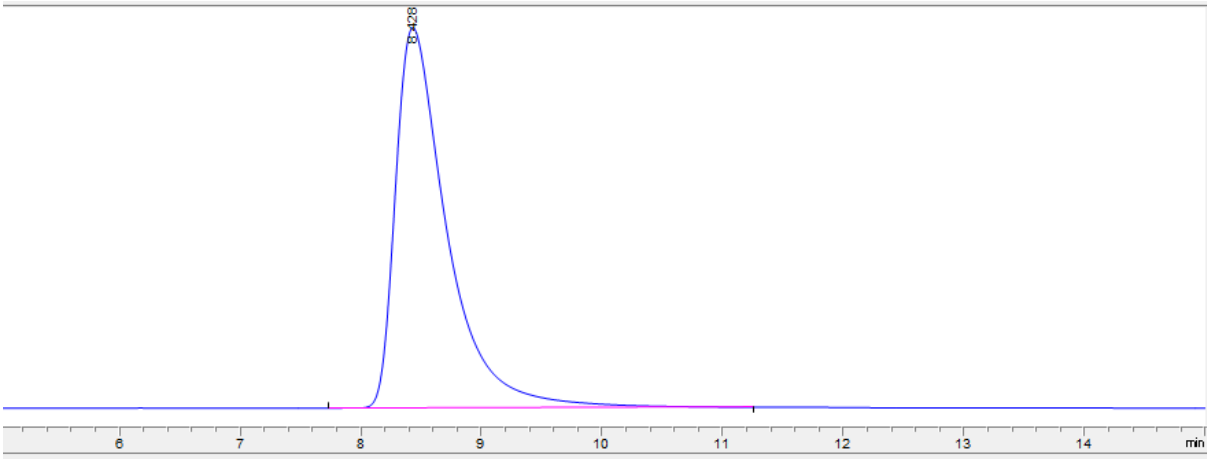

#	Time	Type	Area	Height	Width	Area%	Symmetry
1	8.428	BB	16288.5	545.3	0.4431	100.000	0.458

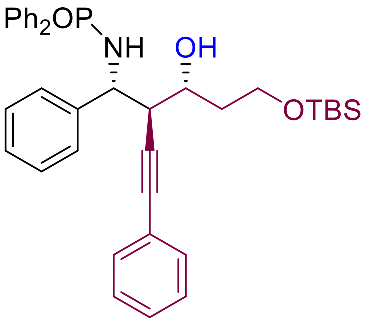

***N*-((1*R*,2*S*,3*R*)-5-((*tert*-Butyldimethylsilyl)oxy)-3-hydroxy-1-phenyl-2-(phenylethynyl)pentyl)-*P*,*P*-diphenylphosphinic amide (6d)**

Compound **6d** was prepared according to general procedure B using (*E*)-enyne (0.192 mmol scale). The title compound was isolated by column chromatography (hexane: ethyl acetate, 60:40-20:80) as a white amorphous solid (74 mg, 0.121 mmol, 63%). M. p.: 71-72 ˚C ($CHCl_3$).

^{1}H NMR (500 MHz, $CDCl_3$) δ ppm 7.85 (dd, *J* = 12.0, 7.9 Hz, 2H, ArC*H* x 2), 7.73 (dd, *J* = 12.2, 7.9 Hz, 2H, ArC*H* x 2), 7.50 – 7.42 (m, 2H, ArC*H* x 2), 7.43 – 7.38 (m, 4H, ArC*H* x 4), 7.34 – 7.23 (m, 10H, ArC*H* x 10), 4.80 (td, *J* = 11.4, 2.7 Hz, 1H, C*H*NH), 4.11 (dd, *J* = 11.6, 6.3 Hz, 1H, NH), 3.95 – 3.85 (m, 2H, C*H*2OTBS x 1 + O*H*), 3.75 (ddt, *J* = 10.7, 8.7, 3.6 Hz, 1H, C*H*2OTBS x 1), 3.41 (br s, 2H CC-C*H* x 1 + C-*H*(OH) x 1), 2.0 (t, *J* = 4.5 Hz, 1H, (CH_2OH)C*H*2 x 1), 1.81 – 1.72 (m, 1H, (CH_2OHC*H*2 x1), 0.86 (s, 9H, Si-*t*Bu), 0.05 (s, 3H, Si-C*H*3) , 0.03 (s, 3H, Si-C*H*3).

^{13}C NMR (126 MHz, $CDCl_3$) δ ppm 140.2 (d, *J* = 6.2 Hz, Ar*C*), 133.1 (d, *J* = 126.5 Hz, Ar*C*), 132.7 (d, *J* = 10.0 Hz, Ar*C*H x 2), 131.8 (d, *J* = 2.8 Hz, Ar*C*H), 131.7 (d, *J* = 9.5 Hz, Ar*C*H x 2), 131.7 (d, *J* = 2.8 Hz, Ar*C*H), 131.6 (d, *J* = 132.1 Hz, Ar*C*), 131.6 (Ar*C*H x 2), 128.5 (d, *J* = 12.5 Hz, Ar*C*H x 2), 128.3 (d, *J* = 10.1 Hz Ar*C*H x 2), 128.2 (Ar*C*H x 2), 128.1 (Ar*C*H), 127.9 (Ar*C*H x 2), 127.7 (Ar*C*H x 2), 127.3 (Ar*C*H), 123.0 (Ar*C*), 86.8 (Ph*C*≡C), 86.7 (PhC≡*C*), 72.03 (*C*OH), 62.55 (*C*H2OTBS), 53.77 (NH*C*H), 48.64 (d, *J* = 2.8 Hz, PhCC*C*H), 36.33 (C(OH)-*C*H2), 25.84 (Si-C*(C*H3)3 x3), 18.10 (Si-*C(*CH3)3), -5.48 (Si-*C*H3), -5.61 (Si-*C*H3).

^{31}P NMR (202 MHz, $CDCl_3$) δ ppm 23.61.

IR ν_{max} (neat/cm^{-1}): 3261, 3060, 2952, 2926, 2855, 1598, 1490, 1438, 1470, 1412, 1253, 1190, 1123, 1109, 1086, 909, 776, 755, 725, 696.

HRMS : calcd for $C_{37}H_{44}NO_3PSiNa1$ [M + Na]$^+$: 632.2720, found 632.2694.

Specific rotation: $[\alpha]_D^{29.3}$ +28.6 (c 0.59, $CHCl_3$).

Enantiomeric purity of **6d** was determined by HPLC analysis in comparison with authentic racemic material (ee >99%; **OD-H** column, 95:5 hexanes: *i*-PrOH, 1 mL/min, 22 °C, 254 nm)

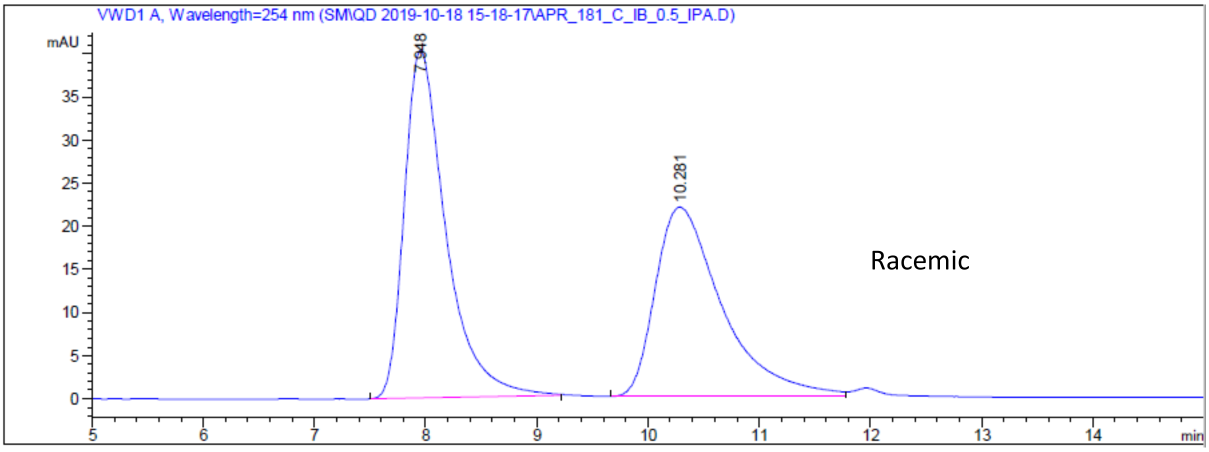

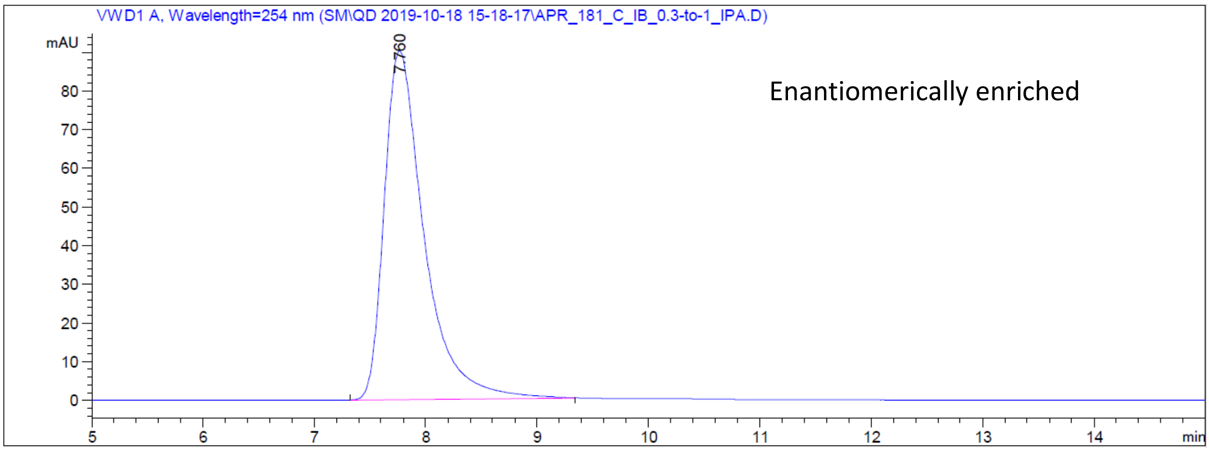

Signal 1: VWD1 A, Wavelength=254 nm

Peak #	RetTime [min]	Type	Width [min]	Area [mAU*s]	Height [mAU]	Area %
1	7.760	BB	0.3740	2249.74023	90.20699	100.0000

Product derivatization

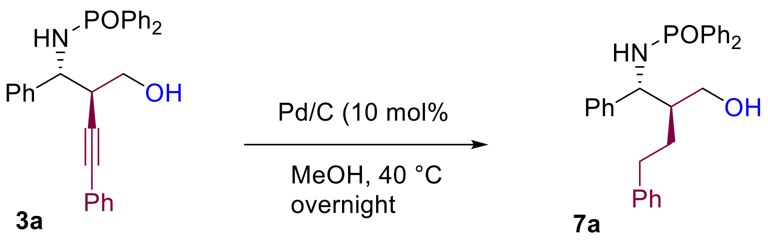

***N*-((1*R*,2*S*)-2-(Hydroxymethyl)-1,4-diphenylbutyl)-*P*,*P*-diphenylphosphinic amide (7a)**

To a solution of **3a** (41.5 mg, 0.1 mmol) in MeOH (2.0 mL) was added 10 % palladium on carbon (10.6 mg, 10% by weight), the mixture was then stirred under a hydrogen gas atmosphere (balloon) for 24 h at 40 ˚C. [1] After completion, the crude reaction mixture was filtered through celite topped with a layer of sand. The solution was concentrated in vacuo. Column chromatography (hexane/EtOAc = 60:40-20:80) afforded the title compound as a colourless solid (43.3 mg, 0.095 mmol, 95 % yield). M.p.: 124-129 ˚C ($CHCl_3$).

^{1}H NMR (400 MHz, $CDCl_3$) δ ppm 7.77 – 7.68 (m, 2H, ArC*H* x 2), 7.64 – 7.55 (m, 2H, ArC*H* x 2), 7.52 – 7.43 (m, 2H, ArC*H* x 2), 7.42 – 7.34 (m, 2H, ArC*H* x 2), 7.33 – 7.27 (m, 2H, ArC*H* x 2), 7.27 – 7.22 (m, 3H, ArC*H* x 3), 7.17 – 7.05 (m, 3H, ArC*H* x 3), 6.94 – 6.84 (m, 4H, ArC*H* x 4), 5.10 (dd, *J* = 8.4, 5.8 Hz, 1H, O*H*), 4.38 – 4.20 (m, 1H, HOCH_2), 4.13 – 3.94 (m, 1H, HOCH_2), 3.87 – 3.73 (m, 1H, NHC*H*), 3.39 (t, *J* = 11.1 Hz, 1H, N*H*), 2.77 – 2.59 (m, 1H, PhCH_2), 2.46 – 2.36 (m, 1H, PhCH_2), 1.77 (dq, *J* = 8.9, 4.4 Hz, 1H, C*H*), 1.63 (ddd, *J* = 9.9, 7.3, 3.6 Hz, 1H, PhCH$_2$CH_2), 1.33 – 1.14 (m, 1H, PhCH$_2$CH_2).

^{13}C NMR (101 MHz, $CDCl_3$) δ ppm 143.2 (d, *J* = 5.3 Hz, Ar*C*), 142.0 (Ar*C*), 132.5 (d, *J* = 126.2 Hz, Ar*C*), 133.3 (d, *J* = 10.2 Hz, Ar*C*H x 2), 132.3 (d, *J* = 2.8 Hz, Ar*C*H), 132.2 (d, *J* = 2.9 Hz, Ar*C*H), 131.3 (d, *J* = 9.8 Hz, Ar*C*H x 2), 130.8 (d, *J* = 133.4 Hz, Ar*C*), 128.8 (d, *J* = 12.6 Hz, ArCH x 2), 128.6 (ArCH x 2), 128.5 (ArCH x 2), 128.4 (d, *J* = 13.0 Hz, Ar*C*H x 2), 128.2 (Ar*C*H x 2), 127.4 (Ar*C*H), 127.2 (Ar*C*H x 2), 125.6 (Ar*C*H), 59.3 (HO*C*H$_2$), 56.7 (CH$_2$CH$_2$*C*H), 47.0 (d, *J* = 2.1 Hz, HN*C*H), 33.4 (Ph*C*H$_2$), 28.9 (PhCH$_2$*C*H$_2$).

^{31}P NMR (162 MHz, $CDCl_3$) δ ppm 26.1.

IR ν_{max} (neat/cm^{-1}): 3368, 3060, 2938, 1453, 1371, 1198, 1153, 1057, 977.

HRMS : calcd for $C_{29}H_{30}NO_2PNa$ $[M + Na]^+$ 478.1906, found 478.1889.

Specific rotation: $[\alpha]_D^{30.1}$ - 51.25 (c 0.48, $CHCl_3$).

Enantiomeric purity of **7a** was determined by HPLC analysis in comparison with authentic racemic material (ee >98%; **OD-H** column, 96:4 hexanes: *i*-PrOH, 1 mL/min, 20 °C, 254 nm)

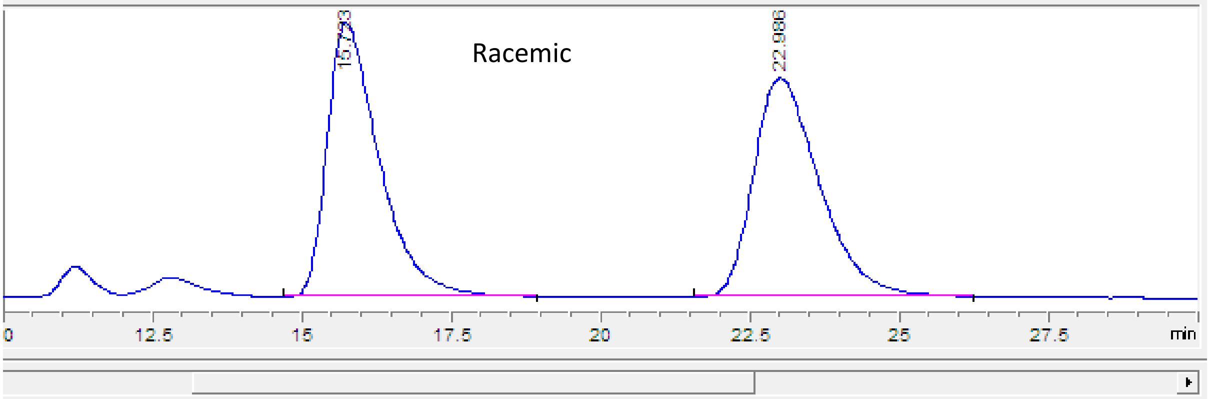

#	Time	Area	Height	Width	Area%	Symmetry
1	15.723	11569.2	189.8	0.8791	49.834	0.528
2	22.986	11646.2	151.6	1.1182	50.166	0.632

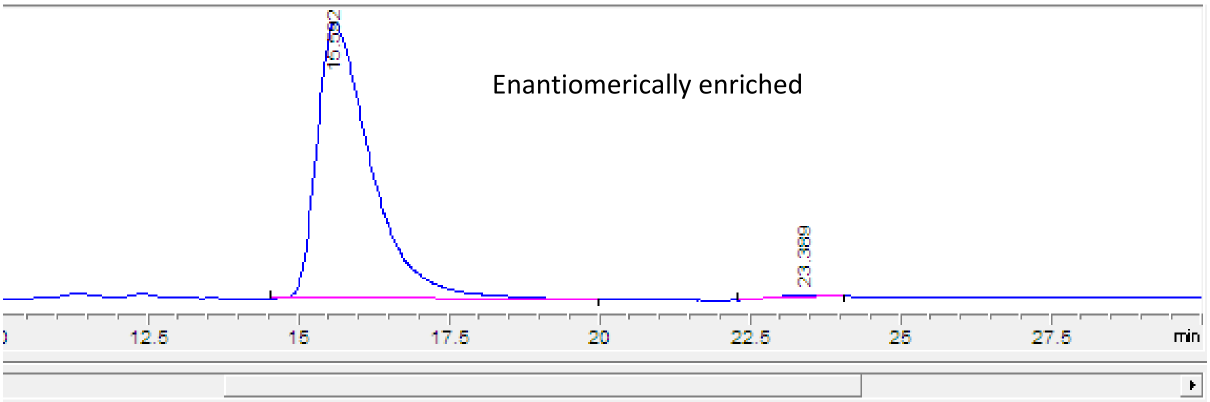

#	Time	Area	Height	Width	Area%	Symmetry
1	15.592	1874.8	29.9	0.9628	98.999	0.454
2	23.389	19	3.5E-1	0.6435	1.001	1.752

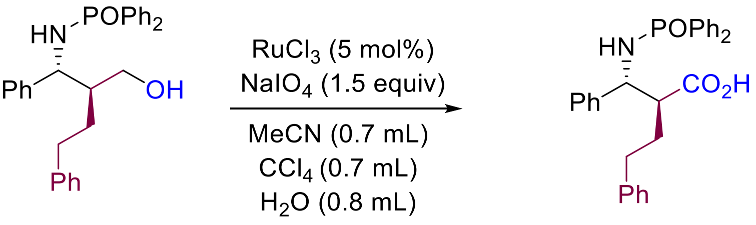

7a **7b**

(*S*)-2-((*R*)-((Diphenylphosphoryl)amino)(phenyl)methyl)-4-phenylbutanoic acid (7b)

To a solution of **7b** (41.5 mg, 0.1 mmol) in CH_3CN (0.7 mL), CCl_4 (0.7 mL) and H_2O (0.8 mL) was added $RuCl_3$ (1.0 mg, 5 mol%) and $NaIO_4$ (32.1 mg, 1.5 equiv), and the reaction stirred for 4 h at room temperature.[2] After completion of the reaction, it was quenched with water (10 mL)

and extracted with EtOAc (3 × 20 mL). The combined organic layer was concentrated in vacuo. The title compound was purified by column chromatography (dichloromethane: methanol, 99:1-95:5) to afforded the title compound as white solid (42.7 mg, 0.091 mmol, 91 % yield). M.p.: 131-136 ˚C ($CHCl_3$).

^{1}H NMR (400 MHz, CD_3OD) δ ppm 7.82 (dd, *J* = 12.1, 7.6 Hz, 2H, Ar*C*H x 2), 7.70 – 7.62 (m, 2H, Ar*C*H x 2), 7.51 (t, *J* = 7.4 Hz, 1H, Ar*C*H), 7.48 – 7.36 (m, 3H, Ar*C*H x 3), 7.25 (td, *J* = 7.6, 2.5 Hz, 2H, Ar*C*H x 2), 7.19 – 7.12 (m, 3H Ar*C*H x 3), 7.11 – 7.05 (m, 3H, Ar*C*H x 3), 7.02 (d, *J* = 7.1 Hz, 2H, Ar*C*H x 2), 4.23 (t, *J* = 10.5 Hz, 1H, NHC*H*), 2. 77 – 2.63 (m, 1H, PhCCC*H*), 2.63 (t, *J* = 12.5 Hz, 1H, PhCH_2), 2.38 – 2.34 (m, 1H, PhCH_2), 1.72 (d, *J* = 11.6 Hz, 1H, PhCH_2CH_2), 1.37 – 1.04 (m, 1H, PhCH_2CH_2).

^{13}C NMR (101 MHz, CD_3OD) δ ppm 182.2 (CO_2H), 144.0 (d, *J* = 2.5 Hz, Ar*C*), 143.1 (Ar*C*), 134.4 (d, *J* = 132.0 Hz, Ar*C*), 134.2 (d, *J* = 10.0 Hz, ArCH x 2), 132.6 (d, *J* = 134.6 Hz, Ar*C*), 129.6 (d, *J* = 12.7 Hz, Ar*C*H x 2), 129.3 (Ar*C*H x 2), 129.3 (Ar*C*H x 3), 129.3 (Ar*C*H), 129.2 (Ar*C*H), 129.2 (Ar*C*H x 2), 129.1 (Ar*C*H), 128.4 (Ar*C*H x 2), 128.2 (Ar*C*H), 126.7 (Ar*C*H), 79.5 (NH*C*H), 59.6 (HO_2CCH), 34.9 (PhCH_2), 33.8 (PhCH_2CH_2).

IR ν_{max} (neat/cm^{-1}): 3058, 3029, 2922, 1693, 1437, 1413, 1179, 1123, 1070, 923.

^{31}P NMR (162 MHz, CD_3OD) δ ppm 24.2.

HRMS : calcd for $C_{29}H_{28}NO_3PNa$ [M + Na]$^+$ 492.1699, found 492.1681.

Specific rotation: $[\alpha]_D^{26.5}$ - 12.8 (c 0.33, $CHCl_3$).

Enantiomeric purity of **7b** was determined by HPLC analysis in comparison with authentic racemic material (ee >99%; **OD-H** column, 97:3 hexanes: *i*-PrOH, 1 mL/min, 20 °C, 220 nm)

(*ee was measured after preparation of methyl ester of product **7b** with trimethylsilyl diazomethane*)

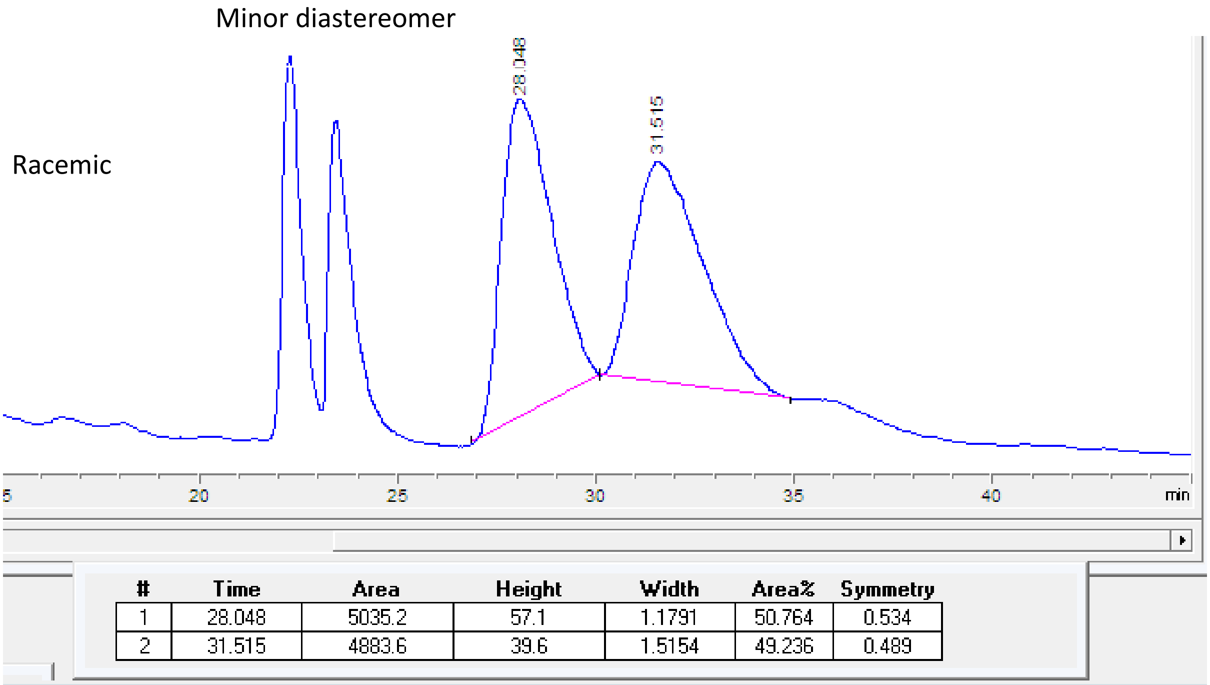

#	Time	Area	Height	Width	Area%	Symmetry
1	28.048	5035.2	57.1	1.1791	50.764	0.534
2	31.515	4883.6	39.6	1.5154	49.236	0.489

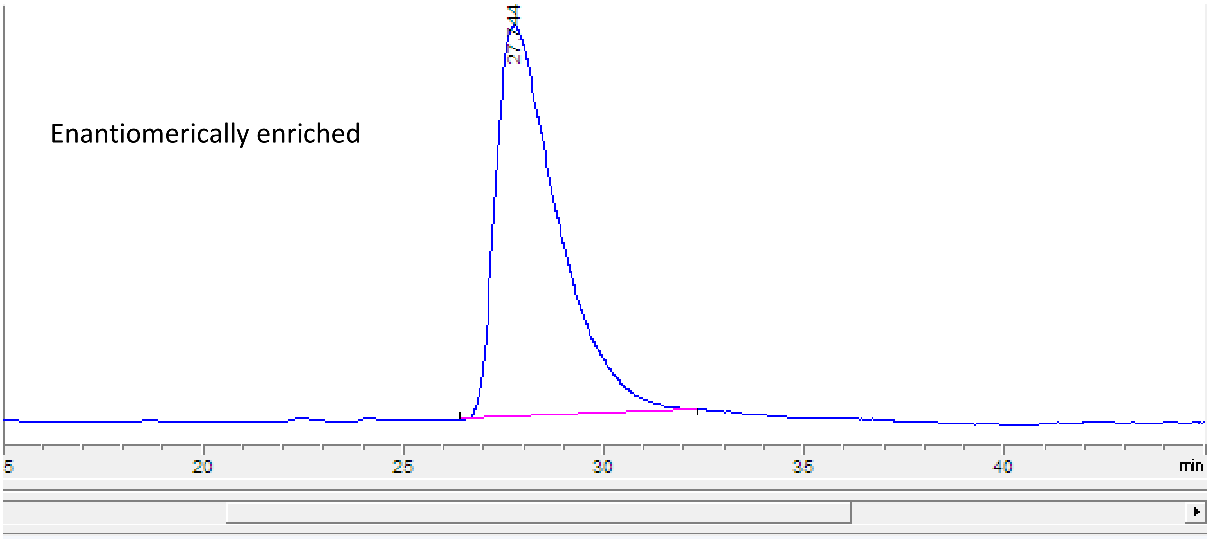

#	Time	Area	Height	Width	Area%	Symmetry
1	27.744	10872.8	101.1	1.4567	100.000	0.403

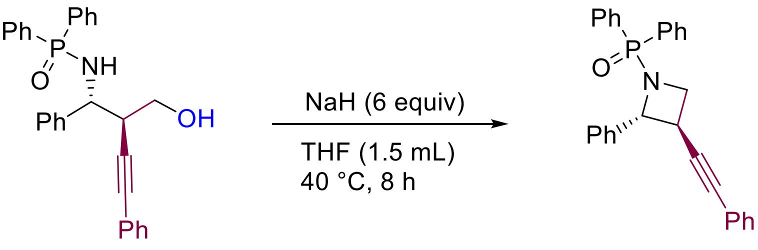

3a **7c**

Diphenyl((2*R*,3*R*)-2-phenyl-3-(phenylethynyl)azetidin-1-yl)phosphine oxide (7c)

To a solution of **7c** (41.5 mg, 0.1 mmol), 4-toluenesulfonyl chloride (28.5 mg, 0.15 mmol) in dry THF (1.5 mL) was added sodium hydride (22.7 mg, 6 equiv). The reaction mixture was stirred for 8 h at 40 ˚C under nitrogen atmosphere.[3] After completion of the reaction, it was quenched with water (10 mL) and extracted with EtOAc (3 × 20 mL). The combined organic layer was concentrated in vacuo. The title compound was purified by column chromatography (hexane/EtOAc = 70:30 -50:50) afforded the title compound as a colourless solid (41.1 mg, 95%). M.p.: 128-132 ˚C ($CHCl_3$).

^{1}H NMR (400 MHz, $CDCl_3$) δ ppm 8.00 – 7.90 (m, 2H, ArCH x 2), 7.57 – 7.42 (m, 5H, ArCH x 5), 7.39 – 7.31 (m, 2H, ArCH x 2), 7.29 – 7.22 (m, 3H, ArC*H* x 3), 7.25 – 7.12 (m, 3H, ArC*H* x 3), 7.15 – 7.08 (m, 3H, ArC*H* x 3), 7.07 – 6.97 (m, 2H, ArC*H* x 2), 5.19 (dd, *J* = 12.3, 7.5 Hz, 1H, C*H*N), 4.30 – 4.03 (m, 1H, NC*H*$_2$), 3.84 – 3.75 (m, 1H, NC*H*$_2$), 3.71 – 3.60 (m, 1H, PhCCC*H*).

^{13}C NMR (101 MHz, $CDCl_3$) δ ppm 140.6 (d, *J* = 1.2 Hz, Ar*C*), 132.5 (d, *J* = 9.0 Hz, Ar*C*H x 2), 132.4 (d, *J* = 9.6 Hz, Ar*C*H x 2), 132.2 (d, *J* = 2.8 Hz, Ar*C*H), 131.8 (Ar*C*H x 2), 131.6 (d, *J* = 2.9 Hz, Ar*C*H), 130.8 (d, *J* = 128.1 Hz, Ar*C*), 129.7 (d, *J* = 126.1 Hz, Ar*C*), 128.8 (d, *J* = 12.3 Hz, ArCH x 2), 128.3 (Ar*C*H x 2), 128.3 (Ar*C*H), 128.2 (Ar*C*H x 2), 128.3 (Ar*C*H), 127.8 (d, *J* = 12.6 Hz, ArCH x 2), 127.3 (Ar*C*H x 2), 123.1 (Ar*C*), 87.5 (Ph*C*≡C), 85.4 (PhC≡*C*). 68.44 (d, *J* = 3.8 Hz, Ph*C*HN), 50.06 (d, *J* = 3.7 Hz, N*C*H$_2$), 32.97 (d, *J* = 22.7 Hz, PhC≡*C*H).

^{31}P NMR (162 MHz, $CDCl_3$) δ ppm 26.00.

IR ν_{max} (neat/cm^{-1}): 3056, 2959, 2880, 1490, 1438, 1366, 1198, 1109, 1013, 996.

HRMS : calcd for $C_{29}H_{23}NOP$ [M + H]$^+$ 432.1523, found 432.1525.

Specific rotation: $[\alpha]_D^{26.5}$ - 47.8 (c 0.8, CH_2Cl_2).

Enantiomeric purity of **7c** was determined by HPLC analysis in comparison with authentic racemic material (ee: 95%; **OD-H** column, 97:3 hexanes: *i*-PrOH, 1 mL/min, 20 °C, 254 nm).

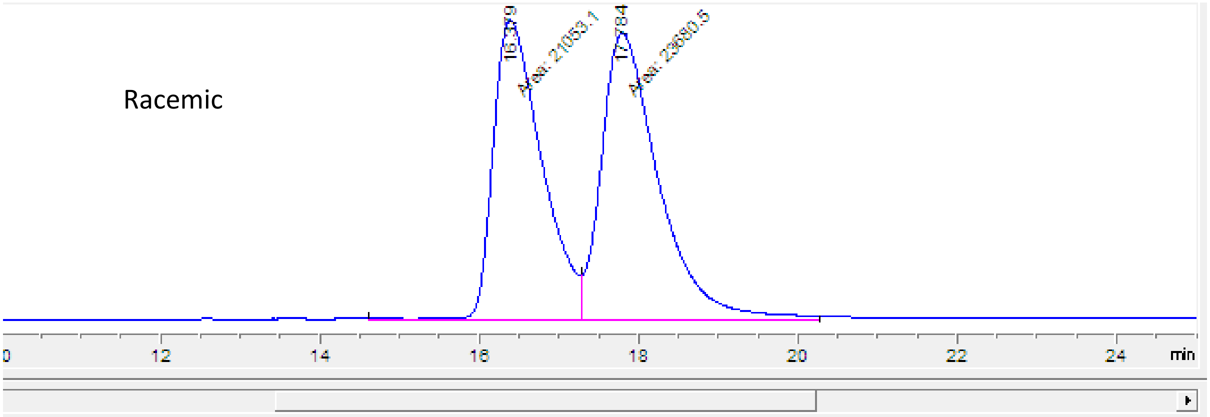

#	Time	Area	Height	Width	Area%	Symmetry
1	16.379	21053.1	494.1	0.7101	47.063	0
2	17.784	23680.5	472.5	0.8352	52.937	0.537

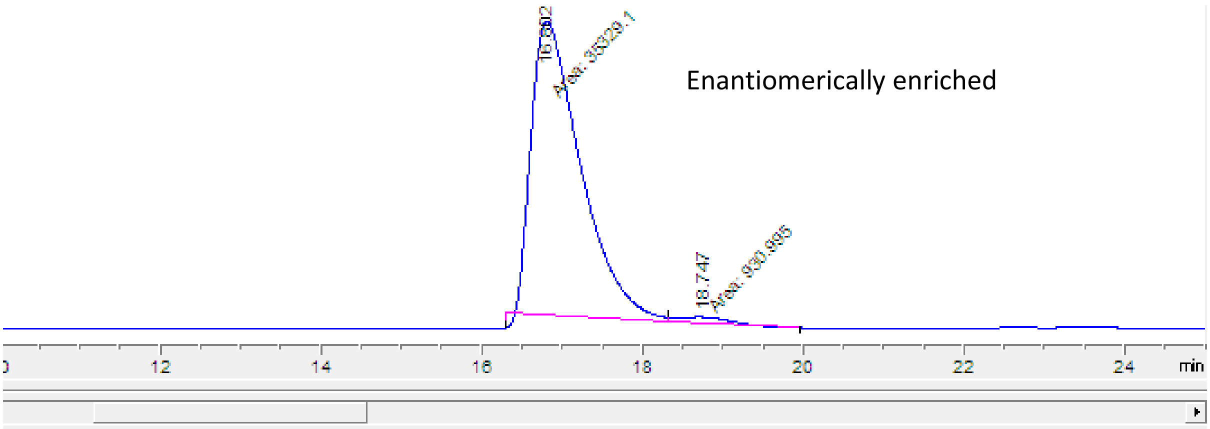

#	Time	Area	Height	Width	Area%	Symmetry
1	16.802	35329.1	822.2	0.7161	97.432	0.454
2	18.747	931	19.1	0.8131	2.568	0.576

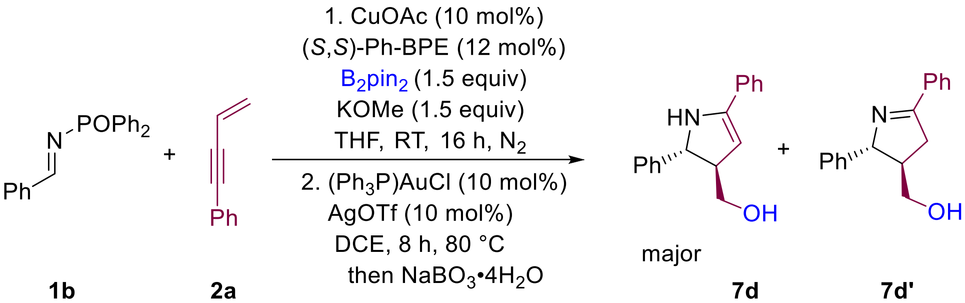

1b **2a** **7d** **7d'**

((2*R*,3*R*)-2,5-Diphenyl-2,3-dihydro-1*H*-pyrrol-3-yl)methanol and **((2*R*,3*R*)-2,5-diphenyl-2,3-dihydro-1*H*-pyrrol-3-yl)methanol** (**7d** and **7d'**)

After completion of first step reaction, the boron-containing crude mixture was dissolved in dry DCE (2 mL). Then, $(Ph_3P)AuCl$ (10 mol%, 9.9 mg) and AgOTf (10 mol%, 5.2 mg) were added to the solution under nitrogen. The resulting mixture was then stirred at 80 °C for 8 h.[4] The

reaction mixture was allowed to room temperature and the solvent was evaporated in vacuo. In the next step, THF (2.0 mL), H_2O (2.0 mL) and $NaBO_3.4H_2O$ (1.2 mmol, 180 mg) were added and the reaction mixture stirred at room temperature for 6 h. The reaction was then quenched by the addition of water (10 mL). The aqueous layer was extracted with EtOAc (3 x 10 mL), and the combined organic layers were washed with brine (1 x 10 mL), dried over $MgSO_4$, and concentrated in vacuo. Column chromatography (hexane: EtOAc, 20:80-30:70) afforded the mixture of compounds (4:1) as a colourless solid (33.2 mg, 0,132 mmol, 66%).

^{1}H NMR (500 MHz,$CDCl_3$) δ ppm 7.94 (dd, *J* = 7.9, 1.7 Hz, 2H, ArC*H*, major), 7.92 – 7.86 (m, 1H, ArC*H* minor), 7.49 – 7.36 (m, 3H, ArC*H*, major and minor), 7.34 – 7.28 (m, 2H, ArC*H* x 2), 7.27 – 7.17 (m, 4H, ArC*H* x 4), 5.5 (d, *J* = 8.0 Hz, 1H, pyrrole-C*H*, major), 5.1 (d, *J* = 5.7 Hz, 1H, pyrrole-C*H, minor*), 3.81 (dd, *J* = 10.5, 6.0 Hz, 1H, HOCH_2, minor), 3.72 (dd, *J* = 10.5, 6.9 Hz, 1H, HOCH_2, minor), 3.31 – 3.23 (m, 1H, PhC*H*NH, major and minor), 3.21 – 3.12 (m, 2H, O*H* and HO-CH_2, major and minor), 3.07 – 2.86 (m, 2H, O*H* and HOCH$_2$-C*H,* major and minor), 2.57 – 2.43 (m, 1H, HOCH$_2$-C*H,* minor).

^{13}C NMR (101 MHz, $CDCl_3$) δ 173.9 (N=*C*), 143.8 (Ar*C*H), 139.5(Ar*C*H), 134.4 (Ar*C*H), 131.0 (Ar*C*H), 130.9 (Ar*C*H), 128.7 (Ar*C*H), 128.7 (Ar*C*H), 128.0 (Ar*C*H), 127.5 (Ar*C*H), 127.2 (Ar*C*), 126.8 (Ar*C*), 78.6 (pyrrole-*C*H), 76.9 (pyrrole-*C*H), 65.0 (HO*C*H$_2$), 63.4(HO*C*H$_2$), 44.0 (HOCH$_2$C*H*), 38.9 (HO*C*H$_2$).

IR ν_{max} (neat/cm^{-1}): 3368, 3060, 2938, 1453, 1371, 1198, 1153, 1057, 977.

HRMS : calcd for $C_{17}H_{18}NO$ $[M + H]^+$ 252.1383, found 252.1384.

Enantiomeric purity of **7d** and **7d'** was determined by HPLC analysis in comparison with authentic racemic material (ee: 93% major, 52% minor; **OD-H** column, 93:7 hexanes: *i*-PrOH, 0.7 mL/min, 20 °C, 254 nm)

(*The minor peak was confirmed by using minor product after recrystallization of mixture of products.)*

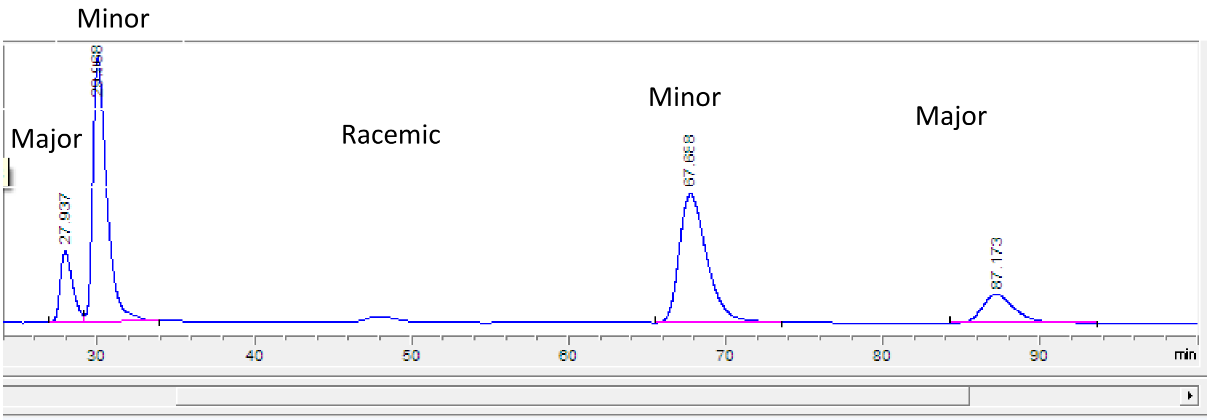

#	Time	Area	Height	Width	Area%	Symmetry
1	27.937	4762.5	87.9	0.8159	9.774	0.622
2	29.968	19878.5	326.2	0.925	40.798	0.502
3	67.688	18946.1	159.7	1.8138	38.884	0.641
4	87.173	5137.6	35	2.0008	10.544	0.756

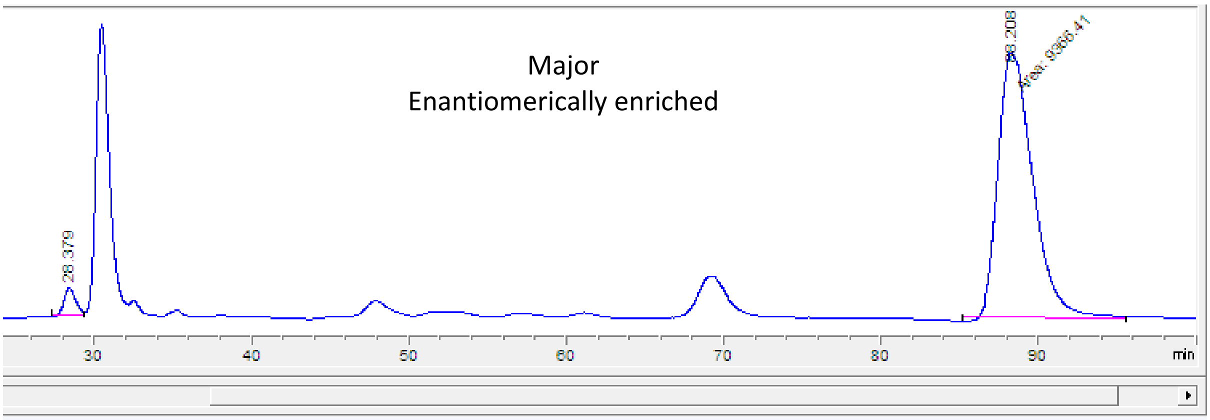

#	Time	Area	Height	Width	Area%	Symmetry
1	28.379	348.9	6.5	0.7647	3.591	0.734
2	88.208	9366.4	61.2	2.5508	96.409	0.564

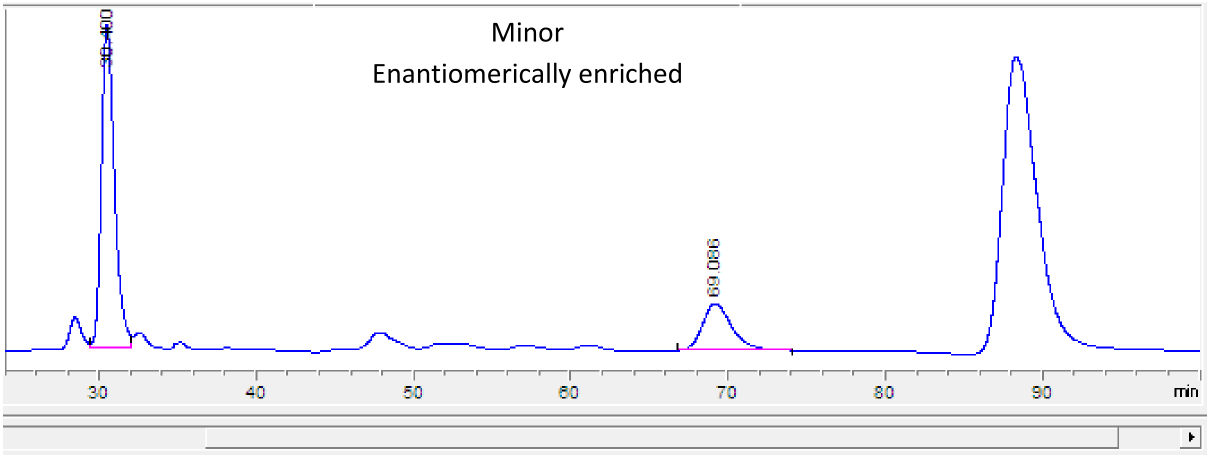

#	Time	Area	Height	Width	Area%	Symmetry
1	30.4	3942.1	67.3	0.8998	75.916	0.675
2	69.086	1250.6	9.6	1.5563	24.084	0.698

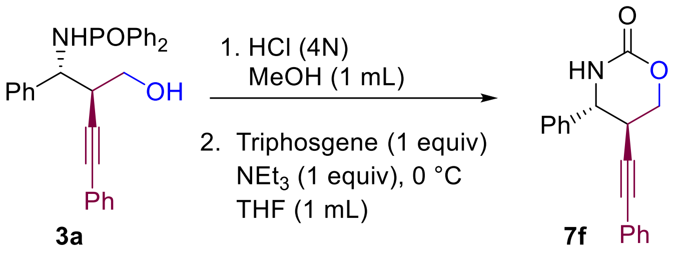

(4*R*,5*S*)-4-Phenyl-5-(phenylethynyl)-1,3-oxazinan-2-one (7f)

To a solution of **3a** (41.5 mg, 0.1 mmol) in MeOH (1 mL) was added slowly 4 N HCl (1 mL). The reaction mixture was stirred for 3 h at room temperature. After completing the reaction, 4 N NaOH (2 mL) was added slowly and the reaction stirred for 10 min.[5] The aqueous layer was extracted with EtOAc (3 x 5 mL), and the combined organic layers were washed with brine (1 x 10 mL), dried over $MgSO_4$, and concentrated in vacuo.

In the next step, the crude mixture was dissolved in dry THF (1.0 mL), and cooled to 0 °C, followed by the addition of triphosgene (29.6 mg, 0.1 mmol) and stirring for 10 min before the addition of triethylamine (14 µL, 0.1 mmol).[2] The resulting mixture was stirred at 0 °C for 3 h.[2] The mixture was allowed to warm room temperature before quenching by addition of water (2 mL). The aqueous layer was extracted with EtOAc (3 x 5 mL), and the combined organic layers were washed with brine (1 x 10 mL), dried over $MgSO_4$, and concentrated in vacuo. Column chromatography (hexane: EtOAc) afforded the title compound as a colourless solid (18.3 mg, 0.066 mmol, 66%). M.p.: 142-147 ˚C ($CHCl_3$).

^{1}H NMR (400 MHz, $CDCl_3$) δ ppm 7.45 – 7.36 (m, 5H, ArC*H* x 5), 7.35 – 7.27 (m, 5H, ArC*H* x 5), 5.51 (s, 1H, N*H*), 4.61 (d, *J* = 9.2 Hz, 1H, HNC*H*), 4.48 (dd, *J* = 11.1, 4.0 Hz, 1H, OCH_2), 4.34 (t, *J* = 10.6 Hz, 1H, OCH_2), 3.12 (td, *J* = 9.7, 4.0 Hz, 1H, PhCCC*H*).

^{13}C NMR (101 MHz, $CDCl_3$) δ ppm 153.1 (NH*C*O), 139.1 (Ar*C*), 131.8 (Ar*C*H x 2), 129.2 (Ar*C*H), 129.1 (Ar*C*H x 2), 128.8 (Ar*C*H), 128.5 (Ar*C*H x 2), 126.8 (Ar*C*H), 122.2 (Ar*C*), 85.9 (Ph*C*≡C), 83.0 (PhC≡*C*), 68.3 (HO*C*H_2), 60.9 (NH*C*H), 34.6 (PhC≡*C*CH).

IR ν_{max} (neat/cm^{-1}): 3269, 3141, 2901, 1719, 1682, 1469, 1366, 1279, 1160, 1028.

HRMS : calcd for $C_{18}H_{16}NO_2$ $[M + H]^+$ 278.1176, found 278.1173.

Specific rotation: $[\alpha]_D^{30.4}$- 11.5 (c .33, $CHCl_3$).

Enantiomeric purity of **7f** was determined by HPLC analysis in comparison with authentic racemic material (ee: 92%; **OD-H** column, 97:3 hexanes: *i*-PrOH, 1 mL/min, 20 °C, 254 nm)

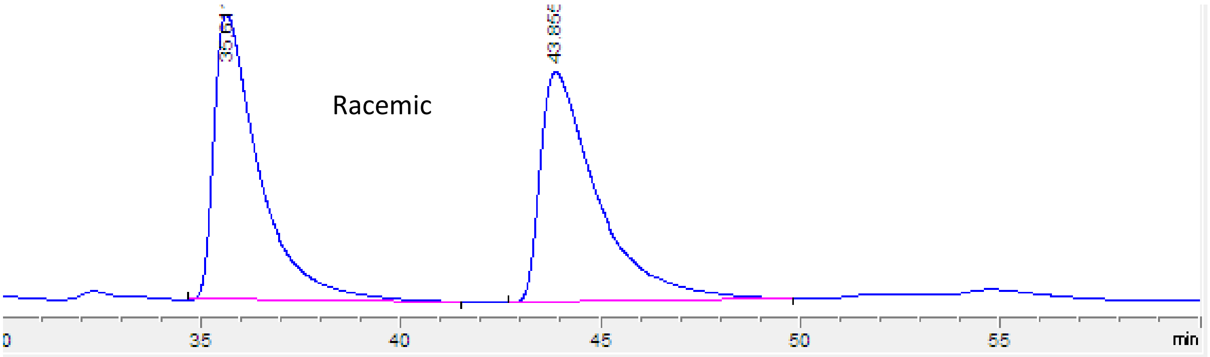

#	Time	Area	Height	Width	Area%	Symmetry
1	35.611	5935.7	76	1.1087	49.491	0.347
2	43.855	6057.8	61.2	1.344	50.509	0.343

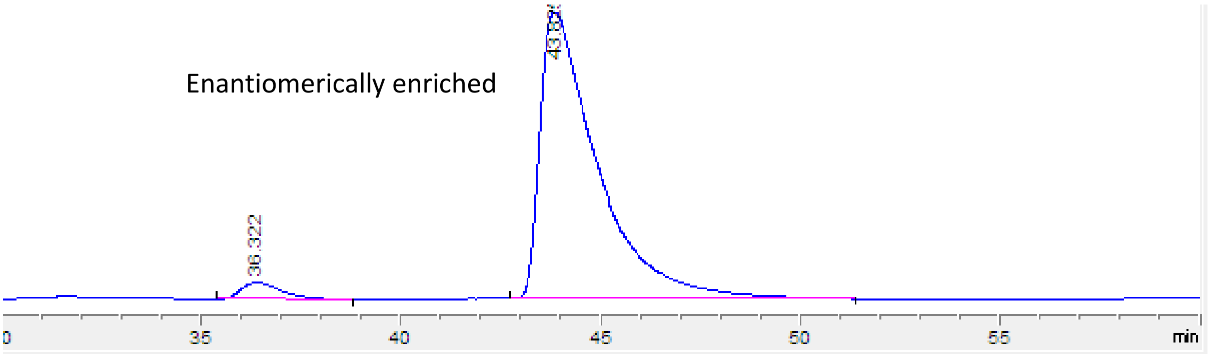

#	Time	Area	Height	Width	Area%	Symmetry
1	36.322	503.7	7	0.8557	4.118	0.518
2	43.825	11729.8	117.9	1.3885	95.882	0.308

NMR spectra of synthesized compounds

^{1}H NMR of **3a** (400 MHz, $CDCl_3$)

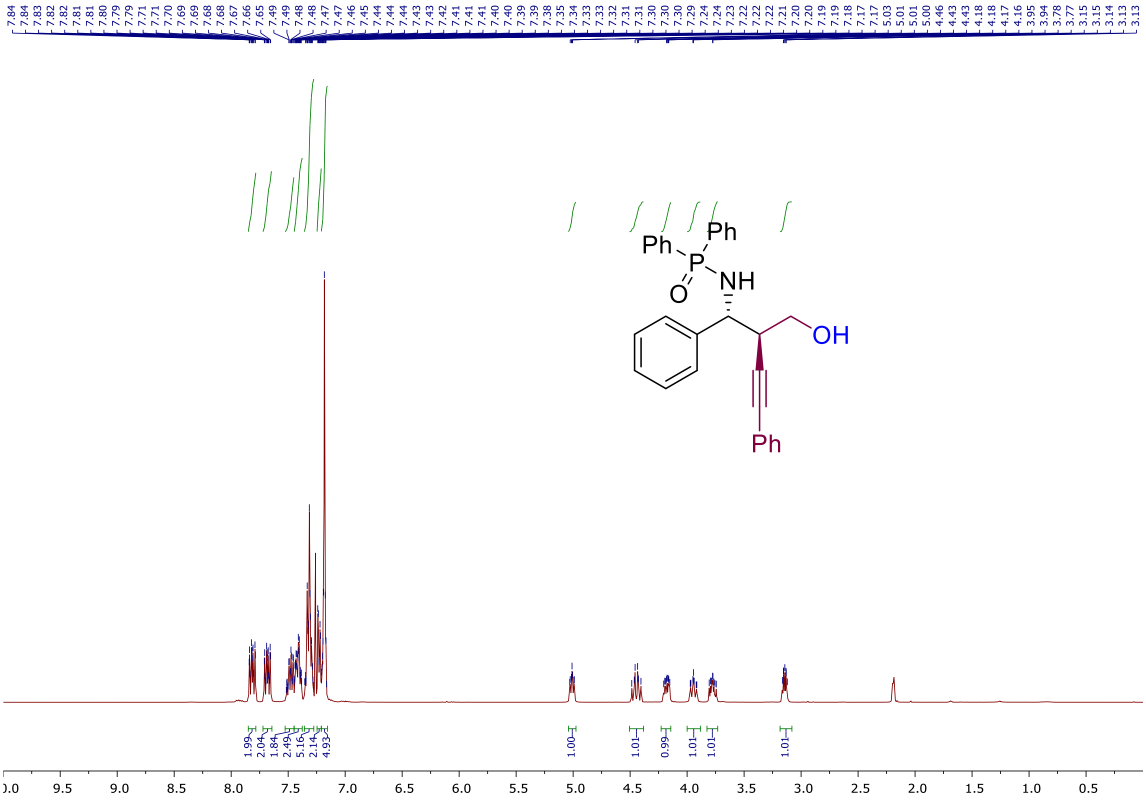

^{13}C NMR of **3a** (101 MHz, $CDCl_3$)

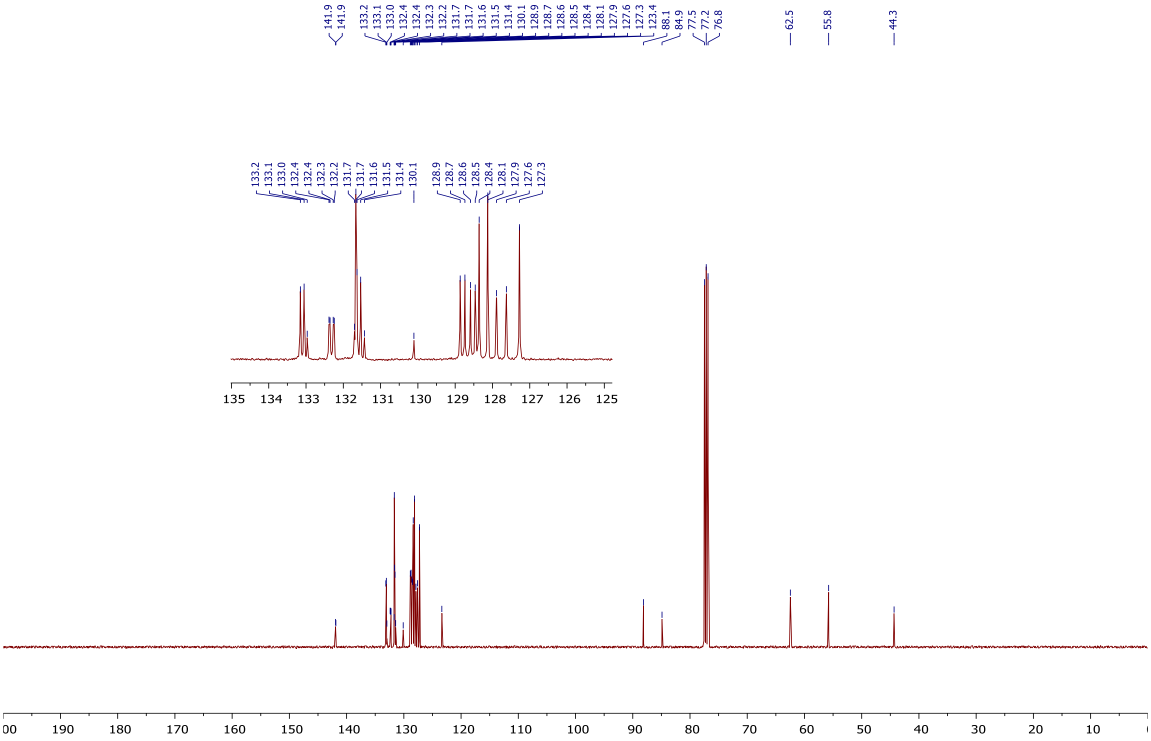

^{1}H NMR of **3b** (500 MHz, DMSO-d_6)

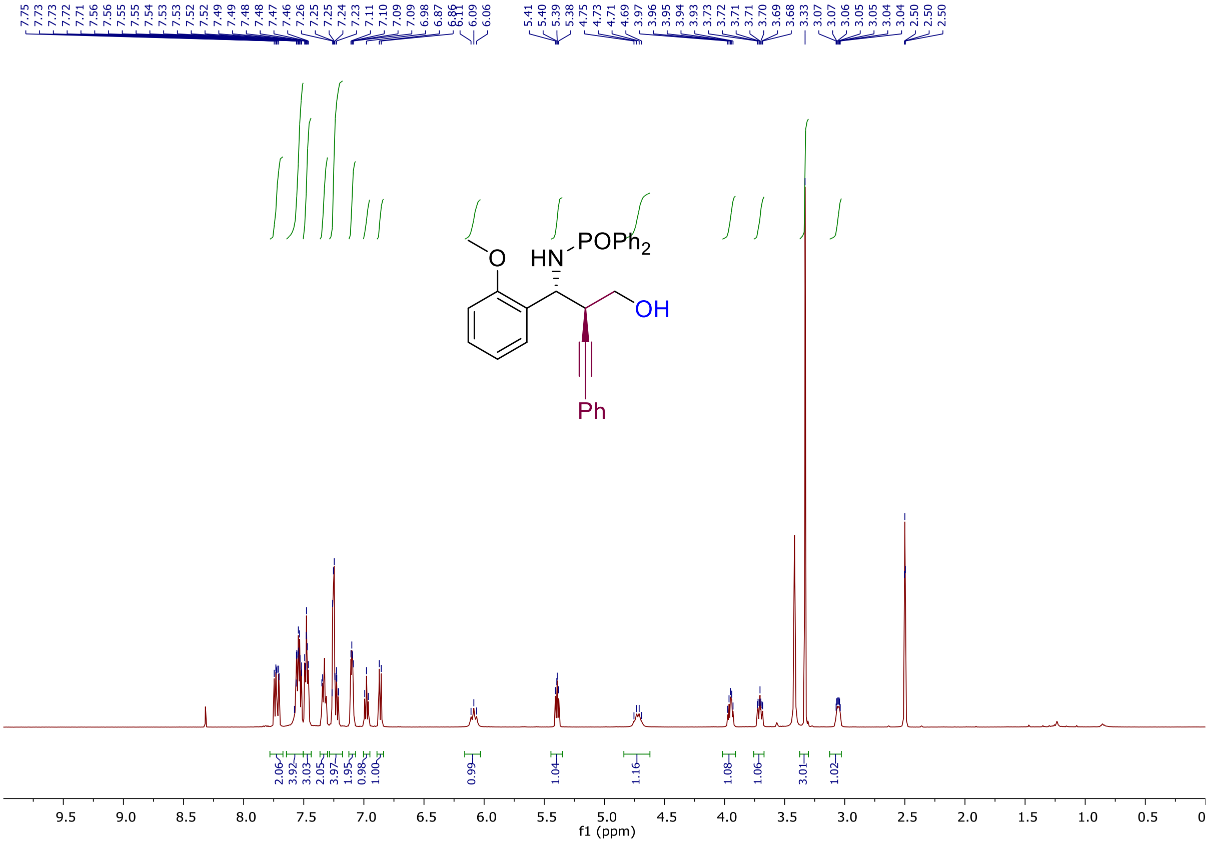

^{13}C NMR of **3b** (126 MHz, DMSO-d_6)

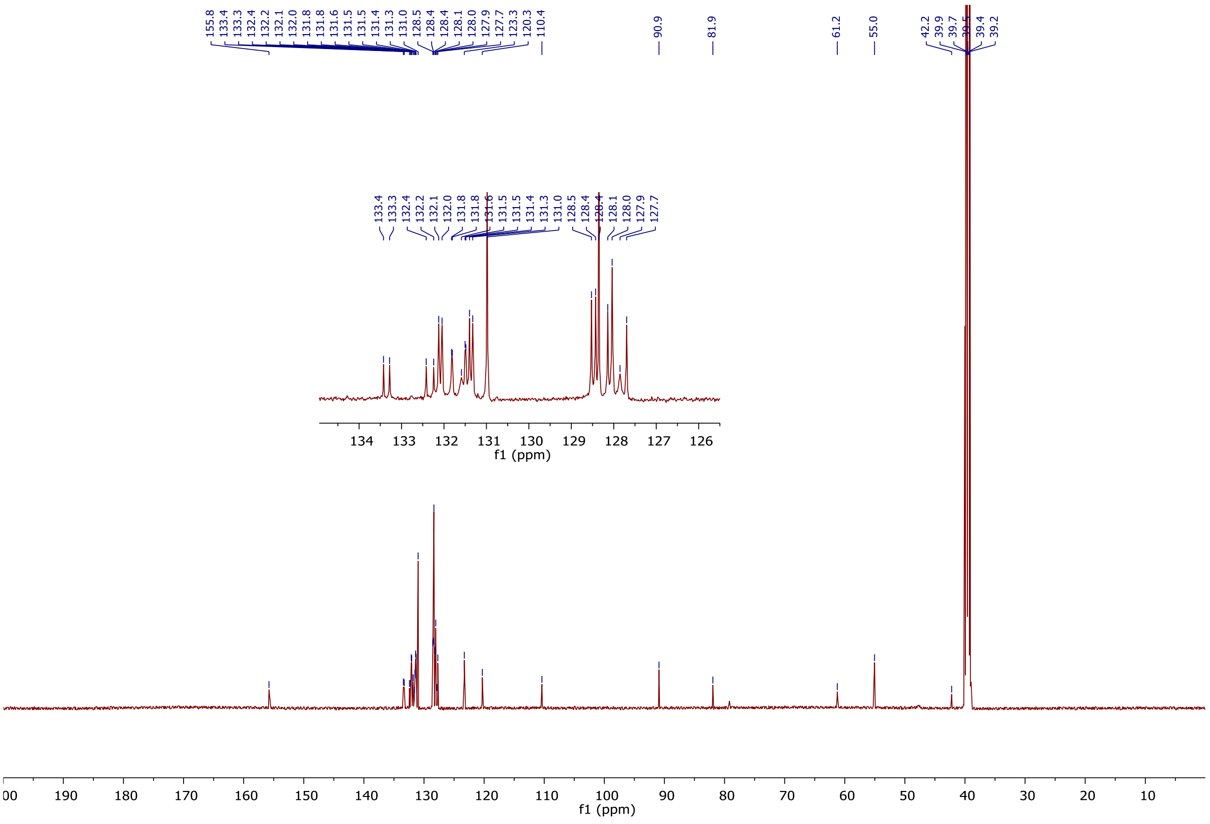

^{1}H NMR of **3c** (400 MHz, $CDCl_3$)

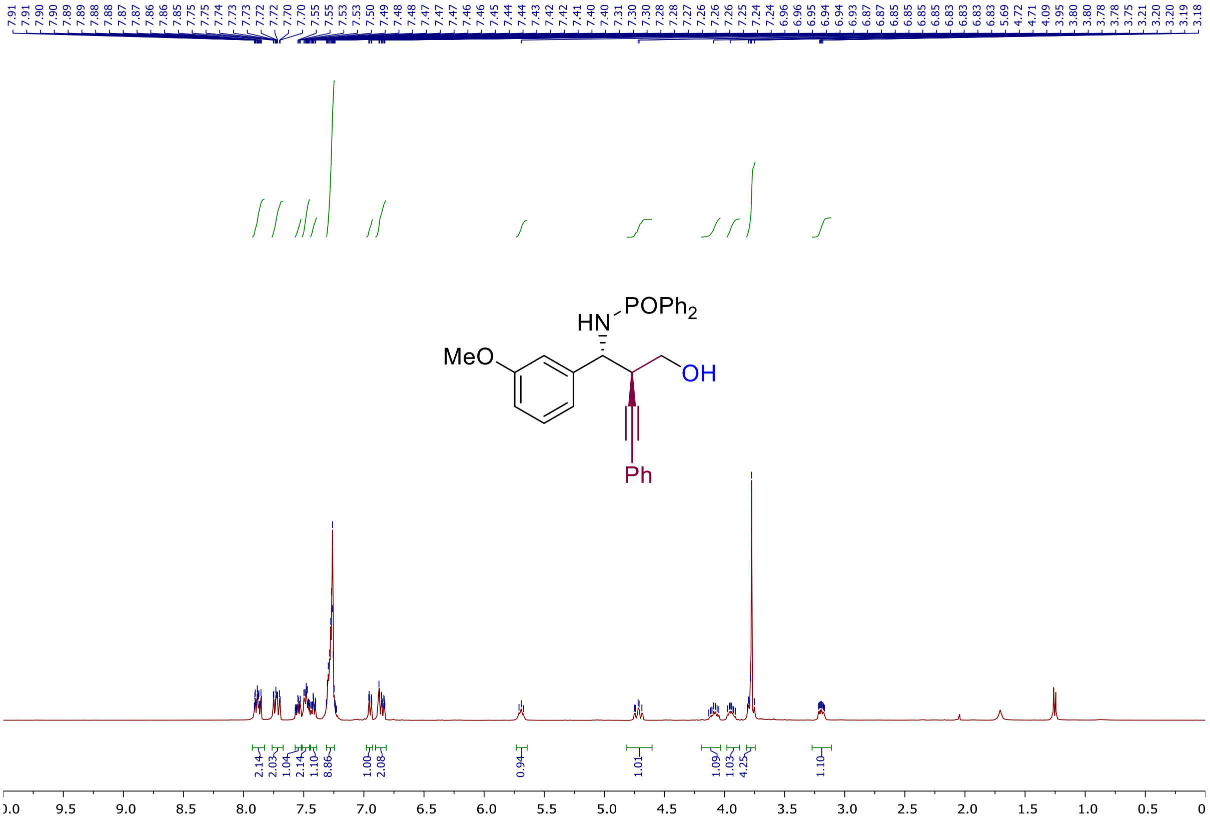

^{13}C NMR of **3c** (101 MHz, $CDCl_3$)

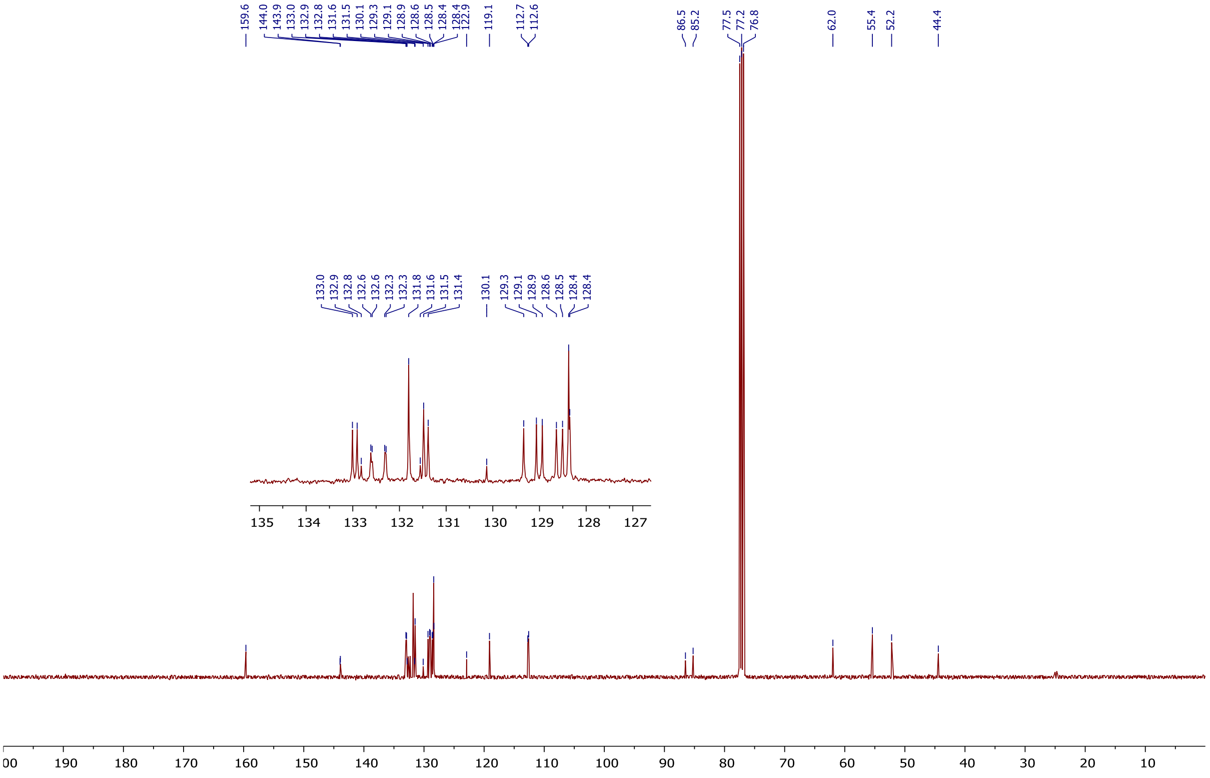

^{1}H NMR of **3d** (400 MHz, $CDCl_3$)

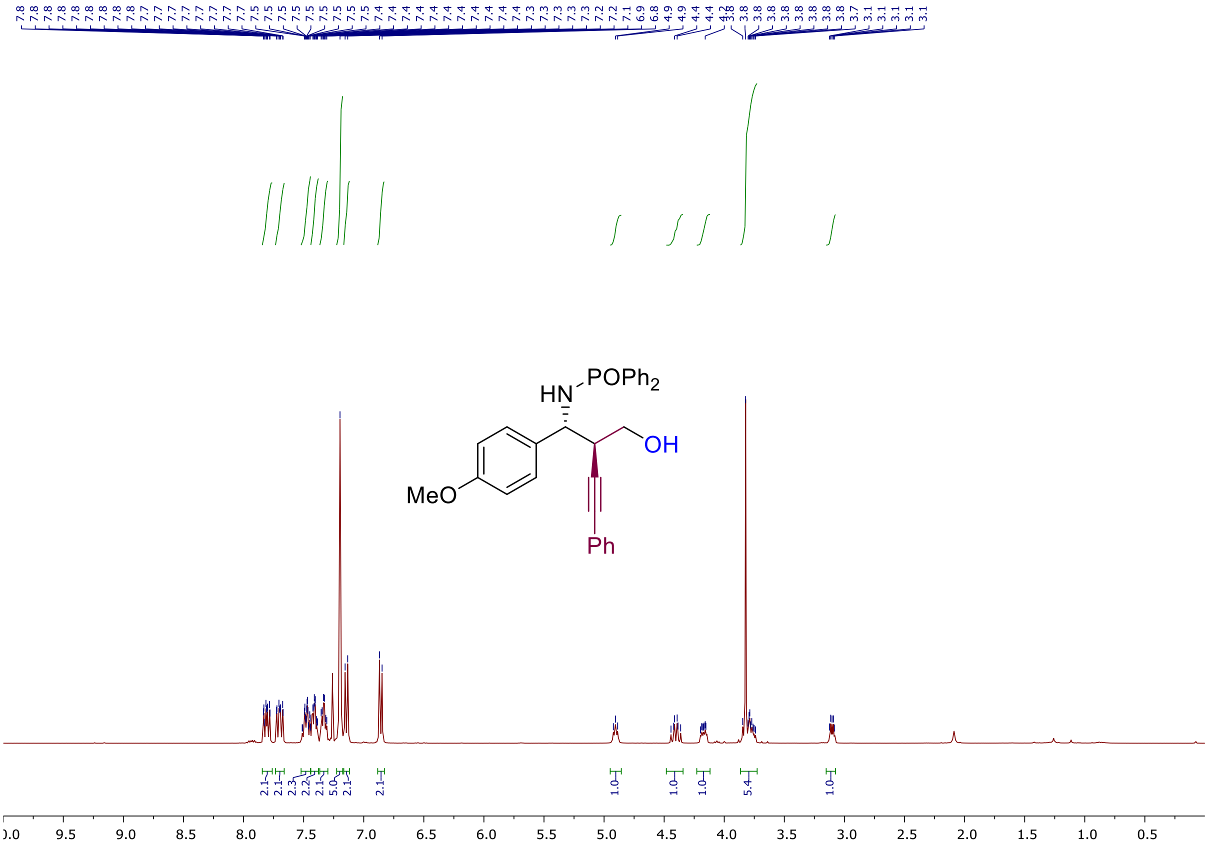

^{13}C NMR of **3d** (101 MHz, $CDCl_3$)

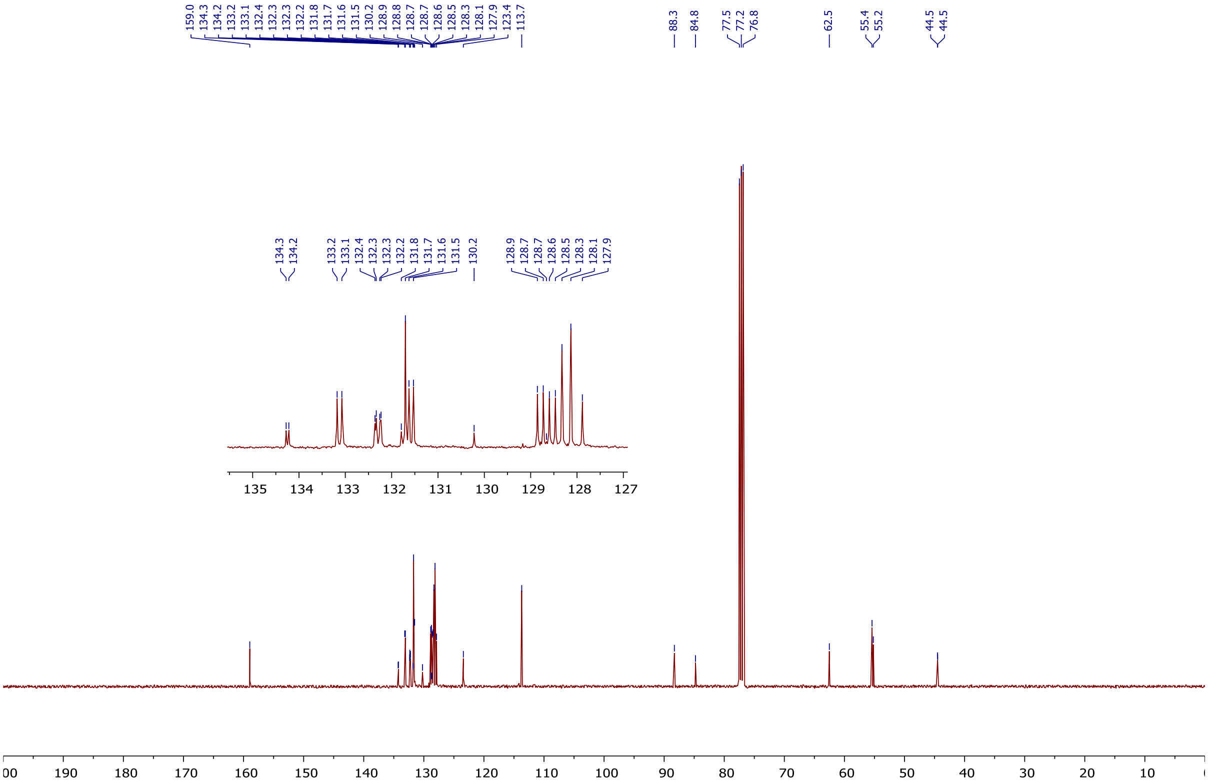

^{1}H NMR of **3e** (500 MHz, DMSO-d_6)

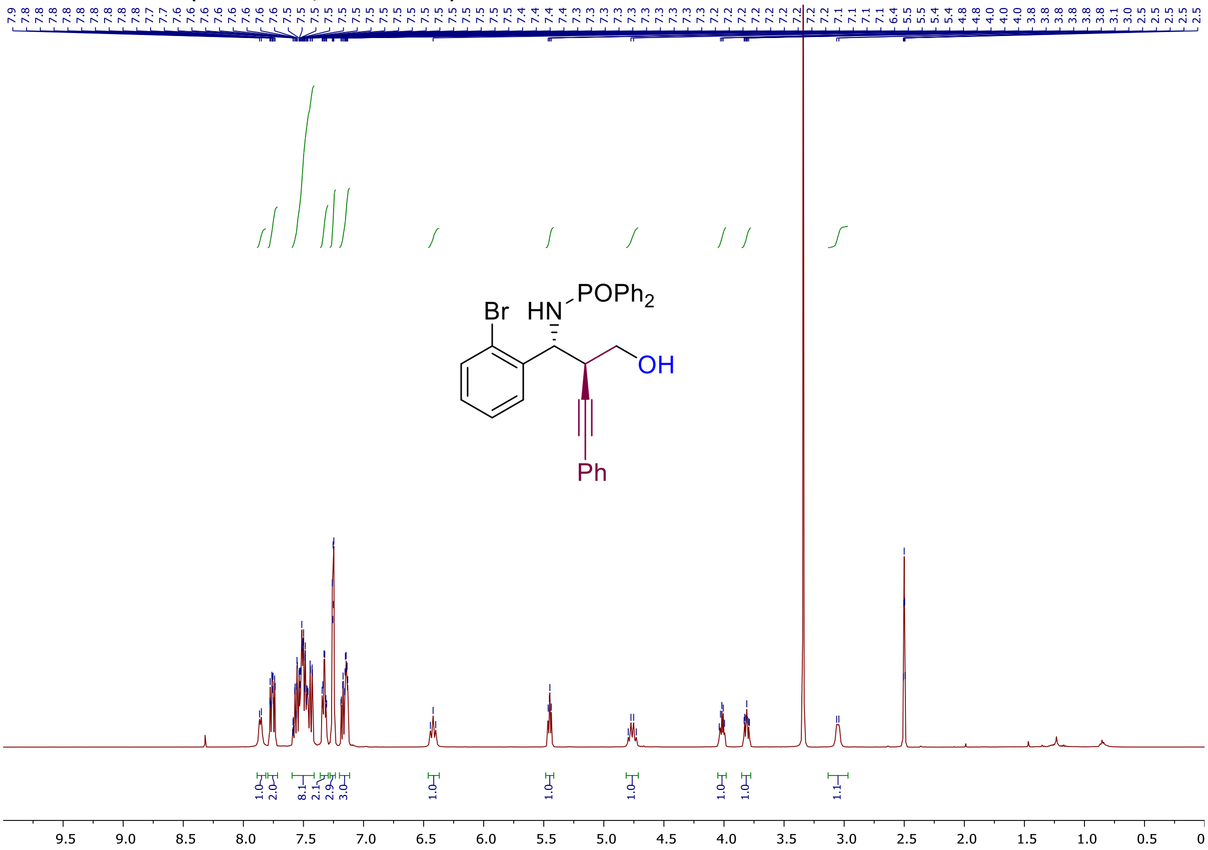

^{13}C NMR of **3e** (126 MHz, DMSO-d_6)

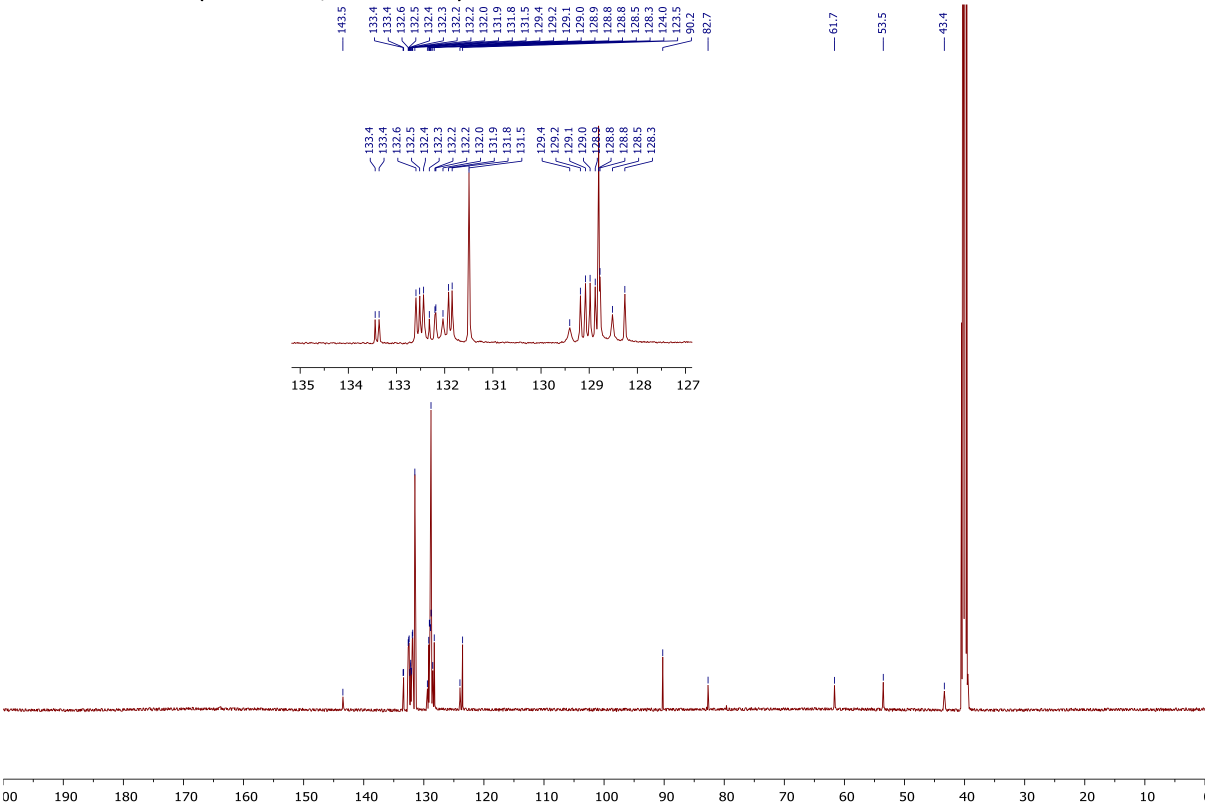

^{1H} NMR of **3f** (400 MHz, $CDCl_3$)

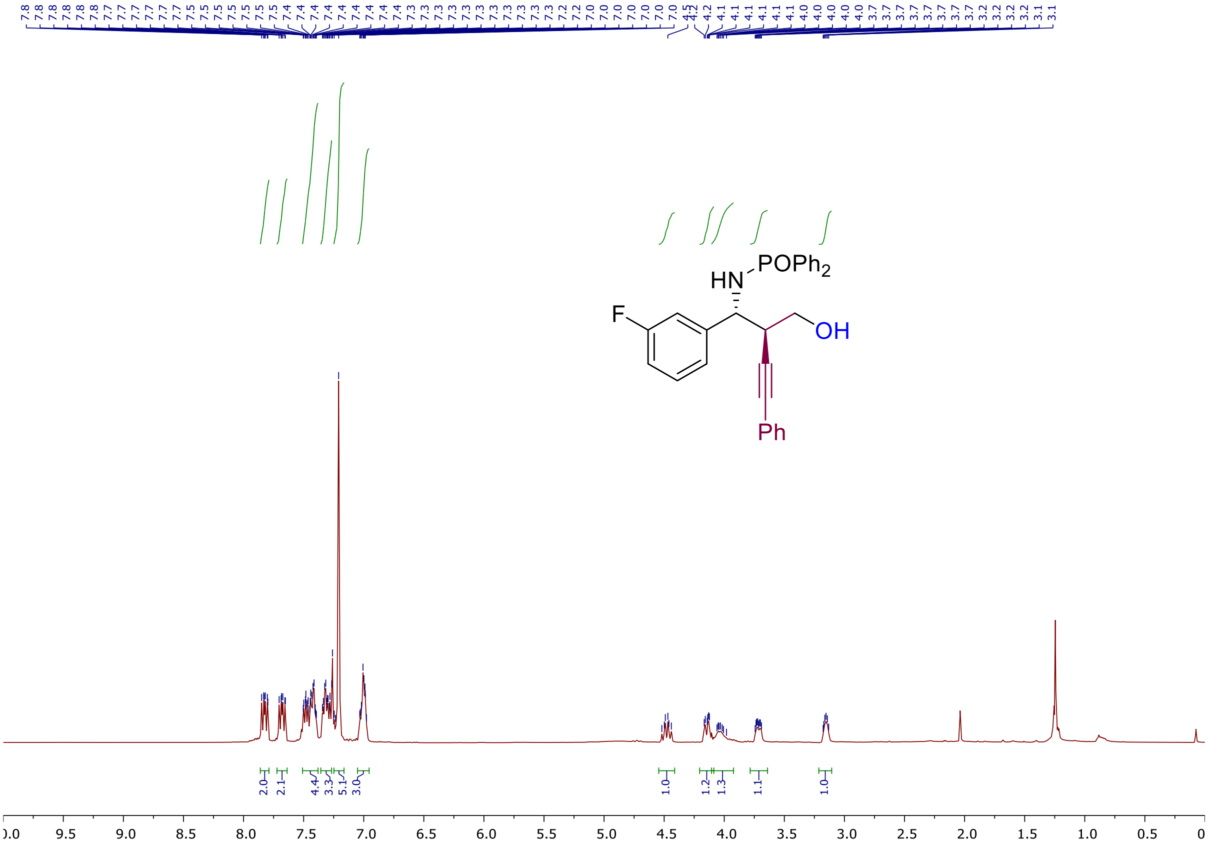

^{13}C NMR of **3f** (101 MHz, $CDCl_3$)

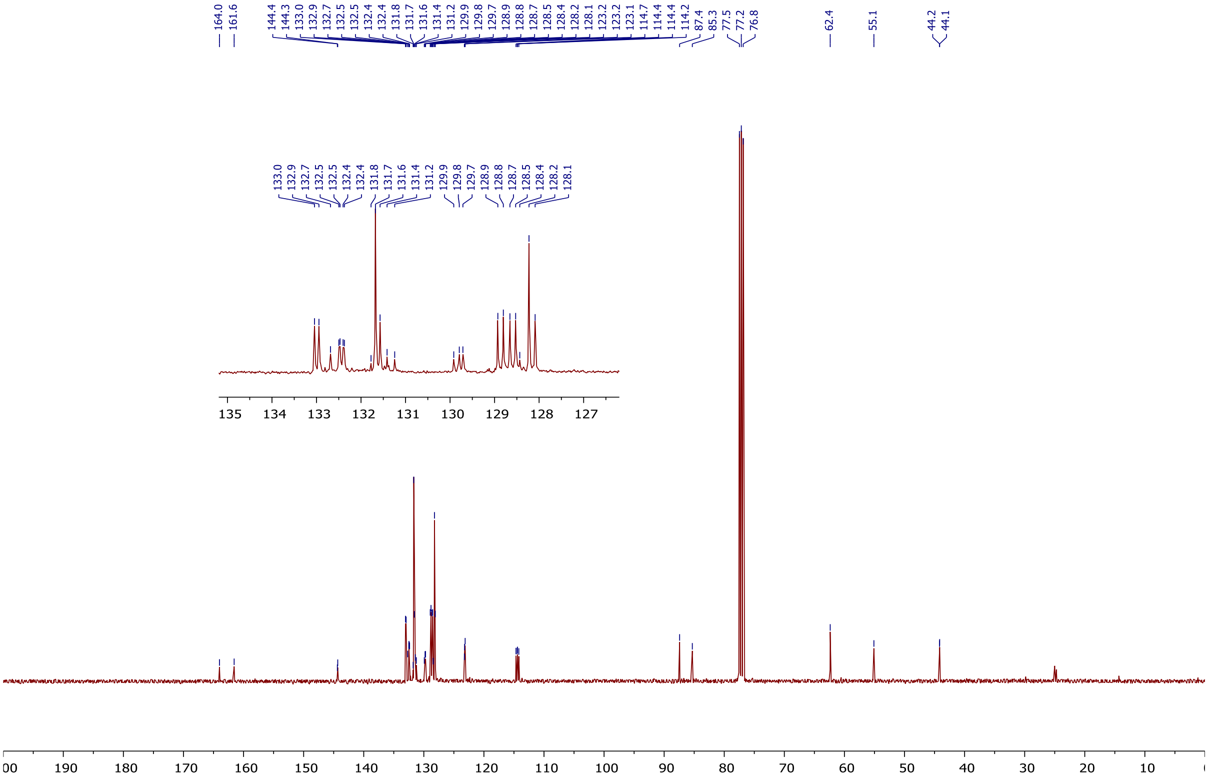

^{1}H NMR of **3g** (400 MHz, $CDCl_3$)

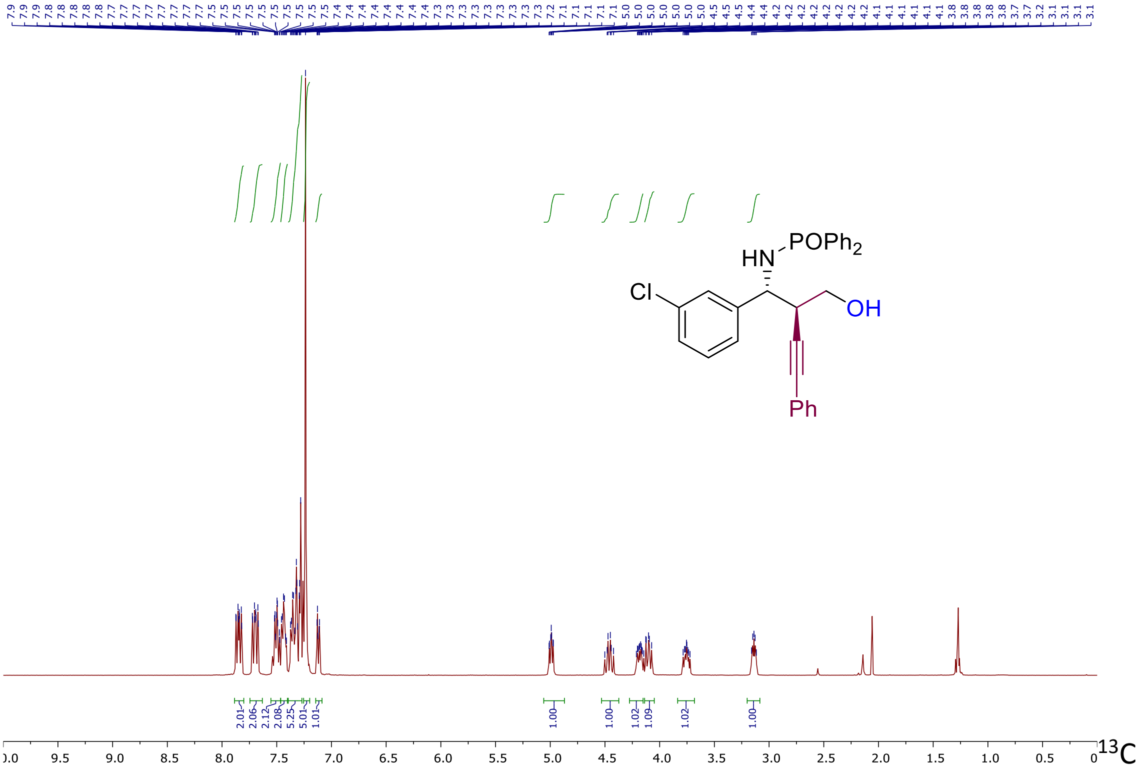

^{13}C NMR of **3g** (101 MHz, $CDCl_3$)

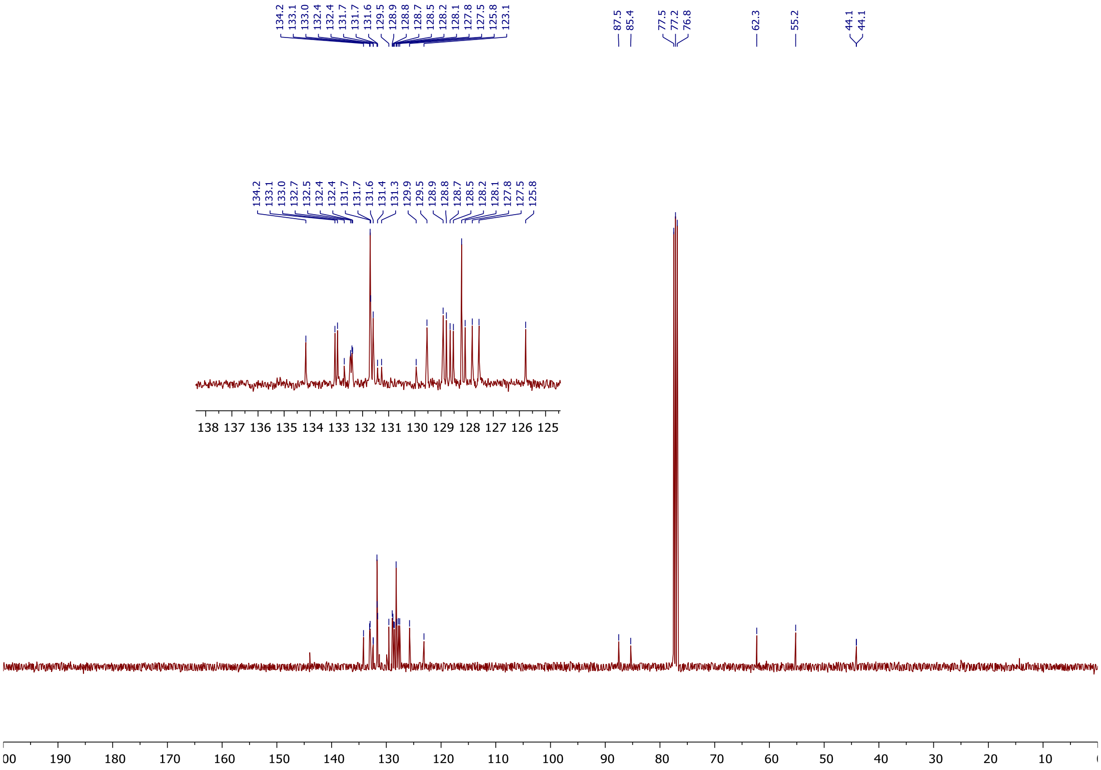

^{1}H NMR of **3h** (400 MHz, Acetone-*d*6)

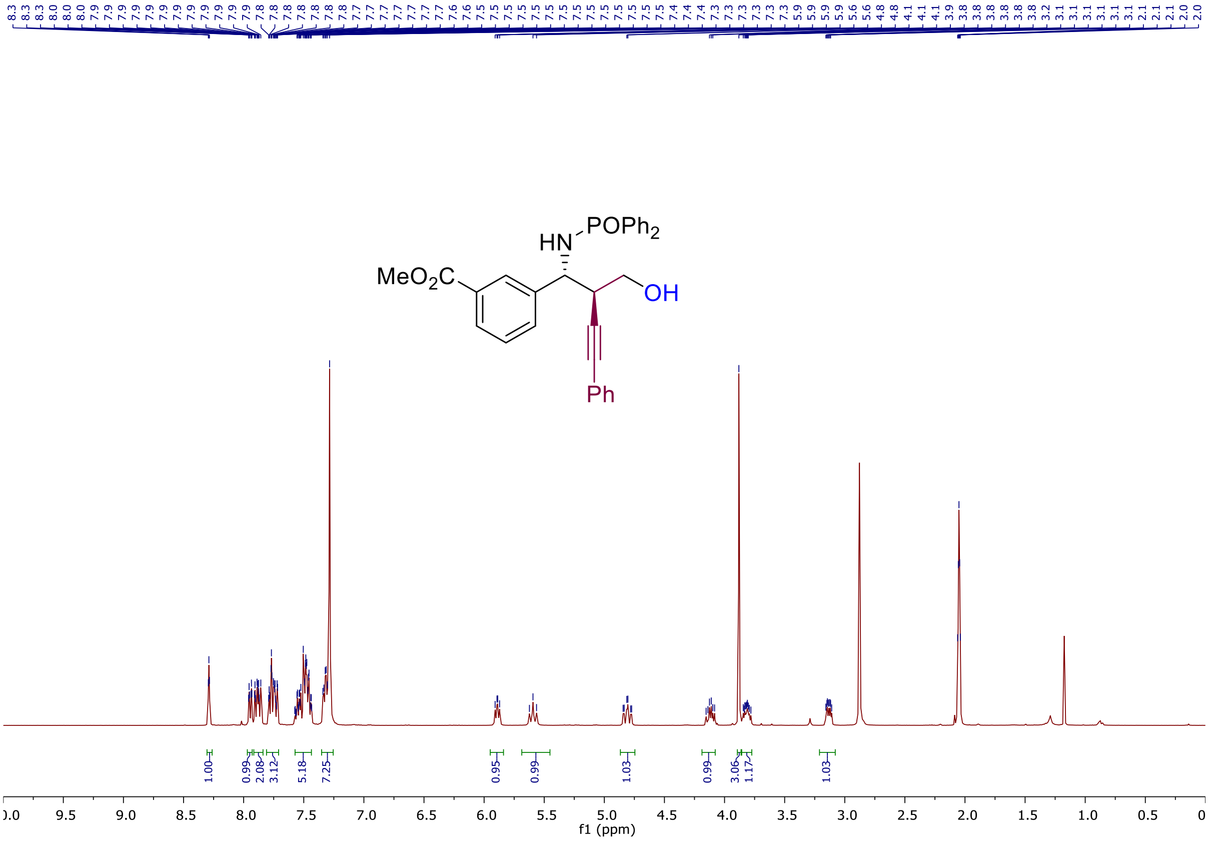

^{13}C NMR of **3h** (101 MHz, Acetone-*d*6)

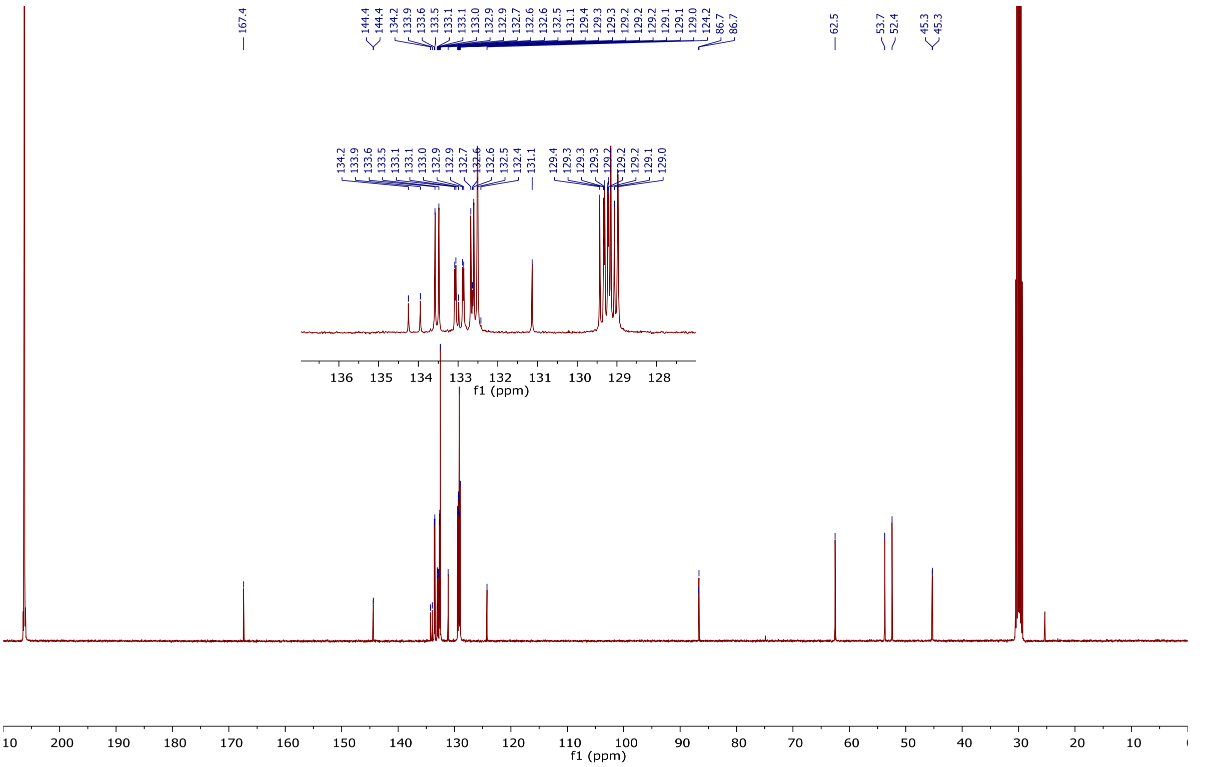

^{1}H NMR of **3i** (400 MHz, $CDCl_3$)

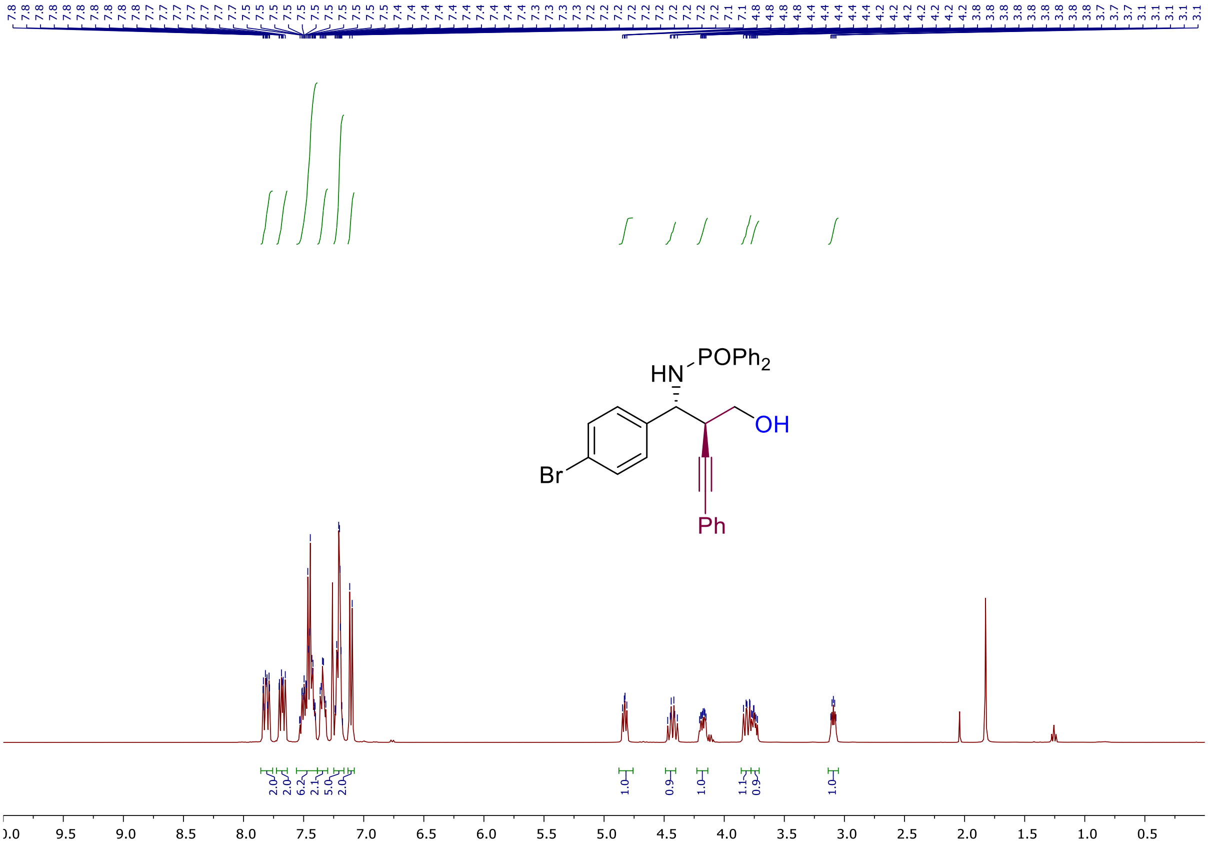

^{13}C NMR of **3i** (101 MHz, $CDCl_3$)

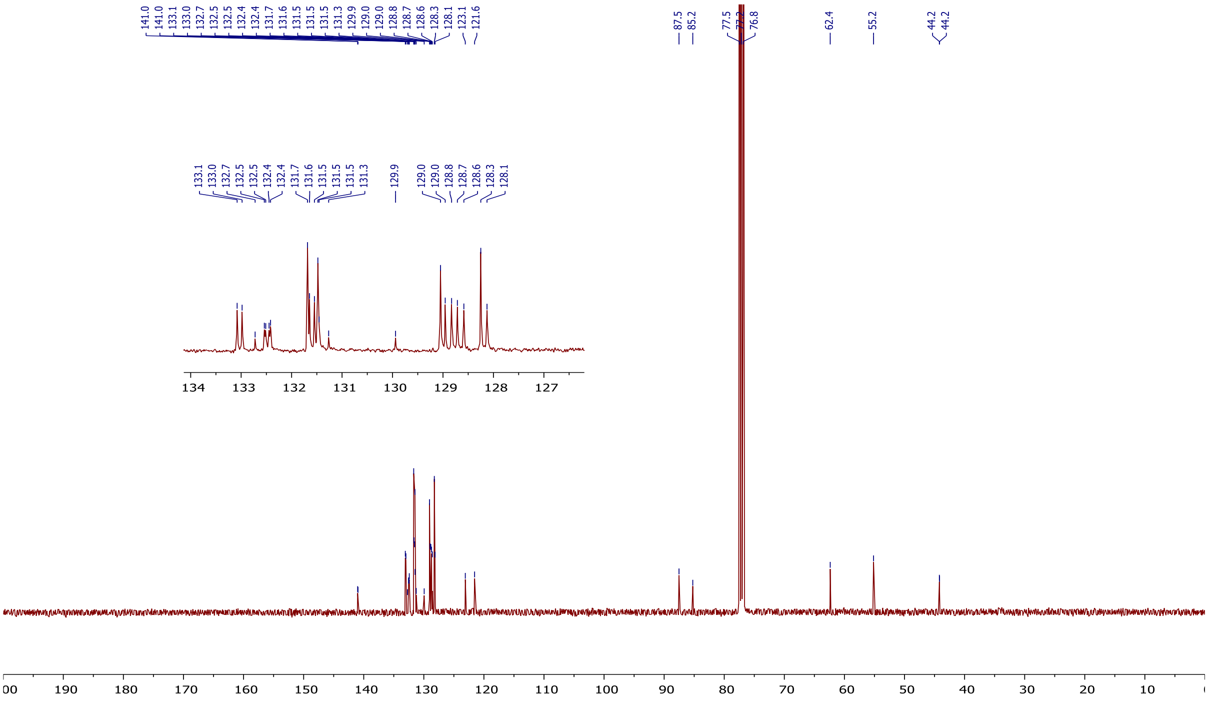

^{1}H NMR of **3j** (500 MHz, $CDCl_3$)

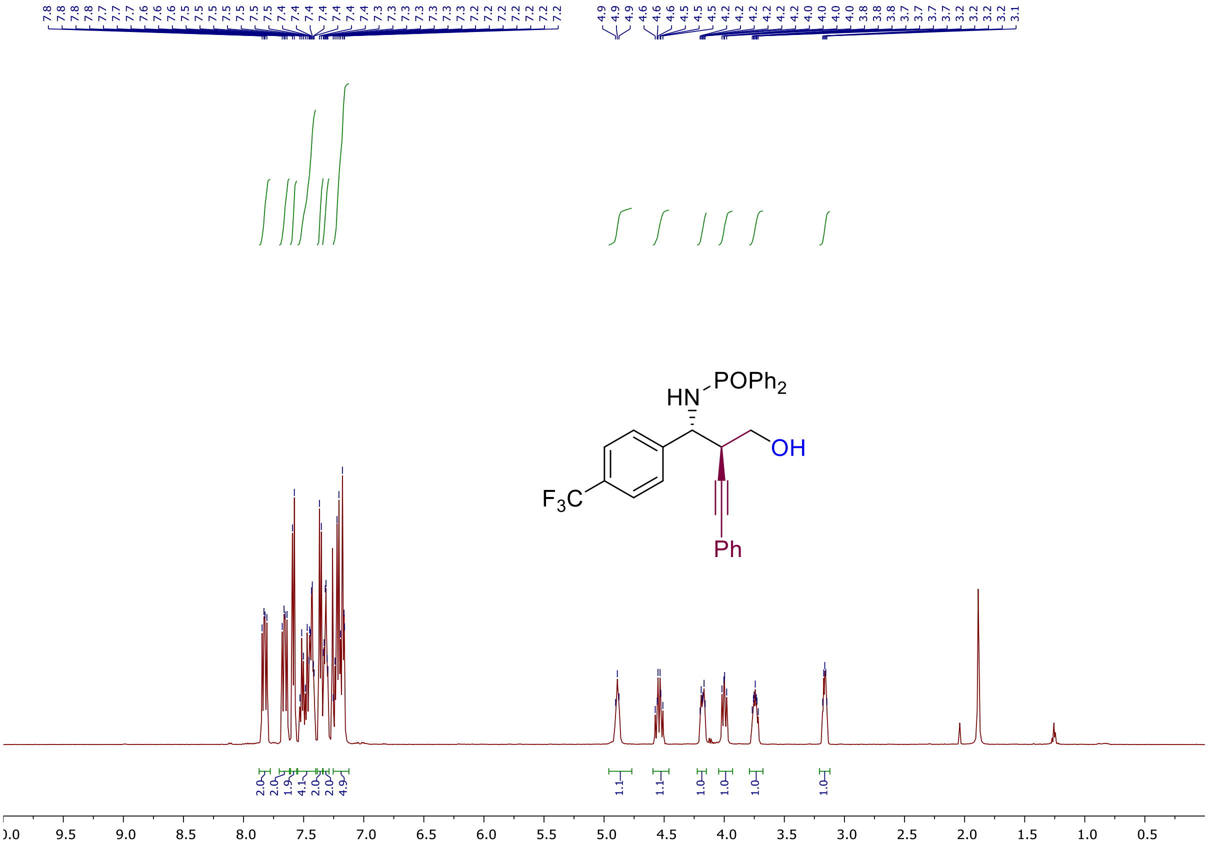

^{13}C NMR of **3j** (126 MHz, $CDCl_3$)

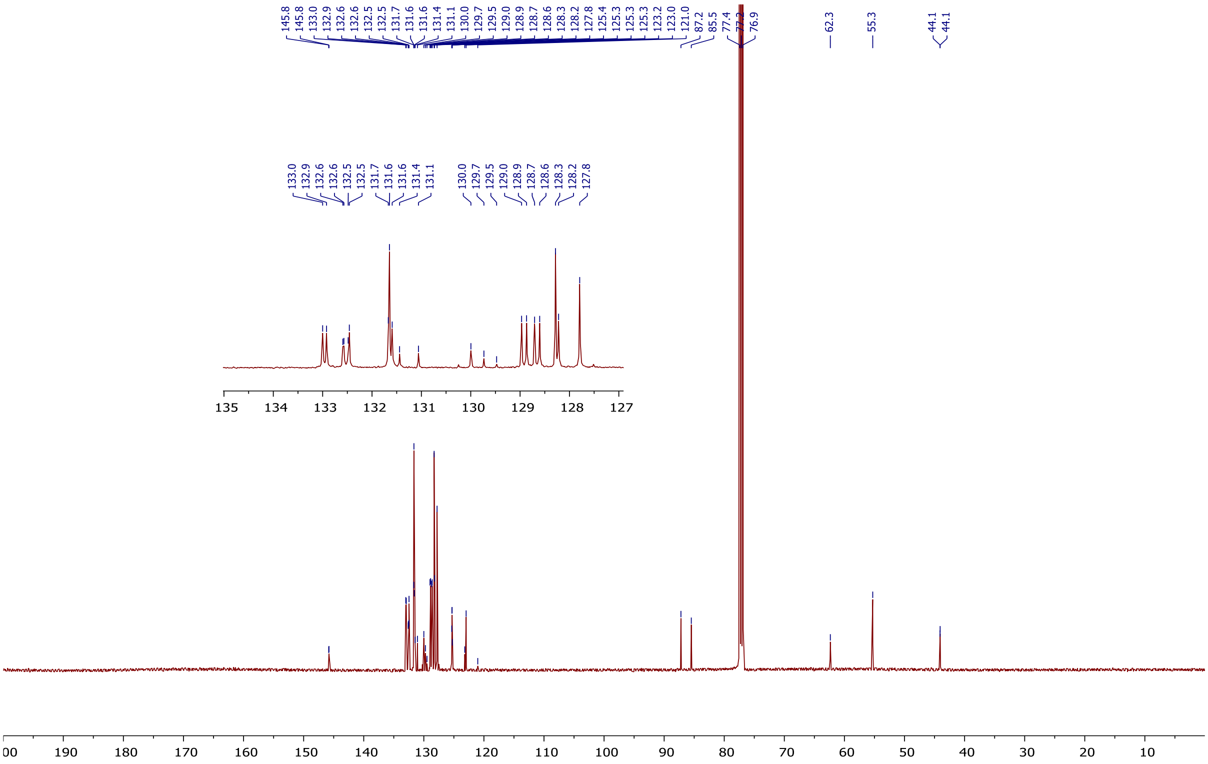

^{1}H NMR of **3k** (400 MHz, $CDCl_3$)

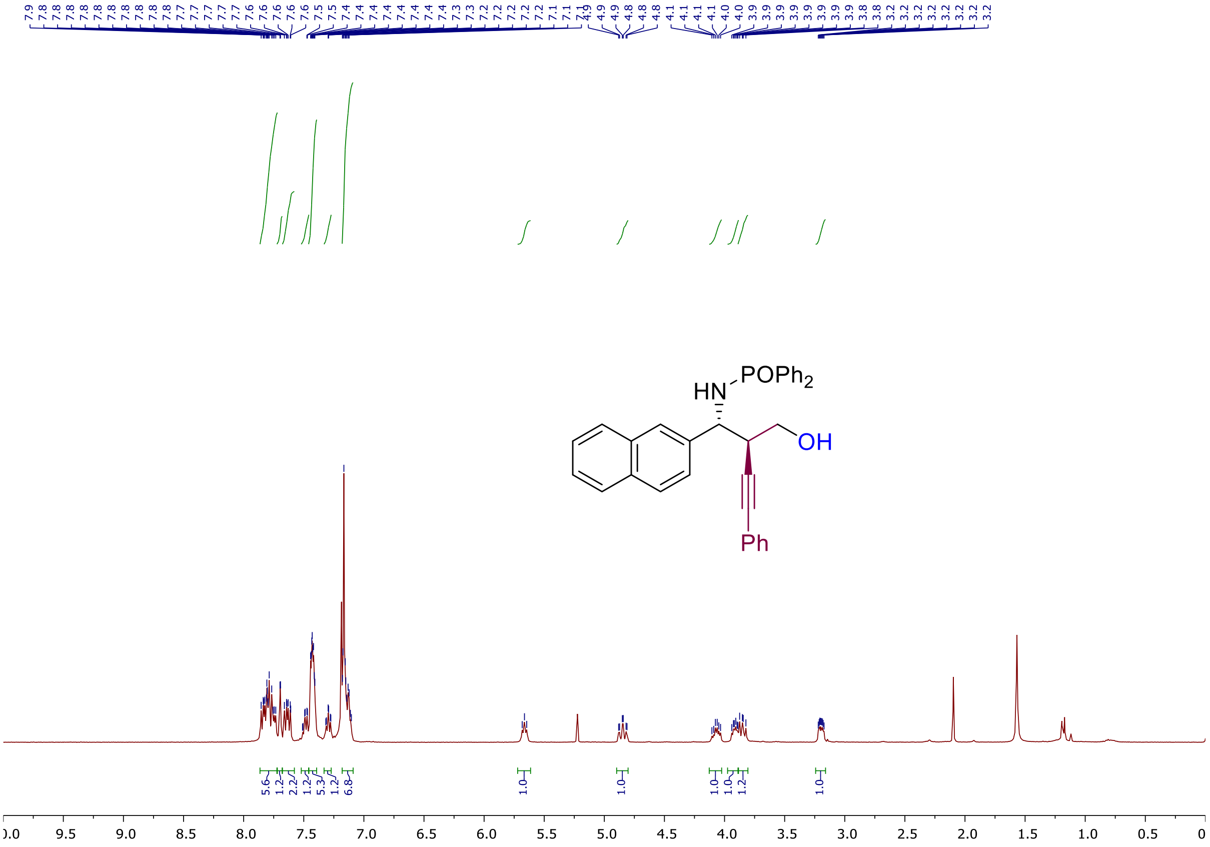

^{13}C NMR of **3k** (101 MHz, $CDCl_3$)

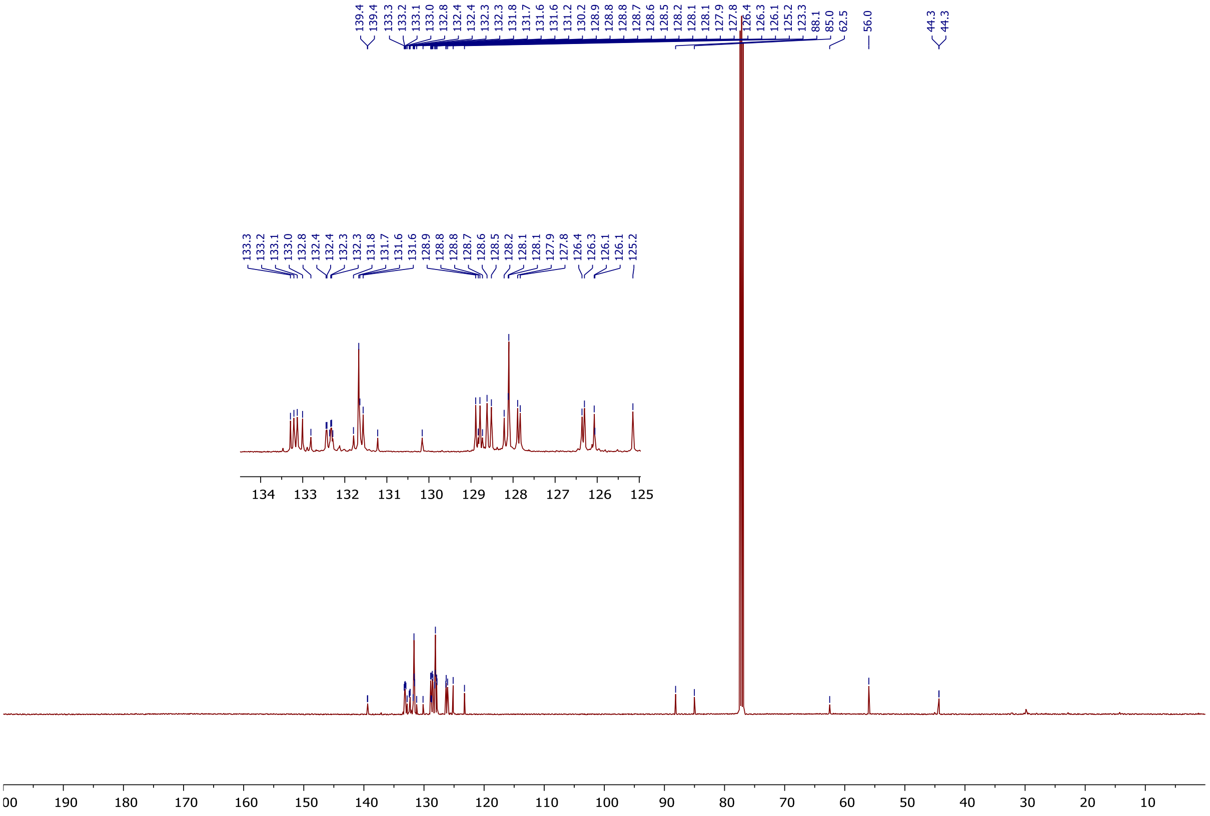

^{1}H NMR of **3l** (400 MHz, $CDCl_3$)

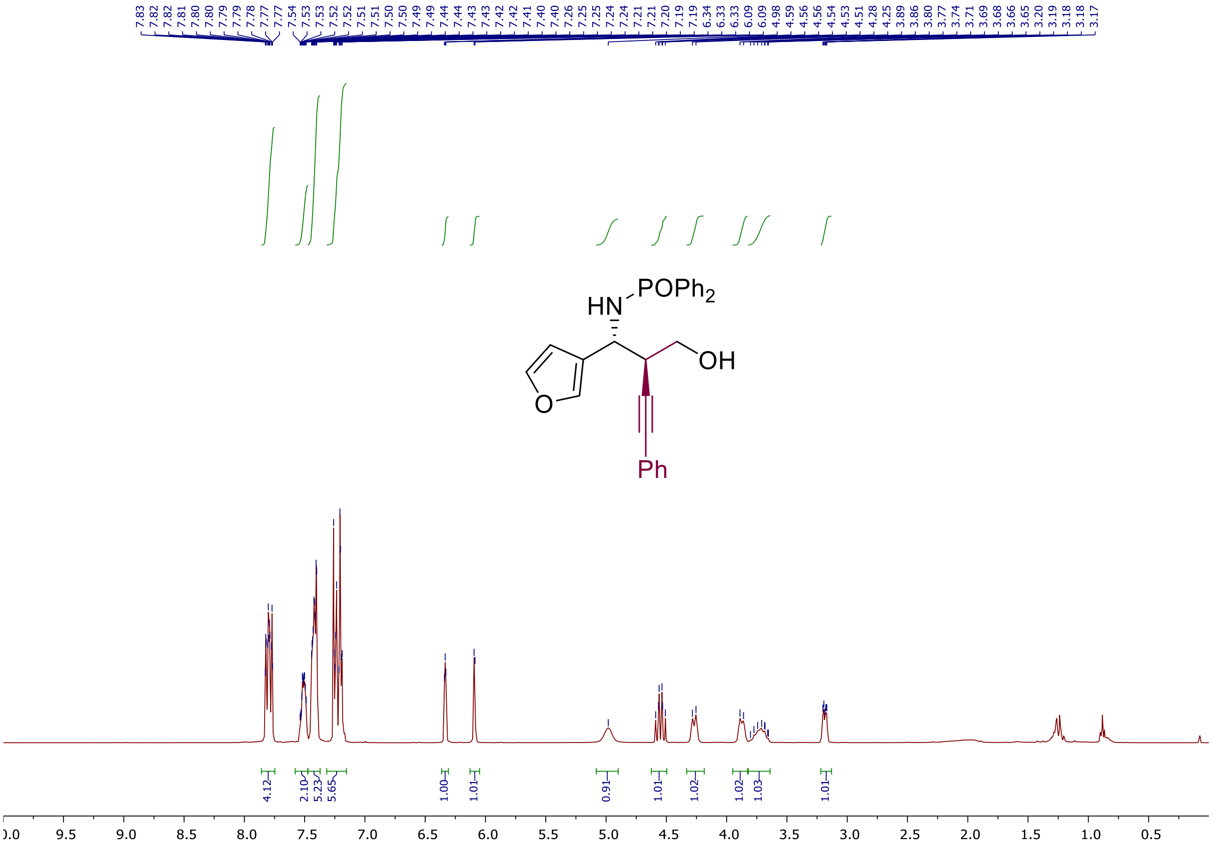

^{13}C NMR of **3l** (101 MHz, $CDCl_3$)

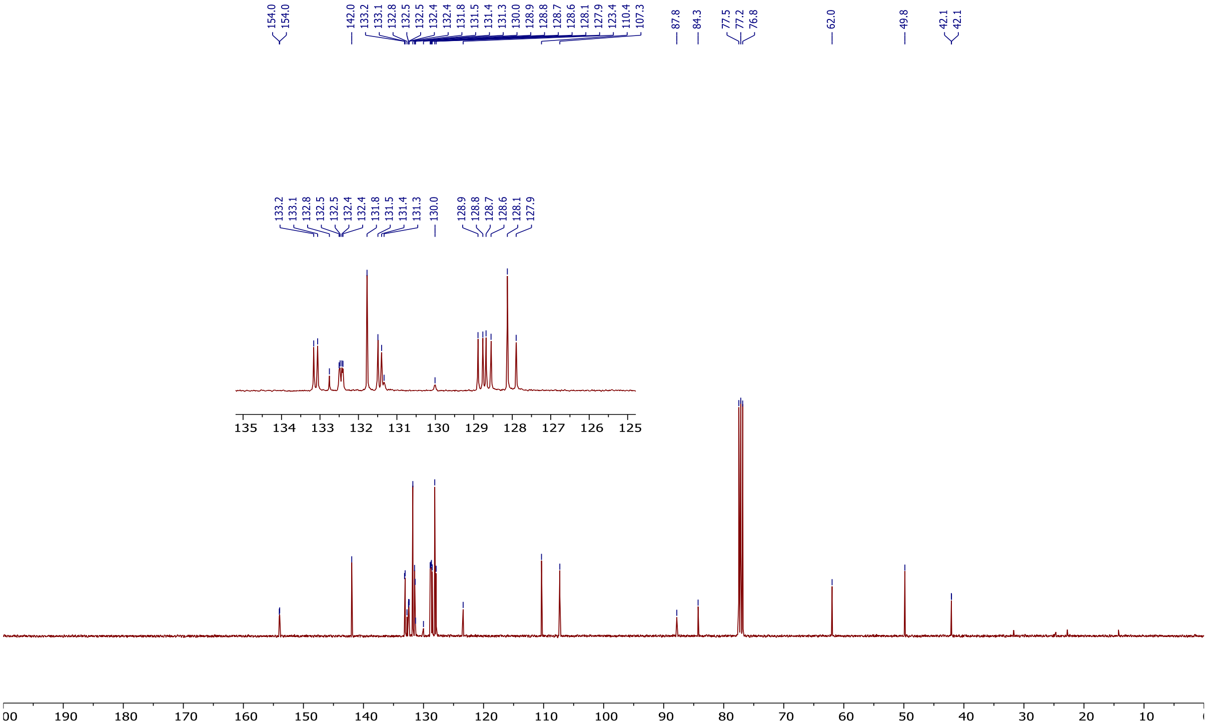

^{1}H NMR of **3m** (400 MHz, CD_3OD)

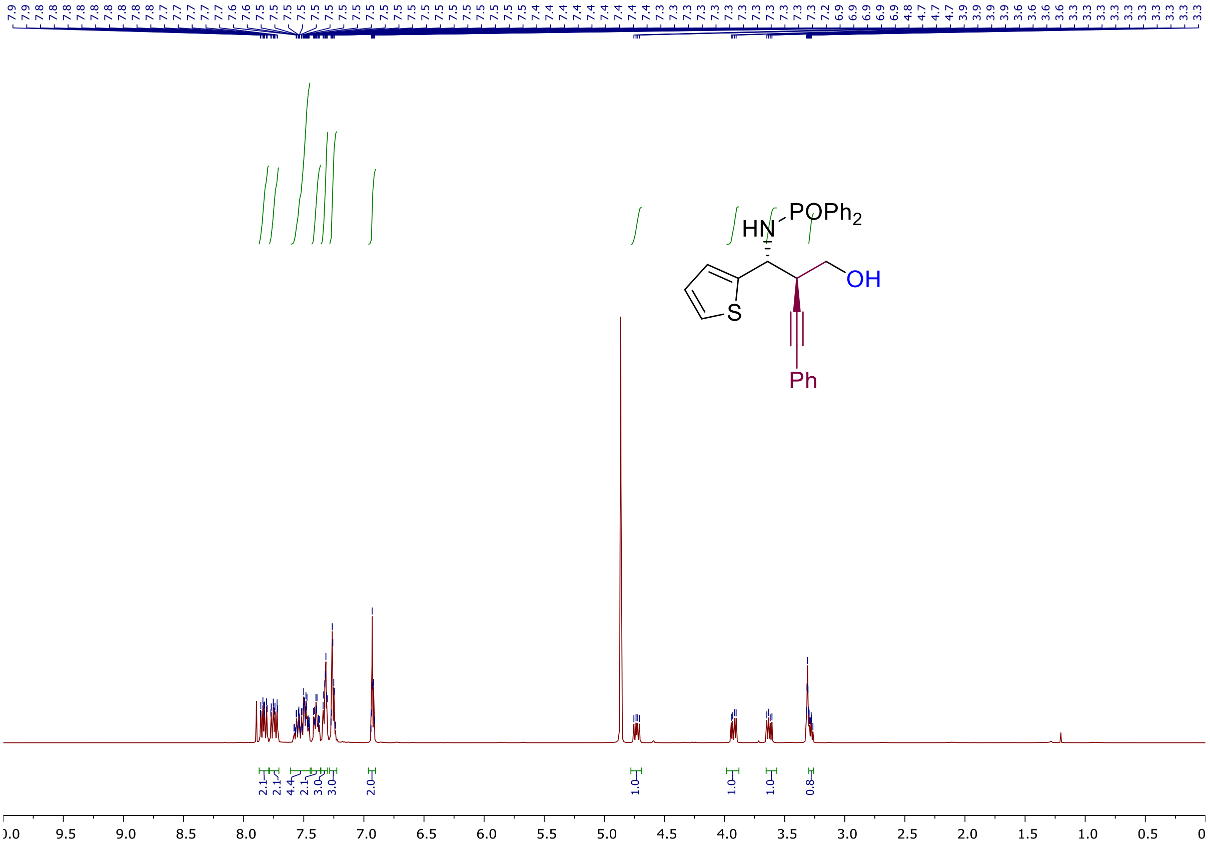

^{13}C NMR of **3m** (101 MHz, CD_3OD)

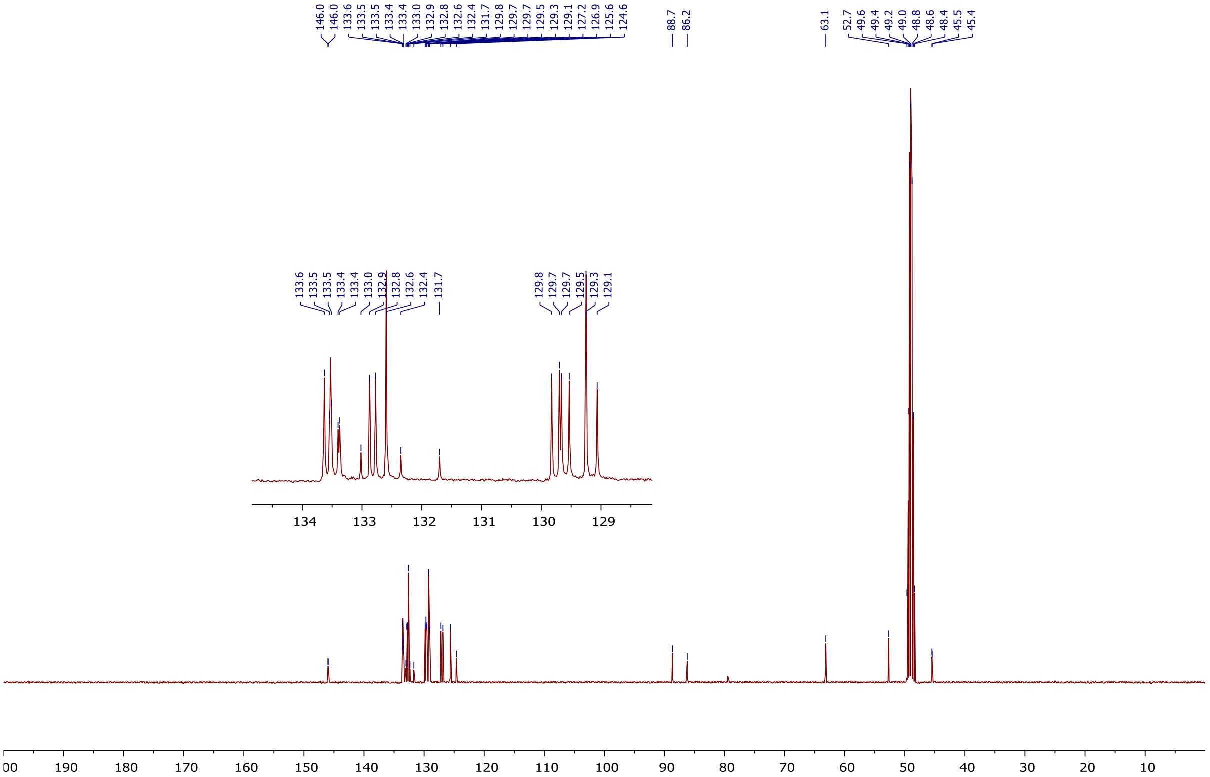

^{1}H NMR of **3n** (400 MHz, $CDCl_3$)

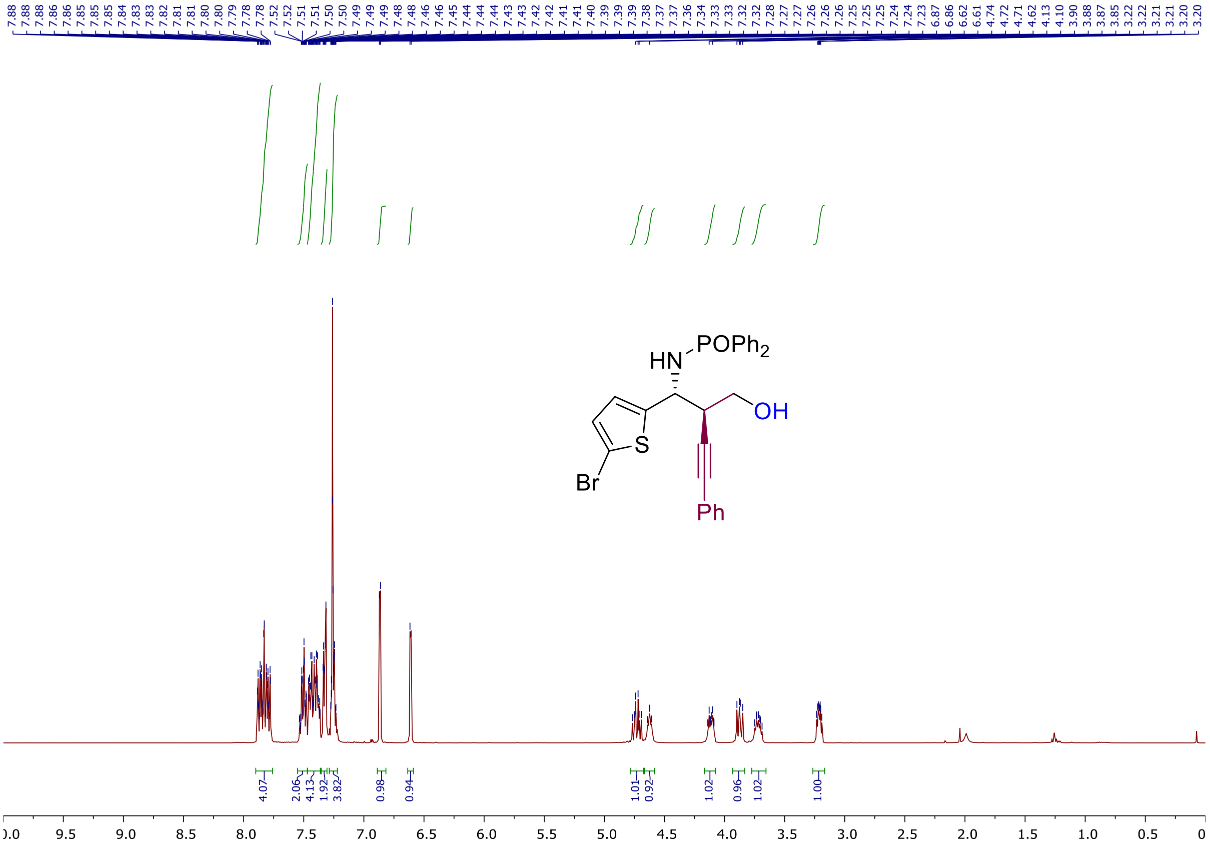

^{13}C NMR of **3n** (101 MHz, $CDCl_3$)

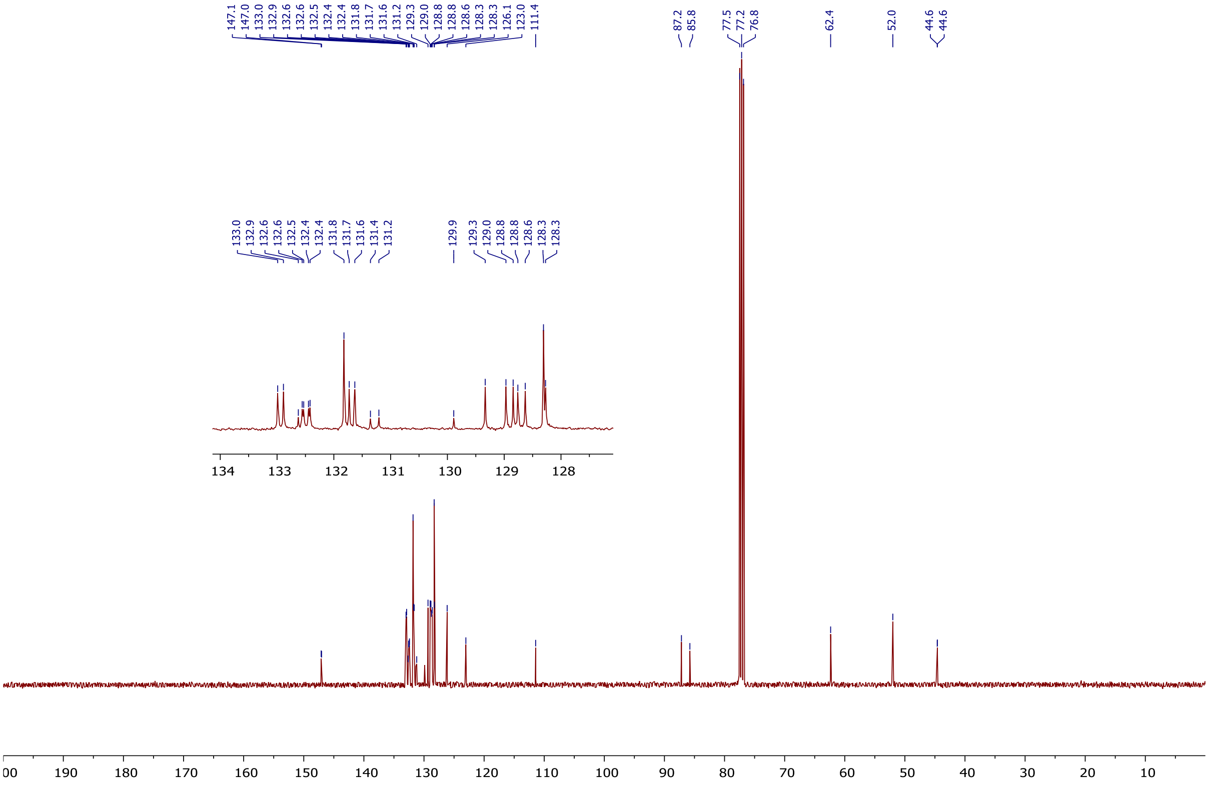

^{1}H NMR of **3o** (400 MHz, $CDCl_3$)

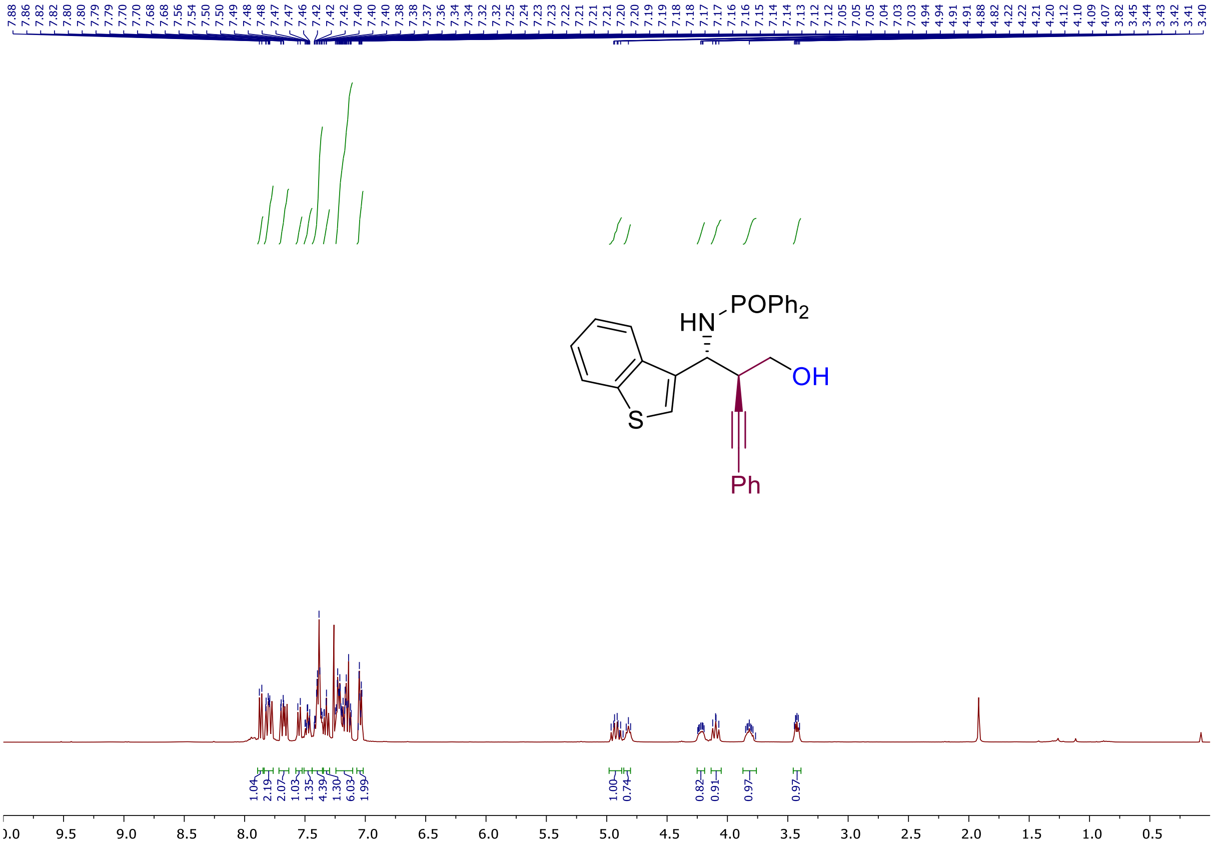

^{13}C NMR of **3o** (101 MHz, $CDCl_3$)

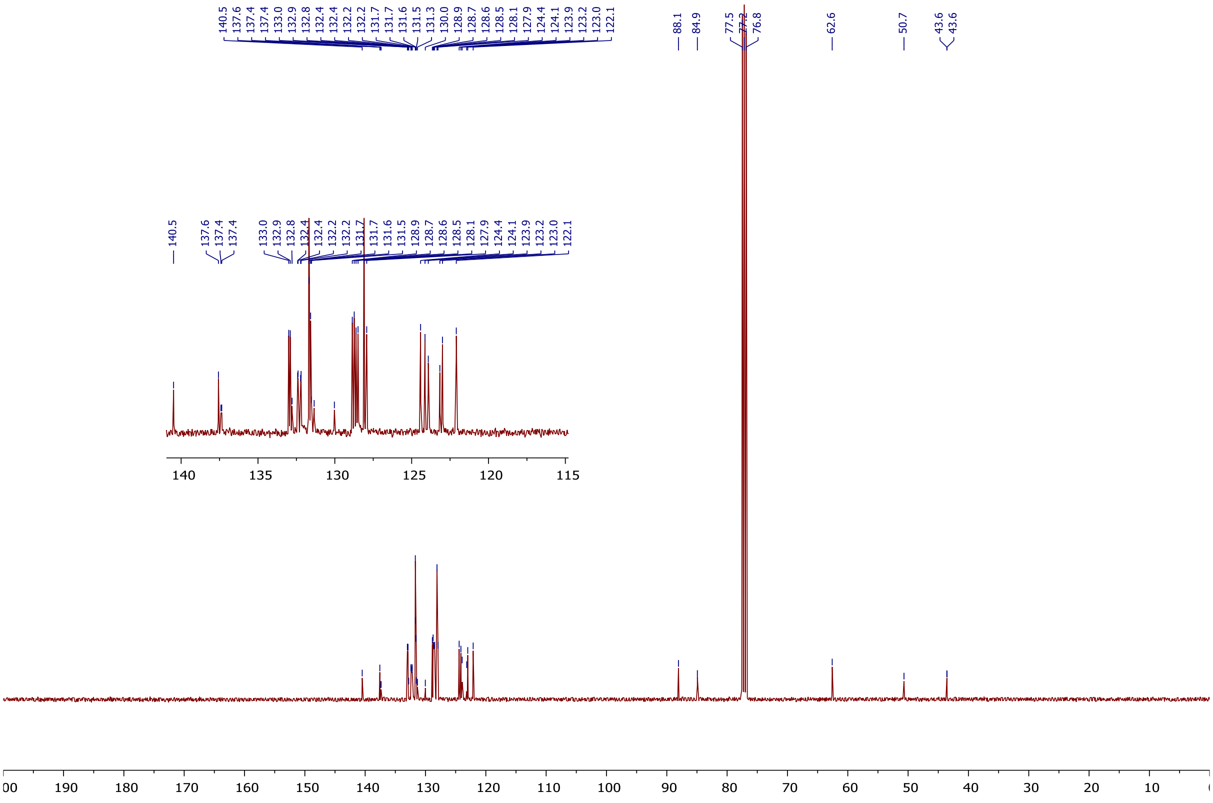

^{1}H NMR of **4a** (400 MHz, $CDCl_3$)

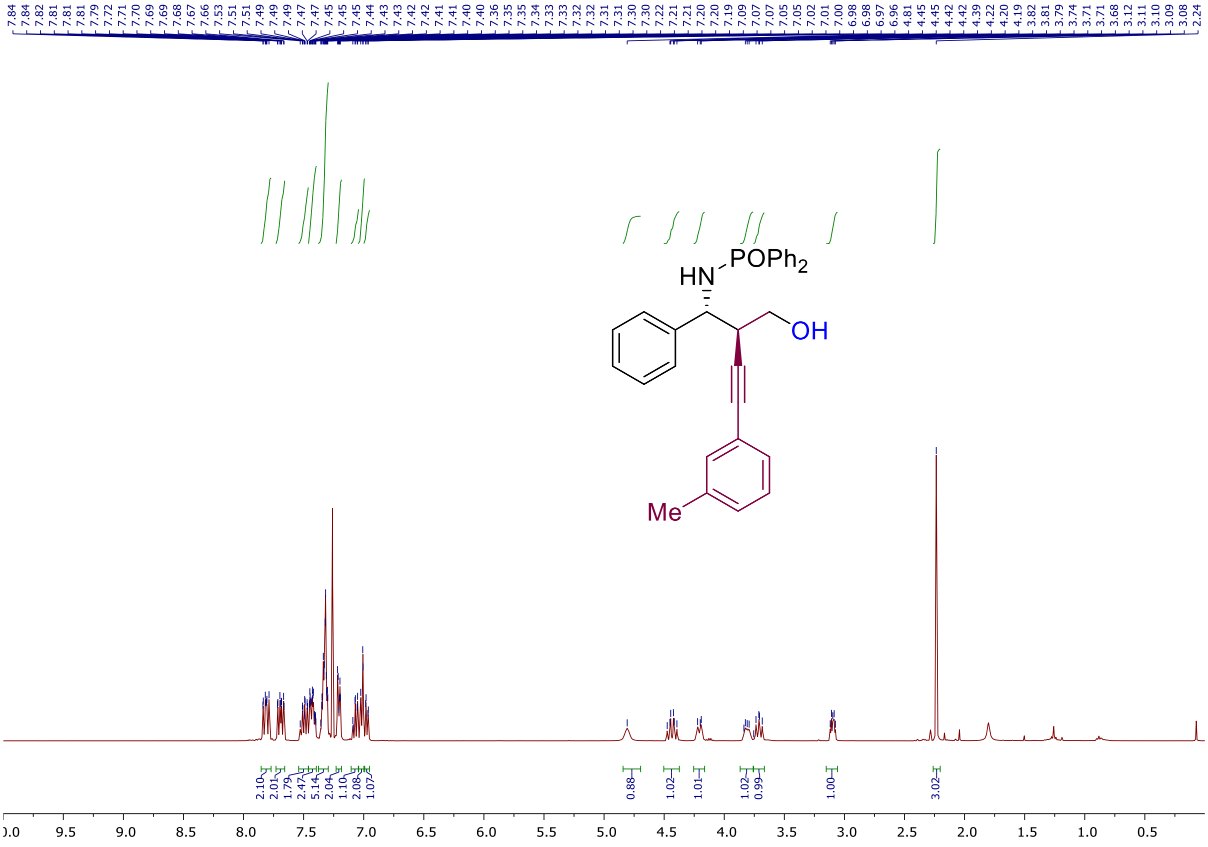

^{1}H NMR of **4a** (101 MHz, $CDCl_3$)

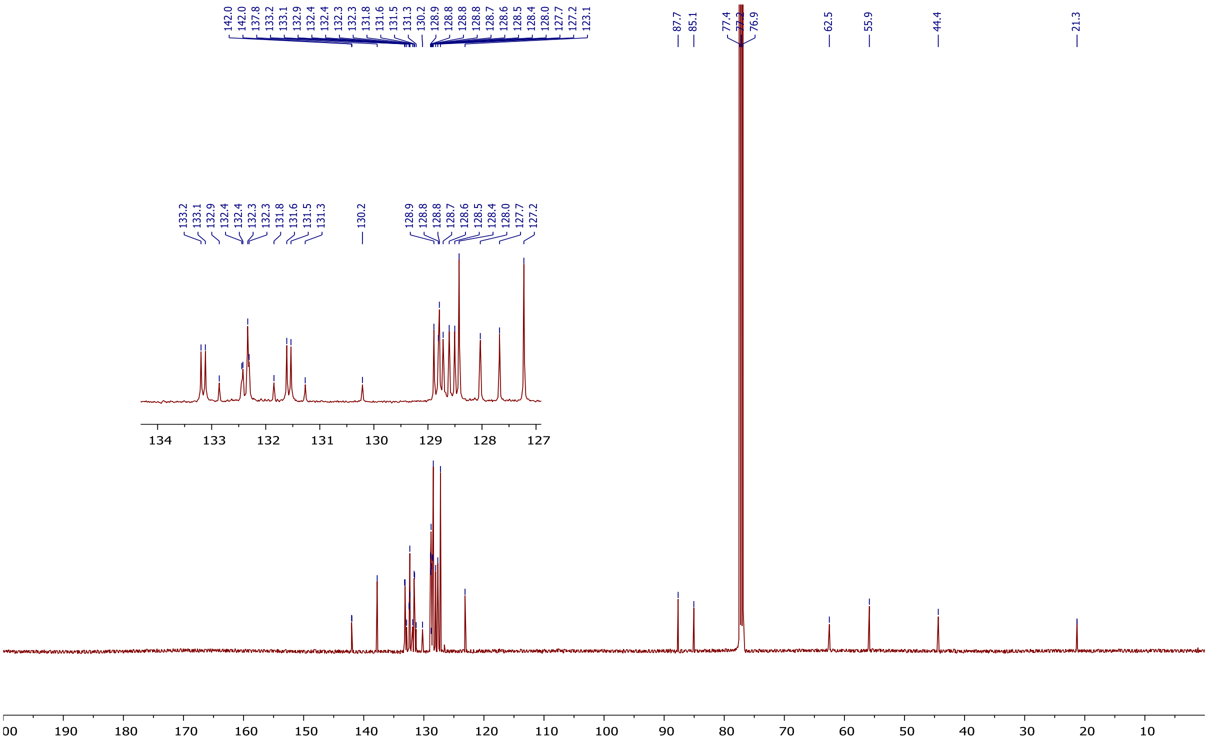

^{1}H NMR of **4b** (500 MHz, $CDCl_3$)

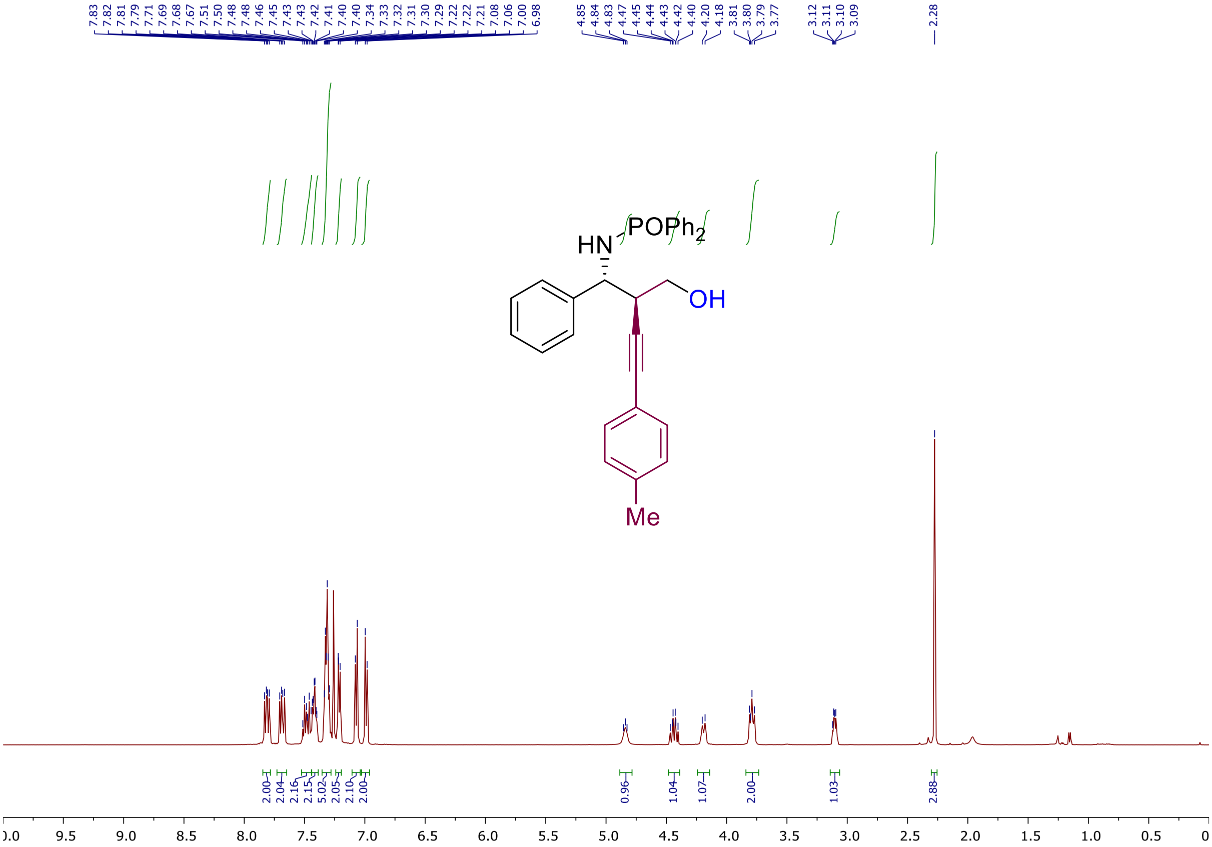

^{13}C NMR of **4b** (126 MHz, $CDCl_3$)

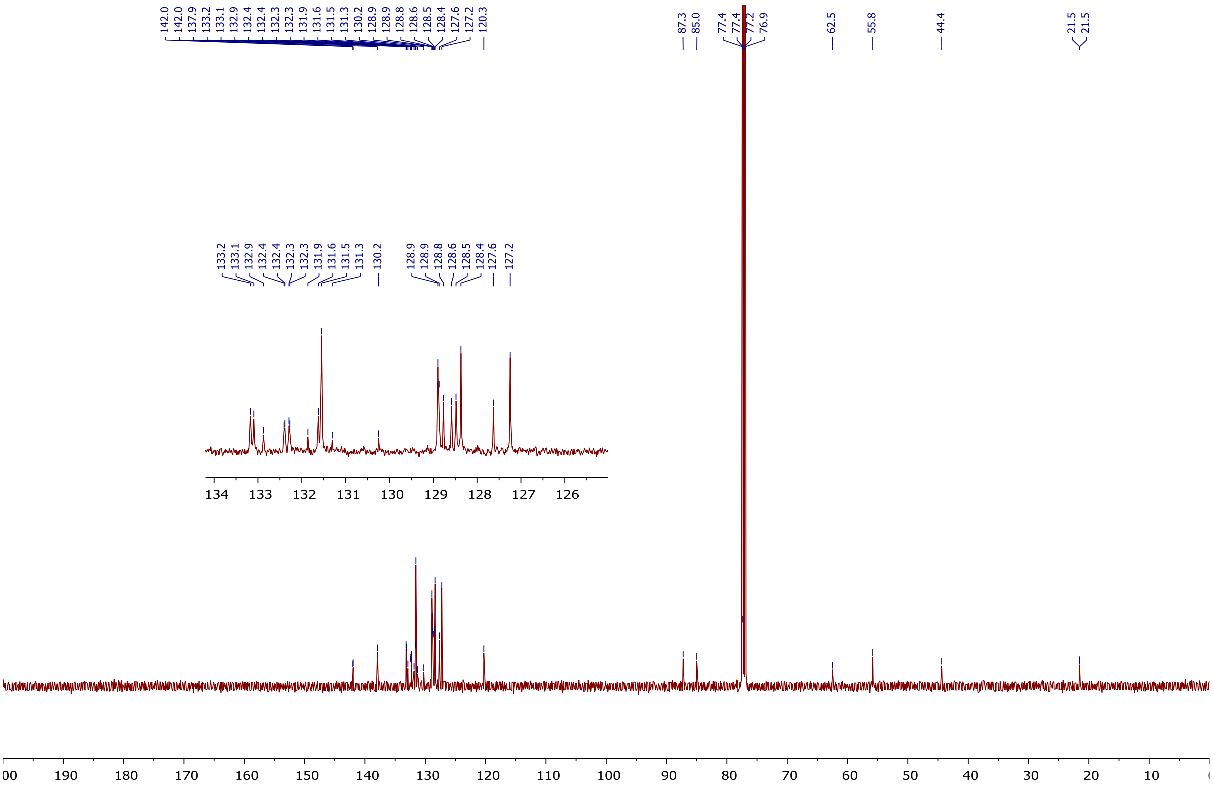

^{1}H NMR of **4c** (400 MHz, $CDCl_3$)

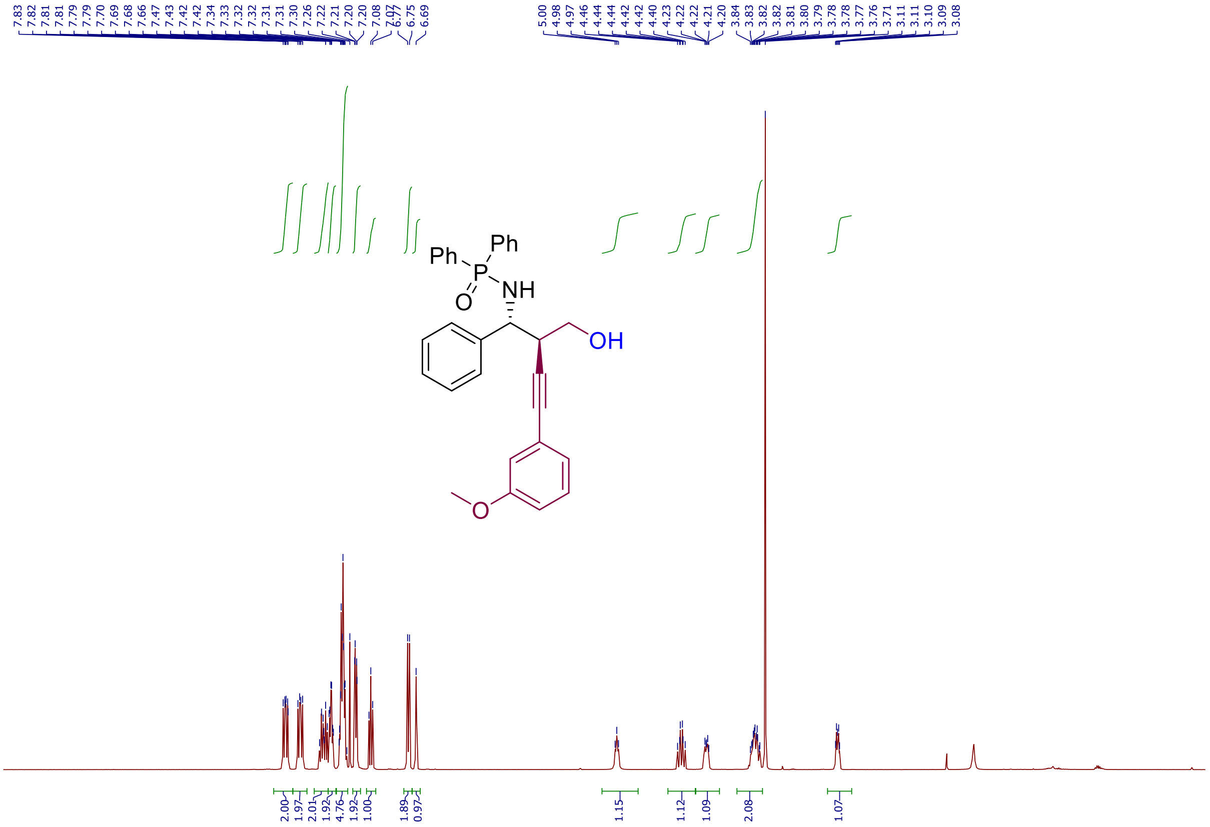

^{13}C NMR of **4c** (101 MHz, $CDCl_3$)

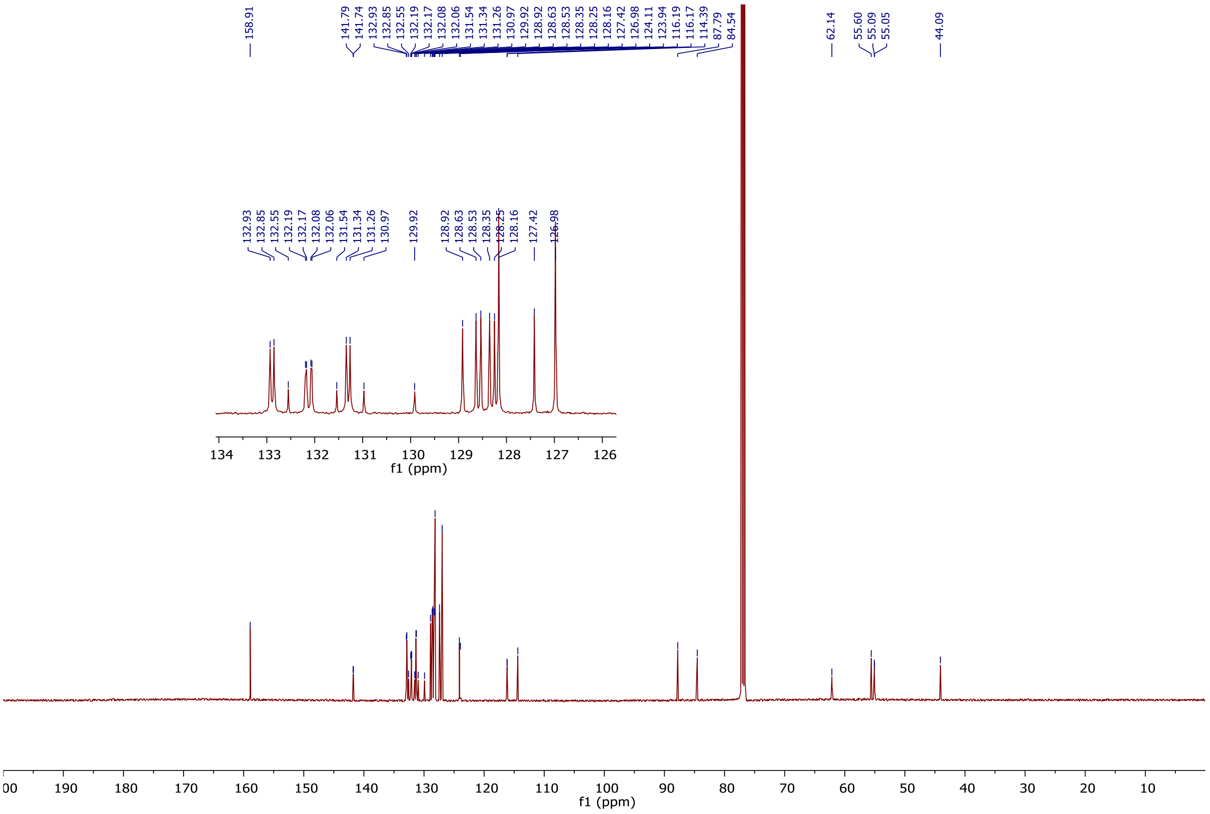

^{1}H NMR of **4d** (400 MHz, $CDCl_3$)

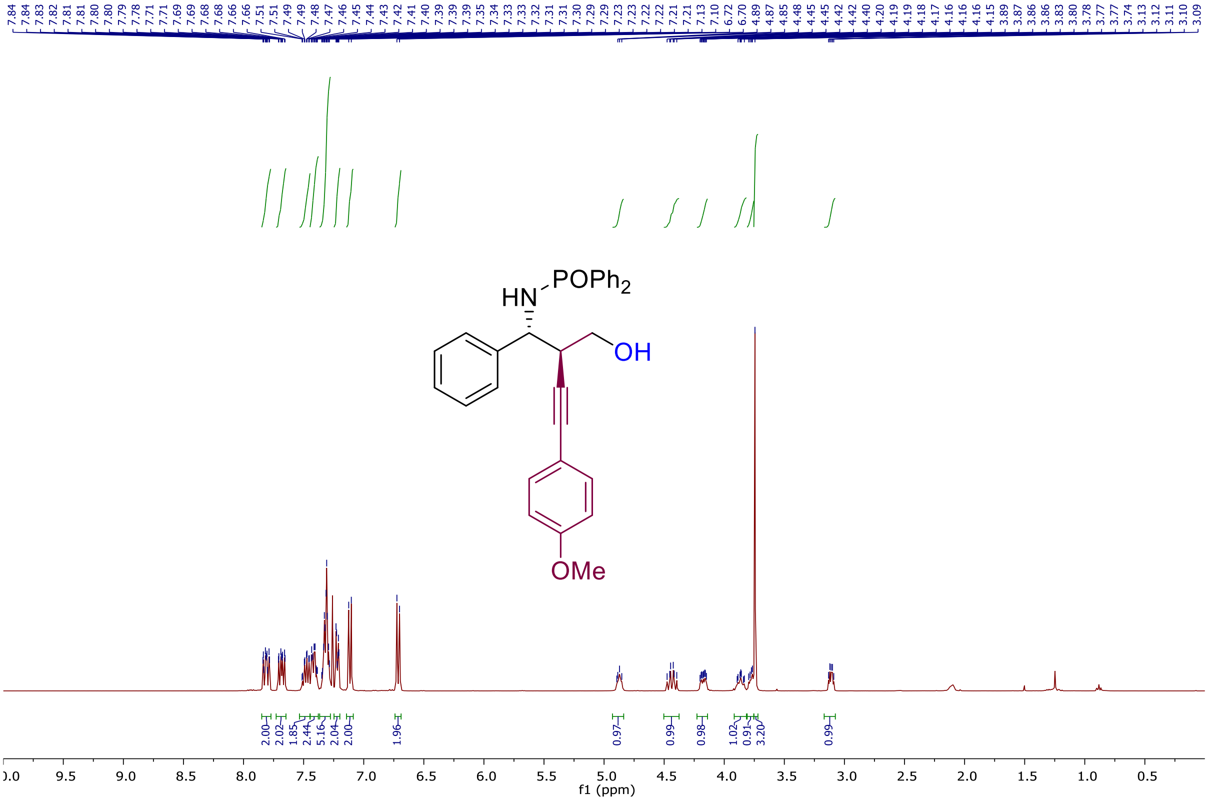

^{13}C NMR of **4d** (101 MHz, $CDCl_3$)

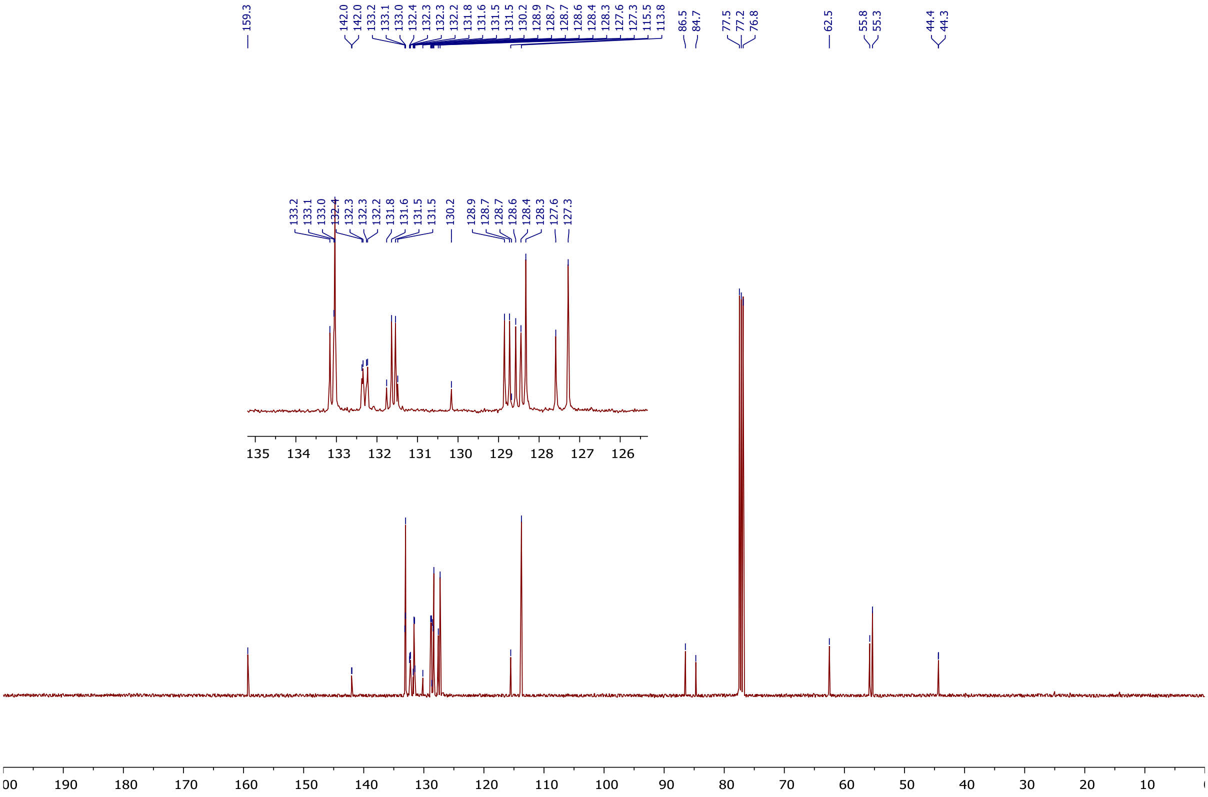

^{1}H NMR of **4e** (400 MHz, $CDCl_3$)

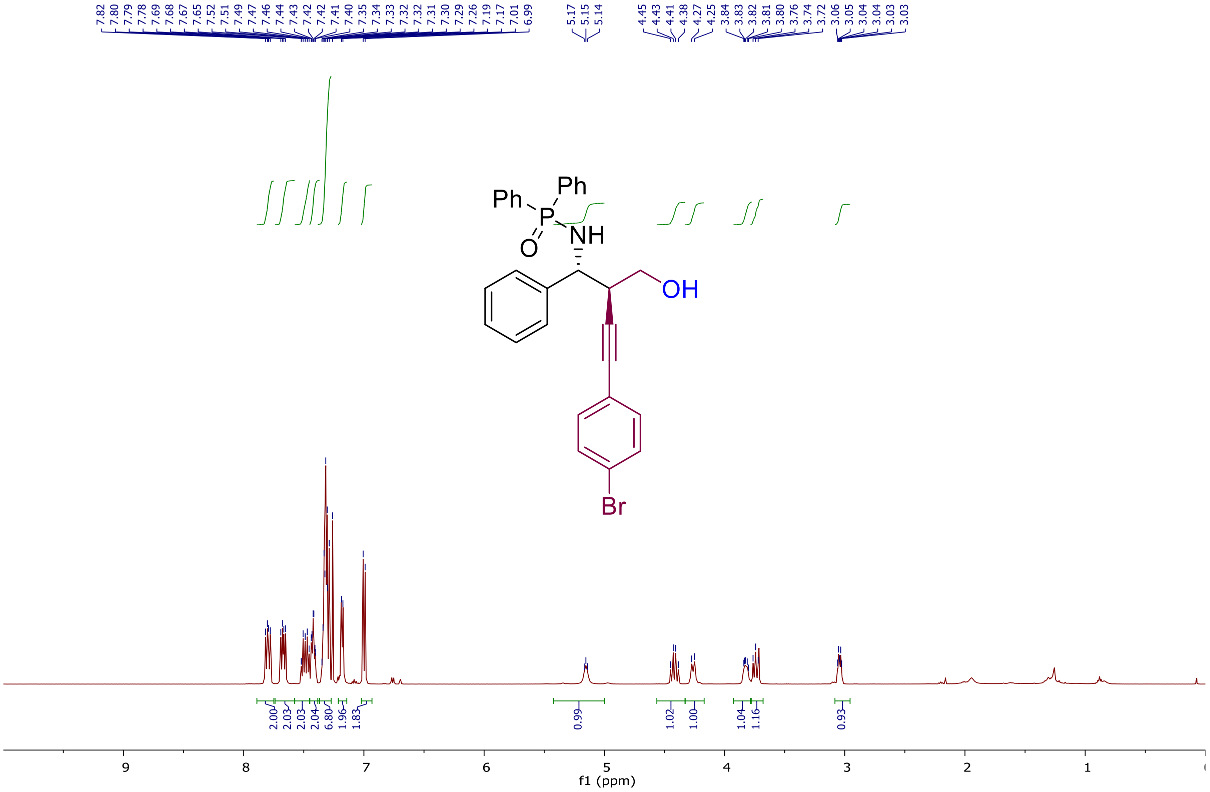

^{13}C NMR of **4c** (101 MHz, $CDCl_3$)

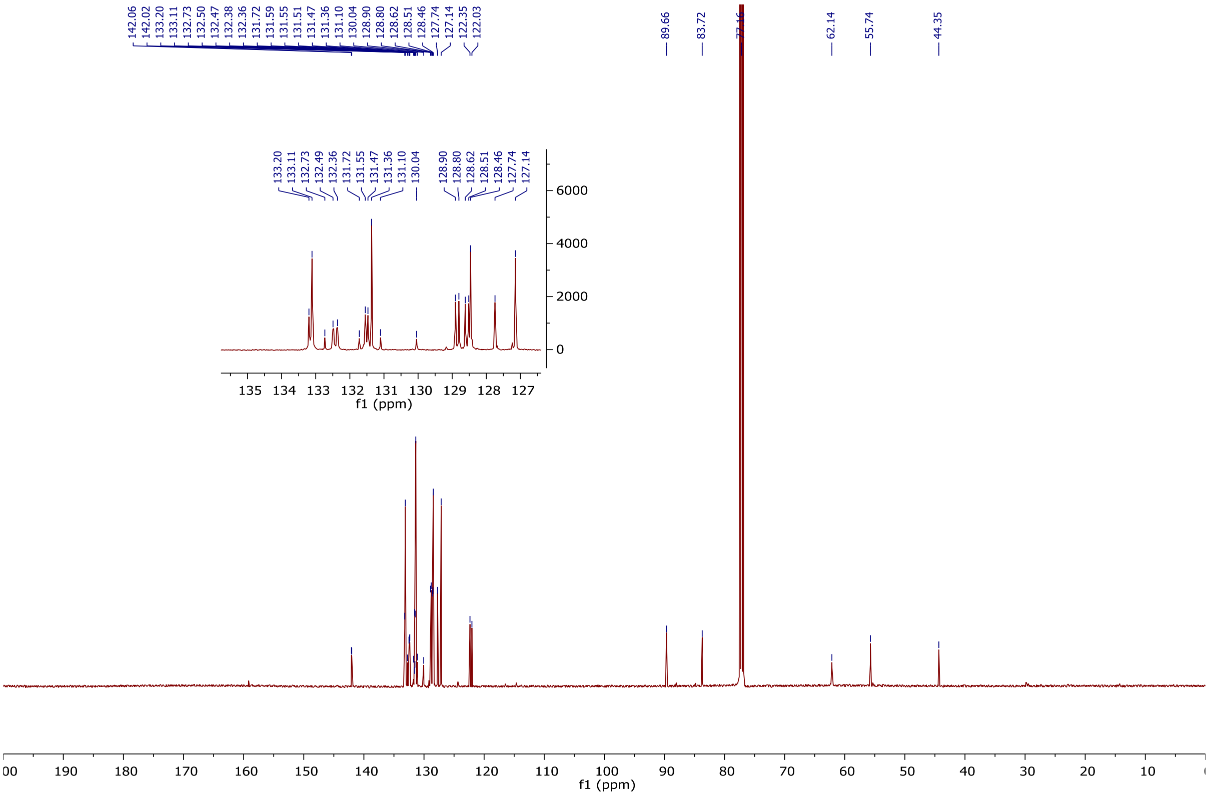

^{1}H NMR of **4f** (500 MHz, $CDCl_3$)

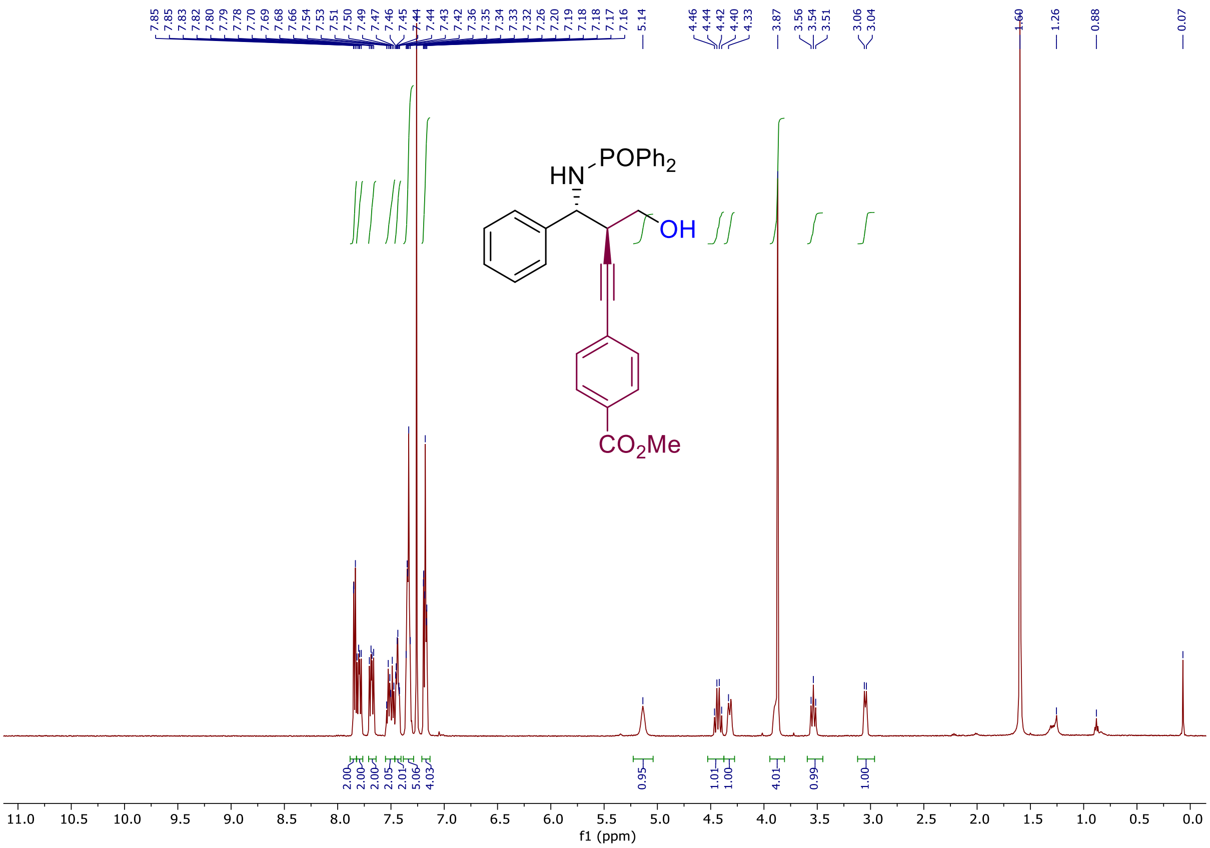

^{13}C NMR of **4f** (126 MHz, $CDCl_3$)

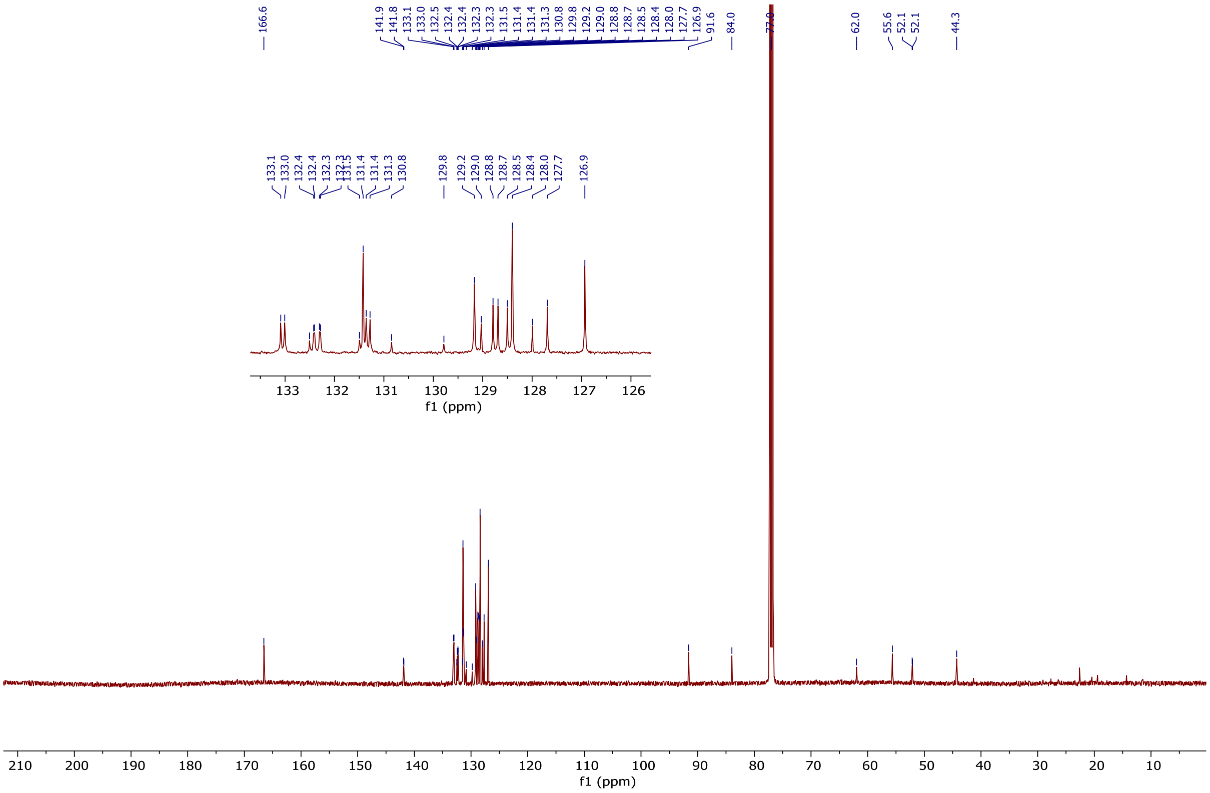

^{1}H NMR of **4g** (500 MHz, DMSO-*d*6)

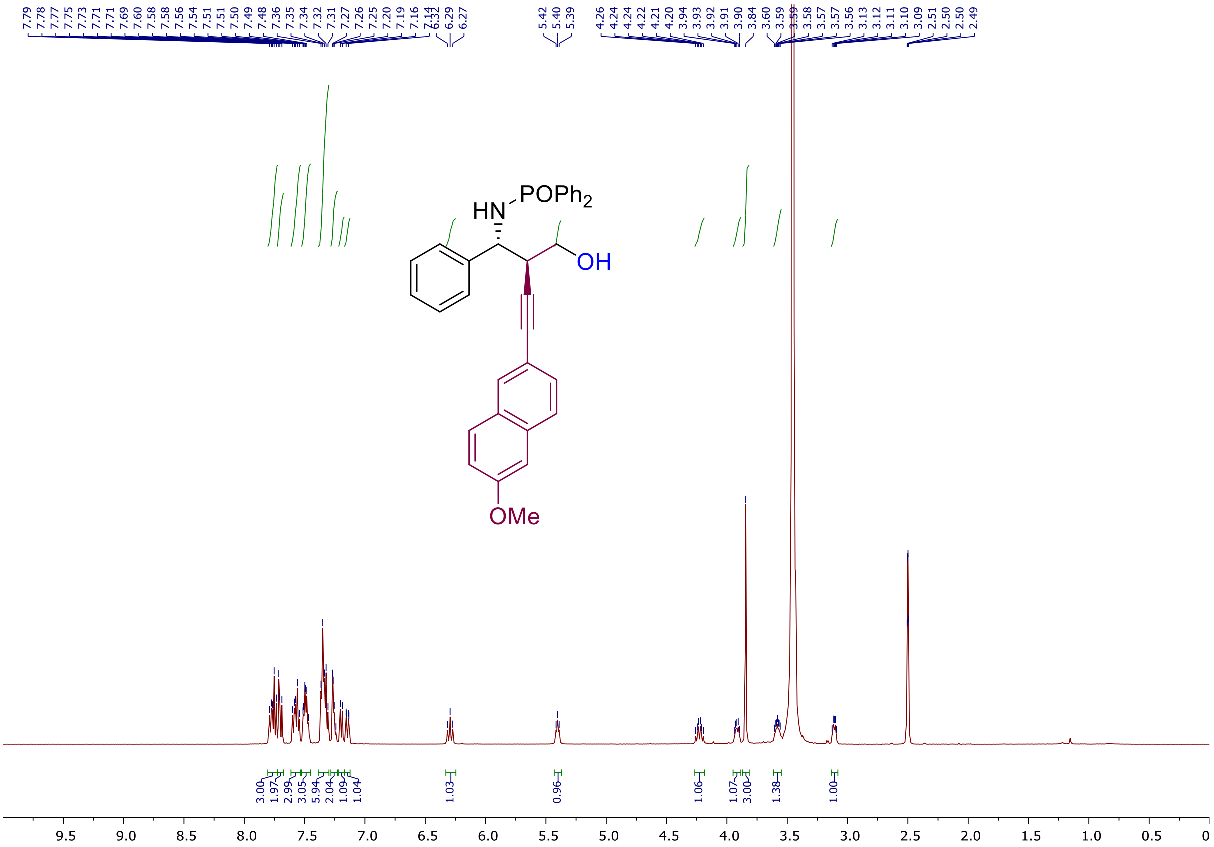

^{13}C NMR of **4g** (126 MHz, DMSO-*d*6)

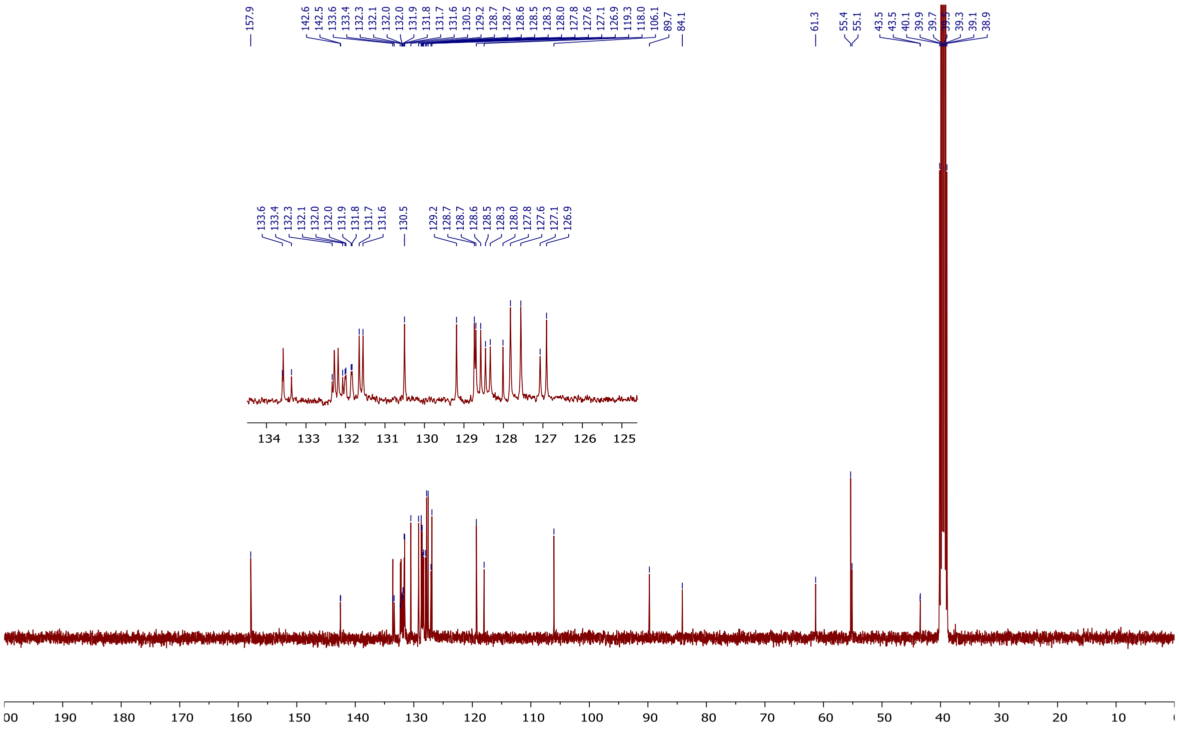

^{1}H NMR of **4h** (500 MHz, $CDCl_3$)

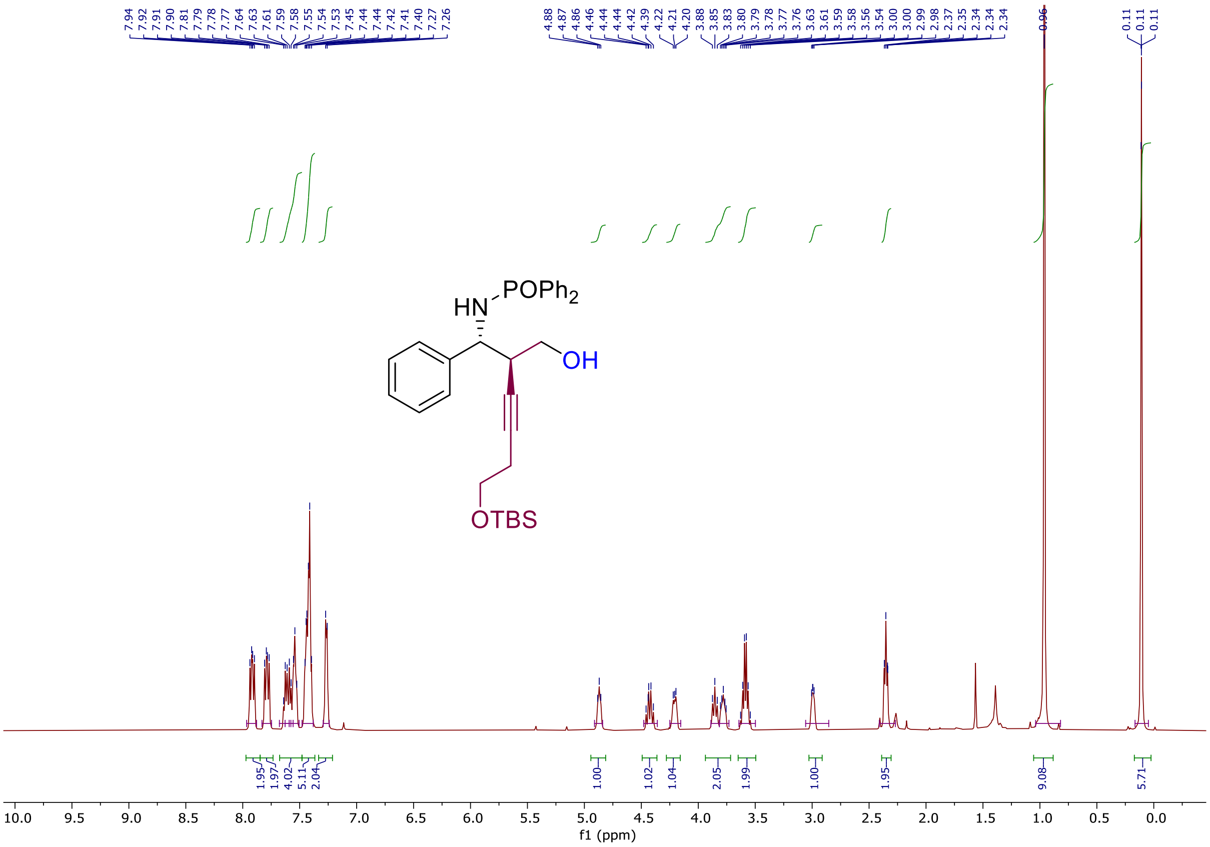

^{13}C NMR of **4h** (126 MHz, $CDCl_3$)

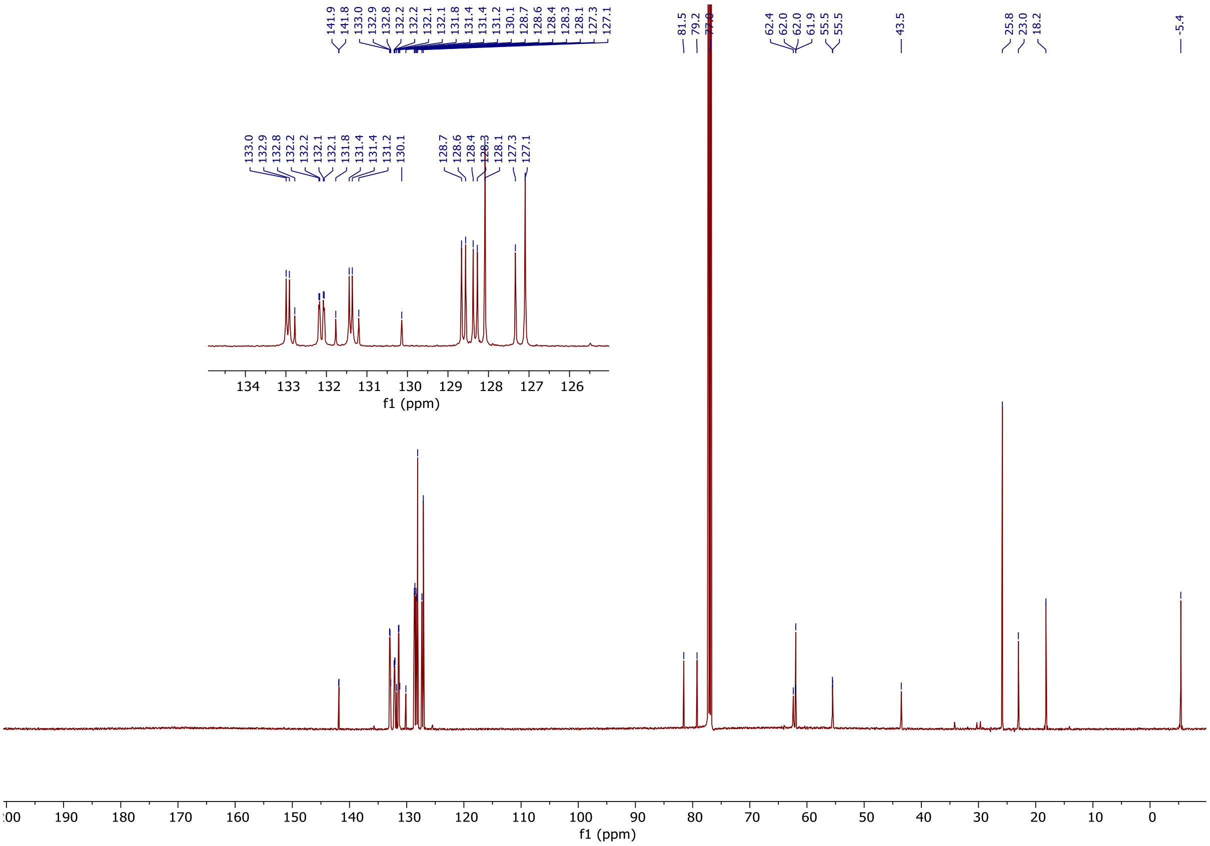

^{1}H NMR of **6a** (400 MHz, Acetone-*d*6)

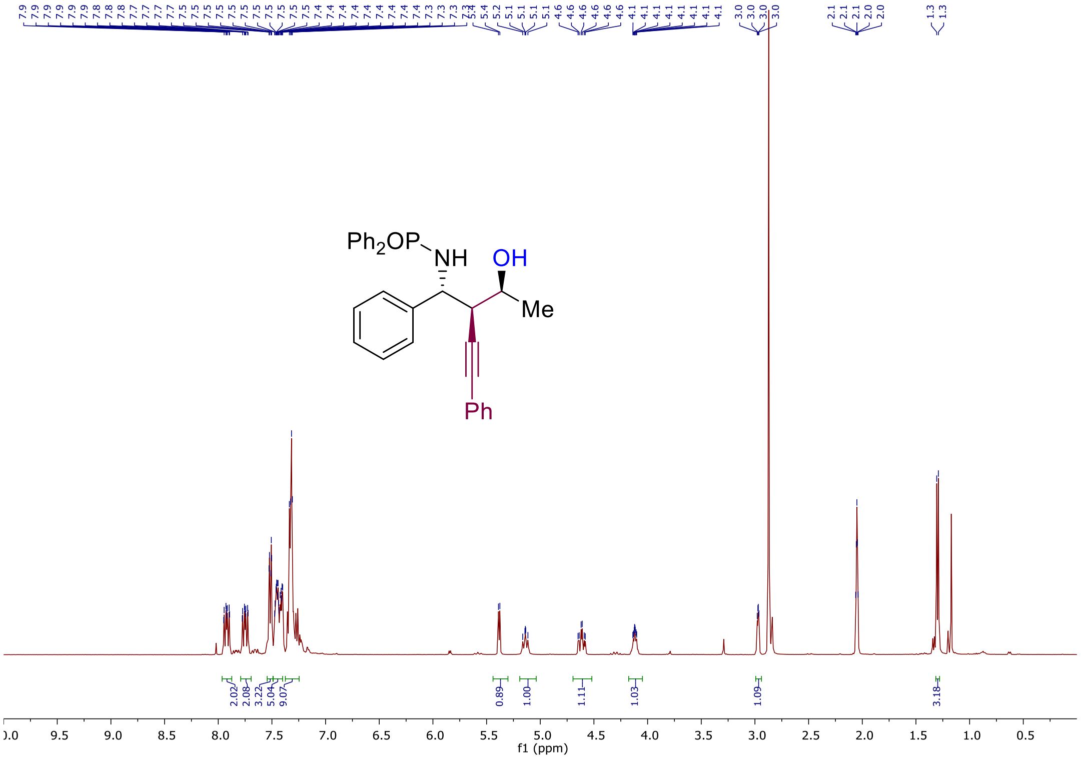

^{13}C NMR of **6a** (101 MHz, Acetone-*d*6)

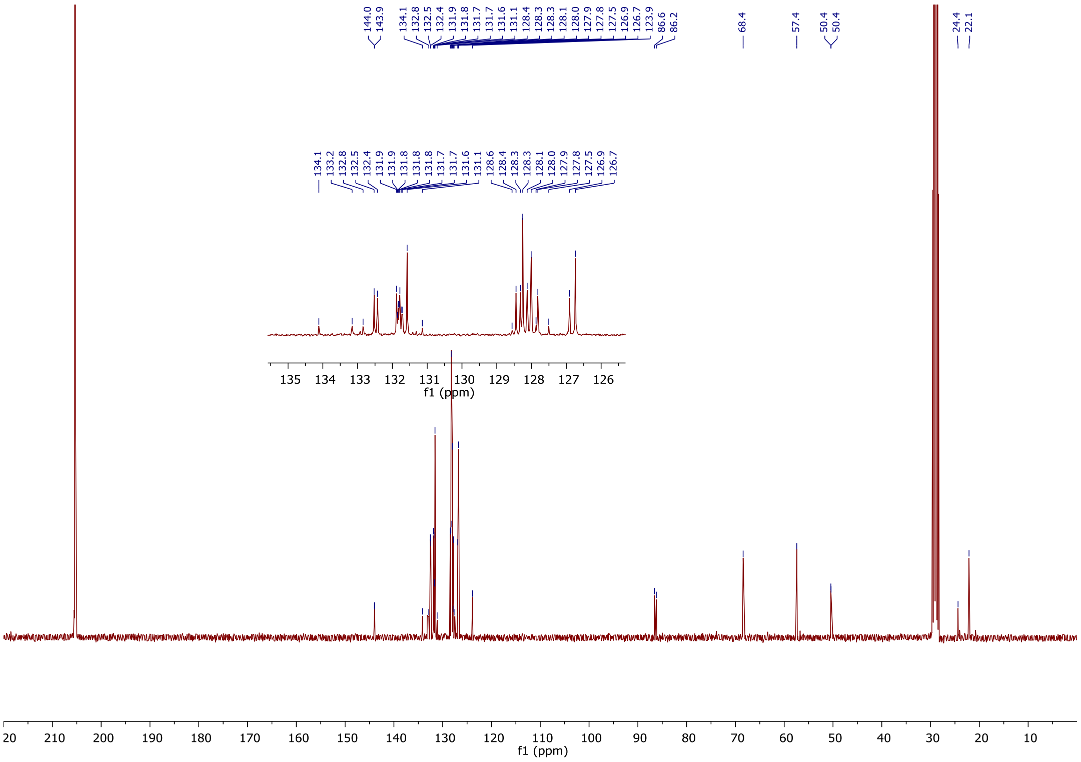

^{1}H NMR of **6b** (400 MHz, Acetone-*d6*)

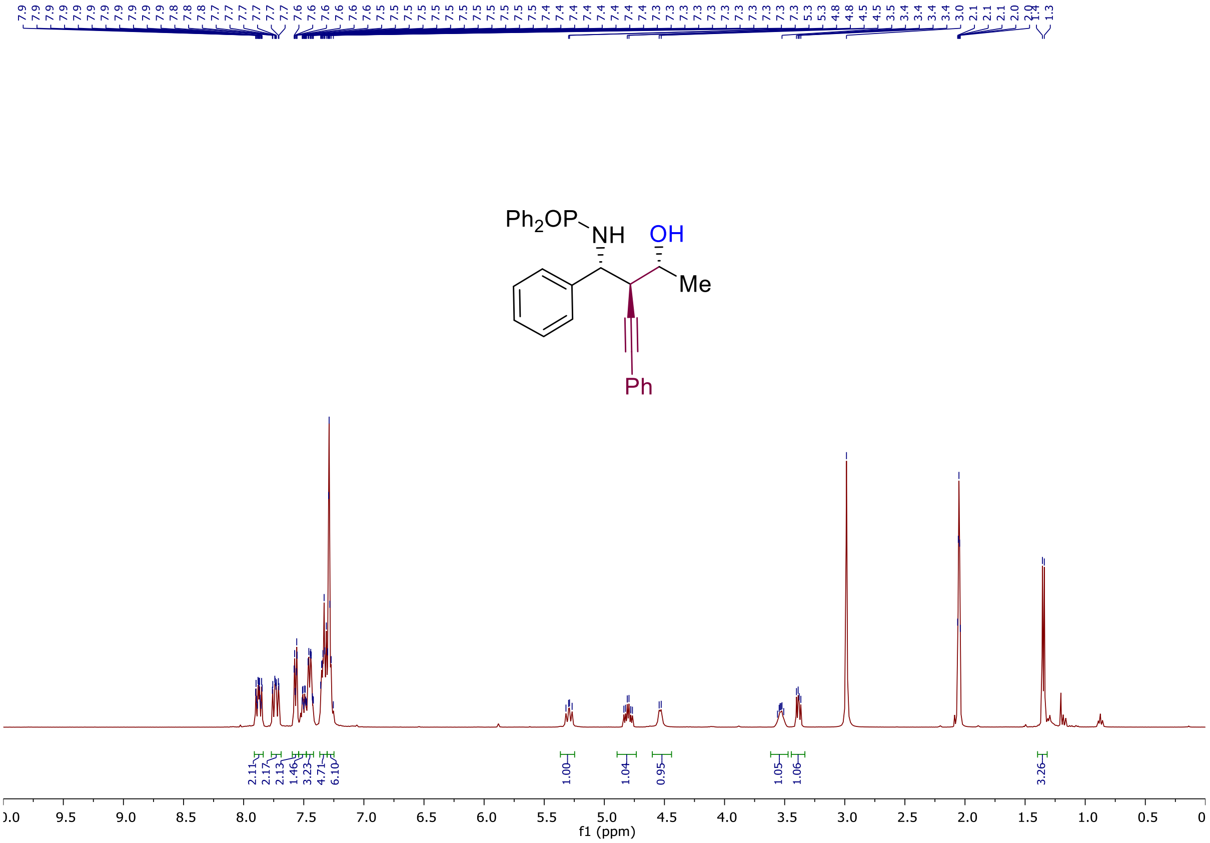

^{13}C NMR of **6b** (101 MHz, Acetone-*d6*)

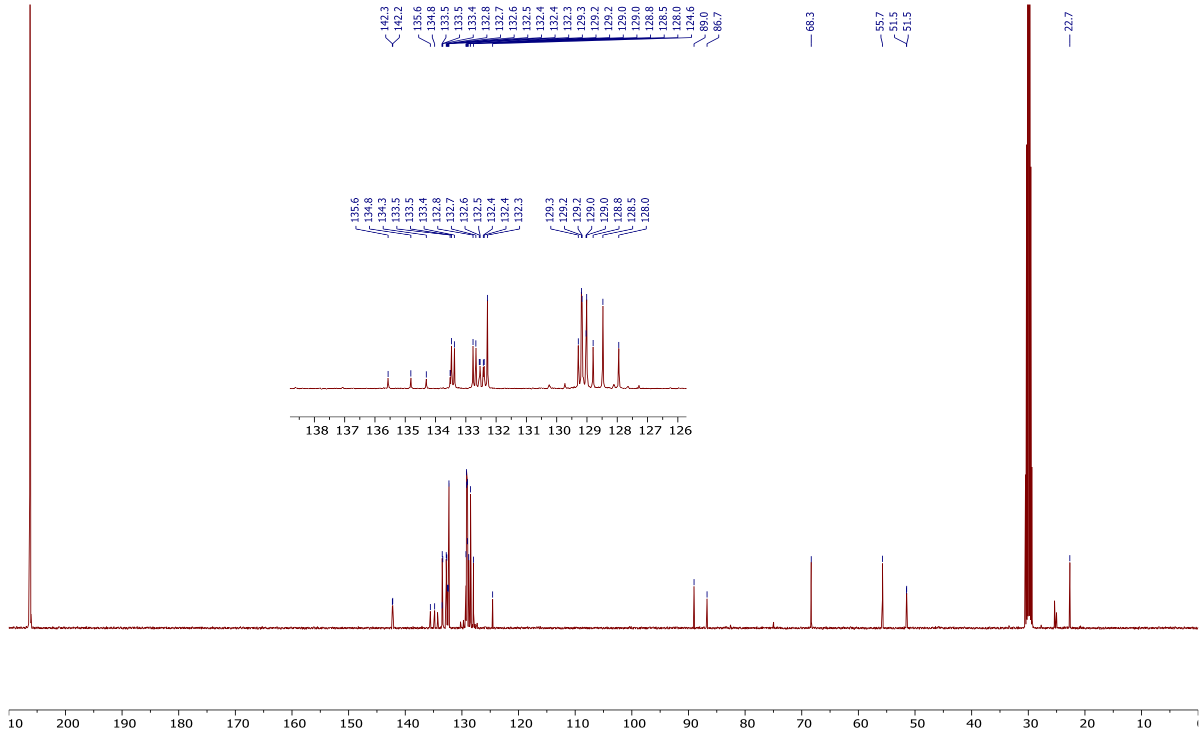

^{1}H NMR of **6c** (400 MHz, Acetone-*d*6)

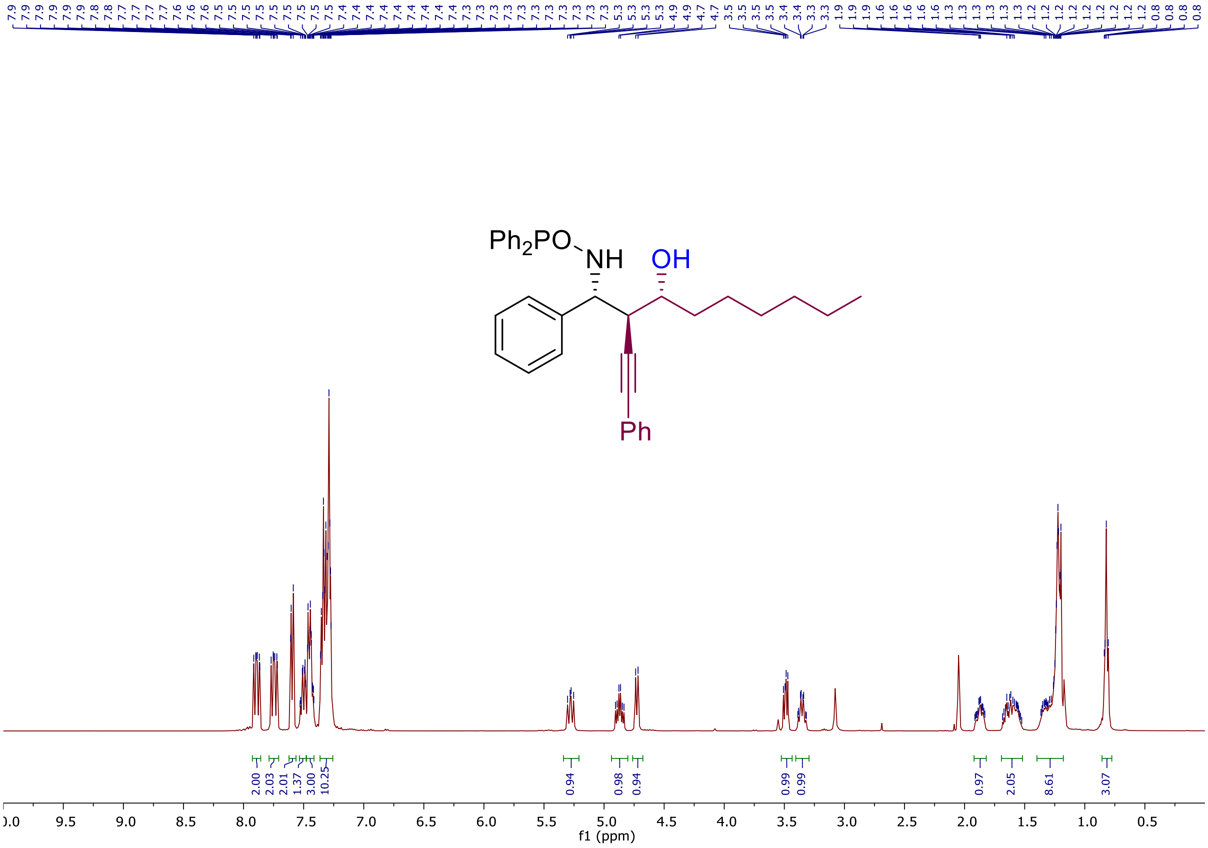

^{13}C NMR of **6c** (101 MHz, Acetone-*d*6)

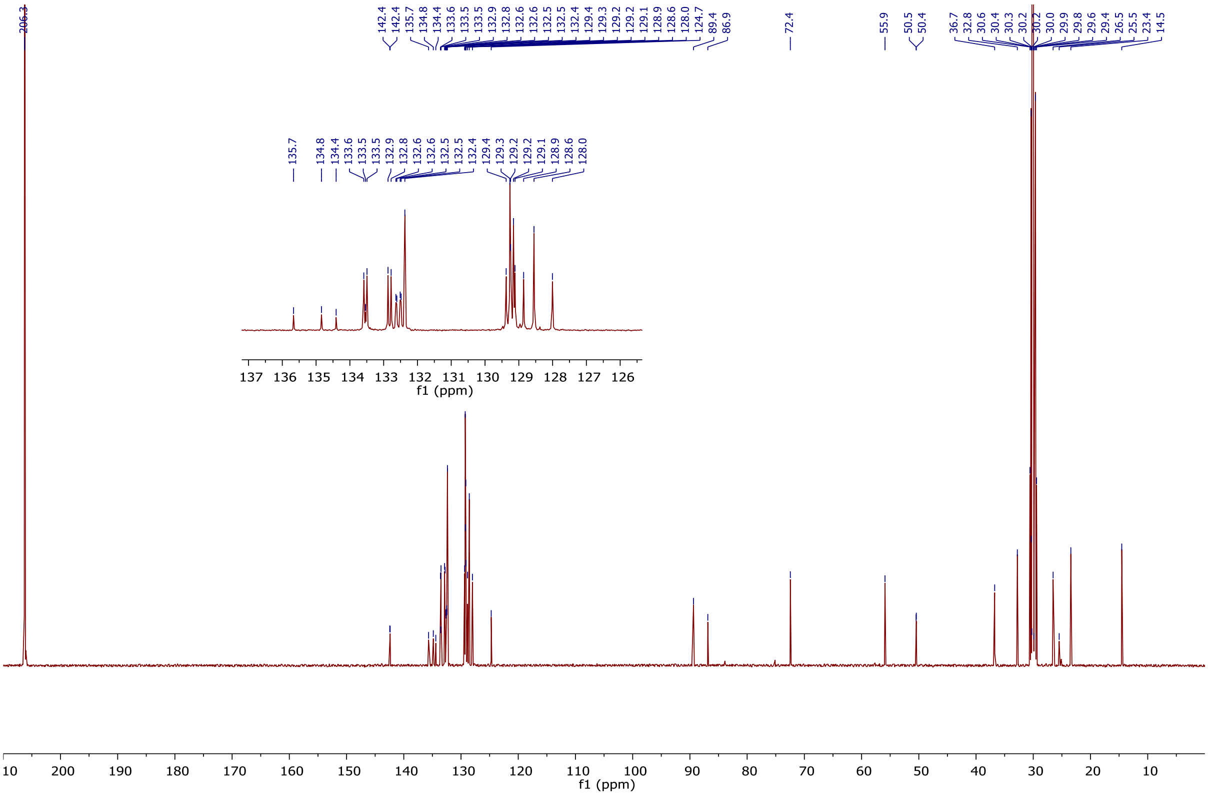

^{1}H NMR of **6d** (500 MHz, $CDCl_3$)

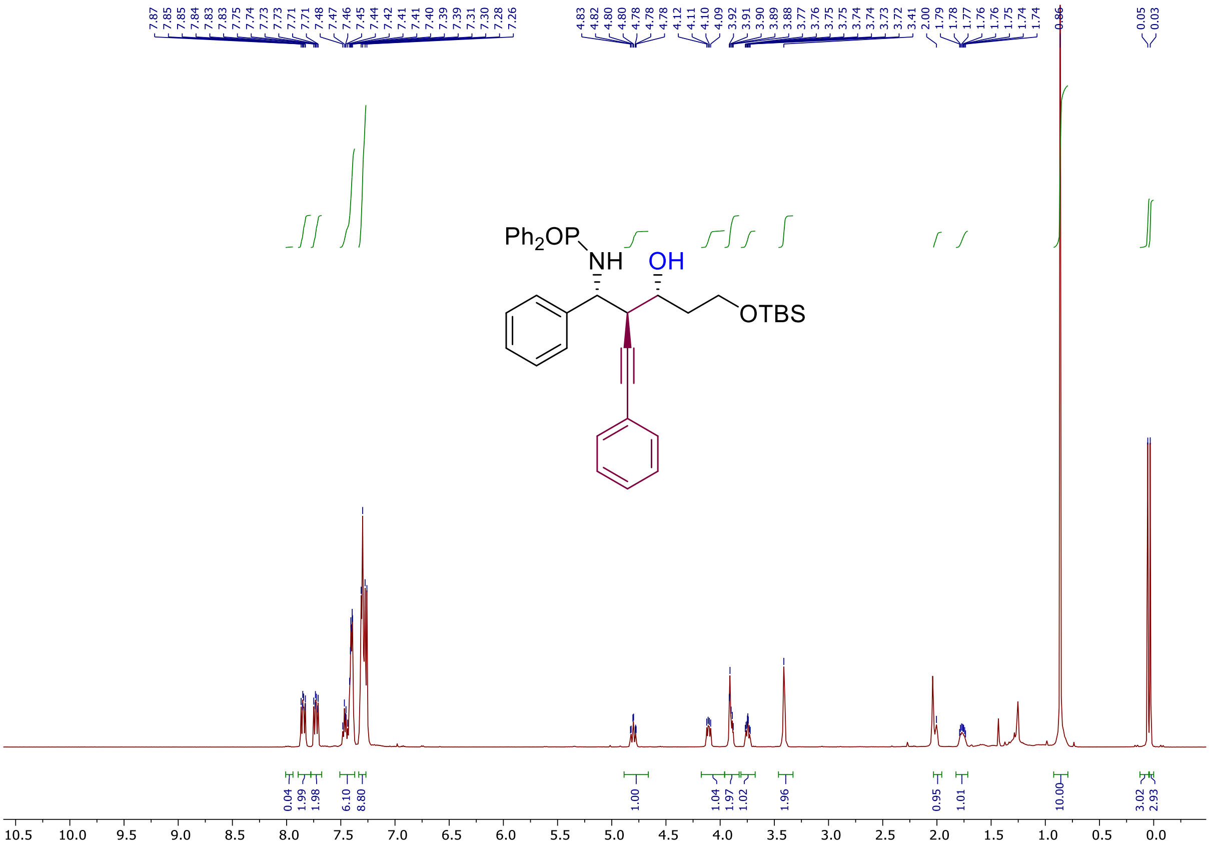

^{13}C NMR of **6d** (126 MHz, $CDCl_3$)

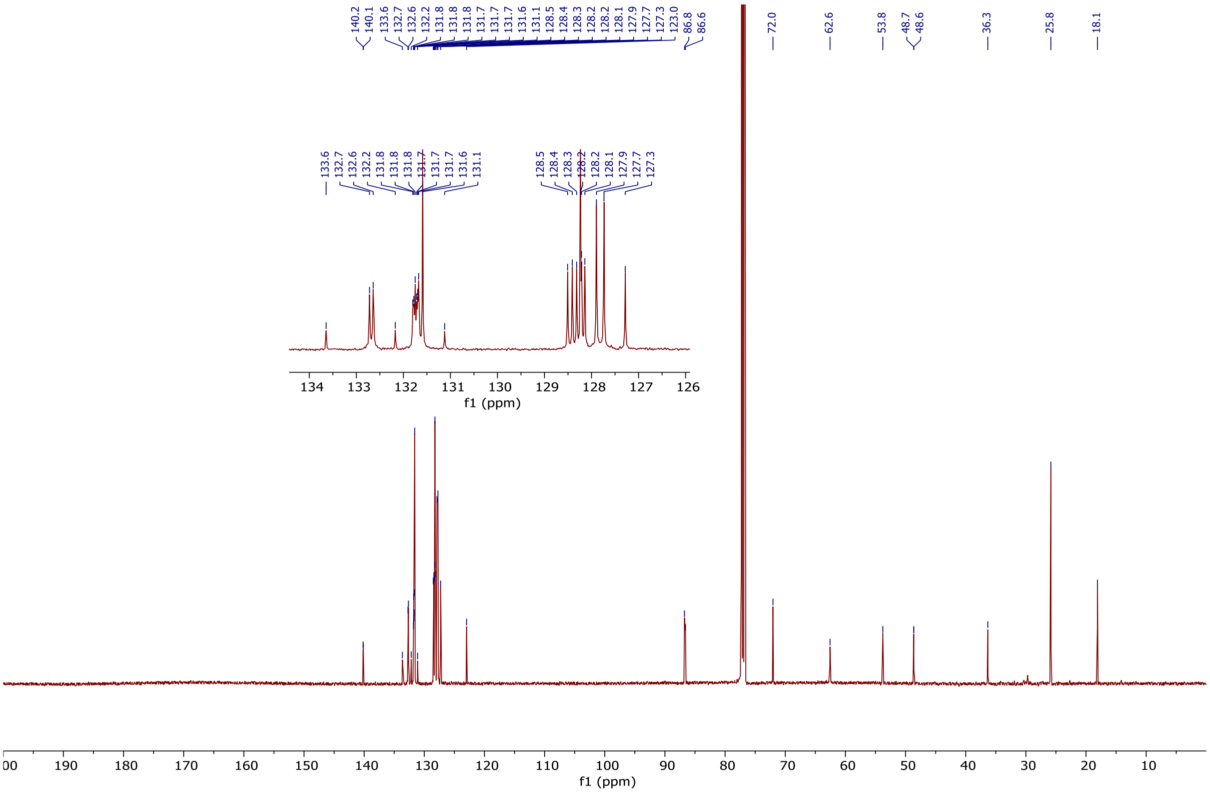

^{1}H NMR of **7a** (400 MHz, $CDCl_3$)

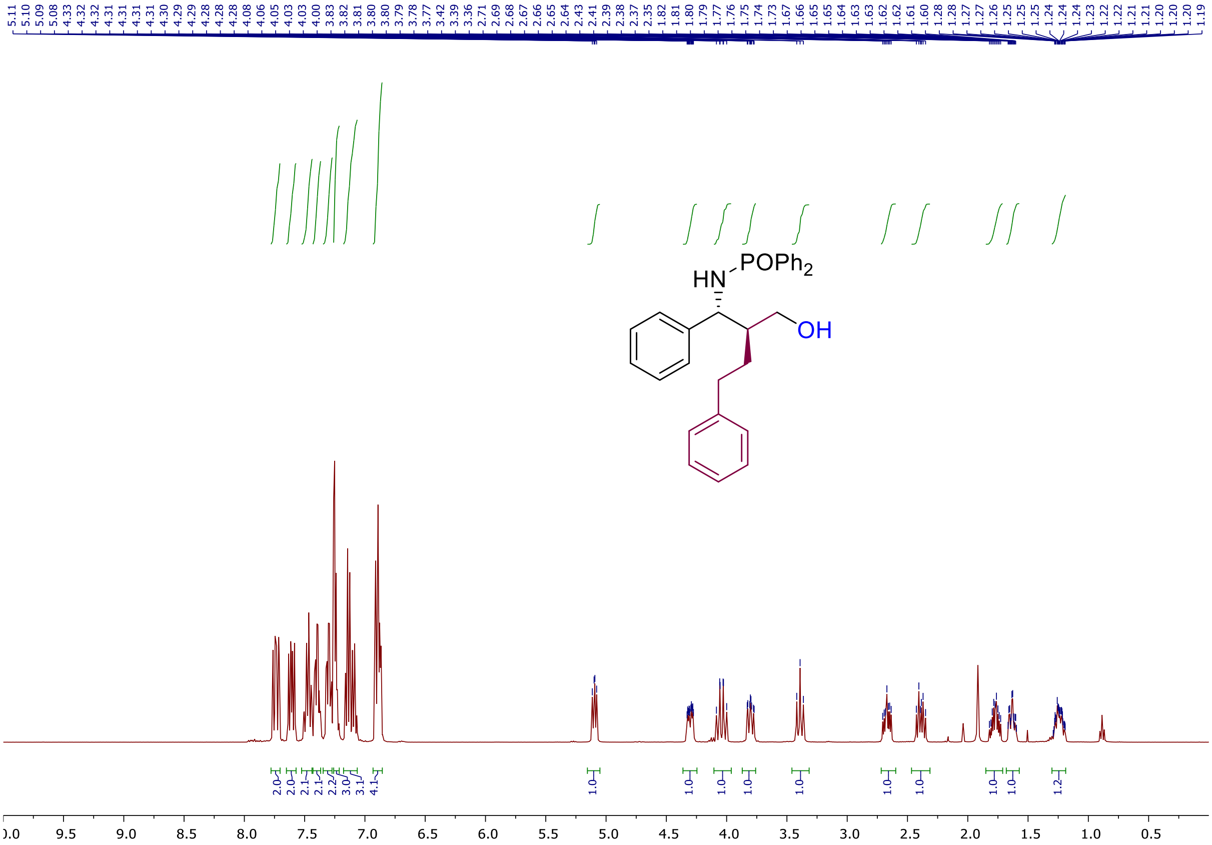

^{13}C NMR of **7a** (101 MHz, $CDCl_3$)

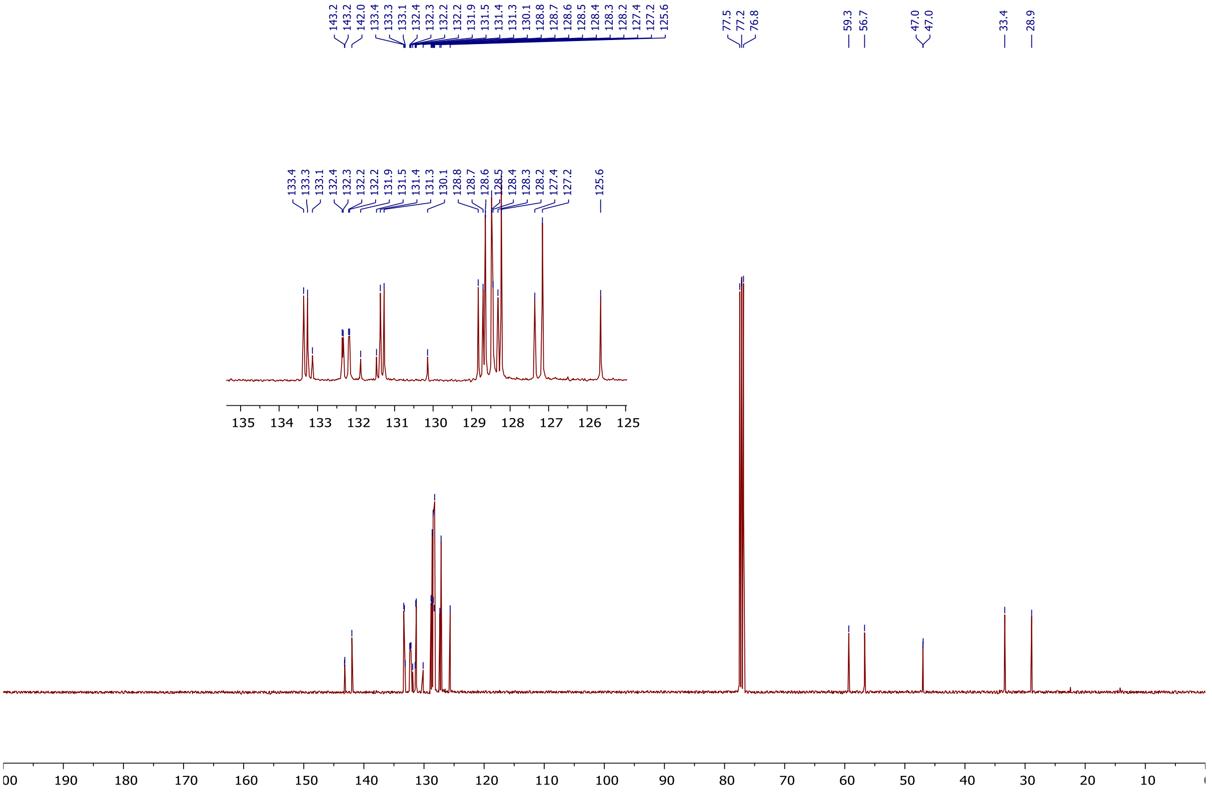

^{1}H NMR of **7b** (400 MHz, CD_3OD)

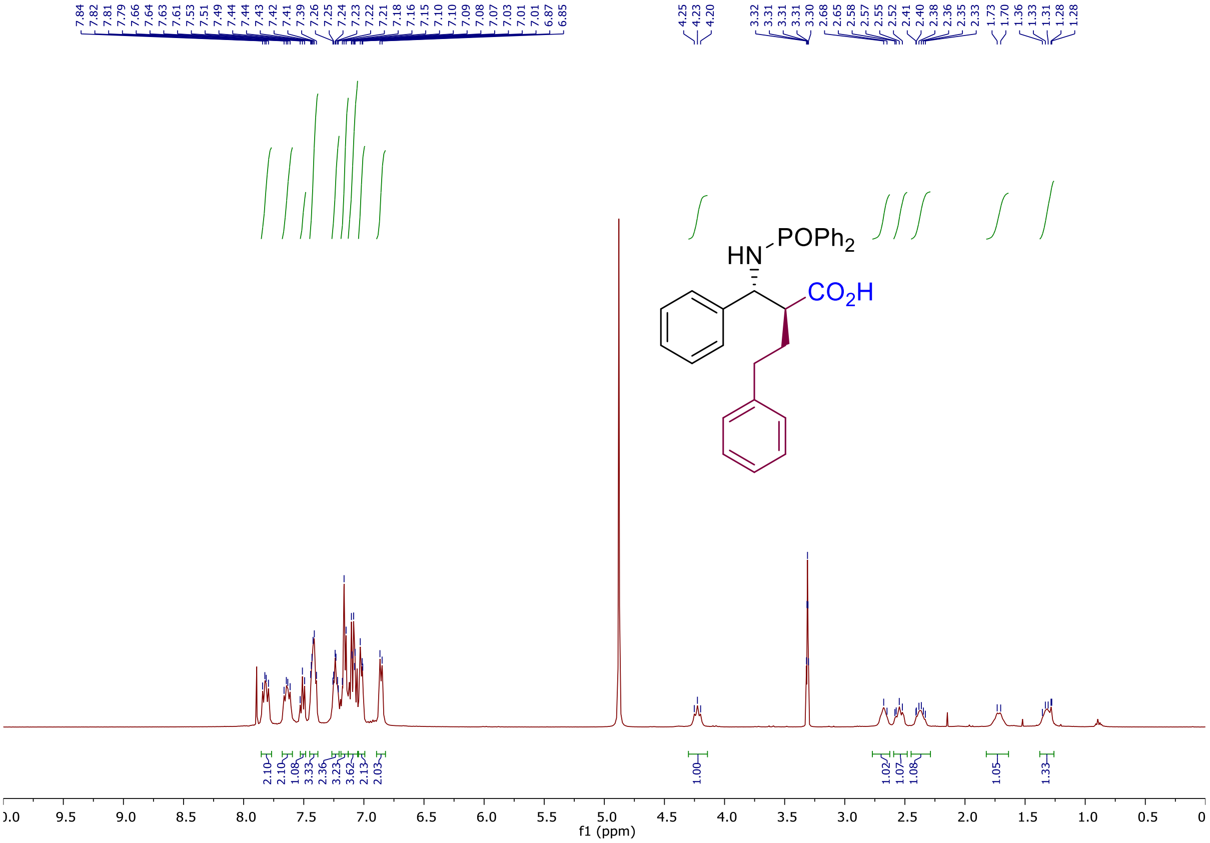

^{13}C NMR of **7b** (101 MHz, CD_3OD)

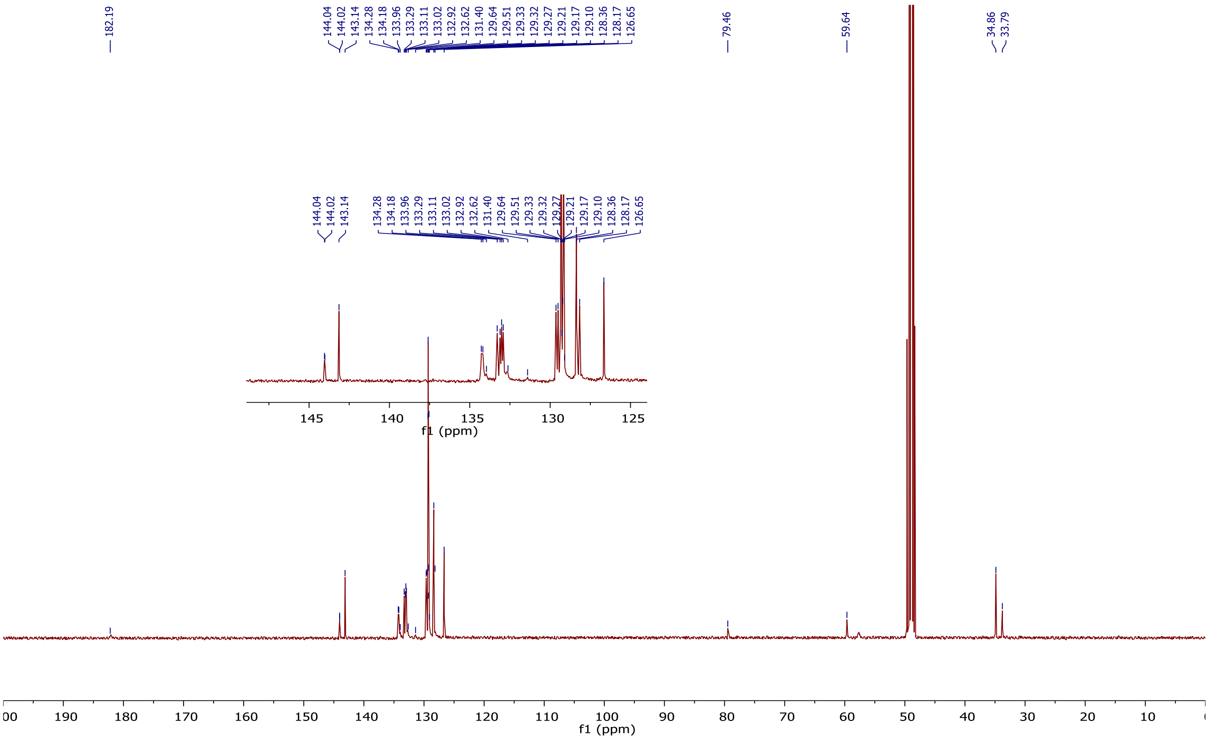

^{1}H NMR of **7c** (400 MHz, $CDCl_3$)

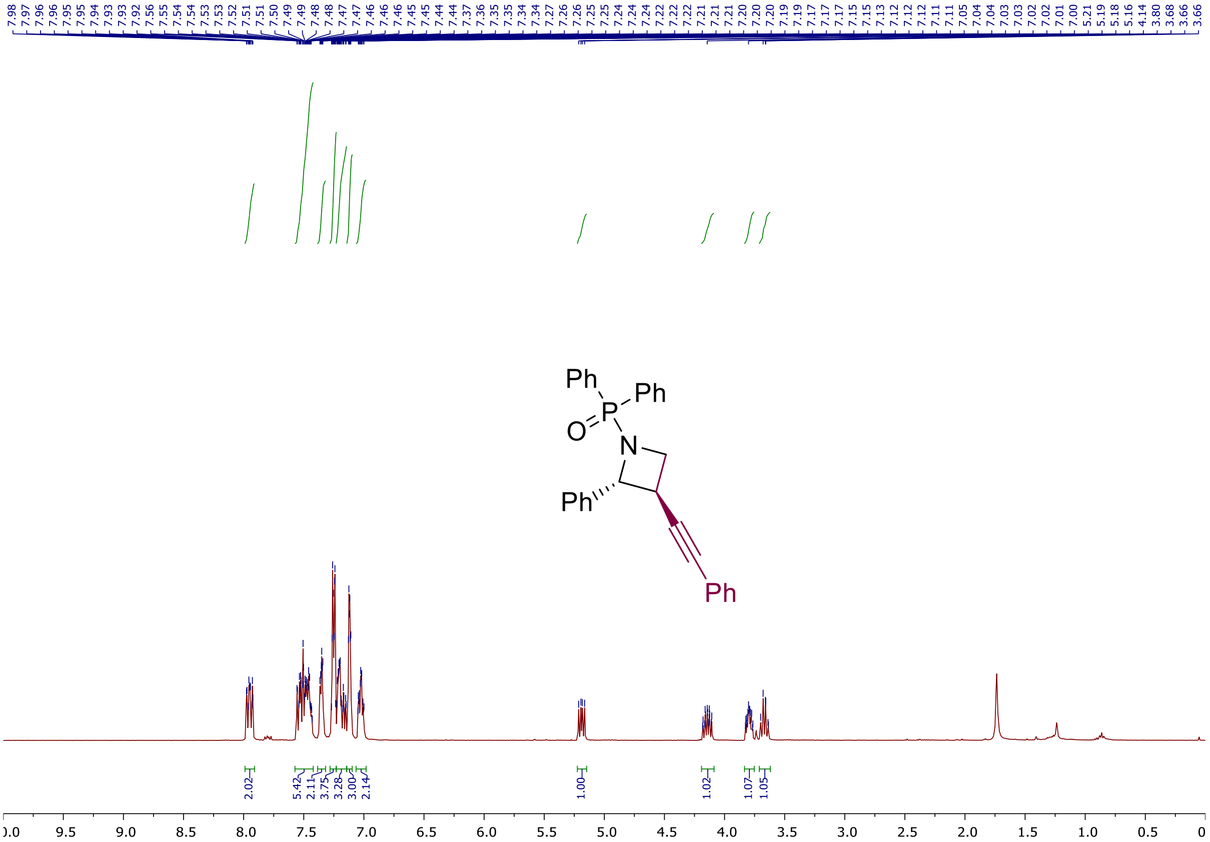

^{13}C NMR of **7c** (101 MHz, $CDCl_3$)

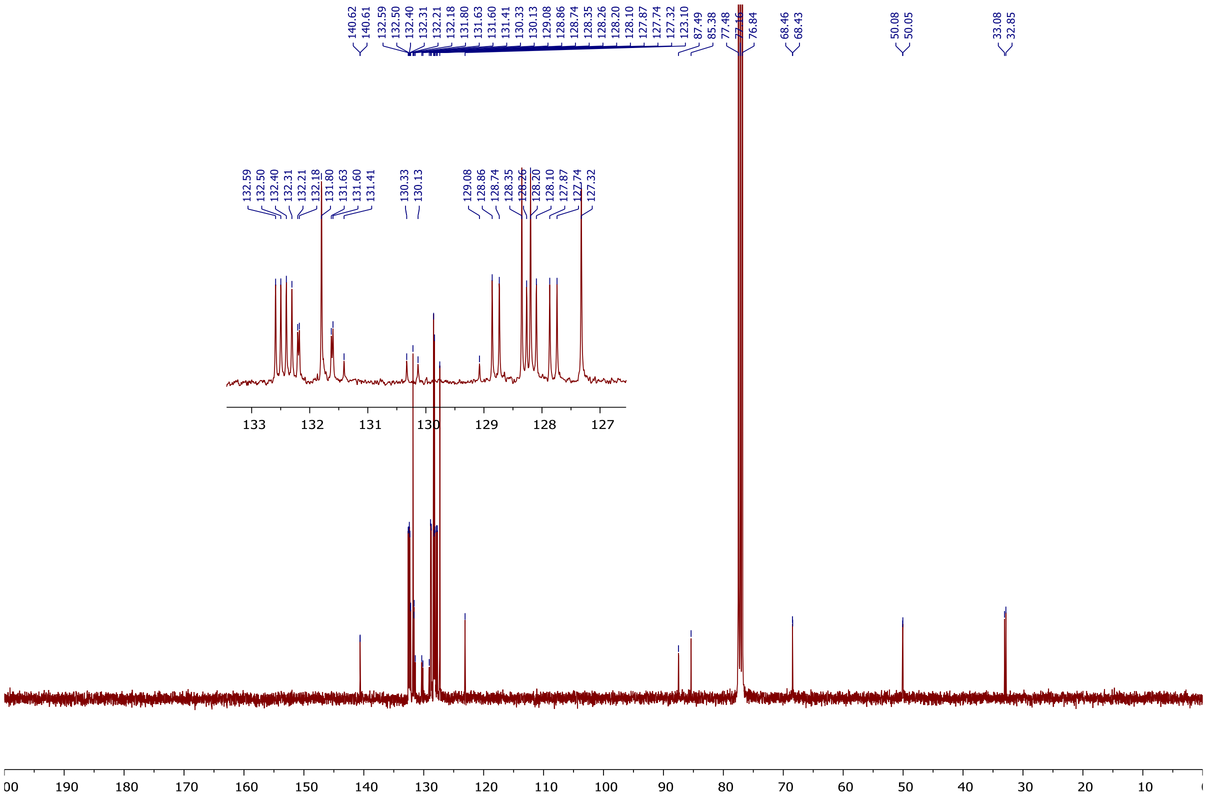

^{1}H NMR of **7d** and **7d'** (500 MHz, $CDCl_3$) **(4:1)**

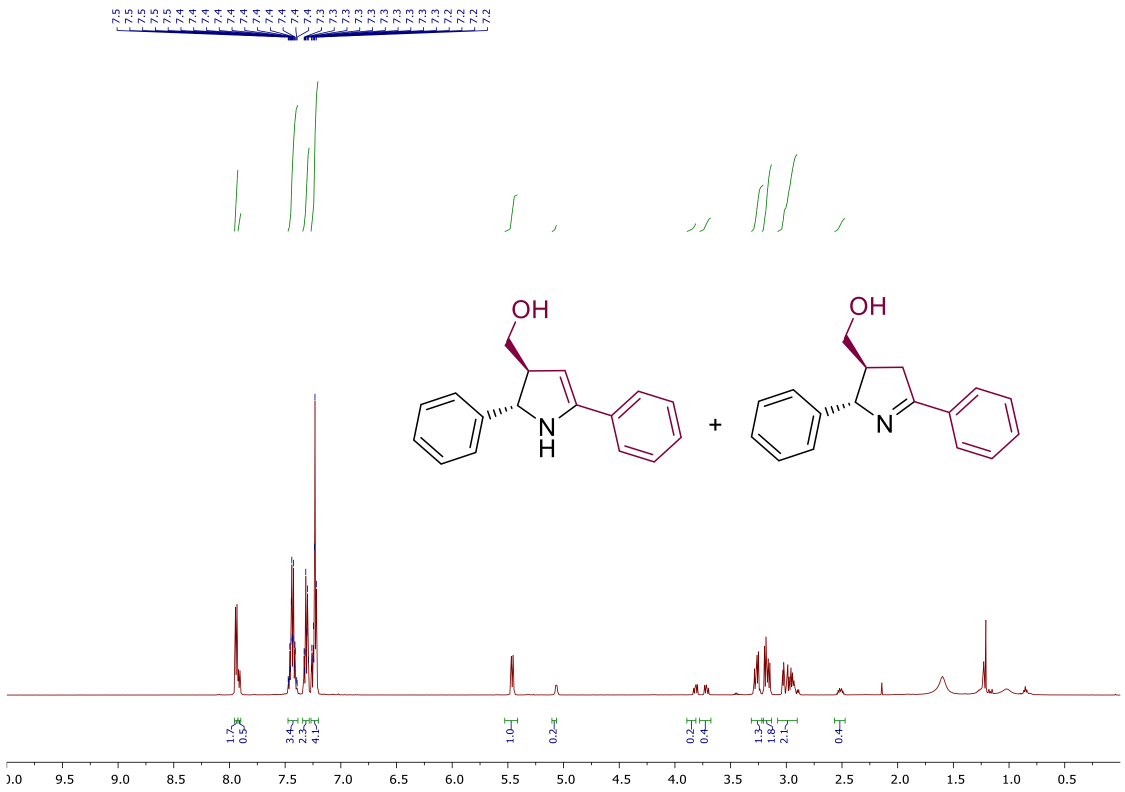

^{1}H NMR of **7d** and **7d'** (126 MHz, $CDCl_3$) **(4:1)**

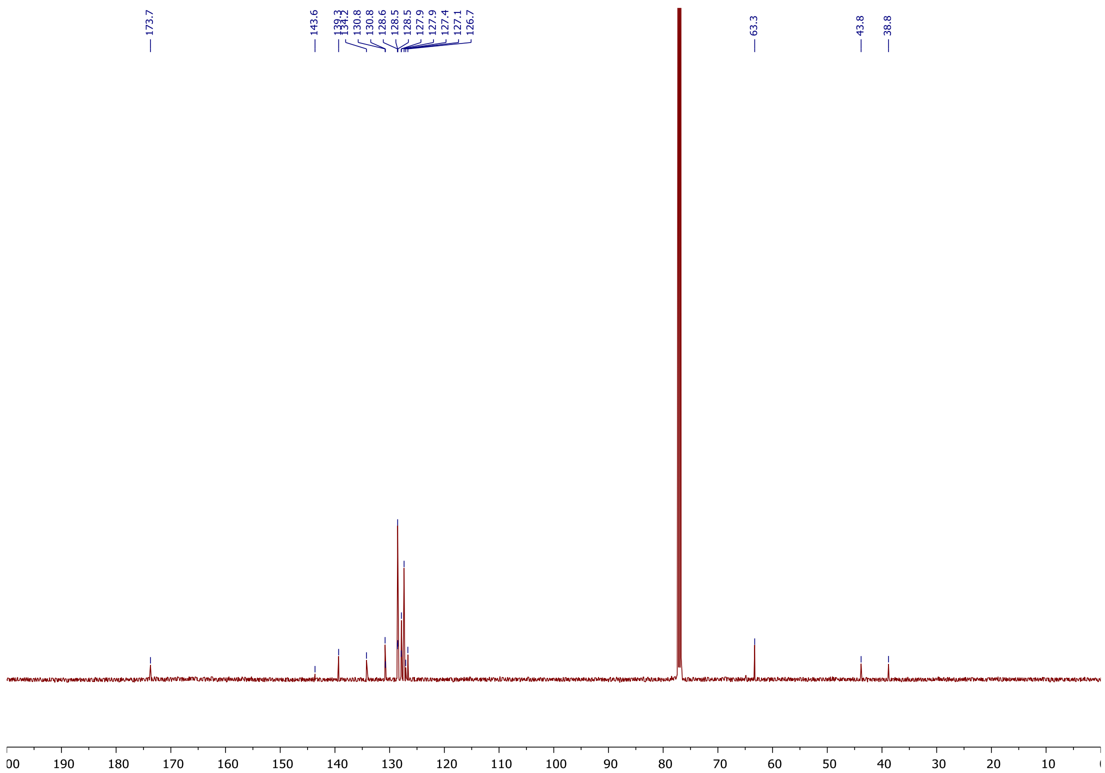

^{1}H NMR of **7f** (400 MHz, $CDCl_3$)

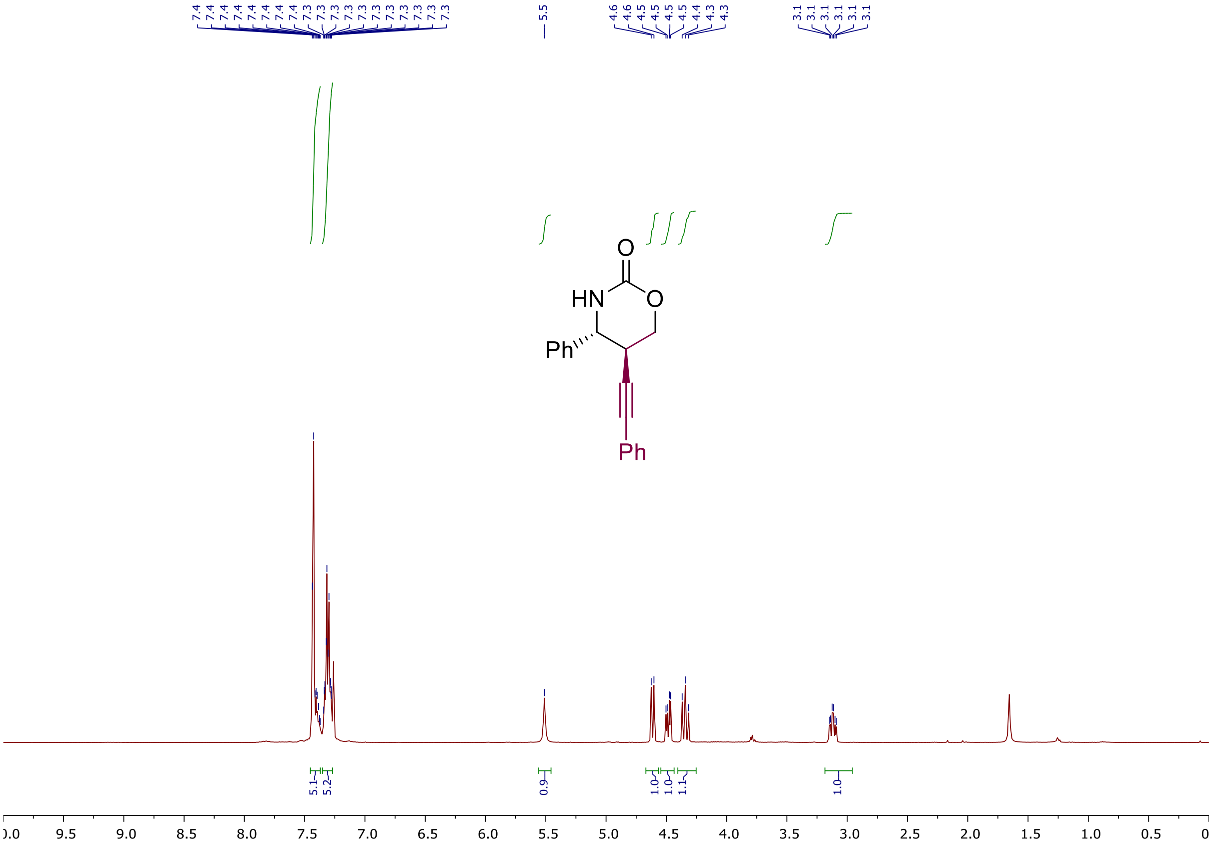

^{13}C NMR of **7f** (101 MHz, $CDCl_3$)

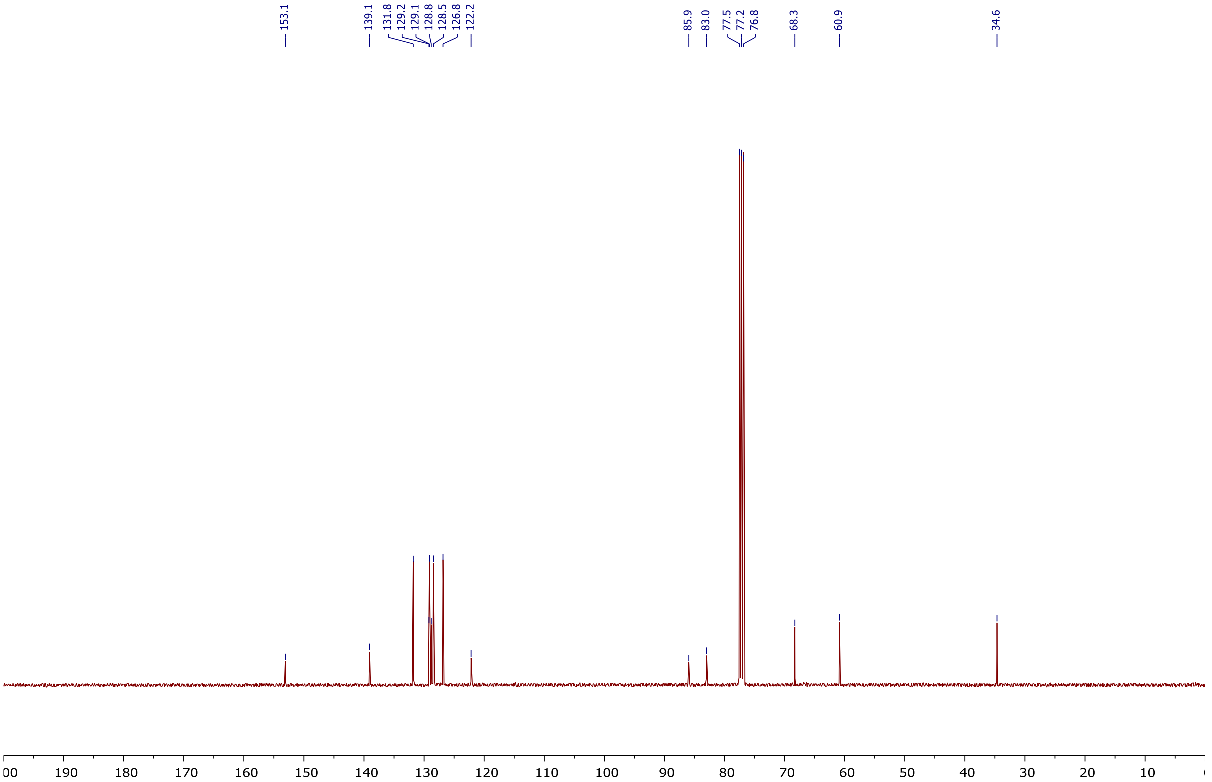

X-Ray structures

Single crystal structure of **3d**

CCDC No = 1952974

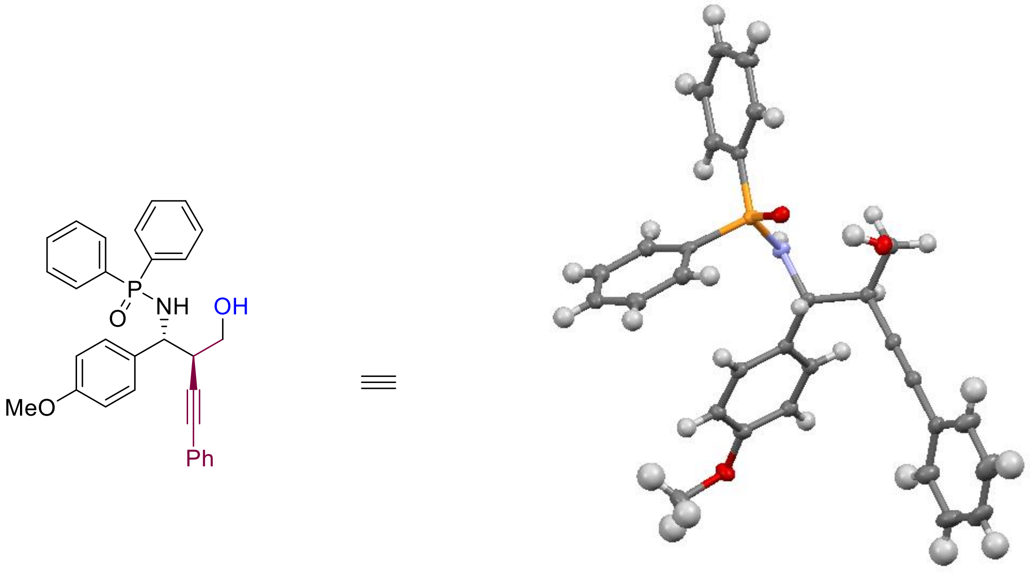

Bond precision: C-C = 0.0036 A Wavelength=1.54184

Cell: a=5.45044(10) b=18.2698(4) c=24.6591(5)

alpha=90 beta=90 gamma=90

Temperature: 100 K

Calculated Reported

Volume 2455.52(9) 2455.52(8)

Space group P 21 21 21 P 21 21 21

Hall group P 2ac 2ab P 2ac 2ab

Moiety formula C30 H28 N O3 P C30 H28 N O3 P

Sum formula C30 H28 N O3 P C30 H28 N O3 P

Mr 481.50 481.50

Dx,g cm-3 1.303 1.302

Z 4 4

Mu (mm-1) 1.251 1.251

F000 1016.0 1016.0

F000’ 1019.94

h,k,lmax 6,23,31 6,23,31

Nref 5179[3001] 5093

Tmin,Tmax 0.866,0.913 0.849,1.000

Tmin’ 0.862

Correction method= # Reported T Limits: Tmin=0.849 Tmax=1.000

AbsCorr = MULTI-SCAN

Data completeness= 1.70/0.98 Theta(max)= 76.932

R(reflections)= 0.0329(4893) wR2(reflections)= 0.0820(5093)

S = 1.093 Npar= 317

Single crystal structure of **6b**

CCDC No = 1954919

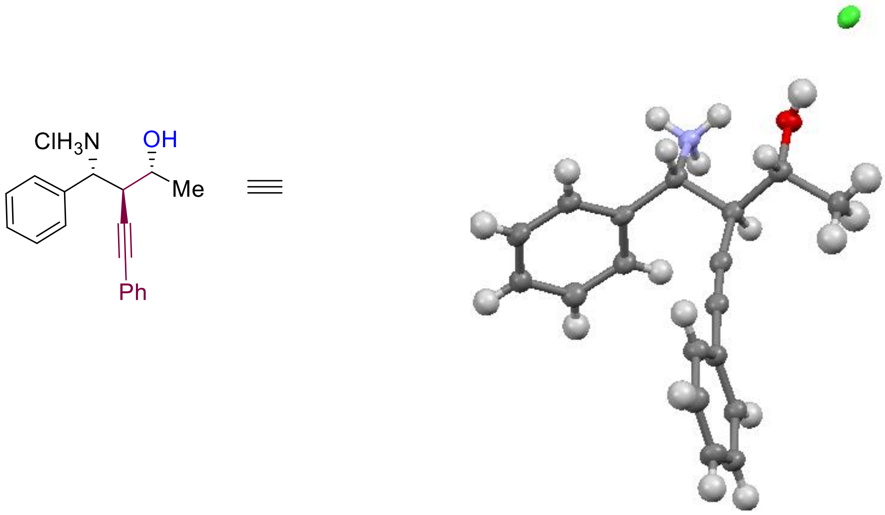

Bond precision: C-C = 0.0054 A Wavelength=1.54184

Cell: a=7.7707(1) b=5.9253(1) c=17.7543(3)

alpha=90 beta=97.859(2) gamma=90

Temperature: 101 K

Calculated Reported

Volume 809.80(2) 809.80(2)

Space group P 21 P 1 21 1

Hall group P 2yb P 2yb

Moiety formula C18 H20 N O, Cl Cl, C18 H20 N O

Sum formula C18 H20 Cl N O C18 H20 Cl N O

Mr 301.80 301.80

Dx,g cm-3 1.238 1.238

Z 2 2

Mu (mm-1) 2.062 2.062

F000 320.0 320.0

F000’ 321.50

h,k,lmax 9,7,22 9,7,22

Nref 3393[1866] 3243

Tmin,Tmax 0.781,0.848 0.322,1.000

Tmin’ 0.322

Correction method= # Reported T Limits: Tmin=0.322 Tmax=1.000

AbsCorr = MULTI-SCAN

Data completeness= 1.74/0.96 Theta(max)= 75.878

R(reflections)= 0.0525(3131) wR2(reflections)= 0.1557(3243)

S = 1.147 Npar= 192

Single crystal structure of **7d'**

CCDC No = 1952977

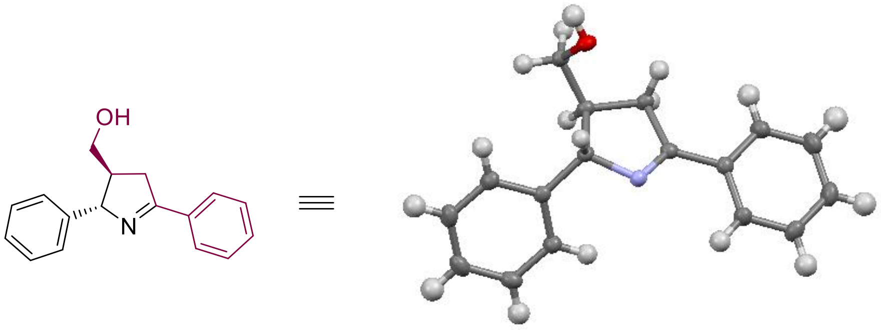

Bond precision: C-C = 0.0016 A Wavelength=1.54184

Cell: a=11.9127(1) b=10.0257(1) c=11.2966(1)

alpha=90 beta=92.862(1) gamma=90

Temperature: 100 K

Calculated Reported

Volume 1347.51(2) 1347.51(2)

Space group P 21/c P 1 21/c 1

Hall group -P 2ybc -P 2ybc

Moiety formula C17 H17 N O C17 H17 N O

Sum formula C17 H17 N O C17 H17 N O

Mr 251.32 251.31

Dx,g cm-3 1.239 1.239

Z 4 4

Mu (mm-1) 0.599 0.599

F000 536.0 536.0

F000' 537.47

h,k,lmax 14,12,14 14,12,14

Nref 2813 2794

Tmin,Tmax 0.924,0.948 0.921,1.000

Tmin' 0.877

Correction method= # Reported T Limits: Tmin=0.921 Tmax=1.000

AbsCorr = MULTI-SCAN

Data completeness= 0.993 Theta(max)= 75.985

R(reflections)= 0.0372(2642) wR2(reflections)= 0.0944(2794)

S = 1.049 Npar= 174

References

[1] J. E. M. Lewis, F. Modicom, S. M. Goldup, *J. Am. Chem. Soc.* **2018**, *140*, 4787-4791

[2] A. Bahamonde, B. Al Rifaie, V. Martín-Heras, J. R. Allen, M. S. Sigman, *J. Am. Chem. Soc.* **2019**, *141*, 8708-8711.

[3] L. R. Reddy, Y. Waman, K. Nayak, K. Baharooni, S. Kotturi, *Org. Lett.* **2019**, *21*, 3481-3484.

[4] K. Shen, Q. Wang, *Chem. Sci.* **2017**, *8*, 8265-8270.

[5] L. Jiang, P. Cao, M. Wang, B. Chen, B. Wang, J. Liao, *Angew. Chem. Int. Ed.* **2016**, *55*, 13854-13858.
